# Metal–ligand–Lewis acid multi-cooperative catalysis: a step forward in the Conia-ene reaction†

**DOI:** 10.1039/d0sc05036a

**Published:** 2020-10-30

**Authors:** Arnaud Clerc, Enrico Marelli, Nicolas Adet, Julien Monot, Blanca Martín-Vaca, Didier Bourissou

**Affiliations:** Laboratoire Hétérochimie Fondamentale et Appliquée (UMR 5069), Université de Toulouse (UPS), CNRS 118 route de Narbonne F-31062 Toulouse France dbouriss@chimie.ups-tlse.fr bmv@chimie.ups-tlse.fr

## Abstract

An original multi-cooperative catalytic approach was developed by combining metal–ligand cooperation and Lewis acid activation. The [(SCS)Pd]_2_ complex featuring a non-innocent indenediide-based ligand was found to be a very efficient and versatile catalyst for the Conia-ene reaction, when associated with Mg(OTf)_2_. The reaction operates at low catalytic loadings under mild conditions with HFIP as a co-solvent. It works with a variety of substrates, including those bearing internal alkynes. It displays complete 5-*exo vs.* 6-*endo* regio-selectivity. In addition, except for the highly congested ^*t*^Bu-substituent, the reaction occurs with high *Z vs. E* stereo-selectivity, making it synthetically useful and complementary to known catalysts.

## Abstract 2

An original multi-cooperative catalytic approach was developed by combining metal–ligand cooperation and Lewis acid activation.

## Graphical abstract

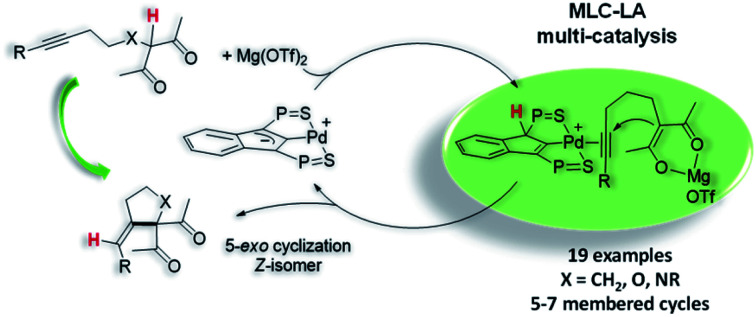

## Introduction

Major breakthroughs have been made in homogeneous catalysis thanks to cooperative approaches.^1^ Concomitant and synergistic participation of several active sites, as in enzymes, is very attractive to achieve chemical transformations under milder conditions and reduce wastes. Thanks to cooperativity, not only the efficiency and selectivity of catalysts can be improved, but the diversity and complexity of the obtainable chemical compounds can also be expanded. In this context, metal–ligand cooperativity (MLC) is clearly prominent.^2^ Here, a ligand participates directly in substrate activation, in addition to modulating the stereoelectronic properties of the metal center. Complexes of the group 7–9 metals featuring non-innocent pincer ligands, in particular amido and pyridine-based scaffolds, occupy a forefront position in this area.^3^ Tremendous achievements in hydrogenation and dehydrogenation reactions have been reported with these catalysts.^4^ Extending the scope of transformations^5^ and developing the MLC catalytic approach further are currently a major endeavor, along with the diversification of complexes displaying MLC.

**Fig. 1:**
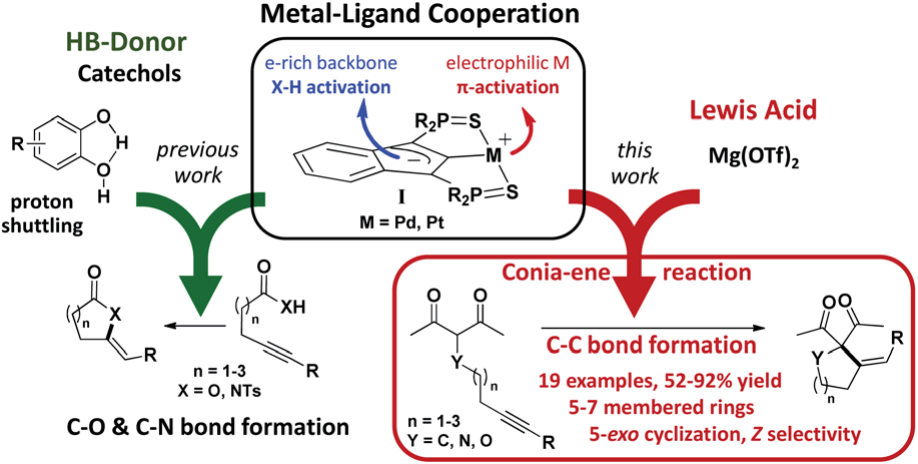
Multi-cooperative catalysis combining metal–ligand cooperation (MLC, indenediide-based (SCS)M pincer complexes) with hydrogen-bond donors (HBD, previous work) or Lewis acid activation (LA, this work) to promote cycloisomerization reactions (C–O, C–N and C–C bond formation).

Our group introduced a few years ago an SCS pincer framework featuring an electron-rich indenediide back-bone.^6^ The paucity of MLC behavior reported for Pd and Pt complexes,^7^ despite the prominent role of these metals in homogeneous catalysis, led us to study complexes of type I (Fig. 1). The electron-rich indenediide motif was shown to be non-innocent and to open a MLC pathway for Pd and Pt-catalyzed C–O and C–N bond formation. The (SCS) Pd,Pt complexes proved to very efficiently promote the cycloisomerization of alkynoic acids and *N*-tosyl alkynylamides, giving access in high yield and selectivity to a wide variety of alkylidene lactones and lactams, including seven-membered rings.^8^ Of note, indepth mechanistic studies substantiated the participation of proton shuttling in addition to MLC.^9^ This drove us to use catechols as hydrogen bond donor (HBD) additives. The activity and selectivity of the (SCS) Pd,Pt complexes were thereby spectacularly improved.^9^ The synergy between MLC and proton shuttling is a very appealing approach to boost catalytic performance. It has been proposed for many hydrogenation/dehydrogenation reactions involving MLC^10^ and recently unequivocally demonstrated by Hazari *et al.*^11^ Its impact in intermolecular hydroamination reactions has also been substantiated.^7*g*^

Encouraged by these results, we wondered about the possibility of developing new multi-cooperative catalytic approaches based on MLC. Another aim was to expand the scope of SCS Pd,Pt catalysts to more challenging transformations. In this work, we focused on Conia-ene cyclizations, *i.e.* the formation of carbo- and heterocycles by C–C coupling between an enolizable group, typically a β-dicarbonyl moiety, and an alkyne. As pointed out by Enders *et al.* in their recent review,^12^ this is a very powerful transformation and much progress has been achieved recently from both methodological and synthetic viewpoints, but several limitations still need to be addressed. While investigating the ability of the MLC Pd indenediide complex to catalyze Conia-ene cyclizations, we discovered that it is extremely efficient and versatile when associated with a Lewis acid (LA). The combined use of TM catalysts and Lewis acids (often referred to as dual activation) has attracted increasing interest and led to impressive developments over the last decade,^13^ including in the Conia-ene reaction.^12,14^ Yet, synergistic effects between MLC and LA activation have not been realized so far. As reported hereafter, the association of [(SCS)Pd]_2_ and Mg(OTf)_2_ provides a first example of such a dual and multi-cooperative catalytic system. It is a very efficient and useful catalyst for Conia-ene cyclizations, displaying broad substrate scope and high regio as well as stereo-selectivity.

## Results and discussion

**Scheme 1:**
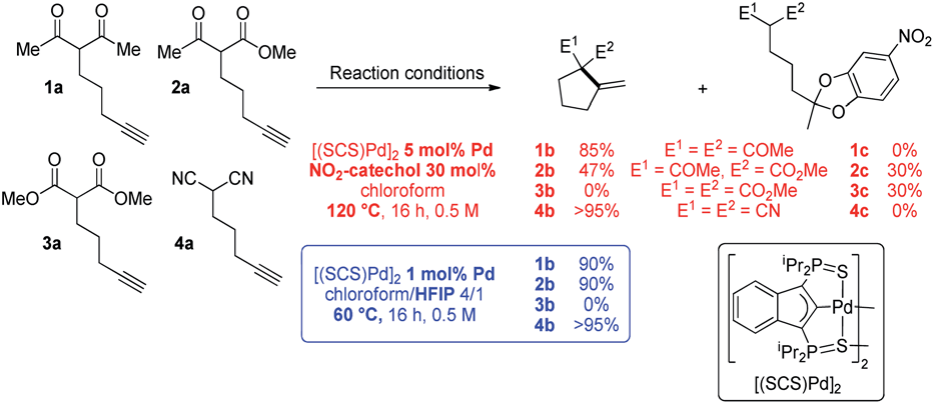
Conia-ene cyclization of the β-dicarbonyl and β-dinitrile substrates 1a–4a catalyzed by the [(SCS)Pd]_2_ complex in the presence of 4-nitrocatechol or HFIP. Throughout the paper, the a/b letters in the compound numbers refer to the substrate/cyclized Conia-ene product, respectively.

To start with, the carbo-cyclization of the β-dicarbonyl compounds 1a–3a and β-dinitrile 4a bearing an alkyne moiety was studied applying the reaction conditions previously optimized for the cycloisomerization of alkynoic acids (Scheme 1).^9^ A series of hydrogen-bond donors (catechols…, 30 mol%, Table S1†)^15^ were probed as proton shuttling additives to the indenediide-derived Pd complex [(SCS)Pd]_2_ (5 mol% Pd). The best results were obtained with 4-nitrocatechol. Heating at 120 °C for 16 h, full conversion could be achieved in all cases but 3a (only 30% in this case). The expected cyclized products 1b and 4b were selectively obtained from the β-diketone and β-dinitrile substrates 1a and 4a, whereas 2a led to the formation of a secondary product 2c (30% yield) resulting from double addition of the catechol to the C≡C triple bond,^16^ besides the targeted carbocycle 2b (47% yield). As for the β-diester 3a, no cyclized product 3b was formed, only the secondary product 3c was obtained.

The known ability of hexafluoroisopropanol (HFIP) to form hydrogen-bond networks and participate in proton shuttling^17^ prompted us to screen a series of fluorinated alcohols as additives. This resulted in noticeable improvement of activity and selectivity (Scheme 1 and Table S2,† entries 1–4).^15^ Using a 4/1 chloroform/HFIP solvent mixture, high conversions of the β-diketone 1a and β-dinitrile 4a were achieved in this case at 60 °C with only 1 mol% of Pd (entry 6). The fact that 4a was readily cyclized is particularly noteworthy since β-dinitriles are usually significantly less reactive than β-dicarbonyl substrates in the Conia-ene reaction.^12^ It is because the linear geometry of the cyano group is not suited for intramolecular hydrogen-bonding and thus tautomerization is more demanding. Under these milder conditions, the β-keto,ester 2a could also be efficiently cyclized and 2b was formed in high yield without a side product. In turn, the β-diester 3a was unreactive. Due to their weaker acidity, β-diester substrates are notoriously less reactive and more challenging in the Conia-ene reaction.^12^ The contribution of proton shuttling to the carbocyclization of 1a, 2a and 4a in the presence of HFIP is supported by a strong solvent dependence. While toluene and dichloromethane gave results close to those obtained in chloroform, hydrogen-bond accepting solvents such as THF or DMSO completely inhibited the reaction.

**Fig. 2:**
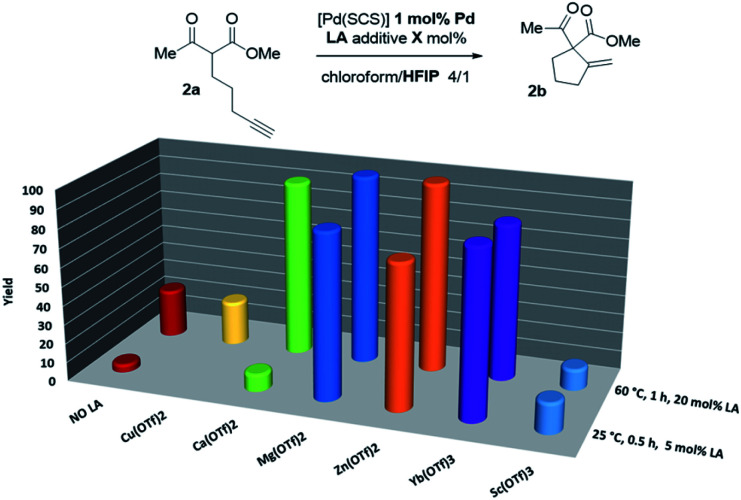
Evaluation of the catalytic activity of the Pd complex [(SCS)Pd]_2_ in the presence of LA additives.

Thus, the indenediide pincer Pd complex [(SCS)Pd]_2_ is a competent catalyst for the Conia-ene reaction of β-diketo, β-keto,ester and β-dinitrile substrates, when combined with HFIP as a co-solvent. However, it fails to cyclize the corresponding β-diester under the same conditions. Although the [(SCS)Pt]_2_ complex proved to be superior for the cyclization of challenging alkynoic acids and amides,^8*c*^ no improvement was observed in the Conia-ene reaction upon changing Pd for Pt. The Pt complex proved to be in fact less active, with only 40% (*vs.* 90%) conversion of 2a after 16 h (Table S2†).^15^ Looking for another way to boost the catalytic activity, we envisioned to use di or trivalent Lewis acids, which are known to activate β-dicarbonyl compounds and facilitate the formation of the corresponding enols. Combinations of hard and soft Lewis acids have been occasionally used in Conia-ene reactions. Such dual activation proved in particular successful to promote enantioselective cyclization of β-diketo and β-keto,ester derivatives.^14*a*–*c*,*f*^ Given these precedents, we wondered about the possibility of implementing a new type of multi-cooperative catalytic approach by merging metal–ligand cooperation, as achieved by the (SCS) Pd complex, with the activation of the β-dicarbonyl moiety by a LA.^18^ Multi-cooperative catalytic systems are very attractive but challenging to achieve. They inherently raise compatibility issues and the different components must be carefully adjusted to act synergistically. In the case of the (SCS) Pd complex, the Lewis acid additive may interact with the electron-rich indenediide motif as well as the sulfur atoms and thereby interfere with the Pd–ligand cooperativity.^19^ A series of triflate salts were evaluated as LA additives to [(SCS)Pd]_2_ in the cyclization of the β-keto,ester 2a in 4/1 chloroform/HFIP as solvent. Initial screenings were performed at 60 °C with 1 mol% Pd and 20 mol% LA (Fig. 2 and Table S3†).^15^ While Cu(OTf)_2_ and Sc(OTf)_3_ had no or negative impact on catalysis, significant improvement of activity was observed with Ca(ii), Mg(ii), Zn(ii) and Yb(iii) salts.^20^ High conversions were achieved in only one hour (>82% *vs.* only 25% without a LA), demonstrating the compatibility and synergy between MLC with the [(SCS)Pd]_2_ complex and electrophilic activation of the β-dicarbonyl moiety with a LA. Taking advantage of the catalytic boost triggered by the LA additive, milder reaction conditions were then used. The temperature was decreased to 25 °C and the LA loading was reduced to 5 mol%. Under these mild conditions, Mg(OTf)_2_ and Yb(OTf)_3_ led to the best results (∼90% conversion to 2b within only 30 min, and >95% after 1.5 h). The β-diketo substrate 1a was also readily cyclized under these conditions (>95% after 8 h). In contrast, similar results were obtained with and without Lewis acids in the case of the β-dinitrile 4a (85% conv. is achieved in 16 h at 25 °C). Chelating coordination of the two cyano groups is not possible geometrically, and thus the LA cannot facilitate deprotonation, as in the case of the β-dicarbonyl substrates. Most remarkably, the combination of [(SCS)Pd]_2_ with the LA allowed for the cyclization of the challenging β-diester 3a as well. Here, Mg(OTf)_2_ significantly outperformed Yb(OTf)_3_ (83 *vs.* 13% conversion of 3a within 30 min). Given the superior efficiency, abundance and biological tolerance of Mg over Yb, catalytic studies were continued with Mg(OTf)_2_ as the LA.^21^

To ascertain the need and synergy of all the catalytic components, a series of control reactions were carried out for the cyclization of the β-keto,ester substrate 2a. The results obtained under conditions deviating from the optimal ones [1 mol% Pd, 5 mol% Mg(OTf)_2_, chloroform/HFIP 4 : 1, 25 °C] are disclosed in Table 1. As previously pointed out, the presence of HFIP and Mg(OTf)_2_ is crucial to boost the catalytic activity and makes the Conia-ene reaction work at 25 °C with low Pd loadings. Conversely, in the absence of the Pd complex, no cyclized product (or only a very small amount) was formed with HFIP alone or with a mixture of Mg(OTf)_2_ and HFIP. The electron-rich indenediide moiety of the SCS ligand also plays a major role, as substantiated by the noticeable decrease in activity observed when shifting to the corresponding 1-Me-2-indenyl Pd complex.

**Table 1:** Impact of the different components of the catalytic system (Pd complex, Lewis acid, and proton shuttle additive) on the carbocyclization of the keto,ester 2a

Reaction conditions, deviations from the optimal	Conv./reaction time
**Optimal: [(SCS)Pd]**_**2**_ **1 mol% Pd, 5 mol% Mg(OTf)**_**2**_ **chloroform/HFIP 4/1, 25 °C**	**>95%/1.5 h**
[(SCS)Pd]_2_**5** mol% Pd, **20** mol% Mg(OTf)_2_ chloroform, 25 °C, **no HFIP**	46%/60 h
**No Mg salt, 60 °C**	>95%/22 h
**No Pd complex, no Mg salt, 60 °C**	Traces/16 h
**No Pd complex, 20 mol% Mg(OTf)**_**2**_	0%/2 h
**Indenyl [(SCS-Me)Pd] complex**	>95%/8.5 h
**Indenyl [(SCS-Me)Pd] complex, 5 mol% Pd, no Mg salt, 60 °C**	90%/15 days
**[PdCl(allyl)]**_**2**_ **1 mol% Pd**	6%/24 h
**[PdCl**_**2**_ **(PhCN)**_**2**_ **] 1 mol% Pd**	7%/24 h
**[PdCl**_**2**_ **(PhCN)**_**2**_ **] 1 mol% Pd, dppe 1 mol%**	35%/24 h
**[PdCl**_**2**_ **(PhCN)**_**2**_ **] 1 mol% Pd, dppe 1 mol%,** ^**i**^ **Pr**_**2**_ **EtN 1 mol%**	78%/24 h
**[(SCS)** ^**Ph**^ **Pd]**_**3**_ **5 mol% Pd, 20** mol% Mg(OTf)_2_	>95%/20 min
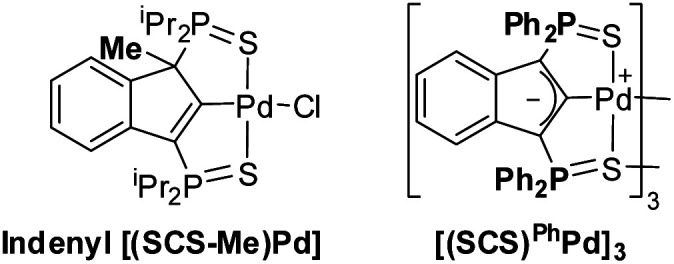	

In addition, much lower conversions were observed with [PdCl(allyl)]_2_ and [PdCl_2_(PhCN)_2_] (6–7% after 24 h), as well as [PdCl_2_(dppe)] (35%). Even the combination of [PdCl_2_(dppe)] with a base (^i^Pr_2_EtN, 1 mol%) performed poorly (78% conversion after 24 h) compared to the [(SCS) Pd] system (>95% in 1.5 h).

Seeking to evidence the non-innocent character of the ligand *via* the formation of indenyl [(SCS-H)PdX] species, several catalytic reactions were *in situ* analyzed by ^31^P NMR spectroscopy (see the ESI for a detailed description, Fig. S1–S4†). These monitorings were complicated by the fact that HFIP has an impact on the structure/association degree of the catalyst, and only broad signals could be observed (the dimer [(SCS)Pd]_2_ was recovered intact after evaporation of the solvent at the end of the catalytic reaction). When the reaction was carried out in the absence of HFIP, only the dimer [(SCS)Pd]_2_ was observed along the reaction, indicating that it is the resting state. This behaviour is reminiscent of that observed in the cycloisomerization of alkynoic acids,^9^ and is consistent with the tight association of the two (SCS)Pd fragments. We decided then to evaluate the behaviour of a pre-catalyst bearing Ph instead of ^i^Pr groups on the P atoms. The less electron-donating Ph_2_P=S sidearms led to weaker association of the (SCS)^Ph^Pd fragments.^8*a*^ Only broad signals were again observed in the presence of HFIP, but when the reaction was carried out in the absence of HFIP, minor signals corresponding to indenyl species were clearly detected by ^31^P NMR spectroscopy. Although [(SCS)^Ph^Pd]_3_ remains the major species, the observation of indenyl species during the catalytic transformation is consistent with the indenediide ligand backbone acting as a base.

**Scheme 2:**
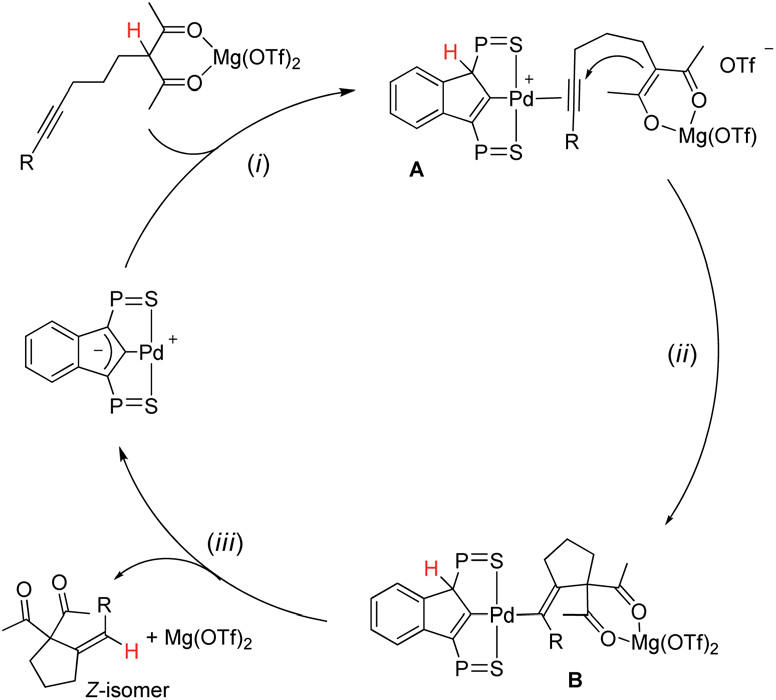
Catalytic cycle proposed to account for the Conia-ene reaction catalyzed by the (SCS)Pd complex and Mg(OTf)_2_ (the ^i^Pr groups at P are omitted for clarity).

A simplified 3-step mechanism for the Conia-ene reaction catalyzed by the association of [(SCS)Pd]_2_ and Mg(OTf)_2_ is depicted in Scheme 2: (i) *double activation of the substrate*: the LA chelates the β-dicarbonyl moiety, the acidic proton is transferred to the electron-rich indenediide backbone of the SCS ligand while the alkyne coordinates to palladium (intermediate A); (ii) *cyclization*: the ensuing enolate adds to the π-activated C≡C triple bond to give the alkenyl Pd complex B; (iii) *product release*: back-transfer of the proton from the ligand backbone to the C(sp^2^) atom bonded to Pd results in proto-depalladation and regenerates the indenediide Pd catalyst. HFIP is presumed to help dissociate the dimeric pre-catalyst,^17*c*^ and to promote proton shuttling from the substrate to the indenediide moiety in step (i) and from the ligand backbone to the C(sp^2^) carbon at Pd in step (iii).

**Fig. 3:**
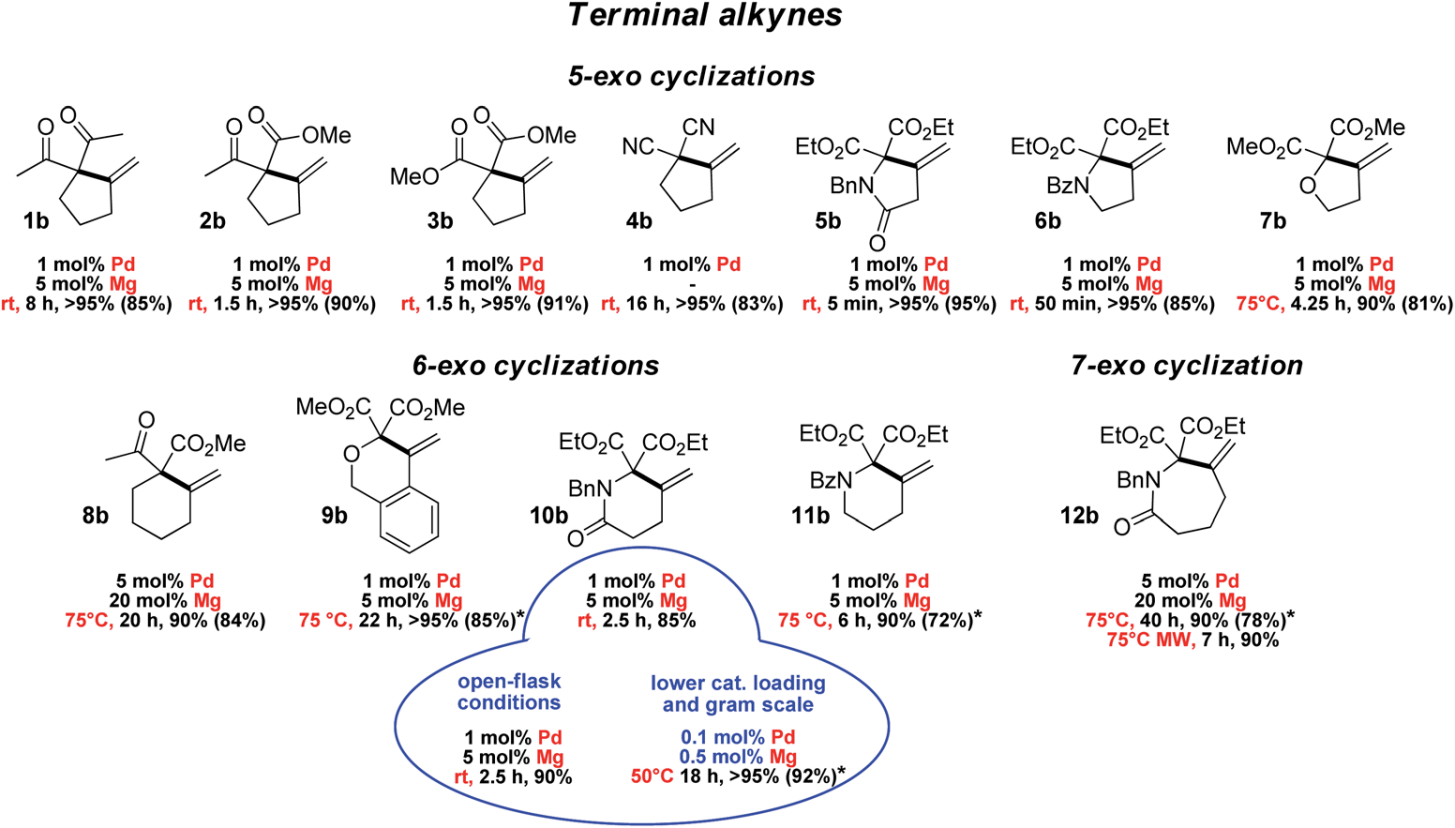
Substrate scope for the Conia-ene reaction catalyzed by the (SCS)Pd complex and Mg(OTf)_2_: β-dicarbonyl substrates bearing a terminal alkyne (isolated yields are indicated in parentheses; * stands for 0.1 M substrate concentration instead of 0.5 M).

The scope and functional group tolerance of the reaction were then explored (Fig. 3). Our aim was not only to probe the generality of the new MLC–LA dual catalytic approach, but also to improve the efficiency of the Conia-ene transformation and expand its synthetic interest. Substrates with a terminal alkyne and prone to 5-*exo* cyclization were investigated first. The (SCS)Pd–Mg association proved to be particularly efficient to cyclize β-diesters 5a–7a featuring an adjacent heteroatom (N or O). The amide-tethered substrate 5a was quantitatively cyclized in only 5 min at 25 °C. Shifting the amide moiety at the *exo* position (6a) slowed down the transformation somewhat, but it still proceeded rapidly and efficiently at 25 °C (>95% in 50 min). Cyclization of the ether-tethered substrate 7a was more challenging, but heating to 75 °C enabled to achieve high conversion (90%) in only 4.25 h.

The length of the linker between the β-dicarbonyl moiety and the terminal alkyne was then modified with the aim to access larger rings (8a–12a). 6-*Exo* cyclization of β-keto,esters such as 8a featuring a flexible butylene tether is tricky. It has only been scarcely achieved, using a Au(i) complex featuring a tris-alkynyl phosphine with bulky end caps (1 mol%, 25 °C, 1.5 h) or In(NTf_2_)_3_ (1 mol%, 80 °C, 4 h).^22,23^ Pleasingly, the methylene-cyclohexane product 8b could also be obtained efficiently using the [(SCS)Pd]_2_ complex (5 mol% Pd) and Mg(OTf)_2_ (20 mol%) within 20 h at 75 °C (90%). The dual catalytic system proved to be particularly efficient when the enol position bears an oxygen or nitrogen atom. Such substrates have been shown by Hatakeyama *et al.* to provide straightforward entry to valuable heterocycles and very good results in terms of activity and selectivity were obtained using In(OTf)_3_ with DBU (5 mol% each) in refluxing toluene.^24^ In our hands, the β-diester substrates 9a–11a reacted smoothly and high yields (>85%) were achieved with low catalytic loadings (1 mol% Pd, 5 mol% Mg). Particularly noteworthy is the formation of the δ-valerolactame 10b which was accomplished in only 2.5 h at 25 °C. Even 7-*exo* cyclization was possible and highly yielding. Using the same conditions as for 8a, the ε-caprolactame 12b was obtained in 90% yield within 40 h, and the reaction time could be reduced to 7 h using microwave irradiation.^25^ The preparation of such seven-membered rings was previously described using In(OTf)_3_/DBU as the catalyst, but in modest yield (41%) and with high catalytic loading (15 mol%).^24^

To assess the robustness of the Pd–Mg dual catalytic system, the cyclization of the amide-tethered substrate 10a was carried out under the optimized conditions (25 °C, 1 mol% Pd, 5 mol% Mg, chloroform/HFIP 4/1) but under open-flask conditions and with technical grade solvents. The δ-valerolactame 10b was obtained in similar yield to under inert conditions, demonstrating that the reaction does not require stringent precautions. Furthermore, when the catalytic loading was reduced by a factor of ten (0.1 mol% Pd and 0.5 mol% Mg), the product was still obtained in very high yield (>95% in 18 h at 50 °C). These reaction conditions were successfully applied to larger scale: 920 mg of pure product 10b were prepared using only 1.4 mg of [(SCS)Pd]_2_ and 4.5 mg of Mg(OTf)_2_. Merging metal–ligand cooperation and Lewis acid activation, as realized with the [(SCS)Pd]_2_ complex and Mg(OTf)_2_, thus appears general, practical and of synthetic value. It promotes 5, 6 and 7-*exo* cyclization of β-dicarbonyl substrates featuring terminal alkynes under mild conditions, and gives access to a variety of carbo- and heterocycles.

**Fig. 4:**
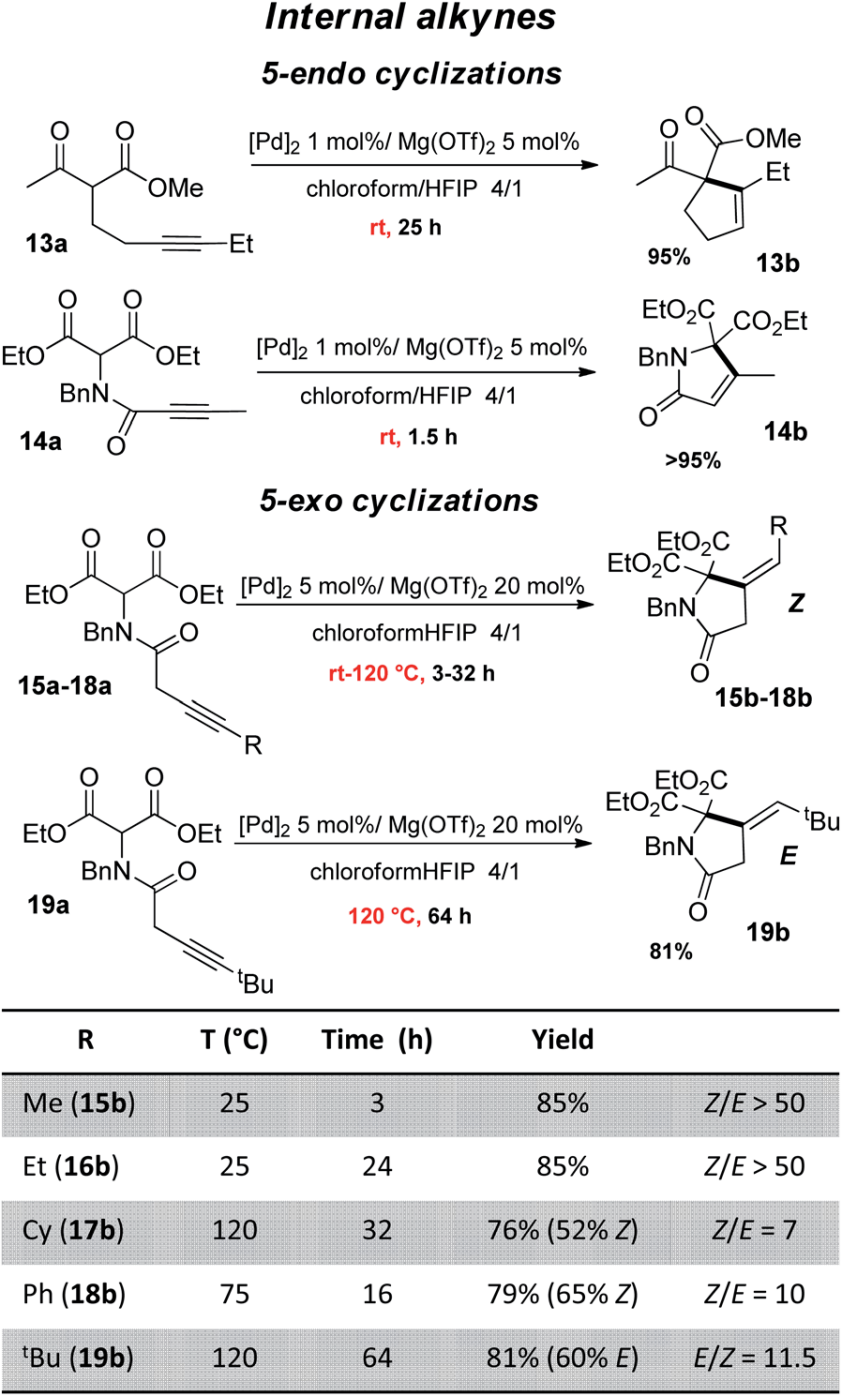
Conia-ene reaction catalyzed by the (SCS)Pd complex and Mg(OTf)_2_: β-dicarbonyl substrates bearing an internal alkyne (isolated yields in the *Z* isomer are indicated in parentheses).

The good performance of the dual Pd–Mg catalytic system prompted us to explore then the cyclization of substrates bearing internal alkynes, which are significantly more challenging than terminal alkynes (Fig. 4). Being way less reactive, these substrates require harsher conditions, which often results in regio-selectivity issues with the formation of mixtures of *exo* and *endo* cyclization products.^26^ Given the preference of 5-*endo* against 4-*exo-dig* cyclization, the β-keto,ester 13a and β-diester 14a were tested first. A longer reaction time was needed for 13a than for 14a (25 *vs.* 1.5 h) but in both cases, the reaction proceeded readily at 25 °C and afforded high yield (95%) of the 5-*endo* product. No trace of the less favored 4-*exo* product was detected by ^1^H NMR spectroscopy. Our dual catalytic system does not compete with (Ph_3_P)AuOTf for the formation of cyclopentene 13b (83% yield was achieved in only 5 min at room temperature using 1 mol% Au),^27^ but on the other hand, the gold catalyst works only with β-keto,esters and failed to cyclize β-diesters.^23^ As far as the cyclization of 14a is concerned, it had only been efficiently accomplished with Zn halides (10 mol%) but required high temperature and long reaction times (100 °C, 15 h).^28^ We then investigated 5-*exo* Conia-ene cyclization with internal alkynes (β-diester substrates 15a–19a). The terminal substituent was varied (R = Me, Et, Cy, Ph, ^*t*^Bu) to assess the impact of sterics and electronics. It should be noted that precedents of Conia-ene cyclization with internal alkynes mostly involve aryl and primary alkyl substituents, while examples with secondary alkyl groups are much rarer and tertiary ones are unprecedented.^12,24,27,29^ Due to the lower reactivity of these substrates, the catalytic loadings were fixed to 5 mol% Pd and 20 mol% Mg. For the less hindered substrates, with Me (15a) and Et (16a) substituents, cyclization occurred at 25 °C within 3 and 24 h, respectively, while those bearing Cy (17a), Ph (18a) and ^*t*^Bu (19a) groups required heating at 75–120 °C to achieve high conversions. For all substrates, including the ^*t*^Bu-substituted 19a, the reaction was highly selective. The structures of the obtained products were unambiguously determined thanks to thorough ^1^H and ^13^C NMR spectroscopic analyses including ADEQUATE, NOESY and dsel-HSQMC-IPAP experiments.^15^ Complete selectivity in favor of 5-*exo vs.* 6-*endo* cyclization was observed, whatever the substituent at the alkyne (alkyl or phenyl). For 15a and 16a, a single 5-*exo* product was obtained, authenticated as the *Z* isomer. For the substrates 17a and 18a bearing bulkier groups, which required higher reaction temperature, two 5-*exo* products were formed. In all cases, the *Z* isomer was very major (*Z*/*E* ratio from 7 to 10). In the case of the ^*t*^Bu group, opposite stereo-selectivity was observed. Steric congestion favored here the *E* product (*E*/*Z* of 11.5). Reaction monitoring indicated that it is not due to isomerization of 19b over prolonged heating, but the same *E*/*Z* ratio being observed from the beginning of the cyclization. From a mechanistic viewpoint, the *Z*-selectivity observed with 15a–18a is consistent with the outer-sphere approach outlined in Scheme 2. The enolate adds to the π-activated alkyne *trans* to Pd, resulting in the *Z* product after proto-depalladation.^30^ This *Z*-selectivity markedly contrasts with the *E*-selectivity induced by the In(OTf)_3_/DBU catalyst (with R = Me, ^n^Bu).^24^ In the latter case, the reaction was proposed to proceed by *syn* carbometalation, with the In(iii) center simultaneously activating the β-dicarbonyl and alkyne moieties.^31^ Formation of the *E* product 19b most likely results from isomerization of the vinyl-Pd intermediate B prior to proto-depalladation, due to steric congestion.^32^

## Conclusions

When combined with Mg(OTf)_2_ and HFIP, the indenediide-based SCS Pd pincer complex was found to be a very powerful catalyst for Conia-ene cyclizations. It works with a variety of substrates, β-keto,esters as well as β-dinitriles and β-diesters, and gives access with complete regio-selectivity to 5, 6 and 7-membered carbo- as well as heterocycles. It is robust and operates at low catalytic loadings (down to 0.1 mol% Pd and 0.5 mol% Mg). Most remarkably, it is also active and selective with substrates featuring internal alkynes. Whatever the substituent at the terminal position is (1°/2°/3° alkyl, aryl), 5-*exo* fully prevails over 6-*endo* cyclization. Moreover, except for the highly congested ^*t*^Bu product, the reaction occurs with high *Z*-selectivity. This behavior is in contrast with the *E*-selectivity observed with In-based catalysts, the other known catalysts for internal alkynes. This is due to the way the C–C bond formation and cyclization proceeds with the dual Pd/Mg catalyst; the Mg-chelated enolate attacks the π-coordinated C≡C triple bond trans to Pd.

These results expand the scope of MLC catalysis in terms of metal (the potential of Pd complexes in MLC catalysis is clearly under-exploited) and catalytic transformations (while hydrogenation/dehydrogenation processes have been extensively developed, examples of C–C bond forming reactions are very rare). Moreover, this work introduces a new multi-cooperative catalytic approach combining MLC and Lewis acid activation. The MLC/LA synergy parallels and complements that involving MLC and proton shuttling. It also represents a new type of dual activation, in which the TM catalyst displays MLC behavior (towards the enolizable C–H bond in this case) while the LA activates another reactive site of the substrate (the β-dicarbonyl motif). Future studies will seek to explore the generality of such MLC/LA synergy and to exploit it in other catalytic transformations.

## Conflicts of interest

There are no conflicts to declare.

## Supplementary Material

SC-012-D0SC05036A-s001
