# Supplementary material for: Metal–ligand–Lewis acid multi-cooperative catalysis: a step forward in the Conia-ene reaction
Source: Chem Sci. 2020 Oct 30;12(1):435–41. doi: 10.1039/d0sc05036a (PMC8178805; doi:10.1039/d0sc05036a)

Electronic Supplementary Material (ESI) for Chemical Science.
This journal is © The Royal Society of Chemistry 2020

SUPPORTING INFORMATION

Metal-Ligand-Lewis Acid Multi-Cooperative Catalysis: A Step Forward in the Conia-Ene Reaction

Arnaud Clerc,[#] Enrico Marelli,[#] Nicolas Adet, Julien Monot, Blanca Martín-Vaca,* Didier Bourissou*

GENERAL REMARKS S1

CONDITIONS SCREENING AND OPTIMIZATION, GENERAL PROCEDURE S2

$^{31}P\{^{1}H\}$ MONITORING OF CATALYTIC REACTIONS S5

SYNTHESIS OF PRECURSORS S8

GENERAL PROCEDURE A FOR THE FORMATION OF PRECURSORS: S8

GENERAL PROCEDURE B FOR THE FORMATION OF AMIDES: S8

GENERAL PROCEDURE C FOR THE FORMATION OF AMIDES: S8

CYCLIZED PRODUCTS S15

GENERAL PROCEDURE FOR THE CONIA-ENE CYCLISATION S15

REFERENCES S20

General Remarks

All reactions and manipulations were carried out under argon atmosphere using standard Schlenk techniques unless otherwise stated. Dry, oxygen–free solvents were employed. All organic reagents were obtained from commercial sources and used as received or prepared from known literature procedures. The complexes ([(SCS)^{Ph}Pd]$_3$, [(SCS-H)Pd], [(SCS)Pd]$_2$, [(SCS)Pt]$_2$ were synthesized using methods previously reported by our group.[1,2,3] Complex [(SCS-Me)Pd] was prepared using the same method reported for the corresponding phenyl derivative.[4] % of complexes for catalyst loadings are given relative to the effective [Pd] concentration. Preparative HPLC were performed using an Xbridge C18 150x19 mm column with a flowrate of 20 mL.min^{-1}. H_2O (0.1%

HCOOH) and acetonitrile (0.1% HCOOH) were used as solvents.^{1}H and ^{13}C spectra were obtained on a Bruker Avance 300, 400 or 500 MHz spectrometers. Chemical shifts are given in ppm relative to residual solvent as an internal standard ($CDCl_3$: δ = 7.26 ppm for ^{1}H and δ = 77.16 ppm for ^{13}C). Unless otherwise stated, NMR spectra were recorded at 293 K. When necessary, ADEQUATE, NOESY, NOESY ZQF selective analyses were used to determine the regio- and stereo-chemistry of the obtained products.

Conditions screening and optimization, general procedure

The model substrate (0.25 mmol), the additive and catalyst were dissolved in 0.5 mL of solvent in an NMR tube. The reaction was then heated at the indicated temperature for the corresponding time. Yields were determined by ^{1}H NMR analysis with 1,2,4,5-tetramethylbenzene as internal standard.

Table S1. Solvent and additive screening

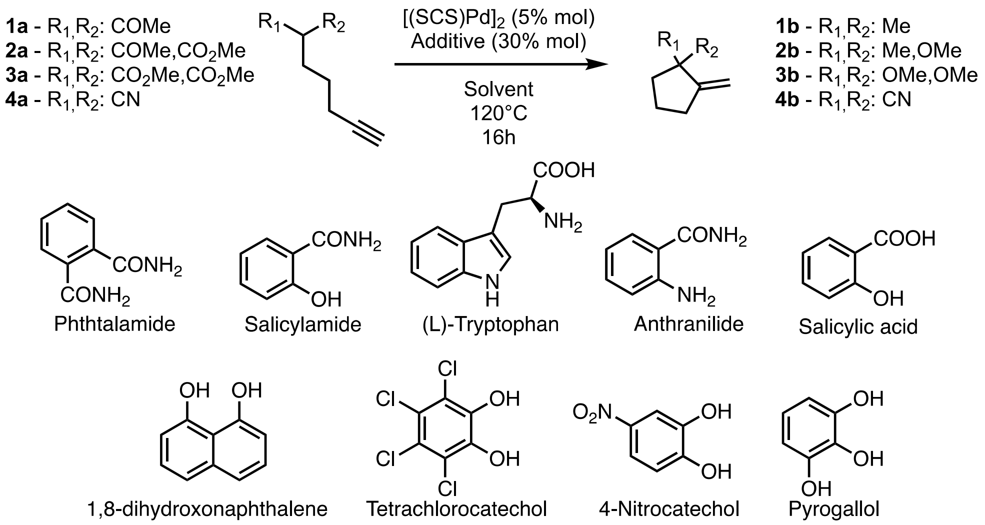

Entry	Substrate	Solvent	Additive	Yield
1	**2a**	$CDCl_3$	none	No reaction
2	**2a**	$CDCl_3$	Phthalamide	24
3	**2a**	$CDCl_3$	Salicylamide	14
4	**2a**	$CDCl_3$	(L)-Tryptophan	No reaction
5	**2a**	$CDCl_3$	Anthranilide	No reaction
6	**2a**	$CDCl_3$	Salicylic acid	27
7	**2a**	$CDCl_3$	1,8-dihydroxonaphthalene	50
8	**2a**	$CDCl_3$	Tetrachlorocatechol	23
9	**2a**	$CDCl_3$	Nitrocatechol	47[a]
10	**2a**	$CDCl_3$	Pyrogallol	50
11	**2a**	Toluene-d8	Nitrocatechol	63[a]
12	**2a**	DMSO-d6	Nitrocatechol	12
13	**2a**	Toluene-d8	Pyrogallol	61
14	**1a**	$CDCl_3$	Nitrocatechol	85
15	**3a**	$CDCl_3$	Nitrocatechol	0[a]
16	**4a**	$CDCl_3$	Nitrocatechol	>95

[a] ca. 30 mol% of product of addition of 4-nitrocatechol on the triple bond was detected by ^{1}H NMR

Table S2. Fluorinated additive and catalyst screening

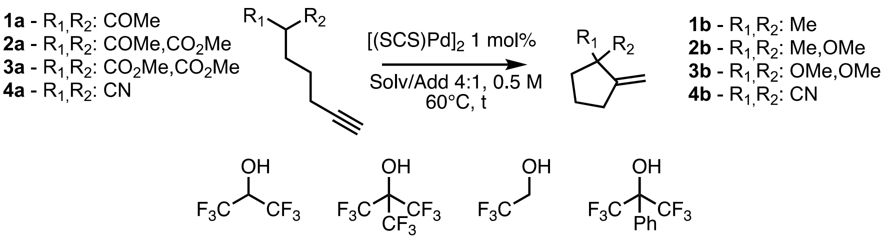

HFIP NFTB TFE Ph-HFIP

Entry	Substrate	Solvent	Catalyst	Additive	Time (h)	Yield
1	**2a**	$CDCl_3$	$[(SCS)Pd]_2$	NFTB	16	53[a]
2	**2a**	$CDCl_3$	$[(SCS)Pd]_2$	HFIP	16	90[a]
3	**2a**	$CDCl_3$	$[(SCS)Pd]_2$	HFIP	4	58[b]
4	**2a**	$CDCl_3$	$[(SCS)Pd]_2$	TFE	4	Traces
5	**2a**	$CDCl_3$	$[(SCS)Pd]_2$	Isopropanol	16	n.r.
6	**2a**	$CDCl_3$	$[(SCS)Pd]_2$	Ph-HFIP	16	n.r.
7	**2a**	$CDCl_3$	-	HFIP	16	Traces[c]
8	**2a**	$CDCl_3$	$[(SCS)^{Ph}Pd]_3$	HFIP	16	8
9	**2a**	$CDCl_3$	$[(SCS)Pt]_2$	HFIP	16	40
10	**2a**	THF-d8	$[(SCS)Pd]_2$	HFIP	16	n.r.
11	**2a**	DMSO-d6	$[(SCS)Pd]_2$	HFIP	16	n.r.
12	**2a**	Toluene-d8	$[(SCS)Pd]_2$	HFIP	16	90
13	**2a**	CD_2Cl_2	$[(SCS)Pd]_2$	HFIP	16	88
14	**1a**	$CDCl_3$	$[(SCS)Pd]_2$	HFIP	16	90
15	**3a**	$CDCl_3$	$[(SCS)Pd]_2$	HFIP	16	n.r.
16	**4a**	$CDCl_3$	$[(SCS)Pd]_2$	HFIP	16	>95

[a] T = 90°C; [b] 90% after 16h; [c] full conversion and ca. 80% yield at 120°C

Table S3. Lewis acid screening

2a - R_1,R_2: Me,OMe
3a - R_1,R_2: OMe,OMe

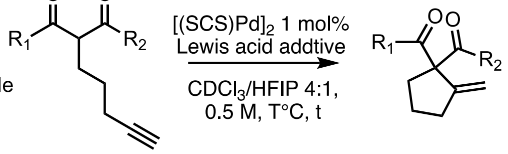

2b - R_1,R_2: Me,OMe
3b - R_1,R_2: OMe,OMe

5a

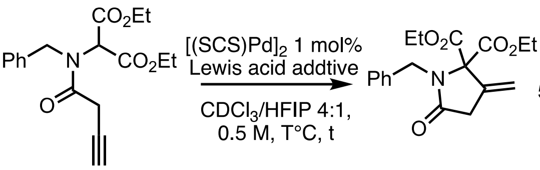

5b

Entry	Substrate	LA (mol %)	T °C	Time (h)	Yield
1	**2a**	none	60	1	25
2	**2a**	Sc(OTf)$_3$ (20)	60	1	11
3	**2a**	Cu(OTf)$_2$ (20)	60	1	23
4	**2a**	Ca(OTf)$_2$ (20)	60	1	92
5	**2a**	Mg(OTf)$_2$ (20)	60	1	>95
6	**2a**	Zn(OTf)$_2$ (20)	60	1	>95[a]
7	**2a**	Yb(OTf)$_3$ (20)	60	1	82[b]
8	**2a**	Ca(OTf)$_2$ (20)	40	1	24
9	**2a**	Mg(OTf)$_2$ (20)	40	1	>95
10	**2a**	none	25	0.5	4
11	**2a**	Zn(OTf)$_2$ (5), No Pd	25	0.5	27
12	**2a**	Zn(OTf)$_2$ (5)	25	0.5	76
13	**2a**	Ca(OTf)$_2$ (5)	25	0.5	9
14	**2a**	Mg(OTf)$_2$ (5)	25	0.5	87
15	**2a**	Yb(OTf)$_3$ (5)	25	0.5	88[b]
16	**2a**	Sc(OTf)$_3$ (5)	25	0.5	16
17	**3a**	Mg(OTf)$_2$ (5)	25	0.5	83
18	**3a**	Yb(OTf)$_3$ (5)	25	0.5	13[b]
19	**4a**	none	25	16h	>95
20	**4a**	Mg(OTf)$_2$ (5)	25	16h	>95
21	**5a**	Mg(OTf)$_2$ (5)	25	0.5	87
22	**5a**	Yb(OTf)$_3$ (5)	25	0.5	No reaction[b]

[a] The reaction was accomplished in 1h even in absence of Pd complex; [b] Direct NMR analysis was hampered due to distortion caused by the Yb salt, the yield reported is the isolated yield after work up.

Table S4. Evaluation of MLC in the absence (run 1-3) and presence (4-6) of Lewis acid

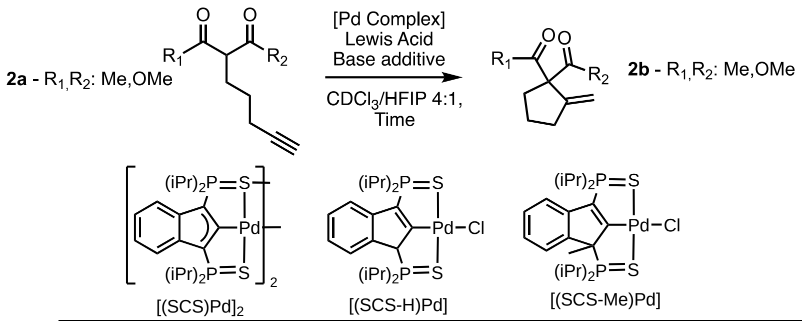

Run	Substrate	Complex (1 mol% Pd)	mol% Mg	Temperature (°C)	Time (h)	Yield
1	**2a**	[(SCS)Pd]2	none	60	22h	>95
2	**2a**	[(SCS-H)Pd]	none	60	12days	>95
3	**2a**	[(SCS-Me)Pd]	none	60	15days	89
4	**2a**	[(SCS)Pd]2	5	25	1h30	>95
5	**2a**	[(SCS-H)Pd]	5	25	8h30	>95
6	**2a**	[(SCS-Me)Pd]	5	25	8h30	>95
7	**2a**	[PdCl(allyl)]2	5	25	3h<br>24h	2<br>6
8	**2a**	[PdCl2(PhCN)2]	5	25	3h<br>24h	2<br>7
9	**2a**	[PdCl2(PhCN)2]<br>dppe (1 mol%)	5	25	3h<br>24h	2<br>35
10	**2a**	[PdCl2(PhCN)2]<br>dppe (1 mol%)<br>DIPEA (1 mol%)	5	25	3h<br>24h	11<br>78

$^{31}P\{^1H\}$ monitoring of catalytic reactions

Seeking to evidence the contribution of the indenediide ligand via the formation of indenyl species, several catalytic reactions under optimized conditions were monitored by ^{31}P NMR spectroscopy (Figure S1-S4). Upon addition of HFIP to a $CDCl_3$ solution of [(SCS)Pd]$_2$, the two singlet signals corresponding to the inequivalent P atoms of the dimeric complex (Figure S1, a) disappear to give rise to a broad signal at ca 85 ppm, in the typical region for indenyl-Pd species (b).[2] Upon addition of the substrate and Mg Lewis acid, the Conia-Ene reaction occurs, during which no significant change was observed in the ^{31}P spectrum (c). At the end of the reaction, two additional small broad signals appeared in the indenediide region (d). Evaporation of the HFIP/$CDCl_3$ mixture and addition of pure $CDCl_3$ resulted in the clean reformation of the dimeric [(SCS)Pd]$_2$, as evidenced by the two singlet signals depicted by the ^{31}P spectrum (e). This observation supports the robustness of the catalyst that is fully regenerated at the end of the reaction. When the reaction was carried out in the absence of HFIP, the sole species detected during the reaction was the pre-catalyst [(SCS)Pd]$_2$, it is the resting-state of the process. This behaviour is reminiscent of that observed in the cycloisomerization of alkynoic acids,[5] and is consistent with the tight association of the two (SCS)Pd fragments. We decided then to evaluate the behaviour of a pre-catalyst bearing Ph instead of ^{i}Pr groups on the P atoms. The less electron-donating Ph_2P groups lead to weaker association of the (SCS)^{Ph}Pd fragments,[1,2] supported by the observation of only one signal on the ^{31}P spectrum, despite its trimeric structure in the solid state (Figure S2).[1] Again, addition of HFIP to a $CDCl_3$ solution of [(SCS)^{Ph}Pd]$_3$

results in the disappearance of the initial signal in the ^{31}P spectrum (Figure S3, a), while a series of signals appears in the indenyl and indenediide regions (b). Addition of the substrate leads to the broadening of the signals (c,d) and at the end of the reaction, and $[(SCS)^{Ph}Pd]_3$ is fully regenerated after removal of HFIP (e). However, when the reaction was carried out in the absence of HFIP (Figure S4, a), minor indenyl Pd complexes could be clearly observed, although $[(SCS)^{Ph}Pd]_3$ remains the major species (a). Even if they are minor, the observation of these indenyl species during the catalytic transformation is consistent with the indenediide ligand backbone acting as a base.

Figure S1. NMR Monitoring of $[(SCS)Pd]_2$ under catalytic reaction conditions

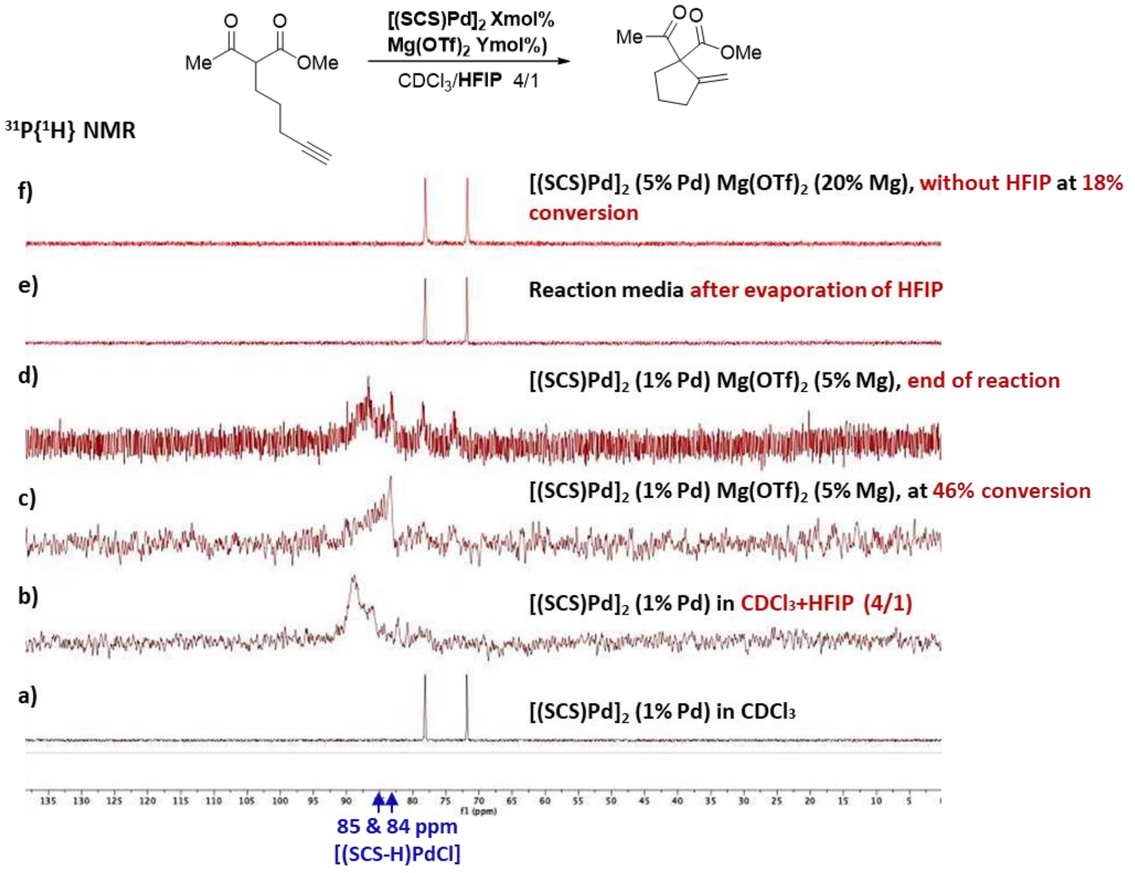

Figure S2. Structure of the complex $[(SCS)^{Ph}Pd]_3$

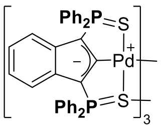

$[(SCS)^{Ph}Pd]_3$

Figure S3. NMR Monitoring of $[(SCS)^{Ph}Pd]_3$ under catalytic reaction conditions

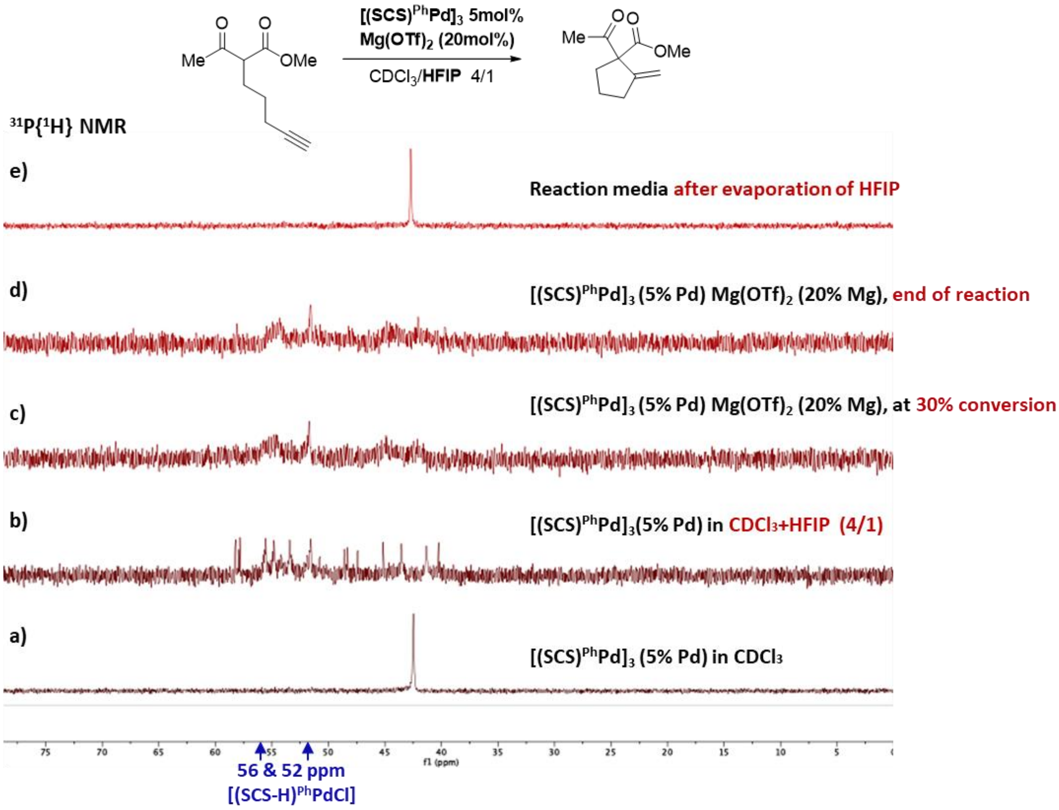

Figure S4. NMR Monitoring of $[(SCS)^{Ph}Pd]_3$ under catalytic reaction conditions without HFIP

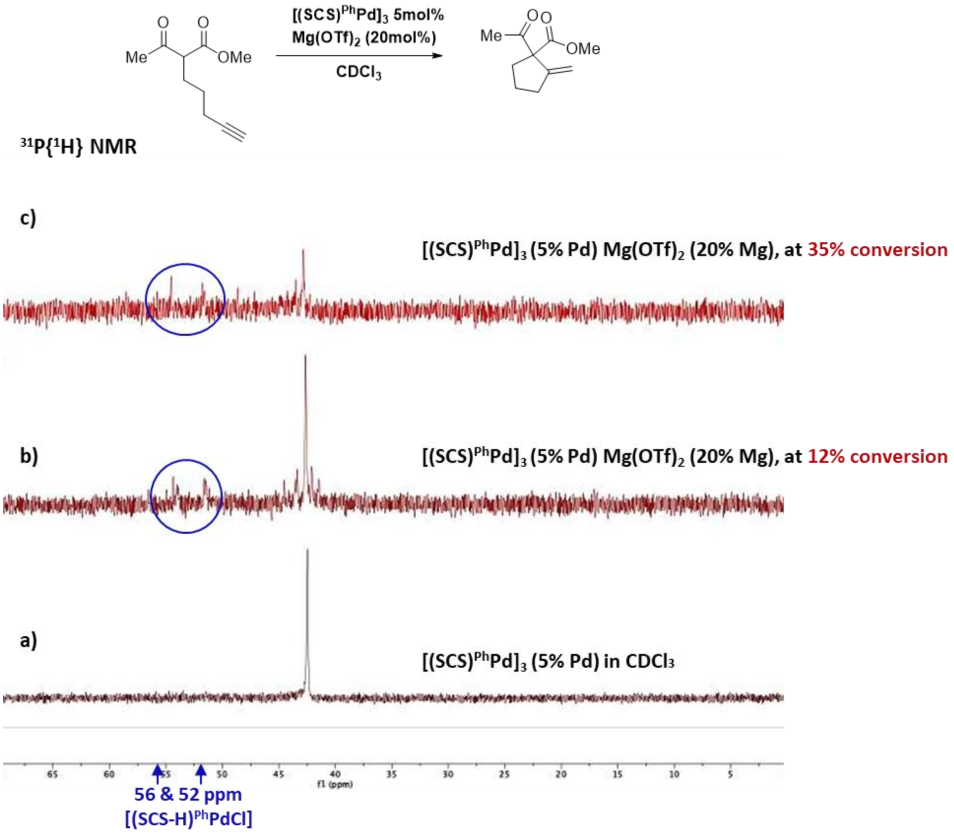

Synthesis of precursors

General procedure A for the formation of precursors:

The pro-nucleophile (1.2 eq) was added dropwise to a suspension of NaH (60% dispersed in mineral oil - 1.3 eq) in THF:DMF (1:1) (0.33 M) at 5°C. The reaction was then allowed to warm at to room temperature and stirred for 15 min. The electrophile was then added dropwise (1.0 eq) and the reaction media was heated at 85°C for 24 h. The volatiles were removed and the reaction was quenched with a saturated solution of $NH_4Cl_{(aq)}$ and EtOAc was added. The organic layer was washed with H_2O, dried over Na_2SO_4 and the solvent was removed *in vacuo* to yield the corresponding precursor that was either directly used or further purified by column chromatography.

General procedure B for the formation of amides:

Couple of drops of DMF was added to a stirred solution of the corresponding carboxylic acid (1.2 equiv) in DCM (1 M). Then oxalyl chloride (1.1 equiv) was added dropwise at room temperature. The reaction media was allowed to stir for 1h (Caution: bubbling of CO_2 and CO occurs). The crude acyl chloride solution was then added to a strongly stirred mixture of the corresponding amine (1.0 equiv) in DCM (0.67 M) and $NaHCO_{3(sat)}$ (1.0 $mL.mmol^{-1}$). After 1h, the solution was filtered through a silica pad using Et_2O as eluent to yield the crude amide. Further purification was achieved by column chromatography.

General procedure C for the formation of amides:

The method is a modified version of Sammakia's procedure.[6] To a solution of the corresponding *t*Bu ester (1.0 equiv) in $SOCl_2$ (10.0 equiv), H_2O (1.0 equiv) was added and the resulting mixture was allowed to stir overnight at room temperature. Then, the thionyl chloride was removed *in vacuo* or by distillation. The residual amount of $SOCl_2$ was removed by azeotropic distillation with toluene (repeated three times). After complete removal of solvent, the crude acyl chloride was dissolved in DCM (1 M) and added to a solution of the corresponding amine (1.0 equiv) in DCM (0.67 M) and $NaHCO_{3(sat)}$ (1.0 $mL.mmol^{-1}$). After 1h, the solution was filtered through a silica pad using Et_2O as eluent to yield the crude amide. Further purification was achieved by column chromatography.

*3-(pent-4-yn-1-yl)pentane-2,4-dione - **1a** - CAS [88459-71-6]:*

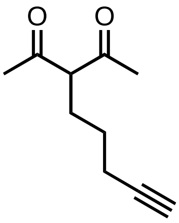

Using general procedure **A**, with acetylacetone (1.2 g - 12 mmol) and 5-chloropent-1-yne (1.16 g - 10 mmol) pure 3-(pent-4-yn-1-yl)pentane-2,4-dione (549 mg - 48% yield) was obtained as a clear oil after purification via column chromatography (95:5 Pentane:EtOAc). Mixture of keto-enol tautomers. ^{1}H NMR (300 MHz, $CDCl_3$): δ = 3.64 (t, *J* = 7.2 Hz, 1H, -CH-$(COMe)_2$), 2.39 - 2.32 (m, 1H, CH_a-CH-$(COMe)_2$), 2.29 - 2.20 (m, 2H, -CH_2-C≡), 2.19 (s, 3H, CH_3), 2.16 (s, 3H, CH_3), 2.03 - 1.90 (m, 2H, CH_b-CH-$(COMe)_2$, C≡CH), 1.68 – 1.40 (m, 2H, CH_2-CH_2-CH_2). Spectroscopic data in accordance with literature.[7]

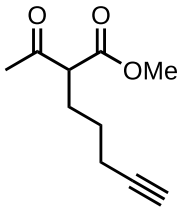

*Methyl 2-acetylhept-6-ynoate - **2a** - CAS [131190-10-8]:*

Using General procedure **A**, with methyl 3-oxobutanoate (1.17 g - 10 mmol) and 5-chloropentyne (975 mg - 8.4 mmol) pure Methyl 2-acetylhept-6-ynoate (1.01 g - 66% yield) was obtained as a clear oil after purification via column chromatography (95:5 Pentane:EtOAc). Mixture of keto-enol tautomers. ^{1}H NMR (300 MHz, $CDCl_3$): δ = 3.74 (s, 3H, CH_3-O), 3.46 (t, *J* = 7.4 Hz, 1H, CO_2Me-CH-COMe), 2.23 (s, 3H, CH_3-C=O), 2.26 – 2.17 (m, 2H, -CH_2-C≡), 2.06 – 1.90 (m, 3H, -CH-CH_2-CH_2; C≡CH), 1.62 – 1.42 (m, 2H, CH_2-CH_2-CH_2). Spectroscopic data in accordance with literature.[8]

*Dimethyl 2-(pent-4-yn-1-yl)malonate - **3a** - CAS [130905-55-4]:*

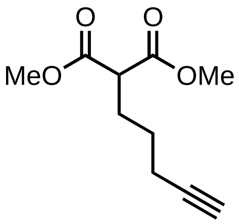

Using General procedure **A**, with dimethyl malonate (653 mg - 5 mmol) and 5-chloropentyne (480 mg - 4.1 mmol) pure dimethyl 2-(pent-4-yn-1-yl)malonate (609 mg - 72% yield) was obtained as a clear oil after purification via column chromatography (90:10 Cyclohexane:EtOAc). ^{1}H NMR (300 MHz, $CDCl_3$): δ = 3.74 (s, 6H, $(OCH_3)_2$), 3.39 (t, *J* = 7.5 Hz, 1H, CH- $(CO_2Me)_2$), 2.23 (td, *J* = 7.0, 2.6 Hz, 2H, -CH_2-C≡CH), 2.08 – 1.98 (m, 2H, -CH-CH_2-CH_2), 1.96 (t, *J* = 2.6 Hz, 1H, C≡CH), 1.62 – 1.50 (m, 2H, CH_2-CH_2-CH_2). Spectroscopic data in accordance with literature.[9]

*2-(pent-4-yn-1-yl)malononitrile - **4a** - CAS [106814-30-6] :*

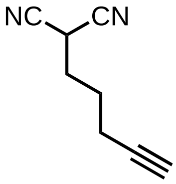

Using general procedure **A**, with malonitrile (680 mg - 10.3 mmol) and 5-chloropent-1-yne (1 g - 8.6 mmol) pure 2-(pent-4-yn-1-yl)malononitrile (549 mg - 48% yield) was obtained as a clear oil after purification via column chromatography (70:30 Pentane:DCM). ^{1}H NMR (300 MHz, $CDCl_3$): δ = 3.83 (t, *J* = 7.0 Hz, 1H, CH-$(CN)_2$), 2.36 (td, *J* = 6.6, 2.7 Hz, 2H, -CH_2-C≡), 2.22 – 2.10 (m, 2H, -CH-CH_2-CH_2), 2.05 (t, *J* = 2.7 Hz, 1H, C≡CH), 1.87 – 1.72 (m, 2H, CH_2-CH_2-CH_2). Spectroscopic data in accordance with literature.[9]

Diethyl 2-(benzylamino)malonate - CAS [56599-00-9] :

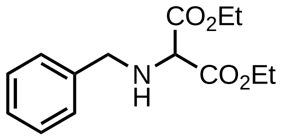

Freshly ground K_2CO_3 (10.36 g - 75 mmol - 1.0 eq) was added to a solution of benzylamine (8.2 mL - 75 mmol - 1.0 eq) and diethylbromomalonate (12.81 mL - 75 mmol - 1.0 eq) in acetonitrile (500 mL). The resulting suspension was heated at reflux overnight. The next day the volatiles were removed. DCM (100 mL) and H_2O (200 mL) were then added and the organic layer was separated. The aqueous layer was then extracted with DCM (2x100 mL). The combined organic layers were dried over Na_2SO_4 and the solvent was removed *in vacuo*. Pure diethyl 2-(benzylamino)malonate (5.97 g - 30% yield) was obtained as a clear oil after purification by column chromatography (using a gradient from 100:0 to 70:30 of Pentane:EtOAc). ^{1}H NMR (300 MHz, $CDCl_3$): δ = 7.41 – 7.22 (m, 5H, H_{arom}), 4.23 (qd, *J* = 7.1, 0.9 Hz, 4H, 2xCH_2-CH_3), 4.05 (s, 1H, CH-CO_2Et), 3.81 (s, 2H, Ph-CH_2-NH), 2.08 (bs, 1H, NH), 1.28 (t, *J* = 7.1 Hz, 6H, 2xCH_3-CH_2). Spectroscopic data in accordance with literature.[10]

*Diethyl 2-(N-benzylbut-3-ynamido)malonate - **5a** - CAS [1383445-11-1] :*

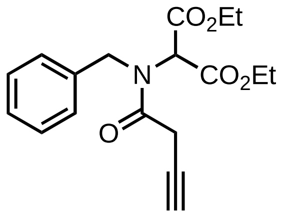

Using the general procedure **B**, with but-3-ynoic acid (250 mg - 2.97 mmol) and diethyl 2-(benzylamino)malonate (657 mg - 2.47 mmol). The corresponding diethyl 2-(*N*-benzylbut-3-ynamido)malonate (439 mg - 53% Yield) was obtained as an orange oil together with cyclized side product (20%). Mixture of 2 rotamers at room temperature (89%:11%), only the major rotamer is assigned. ^{1}H NMR (300 MHz, $CDCl_3$): δ = 7.43 – 7.13 (m, 5H, H_{arom}), 5.44 (s, 1H, CH-CO_2Et), 4.79 (s, 2H, Ph-CH_2-N), 4.26 – 4.01 (m, 4H, 2xCH_2-CH_3), 3.32 (d, *J* = 2.7 Hz, 2H, -CH_2-C≡CH), 2.23 (t, *J* = 2.7 Hz, 1H, -C≡CH), 1.21 (t, *J* = 7.1 Hz, 6H, 2xCH_2-CH_3). Spectroscopic data in accordance with literature.[11]

Diethyl 2-(but-3-yn-1-ylamino)malonate - CAS [1258538-10-1]:

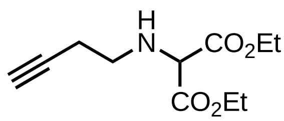

Diethyl bromomalonate (1.16 mL - 6.81 mmol - 1.0 eq) was added to a solution of 1-amino-3-butyne (471 mg - 6.81 mmol - 1.0 eq), NEt_3 (1.1 mL - 8.17 mmol - 1.2 eq) in $CHCl_3$ (12 mL). The resulting solution was heated to 55°C for 48 h. It was then allowed to cool down to room temperature and quenched by addition of 1M NaOH (40 mL). The layers were then separated and the aqueous layer extracted 3 times with EtOAc. The combined organic layers were dried over Na_2SO_4 and the solvent removed *in vacuo*. Pure diethyl 2-(but-3-yn-1-ylamino)malonate (330 mg - 21% Yield) was obtained after purification via column chromatography (80:20 Pentane:EtOAc). ^{1}H NMR (300 MHz, $CDCl_3$): δ = 4.24 (q, *J* = 7.1, 1.1 Hz, 4H, 2xCH_2-CH_3), 4.08 (s, 1H, CH-CO_2Et), 2.79 (t, *J* = 6.8 Hz, 2H, CH_2-NH), 2.41 (td, *J* = 6.8, 2.7 Hz, 2H, CH_2-C≡), 2.36 (bs, 1H, NH), 2.01 (t, *J* = 2.7 Hz, 1H, C≡CH), 1.29 (t, *J* = 7.1 Hz, 6H, 2xCH_3-CH_2). Spectroscopic data in accordance with literature.[12]

*Diethyl 2-(N-(but-3-yn-1-yl)benzamido)malonate - **6a**:*

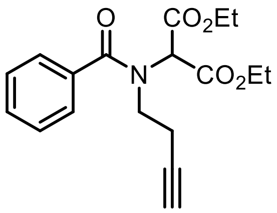

Using the general procedure **B**, with benzoic acid (258 mg - 2.11 mmol) and diethyl 2-(but-3-yn-1-ylamino)malonate (400 mg - 1.76 mmol). The corresponding diethyl 2-(*N*-(but-3-yn-1-yl)benzamido)malonate (264 mg - Yield 45%) was obtained as a clear oil (Mixture of two rotamers (55:45 in $CDCl_3$; 74:26 in CD_3OD), after purification by column chromatography (80:20 to 70:30 Pentane:EtOAc). (Only the major isomer is assigned) ^{1}H NMR (300 MHz, $CDCl_3$): δ = 7.51 – 7.33 (bs, 5H, H_{arom}), 5.41 – 5.02 (bs, 1H, C<u>H</u>-CO_2Et), 4.42 – 4.14 (bs, 4H, 2xC<u>H</u>$_2$-CH_3), 3.79 – 3.47 (bs, 2H, N-C<u>H</u>$_2$-), 2.82 – 2.25 (bs, 2H, -C<u>H</u>$_2$-C≡), 2.01 – 1.90 (bs, 1H, -C≡C<u>H</u>), 1.43 – 1.19 (bs, 6H, 2xC<u>H</u>$_3$-CH_2). NMR signals are all broad and not well defined in $CDCl_3$. Characterization was done in CD_3OD in an attempt to get a better spectrum (Only the major isomer is assigned). ^{1}H NMR (500 MHz, CD_3OD): δ = 7.61 – 7.32 (m, 5H, H_{arom}), 4.36 – 4.29 (q, *J* = 7.1 Hz, 4H, 2xC<u>H</u>$_2$-CH_3), 3.53 (bs, 2H, N-C<u>H</u>$_2$-), 2.44 (bs, 2H, -C<u>H</u>$_2$-C≡), 2.33 (bs, 1H, -C≡C<u>H</u>), 1.33 (t, *J* = 6.8 Hz, 6H, 2xC<u>H</u>$_3$-CH_2), C<u>H</u>-(CO_2Et) not observed. ^{13}C NMR (126 MHz, CD_3OD): δ = 174.4 (Ph-<u>C</u>=O), 167.0 (-O-<u>C</u>=O), 136.3 (C_{arom}), 131.3 ($CH_{arom\ para}$), 129.9 (2xCH_{arom}), 127.6 (2xCH_{arom}), 81.2 (-<u>C</u>≡CH), 72.1 (-C≡<u>C</u>H), 63.8 (N-<u>C</u>H-$(CO_2Et)_2$), 63.2 (<u>C</u>H_2-CH_3), 50.7 (-<u>C</u>H_2-N), 19.7 (CH_2-<u>C</u>H_2-C), 14.4 (CH_2-<u>C</u>H_3) ; CH Not ob. HRMS (DCI-CH_4) m/z (%): Calculated: 332.1498 ($C_{18}H_{22}NO_5$); Found: 332.1503

4-methylbenzenesulfonyl azide - CAS [941-55-9]:

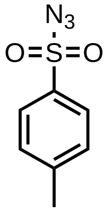

A solution of *p*-toluenesulfonyl chloride (3.85 g - 20 mmol - 1.0 eq) in acetone (20 mL) was added dropwise to a solution of NaN_3 (2 g - 30 mmol - 1.5 eq) in water (10 mL) at 5°C. The reaction was then allowed to warm at room temperature and stirred overnight. The volatiles were then removed, the aqueous layer was extracted 3 times with EtOAc. The organic combined layers were dried using Na_2SO_4 and the solvent was removed *in vacuo* to yield Tosyl azide (3.7 g - 94% Yield) used in the next step without further purification. ^{1}H NMR (300 MHz, $CDCl_3$): δ = 7.85 (d, *J* = 8.7 Hz, 2H, H_{arom}), 7.41 (d, *J* = 8.7 Hz, 2H, H_{arom}), 2.4 (s, 3H, C<u>H</u>$_3$). Spectroscopic data in accordance with literature.[13]

Dimethyl 2-diazomalonate - CAS [6773-29-1]:

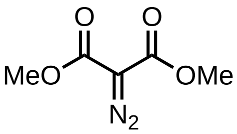

DBU (1.7 mL - 11.35 mmol - 1.5 eq) was added dropwise to a stirred solution of malonate (1g - 7.57 mmol - 1.0 eq) and Tosylazide (1.38 mL - 9.08 mmol - 1.2 eq) in acetonitrile (20 mL) at 5°C. Upon addition of DBU the reaction turns brown. It was then allowed to warm at room temperature and stirred overnight. The volatiles were removed, and the residue dissolved in DCM (20 mL). The organic layer was then washed 3 times with H_2O, dried over Na_2SO_4. The DCM was removed *in vacuo*. Pure Dimethyl 2-diazomalonate (1 g - 84% Yield) was obtained after purification by column chromatography (80:20 to 60:40 Pentane:Et_2O). ^{1}H NMR (300 MHz, $CDCl_3$): δ = 3.84 (s, 6H, 2xC<u>H</u>$_3$). Spectroscopic data in accordance with literature.[14]

*Dimethyl 2-(but-3-yn-1-yloxy)malonate - **7a** - CAS [214914-77-9]:*

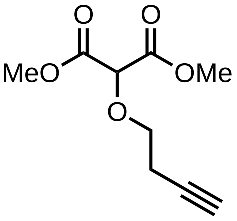

Rhodium acetate dimer (4.5 mg – 0.01 mmol – 0.5 mol%) was added to a solution of dimethyl 2-diazomalonate (332 mg – 2.1 mmol – 1.05 eq) and but-3-yn-1-ol (140 mg – 2.0 mmol – 1.0 eq) in benzene (7 mL). The resulting solution was heated at 60°C for 2 h. The volatiles were then removed and the residue was purified by column chromatography (80:20 Pentane:EtOAc) to yield pure dimethyl 2-(but-3-yn-1-yloxy)malonate (125 mg – 31% yield) as a clear oil. ^{1}H NMR (300 MHz, $CDCl_3$): δ = 4.61 (s, 1H, $(CO_2Me)_2$-C<u>H</u>-O-), 3.82 (s, 6H, 2x(C<u>H</u>$_3$-O)), 3.75 (t, *J* = 7.2 Hz, 2H, O-C<u>H</u>$_2$-CH_2-), 2.58 (td, *J* = 7.2, 2.7 Hz, 2H, -C<u>H</u>$_2$-C≡CH), 1.99 (t, *J* = 2.7 Hz, 1H, -C≡C<u>H</u>). Spectroscopic data in accordance with literature.[15]

Methyl 2-acetyloct-7-ynoate - ***8a*** - *CAS [134149-22-7]:*

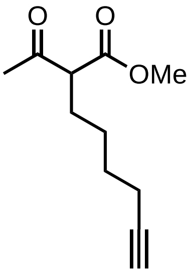

Using general procedure **A**, with methyl 3-oxobutanoate (1.16 g - 10 mmol) and 6-chlorohex-1-yne (979 mg - 8.4 mmol) pure methyl 2-acetyloct-7-ynoate (511 mg - 31% Yield) was obtained as a colorless oil via column chromatography (90:10 to 60:40 Pentane:Et_2O). Mixture of keto-enol tautomers. ^1H NMR (300 MHz, $CDCl_3$): δ = 3.74 (s, 3H, CH_3-O), 3.43 (t, J = 7.4 Hz, 1H, COMe-CH-CO_2Me), 2.23 (s, 3H, CH_3-C), 2.19 (td, *J* = 6.8, 2.5 Hz, 2H, -CH_2-C≡CH), 1.93 (t, *J* = 2.5 Hz, 1H, -C≡CH), 1.91 – 1.81 (m, 2H, CH-CH_2-CH_2-), 1.55 (m, 2H, CH_2-CH_2-C≡CH), 1.45 – 1.31 (m, 2H, CH-CH_2-CH_2-CH_2). Spectroscopic data in accordance with literature.[8]

Dimethyl 2-((2-ethynylbenzyl)oxy)malonate - ***9a***:

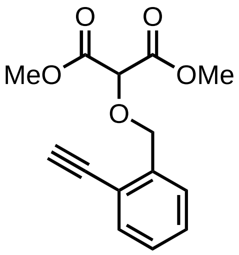

Rhodium acetate dimer (4.5 mg - 0.01 mmol - 0.5 mol%) was added to a solution of dimethyl 2-diazomalonate (350 mg - 2.2 mmol - 1.0 eq) and 2-ethynylbenzyl alcohol (307 mg - 2.3 mmol - 1.05 eq), in benzene (6 mL). The resulting solution was then heated at 60°C for 2 h. The volatiles were removed and the residue was purified by column chromatography (80:20 Pentane:EtOAc) to yield pure dimethyl 2-((2-ethynylbenzyl)oxy)malonate as an orange oil (156 mg - 27% Yield). ^1H NMR (300 MHz, $CDCl_3$): δ = 7.58 – 7.53 (m, 1H, H_{arom}), 7.49 (pseudo-dd, *J* = 7.6, 1.4 Hz, 1H, H_{arom}), 7.37 (pseudo-td, *J* = 7.6, 1.5 Hz, 1H, H_{arom}), 7.30 – 7.21 (m, 1H, H_{arom}), 4.89 (s, 2H, CH_2), 4.64 (s, 1H, -CO_2Me-CH-O-), 3.80 (s, 6H, CH_3-O), 3.29 (s, 1H, -C≡CH). ^{13}C NMR (75 MHz, $CDCl_3$): δ = 166.9 (-O-C=O), 138.7 (CH_2-C_{arom}), 132.8 (CH_{arom}), 129.3 (CH_{arom}), 128.3 (CH_{arom}), 128.0 (CH_{arom}), 121.1 (≡C-C_{arom}), 82.2 (-C≡CH), 81.1 (-C≡CH), 78.3 (O-CH-($CO_2Me)_2$), 70.9 (-CH_2-), 53.0 (-CH_3). HRMS (DCI-CH_4) m/z: Calculated: 263.0919 ($C_{14}H_{15}O_5$); Found: 263.0921.

Diethyl 2-(N-benzylpent-4-ynamido)malonate - ***10a*** - *CAS [1351364-67-4]:*

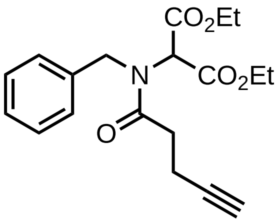

Using the general procedure **A**, with pent-4-ynoic acid (208 mg - 2.14 mmol) and diethyl 2-(benzylamino)malonate (469 mg - 1.77 mmol). The corresponding diethyl 2-(*N*-benzylpent-4-ynamido)malonate (486 mg - 80% Yield) was obtained as a clear oil after purification by column chromatography (50:50 Pentane:Et_2O). Mixture of 2 rotamers at room temperature (91%:9%), only the major rotamer is assigned. ^1H NMR (300 MHz, $CDCl_3$): δ = 7.39 – 7.18 (m, 5H, H_{arom}), 5.54 (s, 1H, CH-CO_2Et), 4.74 (s, 2H, Ph- CH_2-N), 4.22 – 3.88 (m, 4H, 2xCH_2-CH_3), 2.71 – 2.47 (m, 4H, -CH_2-CH_2;-CH_2-CH_2), 1.94 (t, *J* = 2.5 Hz, 1H, -C≡CH), 1.19 (t, *J* = 7.1 Hz, 6H, 2x(CH_3-CH_2-)). Spectroscopic data in accordance with literature.[11]

Diethyl 2-(pent-4-yn-1-ylamino)malonate:

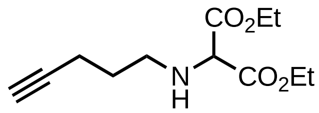

To a solution of pent-4-yn-1-amine (270 mg, 3.25 mmol, 1.0 eq), diethyl bromomalonate (555 µL, 3.25 mmol, 1.0 eq) in 6 mL of $CHCl_3$ was added triethylamine (520 µL, 3.9 mmol, 1.2 eq). The solution was heated to 75°C for 16 h. The resulting black mixture was then poured into a separatory funnel containing 15 mL of 2M NaOH, and the aqueous layer was extracted 3 times with 15 mL of EtOAc. The organic layers were combined, dried over Na_2SO_4 and the volatiles were removed *in vacuo*. Diethyl 2-(pent-4-yn-1-ylamino)malonate (329 mg - 42% Yield) was then obtained as a black oil after column chromatography (80:20; Pentane:EtOAc; 1% of NEt_3). ^1H NMR (300 MHz, $CDCl_3$) δ = 4.24 (qd, *J* = 7.2, 0.8 Hz, 4H, CH_2-CH_3), 4.05 (s, 1H, CO_2Et-CH-CO_2Et), 2.73 (t, *J* = 7.0 Hz, 2H, CH_2-CH_2-NH), 2.30 (td, *J* = 7.0, 2.7 Hz, 2H, ≡C-CH_2-CH_2), 2.08 (s, 1H, NH), 1.96 (t, *J* = 2.7 Hz, 1H, C≡CH), 1.74 (pseudo-quint, *J* = 7.0 Hz, 2H, CH_2-CH_2-CH_2), 1.31 (t, *J* = 7.2 Hz, 6H, CH_2-CH_3). ^{13}C NMR (75 MHz, $CDCl_3$) δ = 168.7 (C=O), 83.9 (≡CH), 68.8 (-C≡), 65.2 (NH-CH-(C=O)$_2$), 61.7 (CH_2-CH_3), 46.7 (-CH_2-NH), 28.8 (CH_2-CH_2-CH_2), 16.2 (CH_2-CH_2-C≡), 14.2 (CH_3). HRMS (DCI-CH_4) m/z (%): Calculated: 242.1392 ($C_{12}H_{20}NO_4$); Found: 242.1387.

*Diethyl 2-(N-(pent-4-yn-1-yl)benzamido)malonate - **11a**:*

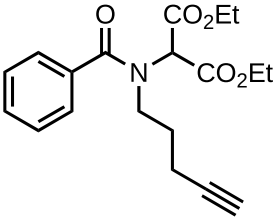

Using the general procedure **B**, with benzoic acid (146 mg - 1.2 mmol) and diethyl 2-(pent-4-yn-1-ylamino)malonate (241 mg - 1.0 mmol). The corresponding diethyl 2-(*N*-(pent-4-yn-1-yl)benzamido)malonate (206 mg - Yield 60%) was obtained as yellow crystals after purification by column chromatography (100:0 to 80:20 Pentane:EtOAc). ^{1}H NMR (300 MHz, CD_3OD, 25°C): δ = 7.57 – 7.27 (m, 5H, H_{arom}), 4.29 (q, *J* = 7.1 Hz, 4H, 2xCH_2-CH_3), 3.48 (t, *J* = 7.6 Hz, 2H, CH_2-N), 2.33 - 1.99 (m, 3H, C≡CH; CH_2-C≡), 1.82 - 1.65 (m, 2H, -CH_2-CH_2-CH_2-), 1.33 (t, *J* = 7.1 Hz, 6H, 2xCH_3-CH_2), CH-(CO_2Et) not observed. It should be stressed that at room temperature NMR Signals are broad and not well resolved. Data acquired at -60°C gave a better signal. ^{1}H NMR (400 MHz, CD_3OD, -60°C): δ = 7.60 – 7.33 (m, 5H, H_{arom}), 4.27 (q, *J* = 7.1 Hz, 4H, 2xCH_2-CH_3), 3.47 (t_{app}, *J* = 7.3 Hz, 2H, CH_2-N), 2.25 (bs, 1H, C≡CH), 2.10 (td, *J* = 7.0, 2.6 Hz, 2H, -CH_2-C≡), 1.82 – 1.73 (m, 2H, -CH_2-CH_2-CH_2-), 1.33 (t, *J* = 7.1 Hz, 6H, 2xCH_3-CH_2), CH-(CO_2Et) not observed. ^{13}C NMR (75 MHz, $CDCl_3$, 25°C): 167.1 (-O-C=O), 136.5 (C_{arom}), 131.2 ($CH_{arom\ para}$), 129.8 (2xCH_{arom}), 127.6 (2xCH_{arom}), 83.3 (-C≡CH), 70.4 (C≡CH), 63.2 (2xCH_2-CH_3), 50.9 (CH_2-N), 28.7 (CH_2-CH_2-CH_2-), 16.1 (-CH_2-C≡), 14.4 (2xCH_3). MS (HR-ESI) m/z (%): Calculated: 346.1654 ($C_{19}H_{24}NO_5$); Found: 346.1660.

*Diethyl 2-(N-benzylhex-5-ynamido)malonate - **12a**:*

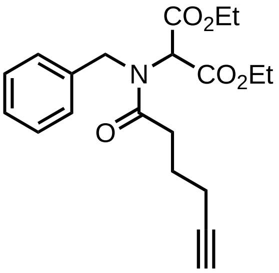

Using the general procedure **A**, with 5-hexynoic acid (507 mg - 4.52 mmol) and diethyl 2-(benzylamino)malonate (1 g - 3.77 mmol). The corresponding diethyl 2-(*N*-benzylhex-5-ynamido)malonate (1.23 g -Yield 91%) was obtained as a clear oil after purification by column chromatography (60:40 Pentane:Et_2O). Mixture of 2 rotamers at room temperature (94%:06%), only the major rotamer is assigned. ^{1}H NMR (300 MHz, $CDCl_3$): δ = 7.39 – 7.20 (m, 5H, H_{arom}), 5.45 (s, 1H, CH-CO_2Et), 4.74 (s, 2H, C-CH_2-N), 4.23 – 3.94 (m, 4H, CH_2-CH_3), 2.53 (t, *J* = 7.1 Hz, 2H, CH_2-C=O), 2.25 (td, *J* = 6.8, 2.7 Hz, 2H, CH_2-C≡C), 1.98 – 1.78 (m, 3H, C≡CH, CH_2-CH_2-CH_2), 1.20 (t, *J* = 7.1 Hz, 6H, CH_3-CH_2). ^{13}C NMR (75 MHz, $CDCl_3$): δ = 173.9 (N-C=O), 166.3 (CO_2Et), 136.5 (C_{arom}), 128.7 (CH_{arom}), 127.6 ($CH_{arom\ para}$), 126.5 (CH_{arom}), 83.6 (-C≡CH), 69.2 (-C≡CH), 62.1 (2xCH_2-CH_3), 61.2 (CH-$(CO_2Et)_2$), 50.9 (N-CH_2-Ph), 31.6 (O=C-CH_2-), 23.8 (-CH_2-CH_2-CH_2-), 17.7 (-CH_2-C≡), 13.9 (-CH_3). HRMS (ESI) m/z (%): Calculated: 360.1811 ($C_{20}H_{26}NO_5$); Found: 360.1805

*Methyl 2-acetyloct-5-ynoate - CAS [811784-45-9] - **13a**:*

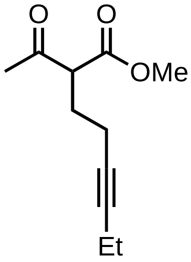

Using general procedure **A**, with methyl 3-oxobutanoate (392 mg - 3.38 mmol) and 1-bromohex-3-yne (500 mg - 2.8 mmol) pure methyl 2-acetyloct-5-ynoate (112 mg - 20% Yield) was obtained as a clear oil via column chromatography (95:05 Pentane:EtOAc). Mixture of keto-enol tautomers. ^{1}H NMR (300 MHz, $CDCl_3$): δ = 3.74 (s, 3H, CH_3-O), 3.72 (t, *J* = 7.2 Hz, 1H, COMe-CH-CO_2Me), 2.27 (s, 3H, CH_3-C), 2.24 – 1.99 (m, 6H, 3x-CH_2-), 1.11 (t, *J* = 7.5 Hz, 3H, CH_2-CH_3).

*Diethyl 2-(N-benzylbut-2-ynamido)malonate - **14a**:*

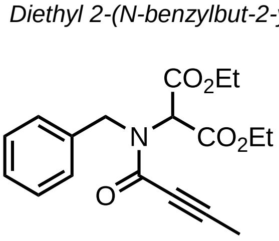

Using the general procedure **A**, with but-2-ynoic acid (250 mg - 2.97 mmol) and diethyl 2-(benzylamino)malonate (656 mg - 2.47 mmol). The corresponding diethyl 2-(*N*-benzylbut-2-ynamido)malonate (800 mg - 97% Yield) was obtained as a white solid after purification by column chromatography (using a gradient from 80:20 to 60:40 of Pentane:EtOAc). Mixture of 2 rotamers at room temperature (77%:23%), only the major rotamer is assigned. mp = 50 °C**.** ^{1}H NMR (300 MHz, $CDCl_3$): δ = 7.37-7.17 (m, 5H, H_{arom}), 5.30 (s, 1H, CH-CO_2Et), 4.97 (S, 2H, Ph-CH_2-N), 4.13-4.02 (m, 2H, CH_2-CH_3), 3.97-3.84 (m, 2H, CH_2-CH_3), 1.97 (s, 3H, CH_3-C≡C), 1.15 (t, 3H, 2xCH_3-CH_2). ^{13}C NMR (75 MHz, $CDCl_3$): δ = 165.6 (CO_2Et), 155.8 (N-C=O), 136.2 (C_{arom}), 128.5 (CH_{arom}), 127.8 (CH_{arom}), 127.7 (CH_{arom}), 91.3 (-C≡C-CH_3), 72.9 (O=C-C≡C-), 62.2 (-CH_2-CH_3), 59.8 (-CH-(CO_2Et), 52.3 (-CH_2-Ph), 13.9 (-CH_2-CH_3), 4.3 (≡C-CH_3). HRMS (DCI-CH_4) m/z (%): Calculated: 332.1498 ($C_{18}H_{22}NO_5$); Found: 332.1501.

*Diethyl 2-(N-benzylpent-3-ynamido)malonate - **15a** - CAS [1351364-62-9]:*

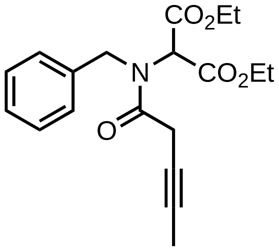

Using the general procedure **B**, with pent-3-ynoic acid (250 mg - 2.55 mmol) and diethyl 2-(benzylamino)malonate (563 mg - 2.12 mmol). The corresponding diethyl 2-(*N*-benzylpent-3-ynamido)malonate (423 mg - 53% Yield) was obtained as a clear oil after purification by column chromatography (75:25 Pentane:EtOAc). Mixture of 2 rotamers at room temperature (87%:23%), only the major rotamer is assigned. ^{1}H NMR (300 MHz, $CDCl_3$): δ = 7.40 – 7.17 (m, 5H, H_{arom}), 5.38 (s, 1H, CH-CO_2Et), 4.79 (s, 2H, Ph-CH_2-N), 4.22 – 3.97 (m, 4H, 2xCH_2-CH_3), 3.27 (q, *J* = 2.6 Hz, 2H, -CH_2-C≡C), 1.77 (t, *J* = 2.6 Hz, 3H, -C≡C-CH_3), 1.21 (t, *J* = 7.1 Hz, 6H, 2xCH_2-CH_3). Spectroscopic data in accordance with literature.[11]

*Diethyl 2-(N-benzylhex-3-ynamido)malonate - **16a** - CAS [1351364-36-0]:*

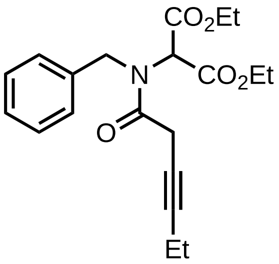

Using the general procedure **B**, with hex-3-ynoic acid (284 mg - 2.53 mmol) and diethyl 2-(benzylamino)malonate (560 mg - 2.11 mmol). The corresponding diethyl 2-(*N*-benzylhex-3-ynamido)malonate (279 mg - 38% Yield) was obtained as a clear oil (mixture of two rotamers 85%:15%) after purification by column chromatography (75:25 Pentane:EtOAc). Mixture of 2 rotamers at room temperature (85%:15%), only the major rotamer is assigned. ^{1}H NMR (300 MHz, $CDCl_3$): δ = 7.39 – 7.17 (m, 5H, H_{arom}), 5.37 (s, 1H, CH-CO_2Et), 4.80 (s, 2H, Ph-CH_2-N), 4.22 – 3.98 (m, 4H, 2xCH_2-CH_3), 3.29 (t, *J* = 2.4 Hz, 2H, -CH_2-C≡C), 2.23 – 2.06 (m, 2H, -C≡C-CH_2), 1.21 (t, *J* = 7.2 Hz, 6H, 2x(CH_3-CH_2-CO_2-), 1.08 (t, *J* = 7.5 Hz, 3H, C-CH_2-CH_3). Spectroscopic data in accordance with literature.[11]

Tert-butyl 4-cyclohexylbut-3-ynoate :

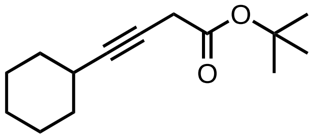

Using the procedure described by Fu *et al.* *t*Bu-diazoacetate (640 mg - 4.5 mmol) was added dropwise to a solution of ethynylcyclohexane (458 mg - 4.5 mmol), CuI (43 mg - 5% mol) in acetonitirile (7 mL).[16] The corresponding tert-butyl 4-cyclohexylbut-3-ynoate (902 mg - 90% yield) was obtained as a clear oil after filtration through a short silica plug (80:20 Pentane:DCM). ^{1}H NMR (300 MHz, $CDCl_3$): δ = 3.17 (d, *J* = 2.2 Hz, 2H, C-CH_2-CO), 2.45 – 2.27 (m, 1H, CH_2-CH-CH_2), 1.85 – 1.59 (m, 4H, 2x(CH_2)-CH-), 1.44 (s, 9H, 3xCH_3), 1.54 – 1.19 (m, 6H, 3x(-CH_2-). ^{13}C NMR (75 MHz, $CDCl_3$): δ = 168.3 (CO_2tBu), 87.8 (Cy-C≡C-), 81.5 (Cy-C≡C-CH_2-), 72.1 (-C-$(CH_3)_3$), 32.8 (2xCH_2), 29.2 (-CH-), 28.0 (3xCH_3-), 27.4 (2xCH_2), 26.0 (-CH_2-), 24.9 (-CH_2-). HRMS (DCI-CH_4) m/z (%): Calculated: 221.1542 ($C_{14}H_{21}O_2$); Found: 221.1534

*Diethyl 2-(N-benzyl-4-cyclohexylbut-3-ynamido)malonate - **17a**:*

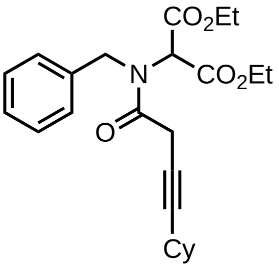

Using the general procedure **C**, with *tert*-butyl 4-cyclohexylbut-3-ynoate (372 mg - 2.04 mmol) and diethyl 2-(benzylamino)malonate hydrochloride (491 mg - 1.62 mmol - 0.75 eq) the corresponding diethyl 2-(*N*-benzyl-5,5-dimethylhex-3-ynamido)malonate (141 mg - 18% Yield) was obtained as a pale yellow oil after HPLC purification. Mixture of 2 rotamers at room temperature (87%:13%), only the major rotamer is assigned. ^{1}H NMR (300 MHz, $CDCl_3$): δ = 7.42 – 7.11 (m, 5H, H_{arom}), 5.35 (s, 1H, CH-CO_2Et), 4.82 (s, 2H, Ph-CH_2-N), 4.28 – 3.92 (m, 4H, 2xCH_2-CH_3), 3.30 (d, *J* = 2.2 Hz, 2H, -CH_2-C≡C), 2.32 (br, 1H, C≡C-CH-), 1.98 – 1.23 (m, 10H, 5xCH_2-Cy), 1.20 (t, *J* = 7.1 Hz, 6H, 2x(CH_3-CH_2-)). ^{13}C NMR (75 MHz, $CDCl_3$): δ = 169.5 (C=O), 166.1 (O-C=O), 136.2 (C^{IV}_{arom}), 128.8 (2xCH_{arom} - meta), 127.8 (CH_{arom} - para), 126.8 (2xCH_{arom} - ortho), 89.2 (Cy-C≡C; Not visible in ^{13}C-zgpg but in HMBC), 70.2 (Cy-C≡C-), 62.2 (CH_2-CH_3), 61.4 (O=C-CH-C=O), 51.5 (Ph-CH_2-), 32.7 (C_{Cy}), 29.8 (C_{Cy}), 29.3 (CH-Cy), 26.8 (C_{Cy}), 26.0 (C_{Cy}), 25.0 (C_{Cy}), 14.0 (CH_3-CH_2). HRMS (DCI-CH_4) m/z (%): Calculated: 414.2280 ($C_{24}H_{32}NO_5$); Found: 414.2300

Tert-butyl 4-phenylbut-3-ynoate - CAS [945488-48-2]:

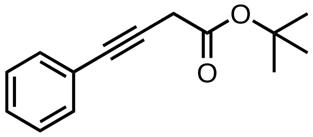

Using the procedure described by Fu *et al.* *tert*-Butyl diazoacetate (355 mg - 2.5 mmol) was added dropwise to a solution of phenylacetylene (255 mg - 2.5 mmol), CuI (24 mg - 5 mol%) in acetonitrile (3 mL).[16] The resulting solution was stirred at room temperature for 16h. The volatiles were removed *in vacuo* and the residue was filtered through a silica pad with Et_2O as eluent. The amount of allenic ester was then quantified using ^{1}H NMR spectroscopy with an internal standard. $PyHBr_3$ (1.0 equivalent - towards the amount of allenic ester) was added in $CHCl_3$ to the residue and the reaction was stirred for 20h. The volatiles were removed *in vacuo* and the residue was purified by column chromatography (using a gradient from 100:0 to 80:20 Heptane:DCM) to yield the corresponding tert-butyl 4-phenylbut-3-ynoate (157 mg - 28% Yield) as a pale-yellow oil. ^{1}H NMR (300 MHz, $CDCl_3$): δ = 7.48 – 7.27 (m, 5H, H_{arom}), 3.42 (s, 2H, -CH_2-), 1.50 (s, 9H, 3xCH_3). Spectroscopic data in accordance with literature.[17]

*Diethyl 2-(N-benzyl-4-phenylbut-3-ynamido)malonate - **18a** - CAS [1351364-66-3]:*

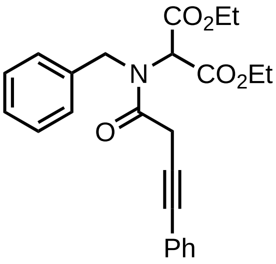

Using the general procedure **C**, with tert-butyl 4-phenylbut-3-ynoate (100 mg - 0.46 mmol) and diethyl 2-(benzylamino)malonate hydrochloride (139 mg - 0.46 mmol) the corresponding diethyl 2-(*N*-benzyl-4-phenylbut-3-ynamido)malonate (132 mg - 70% Yield) was obtained as a yellow oil after HPLC purification. Mixture of 2 rotamers at room temperature (86%:14%), only the major rotamer is assigned. ^{1}H NMR (300 MHz, $CDCl_3$): δ = 7.39 – 7.21 (m, 10H, H_{arom}), 5.39 (s, 1H, CH-CO_2Et), 4.88 (s, 2H, Ph-CH_2-N), 4.25 – 4.00 (m, 4H, 2xCH_2-CH_3), 3.56 (s, 2H, -CH_2-C≡C), 1.21 (t, *J* = 7.2 Hz, 6H, 2x(CH_3-CH_2-)). Spectroscopic data in accordance with literature.[11]

Tert-butyl 5,5-dimethylhex-3-ynoate:

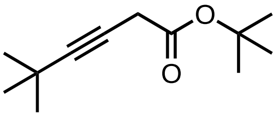

Using the procedure described by Fu *et al.* *tert*-Butyl diazoacetate (854 mg - 6 mmol) was added dropwise to a solution of 3,3-dimethylbut-1-yne (1.14 g - 6 mmol), CuI (56 mg - 5% mol) in acetonitrile (8 mL).[16] The corresponding tert-butyl 5,5-dimethylhex-3-ynoate (888 mg - 81% Yield) was obtained as a pale-yellow oil after purification via column chromatography (80:20 Pentane:DCM). ^{1}H NMR (300 MHz, $CDCl_3$): δ = 3.14 (s, 2H, CH_2), 1.46 (s, 9H, *t*Bu-O), 1.22 (s, 9H, *t*Bu-C≡). ^{13}C NMR (75 MHz, $CDCl_3$): δ = 168.2 (O-C=O), 91.9 (*t*Bu-C≡), 81.4 (C≡C-CH_2), 70.7 (O-C-(CH_3)$_3$), 31.1 ((CH_3)$_3$-C-C≡), 28.0 ((CH_3)$_3$-C-O), 27.4 ((CH_3)$_3$-C-C≡), 27.3 (C-CH_2-C). HRMS (DCI-CH_4) m/z (%): Calculated: 197.1542 ($C_{12}H_{21}O_2$); Found: 197.1533.

*Diethyl 2-(N-benzyl-5,5-dimethylhex-3-ynamido)malonate - **19a**:*

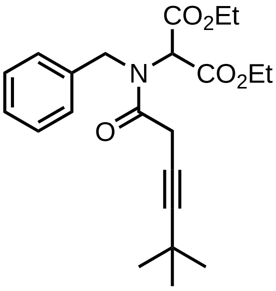

Using the general procedure **C** and heptane to remove the $SOCl_2$ by azeotropic distillation, with tert-butyl 5,5-dimethylhex-3-ynoate (372 mg - 2.04 mmol) and diethyl 2-(benzylamino)malonate hydrochloride (491 mg - 1.62 mmol - 0.75 eq) the corresponding diethyl 2-(*N*-benzyl-5,5-dimethylhex-3-ynamido)malonate (141 mg - 18% Yield) was obtained as a slightly yellow oil after HPLC purification. Mixture of 2 rotamers at room temperature (86%:14%), only the major rotamer is assigned. ^{1}H NMR (300 MHz, $CDCl_3$): δ = 7.40 – 7.15 (m, 5H, H_{arom}), 5.32 (s, 1H, CH-CO_2Et), 4.82 (s, 2H, Ph-CH_2-N), 4.22 – 3.87 (m, 4H, 2xCH_2-CH_3), 3.29 (s, 2H, -CH_2-C≡C), 1.21 (t, *J* = 7.2 Hz, 6H, 2x(CH_3-CH_2-)), 1.17 (s, 9H, *t*Bu). ^{13}C NMR (75 MHz, $CDCl_3$): δ = 169.5 (C=O), 166.1 (O-C=O), 136.2 (C_{arom}), 128.9 (2xCH_{arom} - meta), 127.9 (CH_{arom} - para), 126.9 (2xCH_{arom} - ortho), 93.0 (tBu-C≡C; Not visible in ^{13}C-zgpg but in HMBC), 70.5 (CH_2-C≡C; Not visible in ^{13}C-zgpg but in HMBC) 62.2 (CH_2-CH_3), 61.4 (O=C-CH-C=O), 51.5 (N-CH_2-Ph), 31.0 ((CH_3)$_3$-C), 27.6 ((CH_3)$_3$-C-C), 26.8 (C-CH_2-C=O), 14.0 (CH_2-CH_3). HRMS (DCI-CH_4) m/z (%): Calculated: 388.2124 ($C_{22}H_{30}NO_5$); Found: 388.2143

Cyclized products

General procedure for the Conia-ene cyclisation

The substrate, $Mg(OTf)_2$, HFIP (100 µL), the internal standard and complex were added in $CDCl_3$ (400 µL) in an NMR tube and the resulting solution was heated at the indicated temperature for the indicated time.

*1,1'-(2-methylenecyclopentane-1,1-diyl)bis(ethan-1-one) - **1b** - CAS [88459-74-9]:*

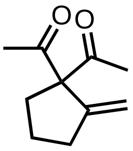

Using the general procedure, with 3-(pent-4-yn-1-yl)pentane-2,4-dione (0.25 mmol - [0.5M]), Pd2(SCS)2 (1 mol% Pd), $Mg(OTf)_2$ (5 mol%) and 1,3,5-trimethoxybenzene as internal standard, 1,1'-(2-methylenecyclopentane-1,1-diyl)bis(ethan-1-one) was formed with >95% yield after 2 h at 25°C as estimated by 1H NMR analysis. Isolated by filtering on a silica pad with DCM as eluant (35 mg - 85% Yield). ^{1}H NMR (300 MHz, Chloroform-d): δ = 5.34 (t, *J* = 2.2 Hz, 1H, H_{vinyl}), 5.14 (t, *J* = 2.2 Hz, 1H, H_{vinyl}), 2.45 (tt, *J* = 7.3, 2.2 Hz, 2H, CH_2-CH_2-C=), 2.28 (t, *J* = 6.9 Hz, 2H, $(MeOC)_2$-C-CH_2-CH_2-), 2.20 (s, 6H, 2xCH_3), 1.73 (quint, *J* = 7.3 Hz, 2H, CH_2-CH_2-CH_2). Spectroscopic data in accordance with literature.[18]

*Methyl 1-acetyl-2-methylenecyclopentane-1-carboxylate - **2b** - CAS [146445-28-5]:*

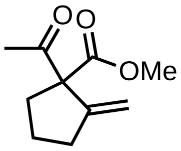

Using the general procedure, with methyl 2-acetylhept-6-ynoate (0.25 mmol - [0.5M]), $Pd_2(SCS)_2$ (1 mol% Pd), $Mg(OTf)_2$ (5 mol%) and 1,3,5-trimethoxybenzene as internal standard methyl 1-acetyl-2-methylenecyclopentane-1-carboxylate was formed with >95% yield after 1 h 30 min at 25°C as estimated by ^{1}H NMR analysis. Isolated by filtering on a silica pad with DCM as eluant (41 mg - 90% Yield). ^{1}H NMR (300 MHz, $CDCl_3$): δ = 5.30 (t, *J* = 2.2 Hz, 1H, H_{vinyl}), 5.23 (t, *J* = 2.3 Hz, 1H, H_{vinyl}), 3.75 (s, 3H, CH_3-O), 2.51 – 2.33 (m, 3H, CH_2-CH_a-C(CO_2Me); -CH_2-CH_2-C_{vinyl}), 2.23-2.14 (m, 1H, CH_2-CH_b-C), 2.22 (s, 3H, CH_3-C), 1.85 – 1.59 (m, 2H, -CH_2-CH_2-CH_2-). Spectroscopic data in accordance with literature.[19]

*Dimethyl 2-methylenecyclopentane-1,1-dicarboxylate - **3b** - CAS [134149-18-1]:*

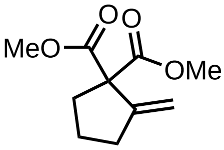

Using the general procedure, with methyl 2-acetylhept-6-ynoate (0.25 mmol - [0.5M]), $Pd_2(SCS)_2$ (1 mol% Pd), $Mg(OTf)_2$ (5 mol%) and 1,2,4,5-tetramethylbenzene as internal standard Dimethyl 2-methylenecyclopentane-1,1-dicarboxylate was formed with >95% yield after 1 h 30 min at 25°C as estimated by ^{1}H NMR analysis. Isolated by filtering on a silica pad with DCM as eluant (45 mg - 91% Yield). ^{1}H NMR (300 MHz, $CDCl_3$): δ = 5.30 (t, *J* = 2.3 Hz, 1H, H_{vinyl}), 5.27 (t, *J* = 2.1 Hz, 1H, H_{vinyl}), 3.74 (s, 6H, 2xCH_3-O), 2.46 (tt, *J* = 7.3, 2.2 Hz, 2H, -CH_2-C_{vinyl}), 2.35 (t, *J* = 6.9 Hz, 2H, -CH_2-CH_2-C($CO_2Me)_2$), 1.74 (quint, *J* = 7.1 Hz, 2H, -CH_2-CH_2-CH_2-). Spectroscopic data in accordance with literature.[20]

*2-methylenecyclopentane-1,1-dicarbonitrile - **4b** - CAS [201146-05-6]:*

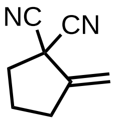

Using the general procedure, with 2-(pent-4-yn-1-yl)malononitrile (0.25 mmol - [0.5M]), $Pd_2(SCS)_2$ (1 mol% Pd), without $Mg(OTf)_2$ and anisole as internal standard 2-methylenecyclopentane-1,1-dicarbonitrile was formed with >95% yield after 16 h at 25°C as estimated by ^{1}H NMR analysis. Isolated by filtering on a silica pad with DCM as eluant (27.5 mg - 83% Yield). ^{1}H NMR (300 MHz, $CDCl_3$): δ = 5.68 (pseudo-q, *J* = 2.1Hz, 1H, H_{vinyl}), 5.45 (q, *J* = 2.1 Hz, 1H, H_{vinyl}), 2.61 (tt, *J* = 7.5, 2.4 Hz, 2H, -CH_2-C_{vinyl}), 2.48 (t, *J* = 6.9 Hz, 2H, -CH_2-C$(CN)_2$)), 2.06 (quint, *J* = 7.1 Hz, 2H, -CH_2-CH_2-CH_2-). Spectroscopic data in accordance with literature.[21]

*Diethyl 1-benzyl-3-methylene-5-oxopyrrolidine-2,2-dicarboxylate - **5b** - CAS [1383445-13-3]:*

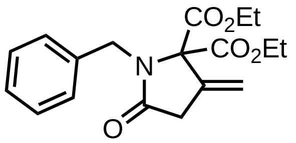

Using the general procedure, with diethyl 2-(*N*-benzylbut-3-ynamido)malonate (0.25 mmol - [0.5M]), $Pd_2(SCS)_2$ (1 mol% Pd), $Mg(OTf)_2$ (5 mol%) and 1,2,4,5-tetramethylbenzene as internal standard diethyl 1-benzyl-3-methylene-5-oxopyrrolidine-2,2-dicarboxylate was formed with >95% yield after less than 5 minutes at 25°C as estimated by ^1H NMR analysis. Isolated by column chromatography (80:20 Pentane:EtOAc - 79 mg - 95% Yield). ^1H NMR (300 MHz, $CDCl_3$): δ = 7.31 – 7.13 (m, 5H, H_{arom}), 5.56 (td, *J* = 2.7, 0.8 Hz, 1H, H_{vinyl}), 5.41 (td, *J* = 2.4, 0.8 Hz, 1H, H_{vinyl}), 4.73 (s, 2H, Ph-CH_2-N) 4.03 – 3.80 (m, 4H, 2xCH_2-CH_3), 3.31 (t, *J* = 2.6 Hz, 2H, -C-CH_2-C), 1.10 (t, *J* = 7.1 Hz, 6H, CH_3-CH_2). Spectroscopic data in accordance with literature.[11]

*Diethyl 1-benzoyl-3-methylenepyrrolidine-2,2-dicarboxylate - **6b**:*

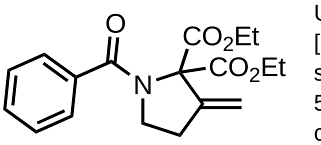

Using the general procedure with diethyl 2-(*N*-(but-3-yn-1-yl)benzamido)malonate (0.25 mmol - [0.5M]), $Pd_2(SCS)_2$ (1 mol% Pd), $Mg(OTf)_2$ (5 mol%) and (methoxymethyl)benzene as internal standard, diethyl 1-benzoyl-3-methylenepyrrolidine-2,2-dicarboxylate was formed with >95% yield after 50 minutes at 25°C as estimated by ^1H NMR analysis. Isolated as a white solid by column chromatography (80:20 Pentane:EtOAc - 70 mg - 85% Yield). ^1H NMR (300 MHz, $CDCl_3$) δ = 7.58 – 7.35 (m, 5H, H_{arom}), 5.62 (t, *J* = 2.1 Hz, 1H, C=CH_aH_b), 5.23 (t, *J* = 2.1 Hz, 1H, C=CH_aH_b), 4.43 – 4.09 (m, 4H, CH_2-CH_3), 3.71 (t, *J* = 7.2 Hz, 2H, CH_2-N), 2.76 (tt, *J* = 7.2, 2.1 Hz, 2H, CH_2-CH_2-C), 1.30 (t, *J* = 7.1 Hz, 6H, CH_2-CH_3). ^{13}C NMR (75 MHz, $CDCl_3$): δ = 166.4 (CO_2Et), 136.0 (C_{arom}), 130.3 (CH_{arom}), 128.5 2x(CH_{arom}), 127.0 2x(CH_{arom}), 111.8 (CH_2vinyl), 62.2 (2xCH_2-CH_3), 48.9 (N-CH_2-CH_2), 32.6 (CH_2-CH_2-C_{vinyl}), 14.1 (2xCH_3-CH_2). C=$O_{benzoyl}$ and C_{quat} vinyl not observed. HRMS (DCI-CH_4) m/z (%): Calculated: 332.1498 ($C_{18}H_{22}NO_5$); Found: 332.1502

*Dimethyl 3-methylenedihydrofuran-2,2(3H)-dicarboxylate - **7b** - CAS [1067676-30-5]:*

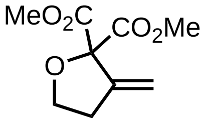

Using the general procedure, with dimethyl 2-(but-3-yn-1-yloxy)malonate (0.25 mmol - [0.5M]), $Pd_2(SCS)_2$ (1 mol% Pd), $Mg(OTf)_2$ (5 mol%) and 1,3,5-trimethoxybenzene as internal standard dimethyl 3-methylenedihydrofuran-2,2(3H)-dicarboxylate was formed with 90% yield after 4 h 15 min at 75°C as estimated by ^1H NMR analysis. Isolated by column chromatography (80:20 Pentane:EtOAc - 40.5 mg - 81% Yield). ^1H NMR (300 MHz, $CDCl_3$): δ = 5.53 (t, *J* = 2.3 Hz, 1H, H_{vinyl}), 5.40 (t, *J* = 2.2 Hz, 1H, H_{vinyl}), 4.13 (t, *J* = 7.0 Hz, 2H, -O-CH_2-CH_2-), 3.80 (s, 6H 2xCH_3-O), 2.72 (tt, *J* = 7.0, 2.2 Hz, 2H, CH_2-CH_2-C). Spectroscopic data in accordance with literature.[15]

*Methyl 1-acetyl-2-methylenecyclohexane-1-carboxylate - **8b** - CAS [134149-23-8]:*

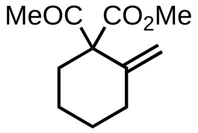

Using the general procedure with methyl 2-acetyloct-7-ynoate (0.25 mmol - [0.5M]), $Pd_2(SCS)_2$ (5 mol% Pd), $Mg(OTf)_2$ (20 mol%) and (methoxymethyl)benzene as internal standard methyl 1-acetyl-2-methylenecyclohexane-1-carboxylate was formed with 90% yield after 20 h at 75°C as estimated by ^1H NMR analysis. Isolated by column chromatography (90:10 Pentane:EtOAc - 41 mg - 84% Yield. ^1H NMR (300 MHz, $CDCl_3$): δ = 5.05 (s, 1H, H_{vinyl}), 4.62 (s, 1H, H_{vinyl}), 3.77 (s, 3H, CH_3-O), 2.42 – 2.30 (m, 1H, (CO_2Me)C-CH_a-CH_2-), 2.24 (s, 3H, CH_3-C), 2.29 – 2.00 (m, 3H, (CO_2Me)C-CH_b-CH_2-; -CH_2-), 1.72 – 1.16 (m, 4H, -CH_2-; -CH_2-). Spectroscopic data in accordance with literature.[20]

*Dimethyl 4-methyleneisochromane-3,3-dicarboxylate - **9b** :*

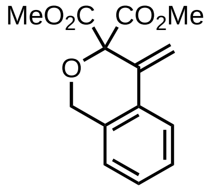

Using the general procedure with dimethyl 2-(but-3-yn-1-yloxy)malonate (0.05 mmol - [0.1M]), $Pd_2(SCS)_2$ (1 mol% Pd), $Mg(OTf)_2$ (5 mol%) and 1,3,5-trimethoxybenzene as internal standard dimethyl 3-methylenedihydrofuran-2,2(3H)-dicarboxylate was formed with >95% yield after 22 h at 75°C as estimated by 1H NMR analysis. Isolated by column chromatography (80:20 Pentane:EtOAc - 12.0mg - 85% Yield). ^1H NMR (500 MHz, $CDCl_3$): δ = 7.68 – 7.63 (m, 1H, H_{Arom}), 7.32 – 7.27 (m, 2H, 2xH_{Arom}), 7.06 – 7.00 (m, 1H, H_{Arom}), 5.96 (s, 1H, H_{aVinyl}), 5.37 (s, 1H, H_{bVinyl}), 4.91 (s, 2H, CH_2-O), 3.84 (s, 6H, 2xCH_3-O). ^{13}C NMR (126 MHz, $CDCl_3$): δ = 168.1 (CO_2Me), 135.3 (C_{Arom}), 132.6 (C_{Arom}), 128.5 (C_{Arom}), 127.8 (CH_{Arom}), 124.4 (CH_{Arom}), 124.4 (CH_{Arom}), 113.5 (CH_{2vinyl}), 84.6 (C_{quat}), 65.7 (C-CH_2-O), 53.5 (CH_3-O). HRMS (DCI-CH4) m/z (%): Calculated: 263.0919 ($C_{14}H_{15}O_5$); Found: 263.0912.

*Diethyl 1-benzyl-3-methylene-6-oxopiperidine-2,2-dicarboxylate - **10b** - CAS [1351364-86-7]:*

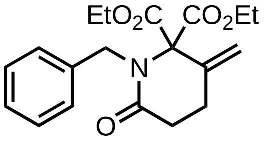

In a 250 mL Schlenk round bottom flask was placed diethyl 2-(*N*-benzylpent-4-ynamido)malonate (1g - 2.89 mmol) along with $Mg(OTf)_2$ (4.6 mg - 0.014 mmol - 0.5 mol%), 5.8 mL of HFIP, 23 mL of $CHCl_3$ finally the $Pd_2(SCS)_2$ (1.4 mg - 0.0029 mmol - 0.1 mol%) was added and the reaction was stirred at 50°C for 16 h. The volatiles were then removed *in vacuo* and the residue was purified by column chromatography (70:30 Pentane:EtOAc) to yield pure diethyl 1-benzyl-3-methylene-6-oxopiperidine-2,2-dicarboxylate (920 mg - 92% Yield). ^{1}H NMR (300 MHz, $CDCl_3$): δ = 7.26 – 7.12 (m, 5H, $\underline{H}_{arom}$), 5.25 (s, 1H, $\underline{H}_{vinyl}$), 5.24 (s, 1H, $\underline{H}_{vinyl}$), 4.61 (s, 2H, Ph-C$\underline{H_2}$-N), 4.09-3.98 (m, 2H, 2x-C$\underline{H_a}H_b$-CH_3), 3.91-3.80 (m, 2H, 2x-$CH_a\underline{H_b}$-CH_3), 2.71-2.55 (m, 4H O=C-C$\underline{H_2}$-CH_2-C_{vinyl} ; O=C-CH_2-C$\underline{H_2}$-C_{vinyl}), 1.11 (t, *J* = 7.1 Hz, 6H, 2xC$\underline{H_3}$-CH_2). Spectroscopic data in accordance with literature.[11]

*Diethyl 1-benzoyl-3-methylenepiperidine-2,2-dicarboxylate - **11b**:*

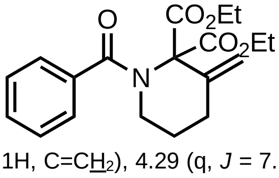

Using the general procedure with diethyl 2-(*N*-(pent-4-yn-1-yl)benzamido)malonate (0.05 mmol - [0.1M]), $Pd_2(SCS)_2$ (1 mol% Pd), $Mg(OTf)_2$ (5 mol%) and 1,3,5-trimethoxybenzene as internal standard, diethyl 1-benzoyl-3-methylenepiperidine-2,2-dicarboxylate was formed with 90% yield after 16 h at 75°C as estimated by ^{1}H NMR analysis. Isolated as by column chromatography (80:20 Pentane:EtOAc - 12.5 mg - 72% Yield). ^{1}H NMR (300 MHz, $CDCl_3$): δ = 7.52 – 7.35 (m, 5H, $\underline{H}_{arom}$), 5.23 (s, 1H, C=C$\underline{H_2}$), 5.20 (s, 1H, C=C$\underline{H_2}$), 4.29 (q, *J* = 7.1 Hz, 4H, C$\underline{H_2}$-CH_3), 3.50 – 3.44 (m, 2H, C$\underline{H_2}$-N), 2.42 – 2.34 (t, *J* = 6.5 Hz, 2H, CH_2-C$\underline{H_2}$-C), 1.85 – 1.72 (quint, *J* = 6.5 Hz, 2H, -CH_2-C$\underline{H_2}$-CH_2-), 1.30 (t, *J* = 7.1 Hz, 6H, C$\underline{H_3}$). ^{13}C NMR (75 MHz, $CDCl_3$): δ = 173.15 ($\underline{C}$=O), 166.66 ($\underline{C}O_2$Et), 139.7($\underline{C}_{quat}$), 136.1 ($\underline{C}_{quat}$), 130.2 ($\underline{CH}_{arom\ para}$), 128.6 ($\underline{CH}_{arom}$), 127.1 ($\underline{CH}_{arom}$), 116.2 (=$\underline{C}H_2$), 73.3 (-$\underline{C}$-(C=O)$_2$), 62.3 (-$\underline{C}H_2$-$CH_3$), 46.3 (-$\underline{C}H_2$-N), 30.8 (-$CH_2$-$\underline{C}H_2$-C), 23.8 (-$CH_2$-$\underline{C}H_2$-$CH_2$-), 14.2 ($\underline{C}H_3$). HRMS (DCI-$CH_4$) m/z (%): Calculated: 346.1654 ($C_{19}H_{24}NO_5$); Found: 346.1648

*Diethyl 1-benzyl-3-methylene-7-oxoazepane-2,2-dicarboxylate - **12b**:*

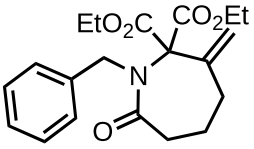

Using general procedure with diethyl 2-(*N*-benzylhex-5-ynamido)malonate (0.05 mmol - [0.1M]), $Pd_2(SCS)_2$ (5 mol% Pd), $Mg(OTf)_2$ (20 mol%) and 1,3,5-trimethoxybenzene as internal standard, diethyl 1-benzyl-3-methylene-7-oxoazepane-2,2-dicarboxylate was formed in 90% yield after 40 h at 75°C as estimated by ^{1}H NMR analysis. Isolated by column chromatography (80:20 Pentane:EtOAc - 70 mg - 78% Yield). ^{1}H NMR (300 MHz, $CDCl_3$): δ = 7.40 – 7.07 (m, 5H, $\underline{H}_{arom}$), 5.63 (s, 1H, $\underline{H}_{vinyl}$), 5.26 (s, 1H, $\underline{H}_{vinyl}$), 4.60 (s, 2H, Ph-C$\underline{H_2}$-N), 4.17-4.06 (m, 2H, 2x-C$\underline{H_a}H_b$-CH_3), 3.95-3.84 (m, 2H, 2x-C$\underline{H_a}H_b$-CH_3), 2.68 (t, *J* = 7.2 Hz, 2H, O=C-C$\underline{H_2}$-CH_2-), 2.47 (t, *J* = 7.0 Hz, 2H, -CH_2-C$\underline{H_2}$-C_{vinyl}), 1.96 – 1.81 (q, 2H, -CH_2-C$\underline{H_2}$-CH_2-), 1.06 (t, *J* = 7.1 Hz, 6H, 2xC$\underline{H_3}$-CH_2). ^{13}C NMR (75 MHz, $CDCl_3$): δ = 175.6 (O=$\underline{C}$-N), 167.3 (2x$\underline{C}O_2$Et), 142.6 ($\underline{C}_{vinyl}$), 138.8 ($\underline{C}_{arom}$), 128.1 (2x$\underline{C}H_{arom}$), 126.8 ($\underline{C}H_{arom\ para}$), 126.6 (2x $\underline{C}H_{arom}$), 117.3 ($\underline{C}H_{2vinyl}$), 77.2 (C_{vinyl}-$\underline{C}$-N), 62.7 (2x$\underline{C}H_2$-CH_3), 52.0 (Ph-$\underline{C}H_2$-N), 33.4 (O=C-$\underline{C}H_2$-CH_2-), 31.9 (-CH_2-$\underline{C}H_2$-C_{vinyl}), 23.1 (CH_2-$\underline{C}H_2$-CH_2), 13.6 ($\underline{C}H_3$-CH_2-). HRMS (HR-ESI) m/z (%): Calculated: 360.1811 ($C_{20}H_{26}NO_5$); Found: 360.1810

*Methyl 1-acetyl-2-ethylcyclopent-2-ene-1-carboxylate - **13b** - CAS [811784-57-3]:*

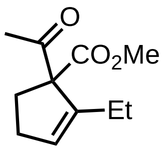

Using the general procedure, with Methyl 2-acetyloct-5-ynoate (0.25 mmol - [0.5M]), $Pd_2(SCS)_2$ (1 mol% Pd), $Mg(OTf)_2$ (5 mol%) and 1,3,5-trimethoxybenzene as internal standard methyl 1-acetyl-2-ethylcyclopent-2-ene-1-carboxylate was formed with 95% yield after 25 h at 25°C as estimated by ^{1}H NMR analysis. Isolated by column chromatography (95:05 Pentane:EtOAc - 43 mg - 88% Yield). ^{1}H NMR (300 MHz, $CDCl_3$): δ = 5.73 (quint, *J* = 2.1 Hz, 1H, $\underline{H}_{Vinyl}$), 3.75 (s, 3H, O-C$\underline{H_3}$), 2.68 – 2.56 (m, 1H, -C-$CH_b\underline{H_a}$-CH_2), 2.47 – 2.35 (m, 2H, CH_2-C$\underline{H_2}$-CH_{Vinyl}), 2.29 – 2.05 (m, 3H, -C$\underline{H_2}$-CH_3; -C-C$\underline{H_b}H_a$-CH_2), 2.16 (s, 3H, C$\underline{H_3}$-C=O) 1.10 (t, *J* = 7.3 Hz, 3H, CH_3-CH_2). Spectroscopic data in accordance with literature.[22]

Diethyl 1-benzyl-3-methyl-5-oxo-1,5-dihydro-2H-pyrrole-2,2-dicarboxylate - ***14b****:*

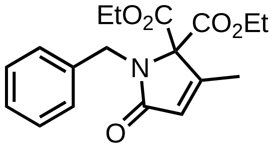

Using the general procedure with diethyl 2-(*N*-benzylbut-2-ynamido)malonate (0.25 mmol - [0.5M]), $Pd_2(SCS)_2$ (1 mol% Pd), $Mg(OTf)_2$ (5 mol%) (methoxymethyl)benzene as internal standard diethyl 1-benzyl-3-methyl-5-oxo-1,5-dihydro-2H-pyrrole-2,2-dicarboxylate was formed with >95% yield after 1 h 30 min at 25°C as estimated by ^{1}H NMR analysis. Isolated by column chromatography (70:30 Pentane:EtOAc - 74 mg - 89% Yield). ^{1}H NMR (300 MHz, $CDCl_3$): δ = 77.28 –7.09 (m, 5H, H_{arom}), 6.09 (q, *J* = 1.6 Hz, 1H, CH_{vinyl}), 4.77 (s, 2H, CH_2-Ph), 4.04 – 3.79 (m, 4H, 2xCH_2-CH_3), 2.12 (d, *J* = 1.6 Hz, 3H, CH_3-C), 1.10 (t, *J* = 7.2 Hz, 6H, 2xCH_3-CH_2-). ^{13}C NMR (75 MHz, $CDCl_3$): δ = 171.8 (O=C-N), 165.2 (CO_2Et), 154.0 (CH_3-C_{quat}-CH), 137.7 (C_{arom}), 128.4 (2xCH_{arom}), 127.2 (2xCH_{arom}), 127.1 ($CH_{arom\ para}$), 125.7 (O=C-CH=C_{quat}), 77.9 (N-C-($CO_2Et)_2$), 62.8 (CH_2-CH_3), 45.1 (CH_2-Ph), 14.5 (CH_3-C_{quat}), 13.8 (CH_3-CH_2-). HRMS (DCI-CH4) m/z (%): Calculated: 332.1498 ($C_{18}H_{22}NO_5$); Found: 332.1511

Diethyl (Z)-1-benzyl-3-ethylidene-5-oxopyrrolidine-2,2-dicarboxylate - ***15b*** *- CAS [1383445-15-5]:*

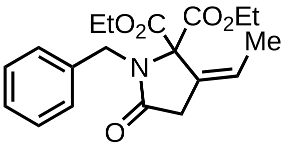

Using the general procedure, with diethyl 2-(*N*-benzylpent-3-ynamido)malonate (0.25 mmol - [0.5M]), $Pd_2(SCS)_2$ (1 mol% Pd), $Mg(OTf)_2$ (5 mol%) and 1,2,4,5-tetramethylbenzene as internal standard diethyl (*Z*)-1-benzyl-3-ethylidene-5-oxopyrrolidine-2,2-dicarboxylate was formed with 85% yield after 3 h at 25°C as estimated by ^{1}H NMR analysis. Isolated by column chromatography (80:20 Pentane:EtOAc - 72 mg - 83% Yield). ^{1}H NMR (300 MHz, $CDCl_3$): δ = 7.30 – 7.14 (m, 5H, H_{arom}), 5.75 (qt, *J* = 7.3, 2.3 Hz, 1H, H_{vinyl}), 4.64 (s, 2H, Ph-CH_2-N), 4.00-3.90 (m, 2H, 2x-CH_aH_b-CH_3), 3.86-3.75 (m, 2H, 2x-CH_aH_b-CH_3), 3.24 (quint, *J* = 2.2 Hz, 2H, -C-CH_2-C), 1.67 (dt, *J* = 7.3, 2.2 Hz, 3H -CH-CH_3), 1.12 (t, *J* = 7.1 Hz, 6H, 2xCH_3-CH_2). Spectroscopic data in accordance with literature.[11]

Diethyl (Z)-1-benzyl-5-oxo-3-propylidenepyrrolidine-2,2-dicarboxylate - ***16b*** *- CAS [1383445-16-6]:*

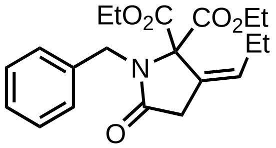

Using the general procedure, with diethyl 2-(*N*-benzylhex-3-ynamido)malonate (0.25 mmol - [0.5M]), $Pd_2(SCS)_2$ (1 mol% Pd), $Mg(OTf)_2$ (5 mol%) and (methoxymethyl)benzene as internal standard diethyl (*Z*)-1-benzyl-3-ethylidene-5-oxopyrrolidine-2,2-dicarboxylate was formed with 84% yield after 24 h at 25°C as estimated by ^{1}H NMR analysis. Isolated by column chromatography (80:20 Pentane:EtOAc - 75 mg - 83% Yield). ^{1}H NMR (300 MHz, $CDCl_3$): δ = 7.30 – 7.11 (m, 5H, H_{arom}), 5.59 (tt, *J* = 7.8, 2.2 Hz, 1H, H_{vinyl}), 4.64 (s, 2H, Ph-CH_2-N), 4.00-3.89 (m, 2H, 2x-CH_aH_b-CH_3), 3.84-3.73 (m, 2H, 2x-CH_aH_b-CH_3), 3.24 (pseudo-q, *J* = 2.0 Hz, 2H, C-CH_2-C), 2.15-2.00 (m, 2H -C_{vinyl}-CH_2-CH_3), 1.11 (t, *J* = 7.2 Hz, 6H, 2xOOC-CH_3-CH_2), 0.93 (t, *J* = 7.4 Hz, 3H, -C_{vinyl}-CH_2-CH_3). Spectroscopic data in accordance with literature.[11]

Diethyl (Z)-1-benzyl-3-(cyclohexylmethylene)-5-oxopyrrolidine-2,2-dicarboxylate - ***17b****:*

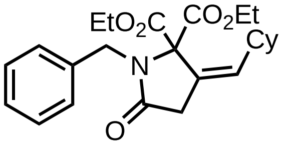

Using the general procedure, with diethyl 2-(*N*-benzyl-4-cyclohexylbut-3-ynamido)malonate (0.25 mmol - [0.5M]), $Pd_2(SCS)_2$ (5 mol% Pd), $Mg(OTf)_2$ (20 mol%) and 1,2,4,5-tetramethylbezene as internal standard diethyl (*Z*)-1-benzyl-3-(cyclohexylmethylene)-5-oxopyrrolidine-2,2-dicarboxylate was obtained with 76% yield after 32 h at 120 °C (Isolated yield 52%). During this experiment, 17% of (*E*) isomer was also formed (Isolated yield 10%). ***Z*-isomer:** ^{1}H NMR (300 MHz, $CDCl_3$): δ = 7.30-7.12 (m, 5H, H_{arom}), 5.40 (dt, *J* = 11.2, 2.2 Hz, 1H, H_{vinyl}), 4.61 (s, 2H, Ph-CH_2-N), 4.01-3.88 (m, 2H, 2x-CH_aH_b-CH_3), 3.83-3.69 (m, 2H, 2x-CH_aH_b-CH_3), 3.22 (d, *J* = 2.2 Hz, 2H, O=C-CH_2-C-), 2.18 (qt, *J* = 11.2, 3.4 Hz, 1H, CH_{Cy}), 1.74-1.51 (m, 6H, 3xCH_{2Cy}), 1.11 (t, *J* = 7.1 Hz, 6H, 2xCH_3-CH_2), 1.31-0.88 (m, 4H, 2xCH_{2Cy}). ^{13}C NMR (75 MHz, $CDCl_3$): δ = 174.1 (O=C-N), 167.3 (CO_2Et), 136.7 (C_{arom}), 136.5 (CH_{vinyl}), 128.2 (2xCH_{arom}), 128.0 (2xCH_{arom}), 127.3 ($CH_{arom\ para}$), 123.4 (-C=CH_{vinyl}), 75.0 (C_{quat}, Not visible in ^{13}C-jmod but in HMBC), 62.6 (2xCH_2-CH_3), 45.7 (Ph-CH_2-N), 38.8 (CH_{Cy}), 36.4 (C-CH_2-C=), 31.7 (2xCH_{2Cy}), 26.0 (CH_{2Cy}), 25.6 (2xCH_{2Cy}), 13.8 (2xCH_3-CH_2-). HRMS (DCI-CH_4) m/z (%): Calculated: 414.2280 ($C_{24}H_{32}NO_5$); Found: 414.2285. ***E*-isomer:** ^{1}H NMR (300 MHz, $CDCl_3$): δ = 7.30 – 7.13 (m, 5H, H_{arom}), 5.72 (dt, *J* = 9.6, 2.8 Hz, 1H, H_{vinyl}), 4.70 (s, 2H, Ph-CH_2-N), 3.95-3.84 (m, *J* = 7.1, 1.4 Hz, 4H, 2xCH_2-CH_3), 3.21 (d, *J* = 2.8 Hz, 2H, O=C-CH_2-C-), 2.17 – 1.99 (m, 1H, CH_{Cy}), 1.80 - 1.15 (m, 10H, 5xCH_{2Cy}), 1.08 (t, *J* = 7.1 Hz, 6H, 2xCH_3-CH_2). ^{13}C NMR (126 MHz, $CDCl_3$): δ = 174.1 (O=C-N), 167.4 (CO_2Et), 136.9 (C_{arom}), 136.0 (CH_{vinyl}), 128.3 (2xCH_{arom}), 127.5 (2xCH_{arom}), 127.2 ($CH_{arom\ para}$), 124.9 (-C=CH_{vinyl}), 75.7 (C_{quat}), 62.3 (2xCH_2-CH_3), 46.0 (Ph-CH_2-N), 39.1 (CH_{Cy}), 32.9 (C-CH_2-C=), 32.0 (2xCH_{2Cy}), 26.0 (2xCH_{2Cy}), 25.7 (2xCH_{2Cy}), 13.8 (2xCH_3-CH_2-). HRMS (DCI-CH_4) m/z (%): Calculated: 414.2280 ($C_{24}H_{32}NO_5$); Found: 414.2280.

The structures of **17b-(*Z*)** and **17b-(*E*)** have been determined based on ^{13}C-^{13}C ADEQUATE and NOESY NMR analysis.

Diethyl (Z)-1-benzyl-3-benzylidene-5-oxopyrrolidine-2,2-dicarboxylate - ***18b*** *- CAS [1383445-19-9]:*

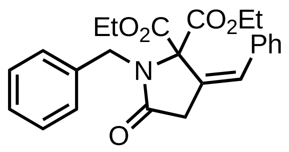

Using the general procedure, with diethyl 2-(*N*-benzyl-4-phenylbut-3-ynamido)malonate (0.25 mmol - [0.5M]), $Pd_2(SCS)_2$ (5 mol% Pd), $Mg(OTf)_2$ (20 mol%) and 1,2,4,5-tetramethylbezene as internal standard diethyl (*Z*)-1-benzyl-3-benzylidene-5-oxopyrrolidine-2,2-dicarboxylate was formed with 79% yield after 16 h at 75°C as estimated by ^1H NMR analysis. Isolated by column chromatography (using a gradient from 80:20 to 70:30 Pentane:EtOAc - 66 mg - 65% Yield). During this experiment 8% of (*E*) isomer was also formed (Confirmed by NOESY experiment - Isolated yield - 5 mg - 5%). ***Z*-isomer:** ^1H NMR (500 MHz, $CDCl_3$): δ = 7.47 – 7.42 (m, 2H, H_{arom}), 7.30 – 7.25 (m, 2H, H_{arom}), 7.24 – 7.13 (m, 6H, H_{arom}), 6.69 (t, *J* = 2.2 Hz, 1H, H_{vinyl}), 4.61 (s, 2H, Ph-CH_2-N), 3.72-3.66 (m, 2H, 2x-CH_aH_b-CH_3), 3.60-3.54 (m, 2H, 2x-CH_aH_b-CH_3), 3.39 (d, *J* = 2.2 Hz, 2H, C-CH_2-C), 0.87 (t, *J* = 7.2 Hz, 6H, 2xOOC-CH_2-CH_3). ^{13}C NMR (126 MHz, $CDCl_3$): δ = 173.2 (N-C=O), 166.2 (O-C=O), 136.6 (CH_2-C_{arom}), 135.1 (CH-C_{arom}), 129.8 (CH_{vinyl}), 128.8 (2xCH_{arom}), 128.4 (2xCH_{arom}), 128.2 (4xCH_{arom}), 128.0 (CH_{arom}), 127.8 (C_{quat}=CH_{vinyl}), 127.4 (CH_{arom}), 75.4 (N-C-($CO_2Et)_2$), 62.5 (2xCH_2-CH_3), 45.9 (CH_2-Ph), 38.9 (O=C-CH_2-), 13.5 (2xCH_3). ***E*-isomer:** ^1H NMR (500 MHz, $CDCl_3$): δ = 7.42 – 7.37 (m, 2H, H_{arom}), 7.35 – 7.27 (m, 4H, H_{arom}), 7.26 – 7.16 (m, 4H, H_{arom}), 6.83 (t, *J* = 2.8 Hz, 1H, H_{vinyl}), 4.76 (s, 2H, Ph-CH_2-N), 4.01 – 3.85 (m, 4H, 2xCH_2-CH_3), 3.56 (d, *J* = 2.8 Hz, 2H, C-CH_2-C), 1.12 (t, *J* = 7.1 Hz, 6H, 2xCH_3-CH_2). ^{13}C NMR (126 MHz, $CDCl_3$): δ = 173.8 (N-C=O), 167.2 (O-C=O), 136.8 (CH_2-C_{arom}), 135.8 (CH-C_{arom}), 129.3 (CH_{vinyl}), 128.9 (2xCH_{arom}), 128.8 (2xCH_{arom}), 128.4 (2xCH_{arom}), 128.3 (CH_{arom}), 127.5 (2xCH_{arom} + C_{quat}=CH_{vinyl}), 127.3 (CH_{arom}), 77.4 (N-C-($CO_2Et)_2$), 62.7 (2xCH_2-CH_3), 46.1 (CH_2-Ph), 35.2 (O=C-CH_2-), 13.9 (2xCH_3).

The structures of **18b-(*Z*)** and **18b-(*E*)** have been determined based on ^{13}C and NOESY NMR analyses.

Diethyl (Z)-1-benzyl-3-(2,2-dimethylpropylidene)-5-oxopyrrolidine-2,2-dicarboxylate - ***19b****:*

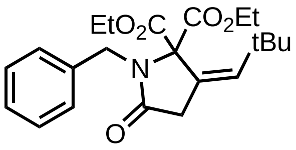

Using the general procedure, with diethyl 2-(*N*-benzyl-5,5-dimethylhex-3-ynamido)malonate (0.25 mmol - [0.5M]), $Pd_2(SCS)_2$ (5 mol% Pd), $Mg(OTf)_2$ (20 mol%) and 1,2,4,5-tetramethylbezene as internal standard. diethyl (*E*)-1 benzyl-3-(2,2-dimethylpropylidene)-5-oxopyrrolidine-2,2-dicarboxylate was formed with 81% yield after 64 h at 120°C as estimated by ^1H NMR analysis. Isolated by column chromatography (using a gradient from 80:20 to 70:30 Pentane:EtOAc - 58 mg - 60% Yield). During this experiment 7% of (*Z*) isomer was also formed (Isolated yield - 6 mg - 6%). ***E*-isomer:** ^1H NMR (300 MHz, $CDCl_3$): δ = 7.32 – 7.09 (m, 5H, H_{arom}), 5.81 (t, *J* = 2.8 Hz, 1H, C-CH-C), 4.70 (s, 2H, CH_2-Ph), 3.90 (q, *J* = 7.1 Hz, 4H, 2xCH_2-CH_3), 3.38 (d, *J* = 2.8 Hz, 2H, O=C-CH_2-C), 1.11 (s, 9H, $(CH_3)_3$-C), 1.09 (t, *J* = 7.1 Hz, 5H, CH_3-CH_2). ^{13}C NMR (75 MHz, $CDCl_3$): δ = 174.5 (N-C=O), 167.5 (O-C=O), 140.5 (C-CH-C), 136.8 (C_{arom}), 128.3 (2xCH_{arom}), 127.6 (2xCH_{arom}), 127.2 ($CH_{arom\ para}$), 123.6 (CH_2-C-CH), 77.4 (C-($CO_2Et)_2$), 62.2 (CH_2-CH_3), 45.9 (CH_2-Ph), 34.0 ($(CH_3)_3$-C-CH), 33.6 (C-CH_2-C=O), 29.8 ($(CH_3)_3$-C), 13.8 (CH_3-CH_2). HRMS ($DCI-CH_4$) m/z (%): Calculated: 388.2126 ($C_{22}H_{30}NO_5$); Found: 388.2124. ***Z*-isomer:** ^1H NMR (300 MHz, $CDCl_3$): δ = 7.30-7.17 (m, 5H, H_{arom}), 5.52 (t, J = 2.2 Hz, 1H, C-CH-C), 4.52 (s, 2H, CH_2-Ph), 4.09-3.84 (m, 2H, 2xCH_aH_b-CH_3), 3.79-3.62 (m, 2H, 2xCH_aH_b-CH_3), 3.32 (d, J = 2.2 Hz, 2H, O=C-CH_2-C), 1.11 (t, J = 7.2 Hz, 6H, CH_3-CH_2), 1.04 (s, 9H, $(CH_3)_3$-C). ^{13}C NMR (126 MHz, $CDCl_3$): δ = 173.8 (N-C=O), 166.9 (O-C=O), 140.1 (C-CH-C), 136.4 (C_{arom}), 128.4 (2xCH_{arom}), 128.3 (2xCH_{arom}), 127.4 ($CH_{arom\ para}$), 122.1 (CH_2-C-CH), 74.1 (C-($CO_2Et)_2$), 62.4 (CH_2-CH_3), 45.7 (CH_2-Ph), 39.3 (C-CH_2-C=O), 34.7 ($(CH_3)_3$-C-CH), 29.6 ($(CH_3)_3$-C), 13.8 (CH_3-CH_2). HRMS ($DCI-CH_4$) m/z (%): Calculated: 388.2126 ($C_{22}H_{30}NO_5$); Found: 388.2125.

The structures of **19b-(*E*)** and **19b-(*Z*)** have been determined based on ^{13}C-^{13}C ADEQUATE, NOESY, NOESY ZQF Selective and Double selective-HSQMBC-IPAP NMR analyses. Residual COSY signal on the NOESY analysis avoided discrimination between *Z* and *E* stereochemistry. This was finally stated thanks to NOESY ZQF Selective analyses and to the determination of the $^3J_{CH}$ values by Double selective-HSQMBC-IPAP. Attribution of the stereochemistry was done taking into account that $^3J_{CH}$ (*trans*) > $^3J_{CH}$ (*cis*).[23]

	Major product ***E*-isomer**	Minor product ***Z*-isomer**
^3J C_{IV}–H	6.5 Hz	11.4 Hz
^3J CH_2–H	8.9 Hz	7.6 Hz

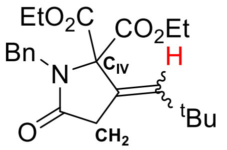

References

1 N. Nebra, J. Monot, R. Shaw, B. Martin-Vaca and D. Bourissou, *ACS Catal.*, 2013, **3**, 2930–2934.
2 N. Á. Espinosa-Jalapa, D. Ke, N. Nebra, L. Le Goanvic, S. Mallet-Ladeira, J. Monot, B. Martin-Vaca and D. Bourissou, *ACS Catal.*, 2014, **4**, 3605–3611.
3 D. Ke, N. Á. Espinosa, S. Mallet-Ladeira, J. Monot, B. Martin-Vaca and D. Bourissou, *Adv. Synth. Catal.*, 2016, **358**, 2324–2331.
4 N. Nebra, J. Lisena, N. Saffon, L. Maron, B. Martin-Vaca and D. Bourissou, *Dalton Trans.*, 2011, **40**, 8912–8921.
5 J. Monot, P. Brunel, C. E. Kefalidis, N. Á. Espinosa-Jalapa, L. Maron, B. Martin-Vaca and D. Bourissou, *Chem. Sci.*, 2016, **7**, 2179–2187.
6 J. A. Greenberg and T. Sammakia, *J. Org. Chem.*, 2017, **82**, 3245–3251.
7 N. Monteiro, J. Gore and G. Balme, *Tetrahedron*, 1992, **48**, 10103–10114.
8 P. Cruciani, R. Stammler, C. Aubert and M. Malacria, *J. Org. Chem.*, 1996, **61**, 2699–2708.
9 E. C. Taylor, J. E. Macor and L. G. French, *J. Org. Chem.*, 1991, **56**, 1807–1812.
10 I. Ugarriza, U. Uria, L. Carrillo, J. L. Vicario and E. Reyes, *Chem. - Eur. J.*, 2014, **20**, 11650–11654.
11 H. A. Keane, W. Hess and J. W. Burton, *Chem. Commun.*, 2012, **48**, 6496–6498.
12 W. Hess and J. W. Burton, *Chem. – Eur. J.*, 2010, **16**, 12303–12306.
13 J. Waser, B. Gaspar, Hisanori Nambu and E. M. and Carreira*, , DOI:10.1021/JA062355+.
14 X. Wang and K. Nozaki, *J. Am. Chem. Soc.*, 2018, **140**, 15635–15640.
15 K. Takahashi, M. Midori, K. Kawano, J. Ishihara and S. Hatakeyama, *Angew. Chem. Int. Ed.*, 2008, **47**, 6244–6246.
16 A. Suárez and G. C. Fu, *Angew. Chem. Int. Ed.*, 2004, **43**, 3580–3582.
17 H. Liu, D. Leow, K.-W. Huang and C.-H. Tan, *J. Am. Chem. Soc.*, 2009, **131**, 7212–7213.
18 M. Blümel, D. Hack, L. Ronkartz, C. Vermeeren and D. Enders, *Chem. Commun.*, 2017, **53**, 3956–3959.
19 C.-L. Deng, T. Zou, Z.-Q. Wang, R.-J. Song and J.-H. Li, *J. Org. Chem.*, 2009, **74**, 412–414.
20 C.-L. Deng, R.-J. Song, Y.-L. Liu and J.-H. Li, *Adv. Synth. Catal.*, 2009, **351**, 3096–3100.
21 N. Tsukada and Y. Yamamoto, *Angew. Chem. Int. Ed. Engl.*, 1997, **36**, 2477–2480.
22 S. T. Staben, J. J. Kennedy-Smith and F. D. Toste, *Angew. Chem. Int. Ed.*, 2004, **43**, 5350–5352.
23 U. R. Couto, A. Navarro-Vázquez and C. F. Tormena, *Magn. Reson. Chem.*, 2019, **57**, 939–945.

^{1}H-NMR ($CDCl_3$): **1a**

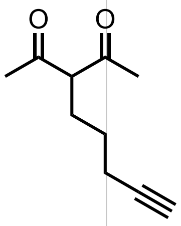

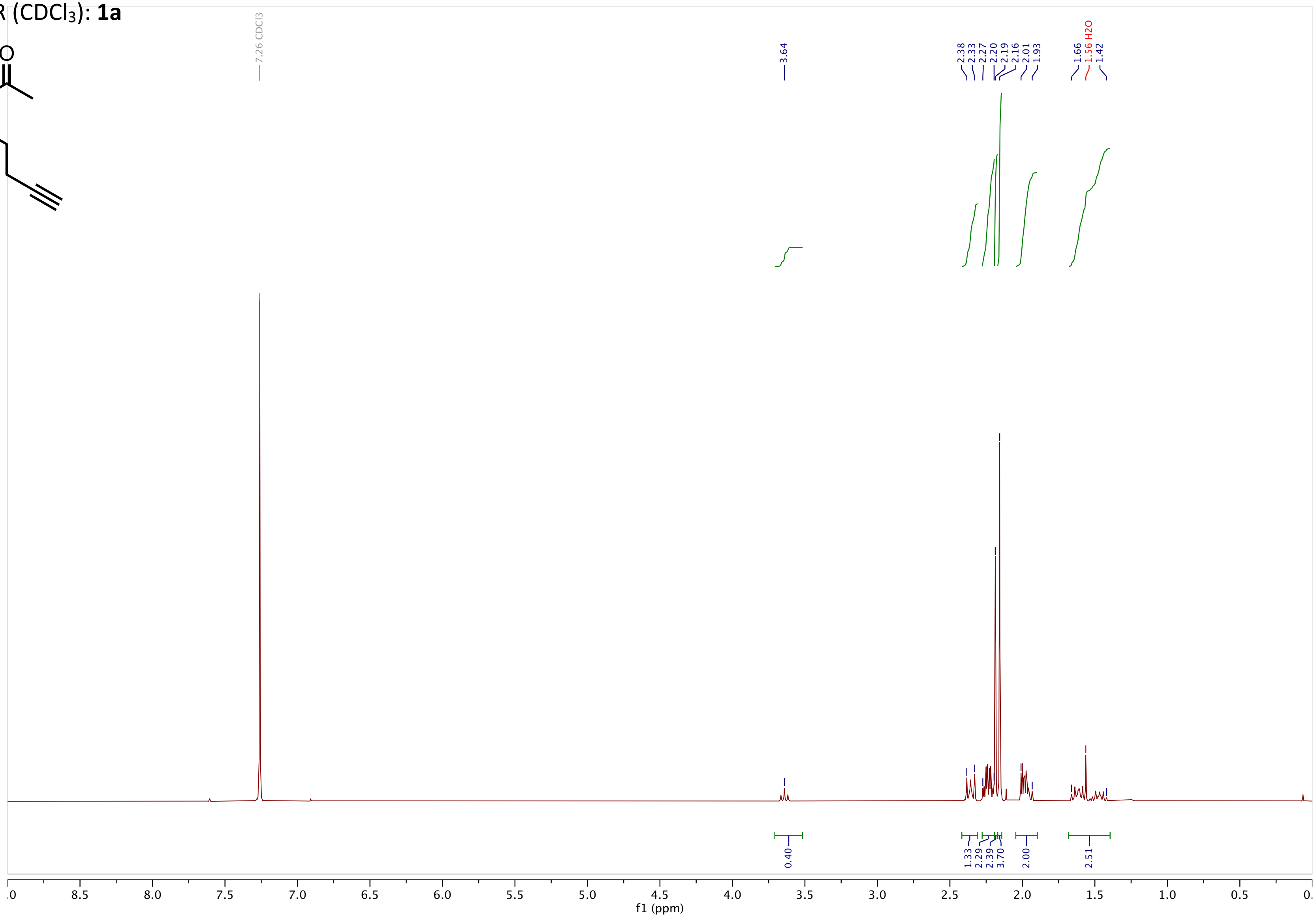

^{1}H-NMR ($CDCl_3$): **2a**

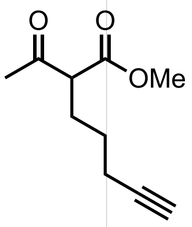

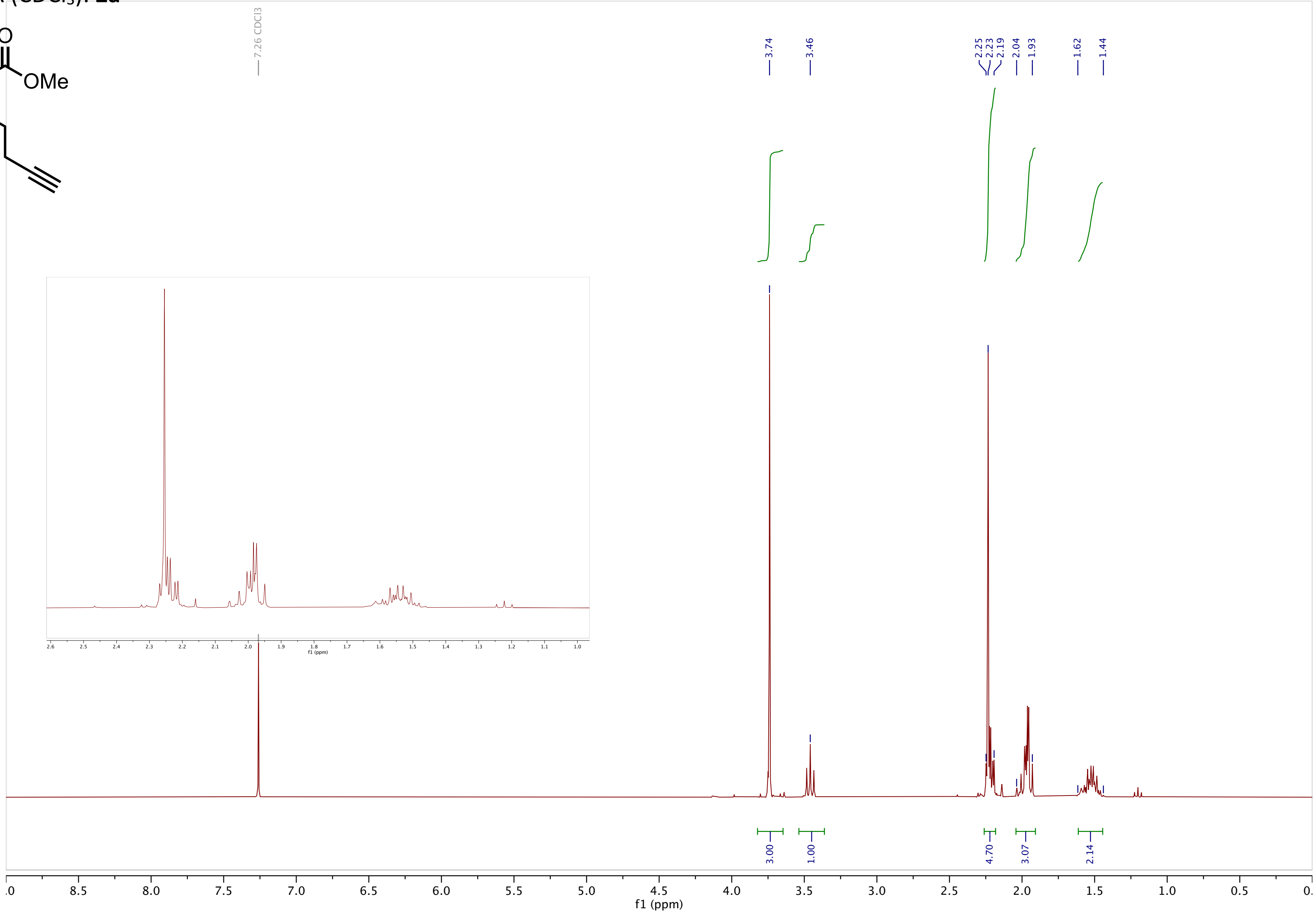

^{1}H-NMR ($CDCl_3$): **3a**

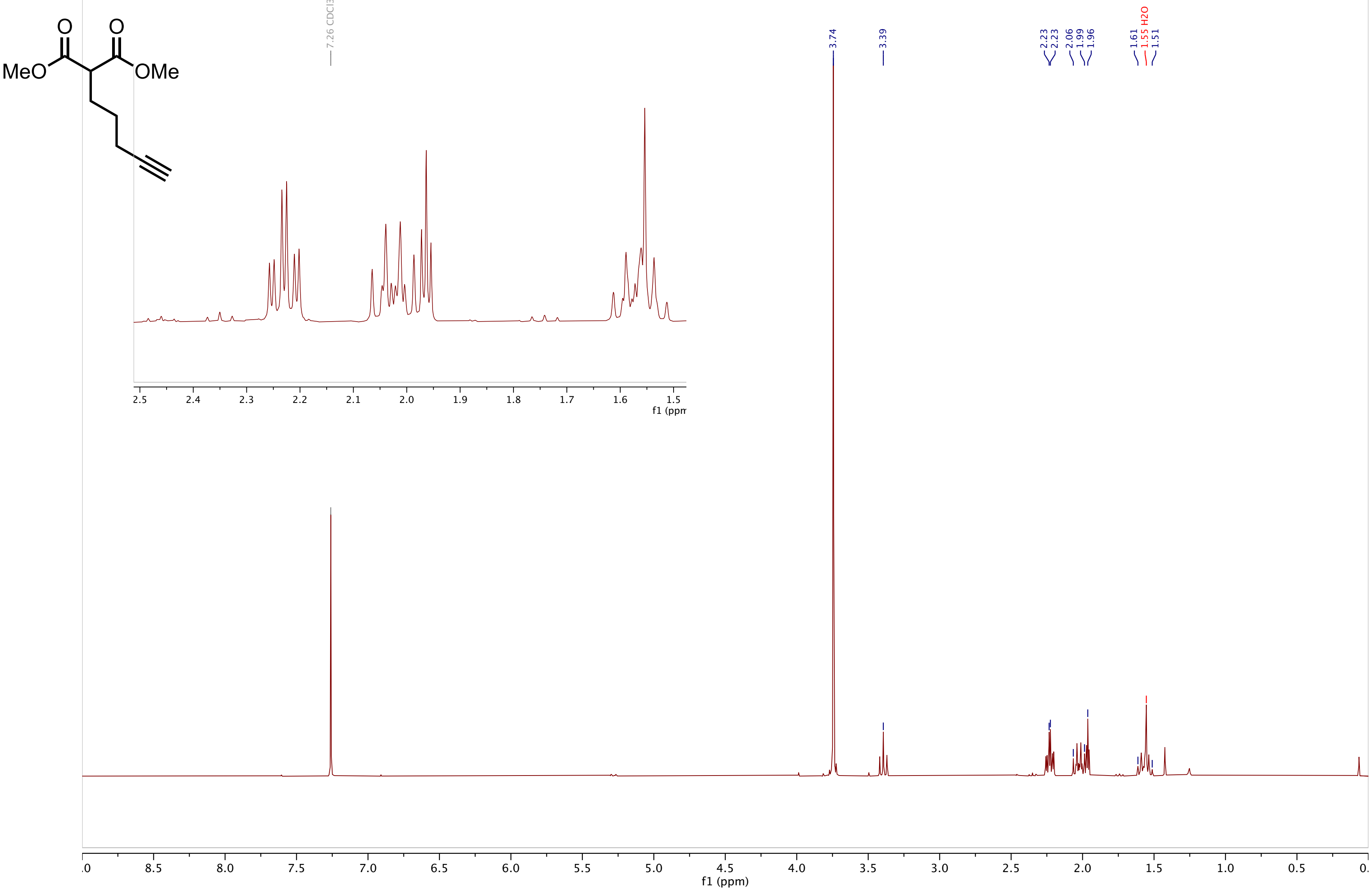

^{1}H-NMR ($CDCl_3$): **4a**

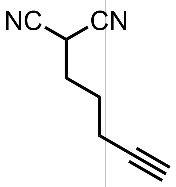

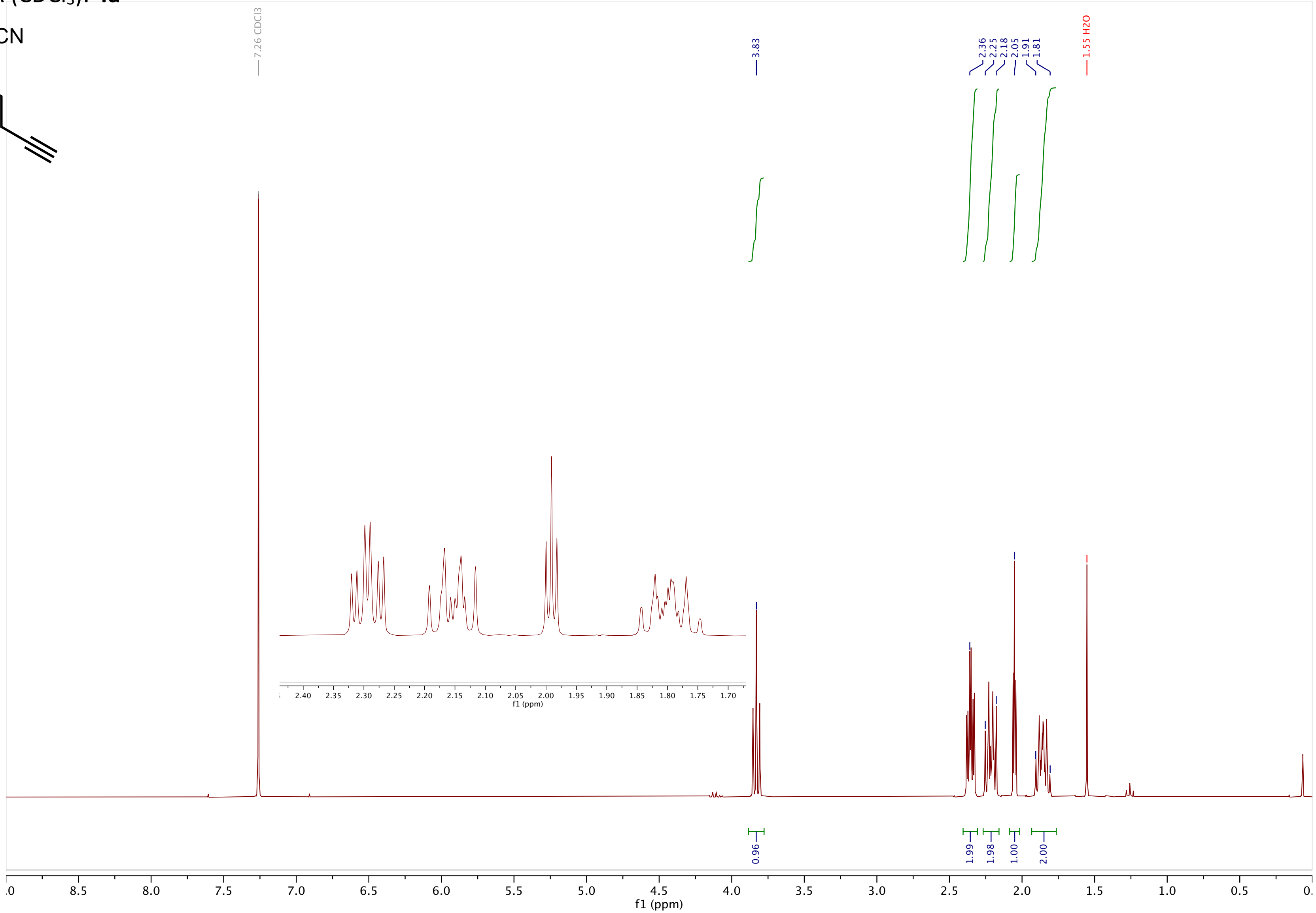

^1H-NMR ($CDCl_3$)

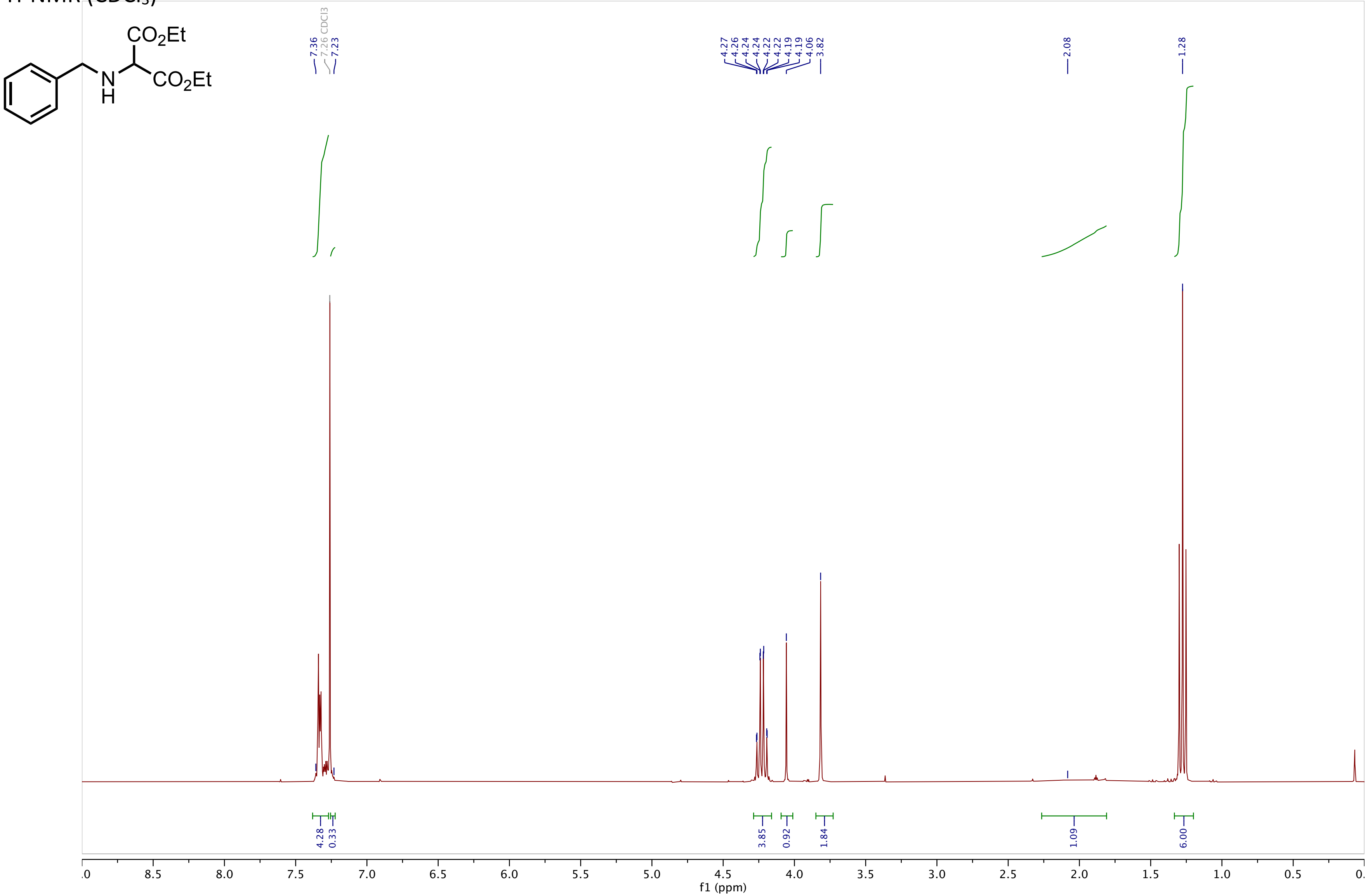

7.36
7.26 CDCl3
7.23
4.27
4.26
4.24
4.24
4.22
4.22
4.19
4.19
4.06
3.82
2.08
1.28
4.28
0.33
3.85
0.92
1.84
1.09
6.00
f1 (ppm)

^{1}H-NMR ($CDCl_3$): **5a**

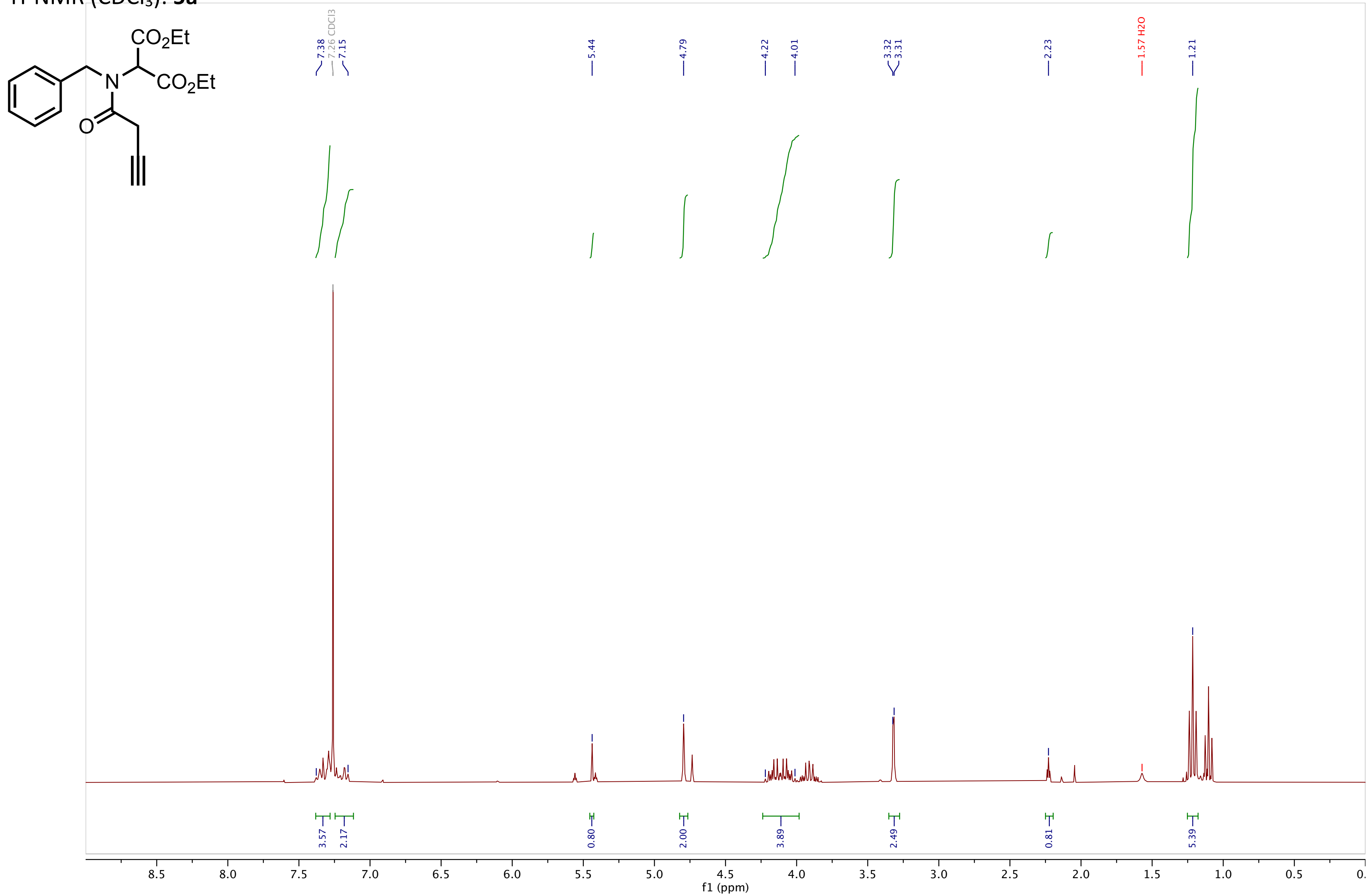

^1H-NMR ($CDCl_3$)

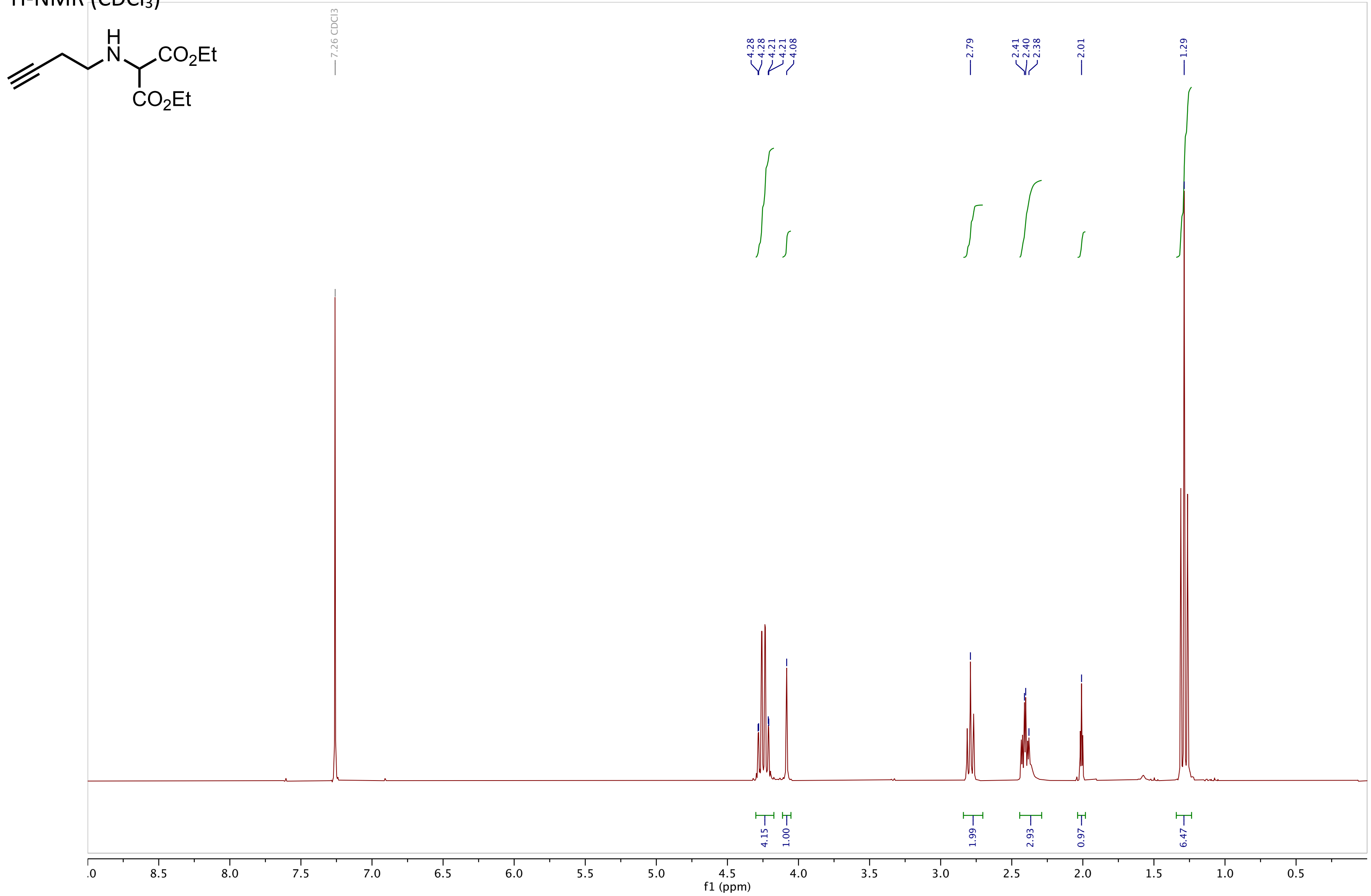

CO2Et
CO2Et
7.26 CDCl3
4.28
4.28
4.21
4.21
4.08
2.79
2.41
2.40
2.38
2.01
1.29
4.15
1.00
1.99
2.93
0.97
6.47
f1 (ppm)

^{1}H-NMR (CD_3OD): **6a**

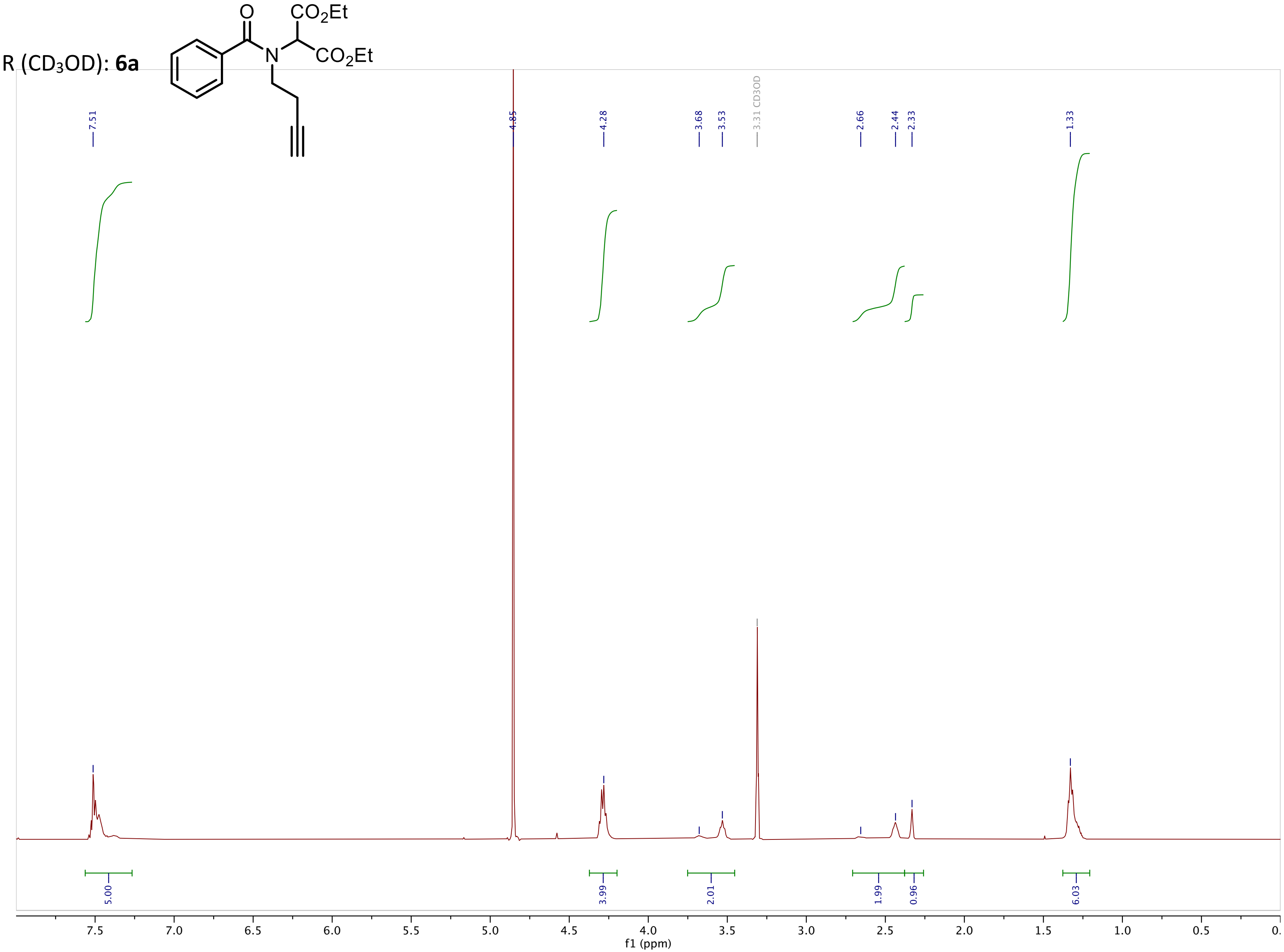

^{13}C-zgpg (CD_3OD): **6a**

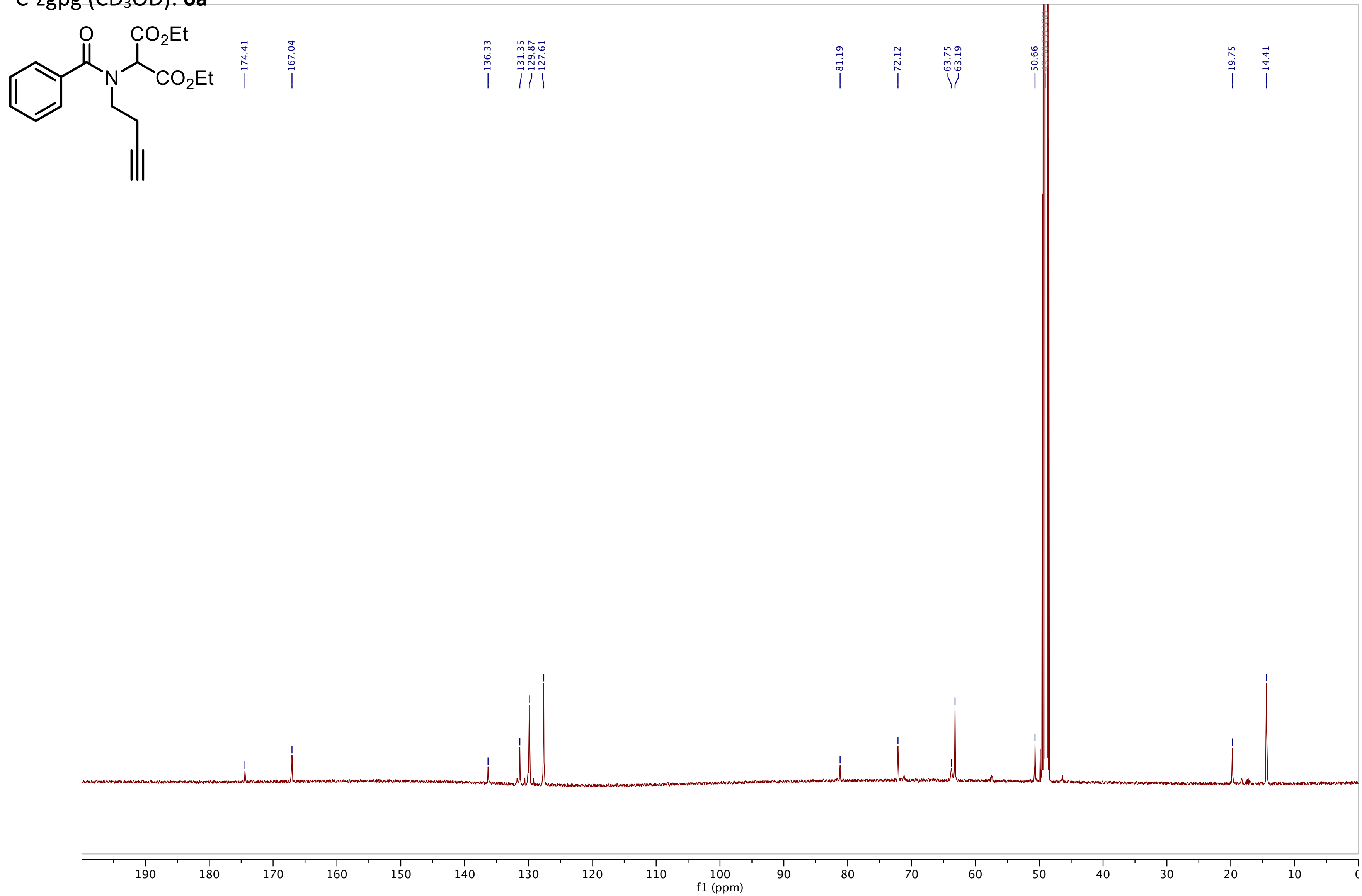

^{1}H-NMR ($CDCl_3$)

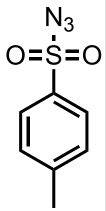
N3
O=S=O

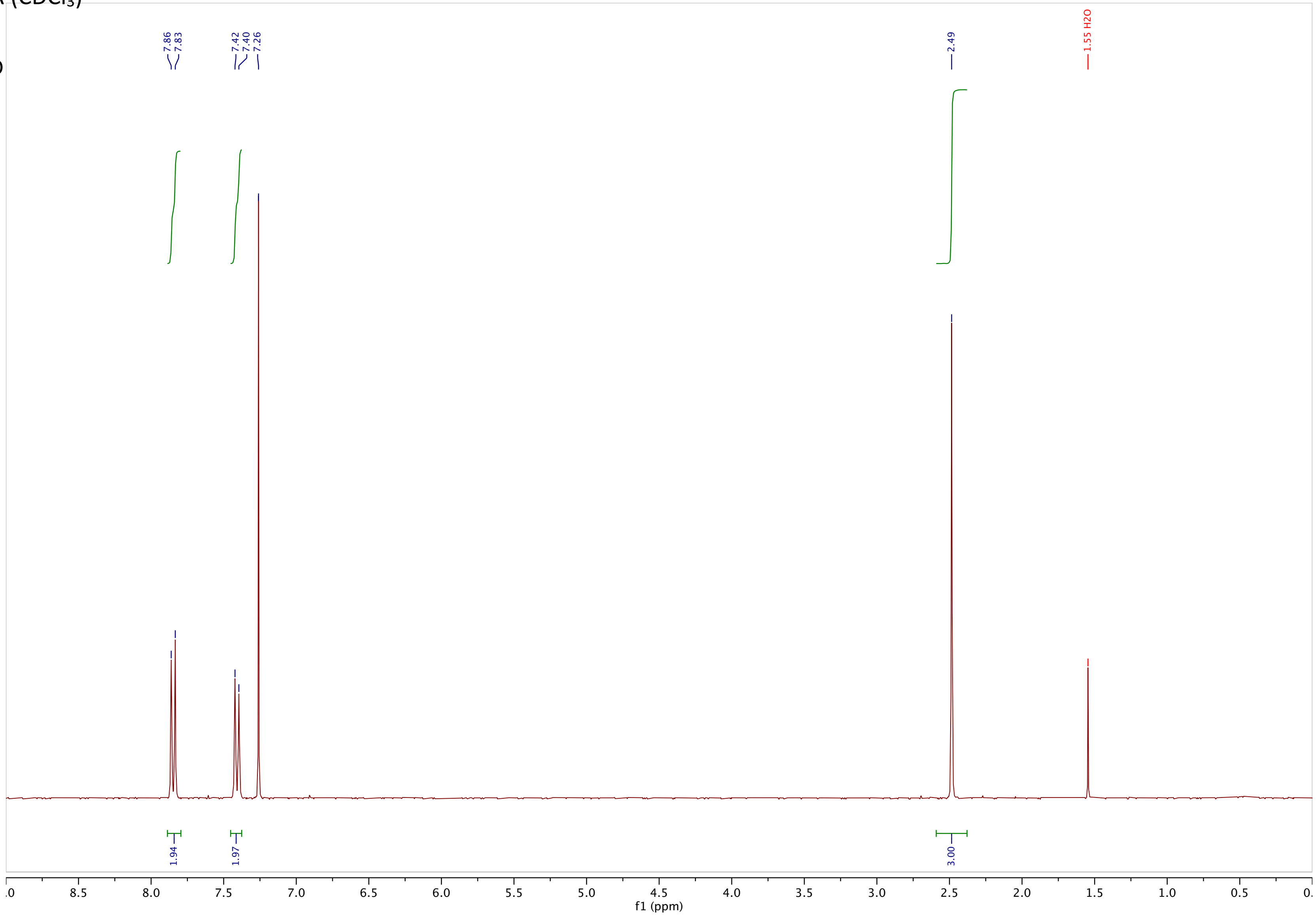
7.86
7.83
7.42
7.40
7.26
2.49
1.55 H2O
1.94
1.97
3.00
.0
8.5
8.0
7.5
7.0
6.5
6.0
5.5
5.0
4.5
4.0
3.5
3.0
2.5
2.0
1.5
1.0
0.5
0.
f1 (ppm)

^{1}H-NMR ($CDCl_3$)

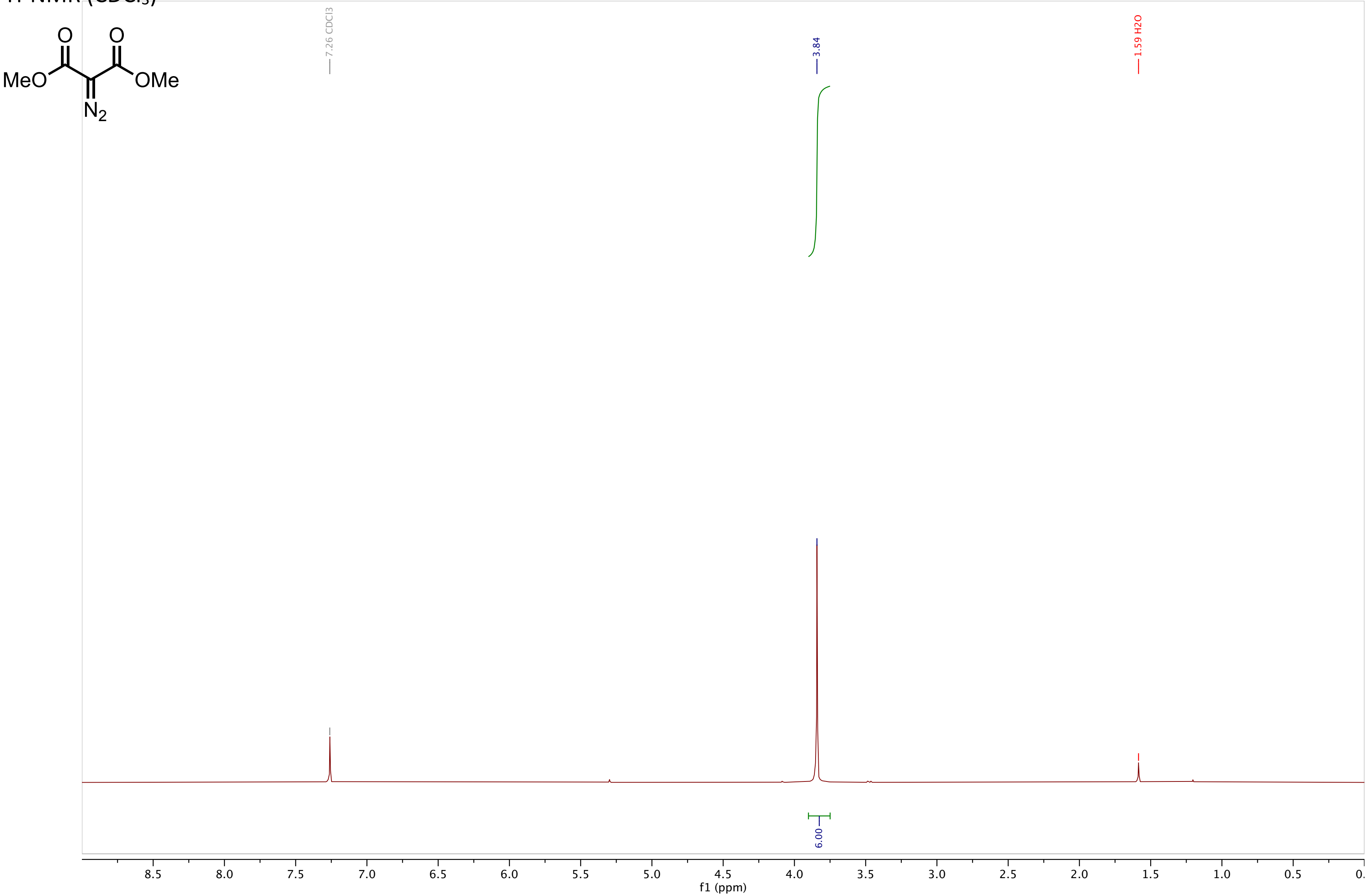

MeO
OMe
O
O
N2
7.26 CDCl3
3.84
1.59 H2O
6.00
8.5
8.0
7.5
7.0
6.5
6.0
5.5
5.0
4.5
4.0
3.5
3.0
2.5
2.0
1.5
1.0
0.5
f1 (ppm)

^{1}H-NMR ($CDCl_3$): **7a**

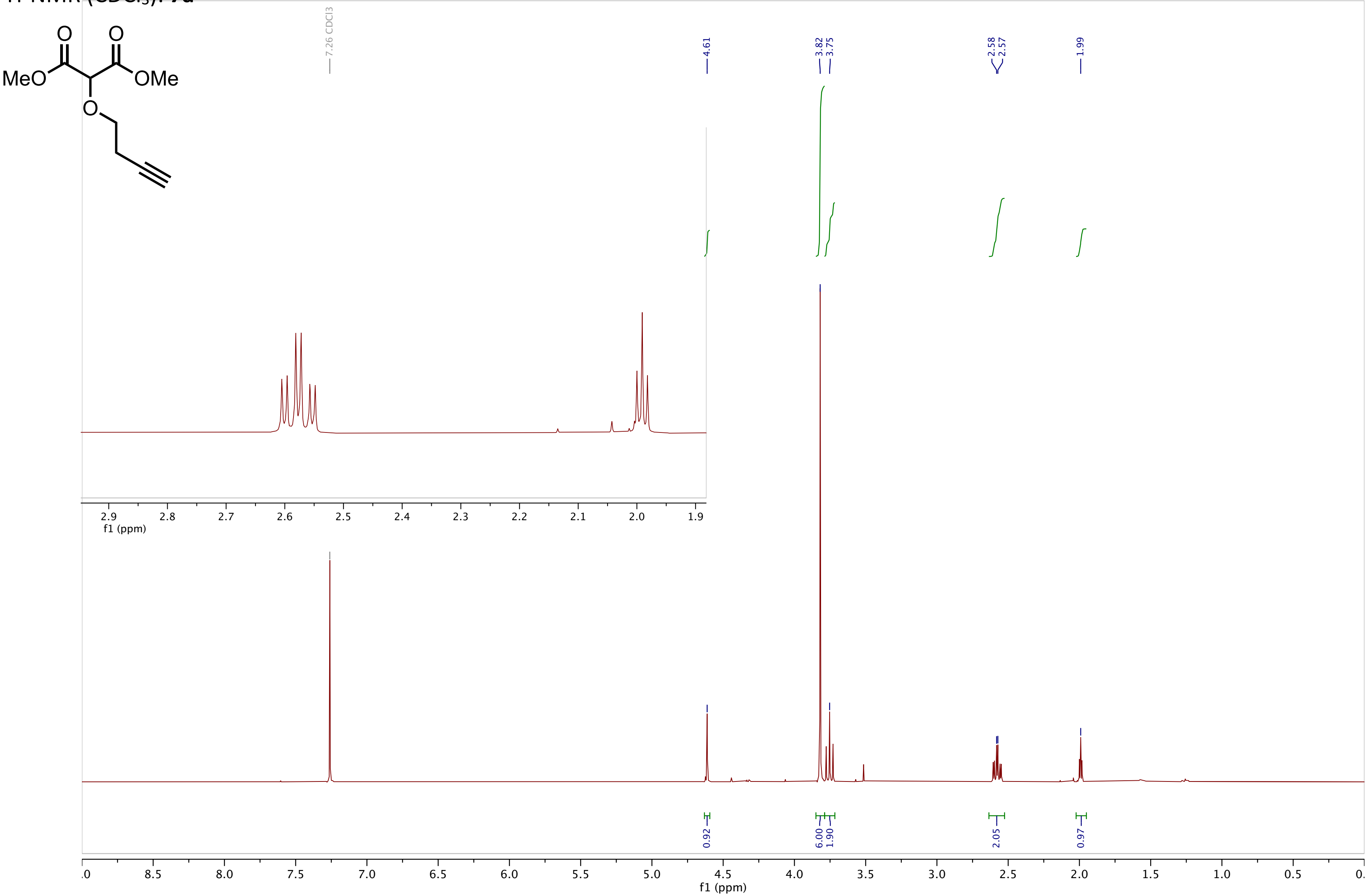

^{1}H-NMR ($CDCl_3$): **8a**

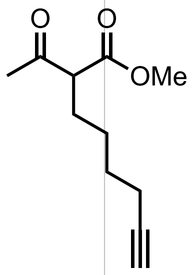

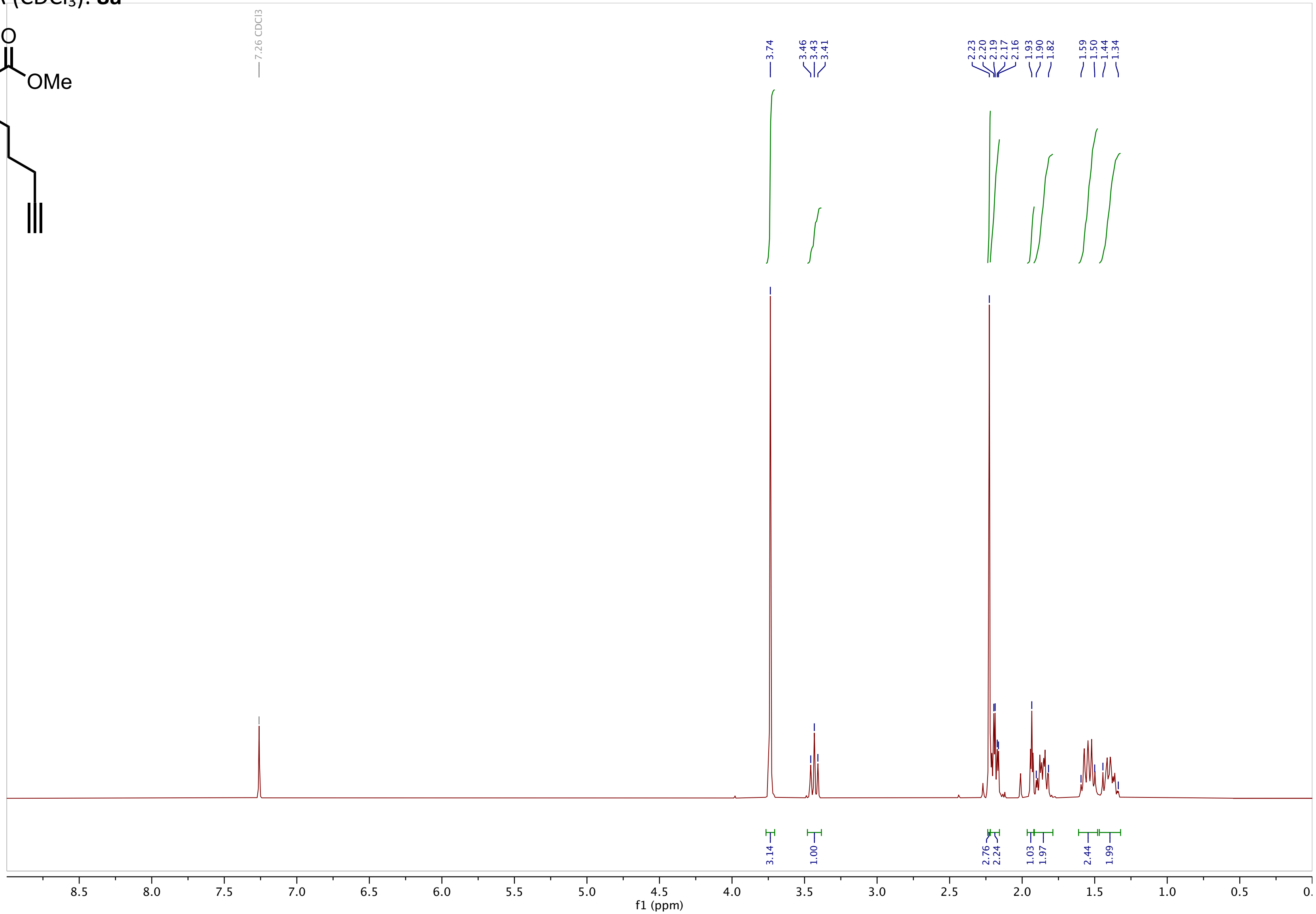

^{1}H-NMR ($CDCl_3$): **9a**

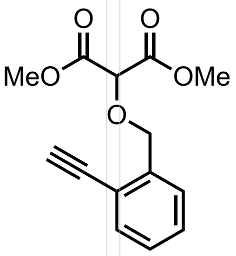

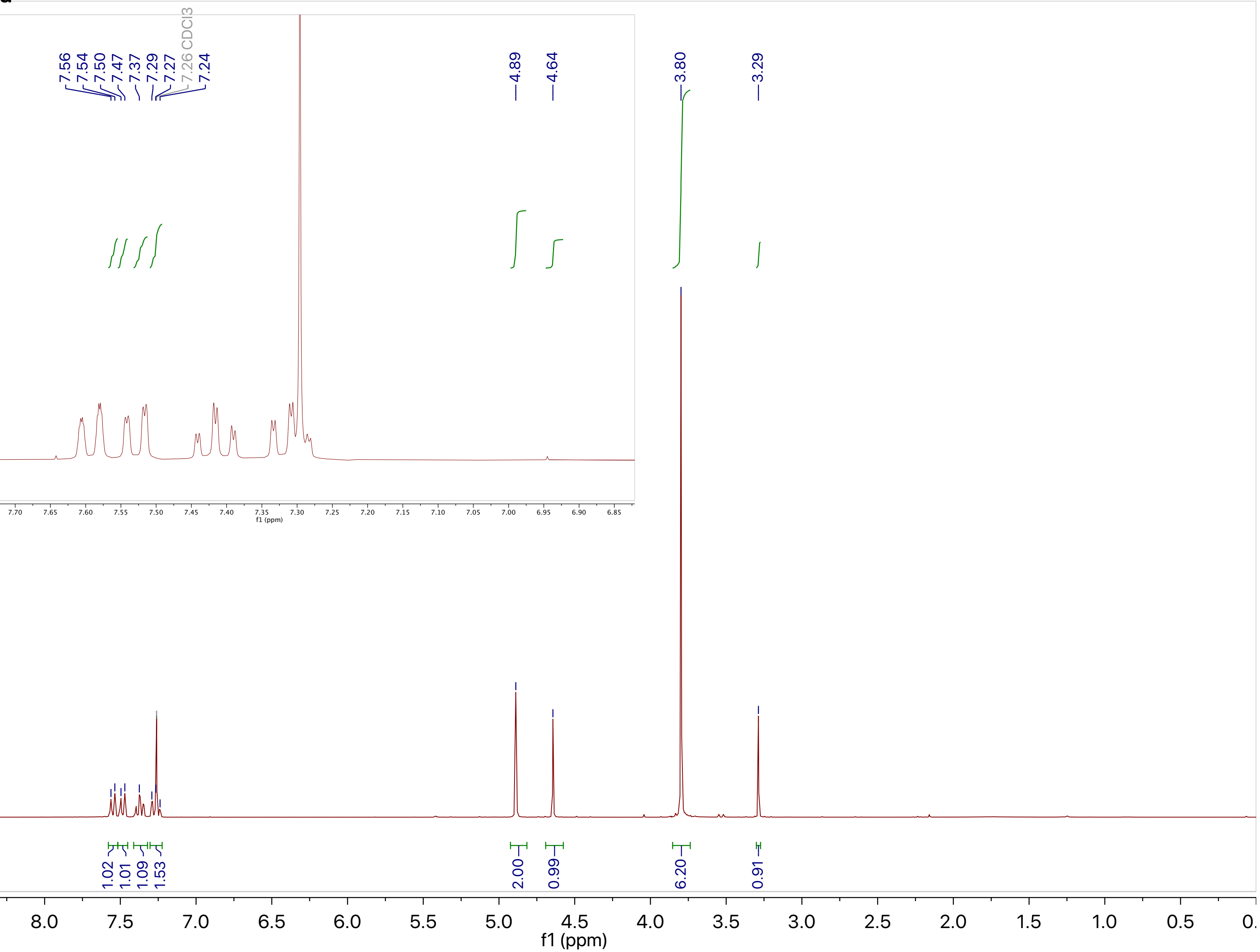

^{13}C-zgpg ($CDCl_3$): **9a**

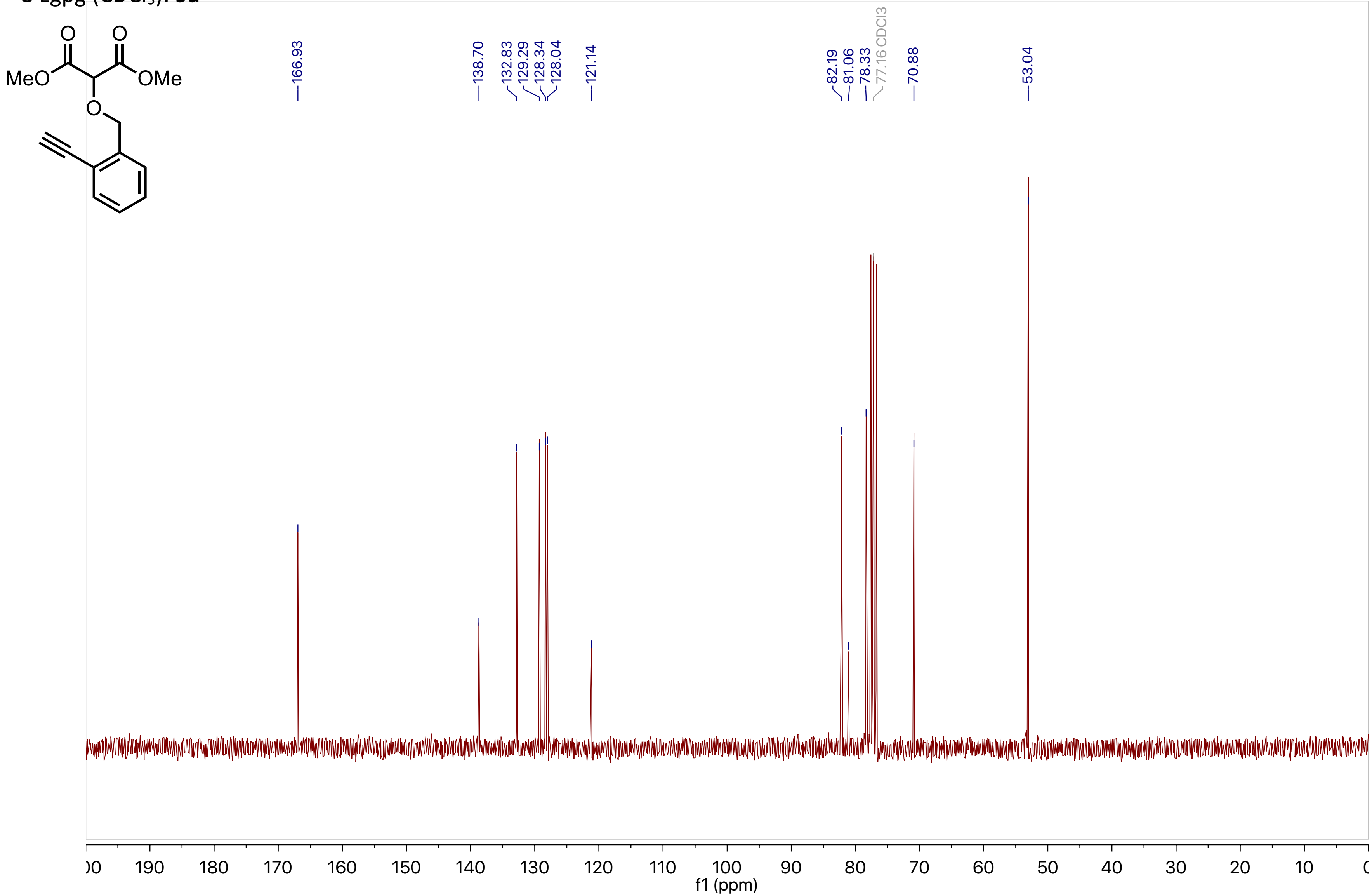

^{1}H-NMR ($CDCl_3$): **10a**

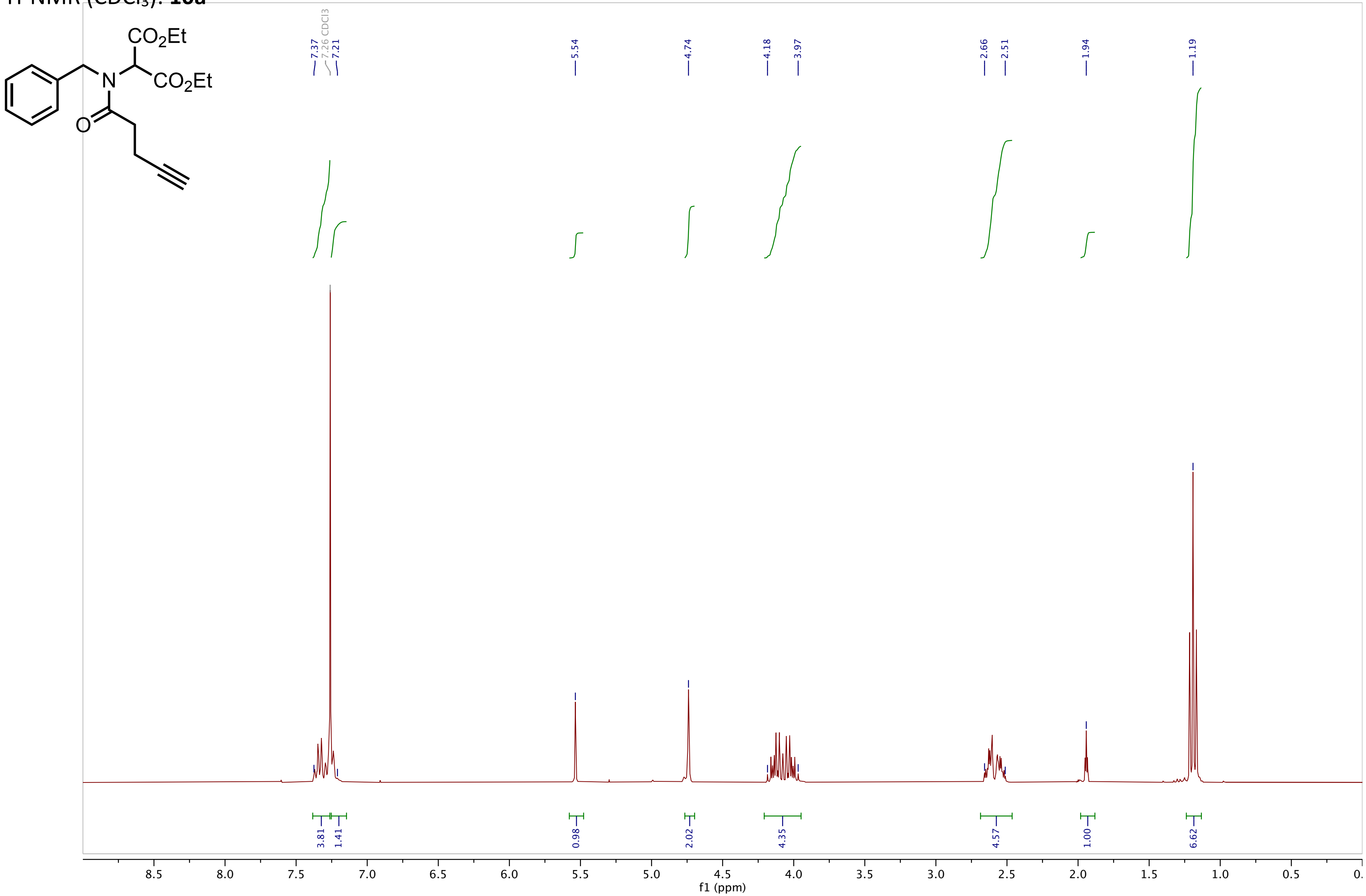

^{1}H-NMR ($CDCl_3$)

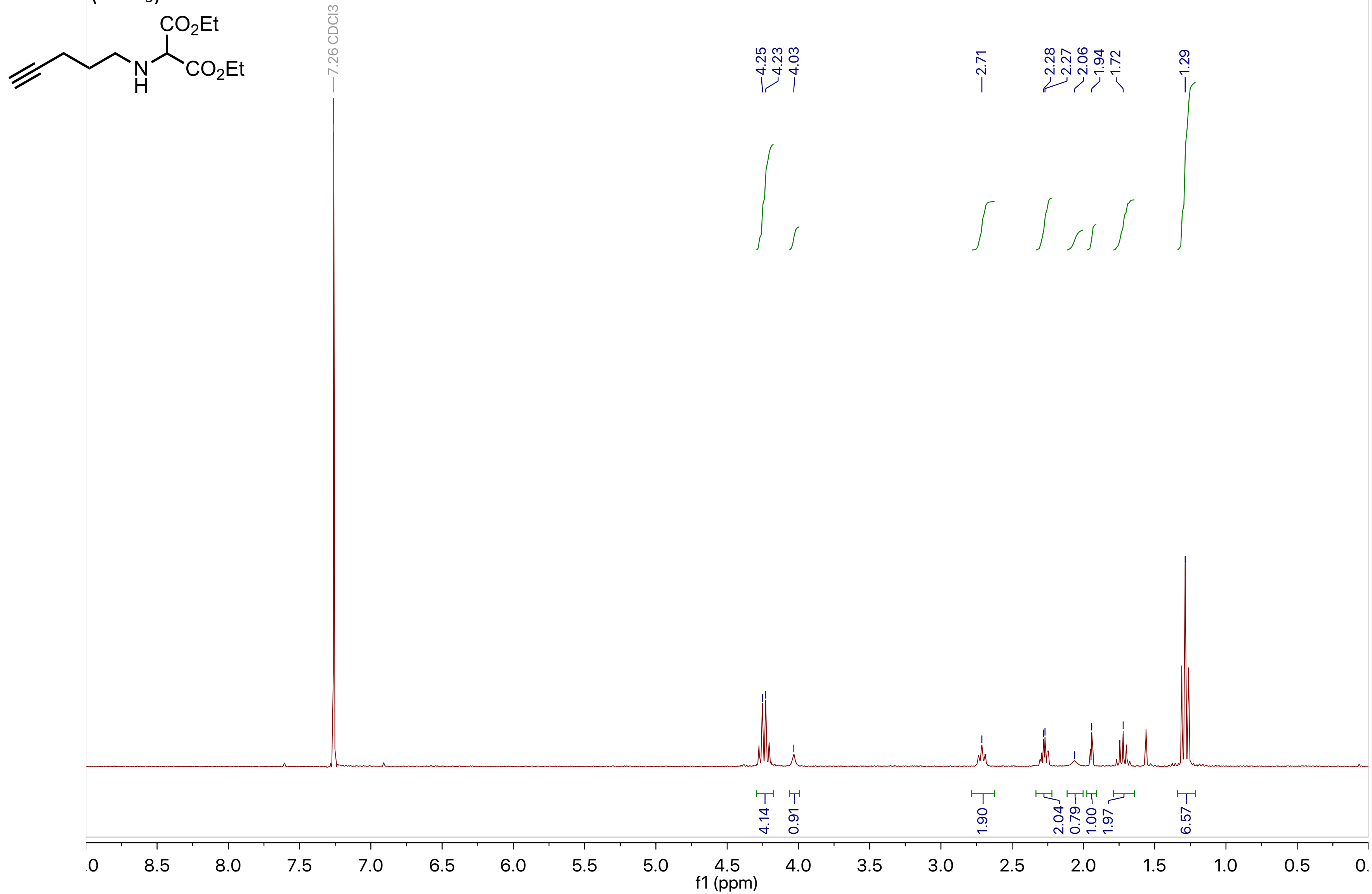

CO2Et
N
H
CO2Et
7.26 CDCl3
4.25
4.23
4.03
2.71
2.28
2.27
2.06
1.94
1.72
1.29
4.14
0.91
1.90
2.04
0.79
1.00
1.97
6.57
8.5
8.0
7.5
7.0
6.5
6.0
5.5
5.0
4.5
4.0
3.5
3.0
2.5
2.0
1.5
1.0
0.5
f1 (ppm)

^{13}C-zgpg ($CDCl_3$)

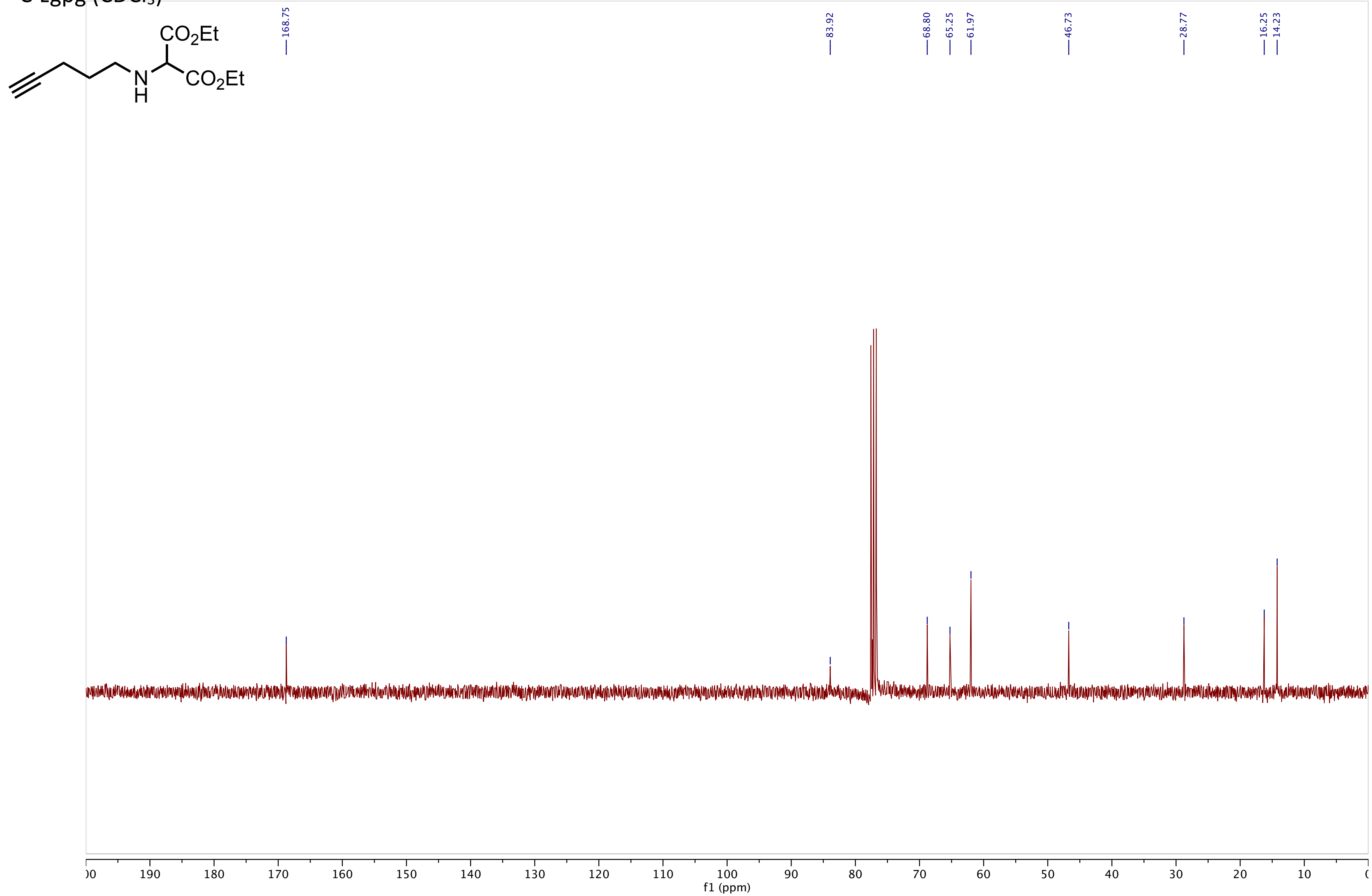

^{1}H-NMR: Room Temperature (CD_3OD): **11a**

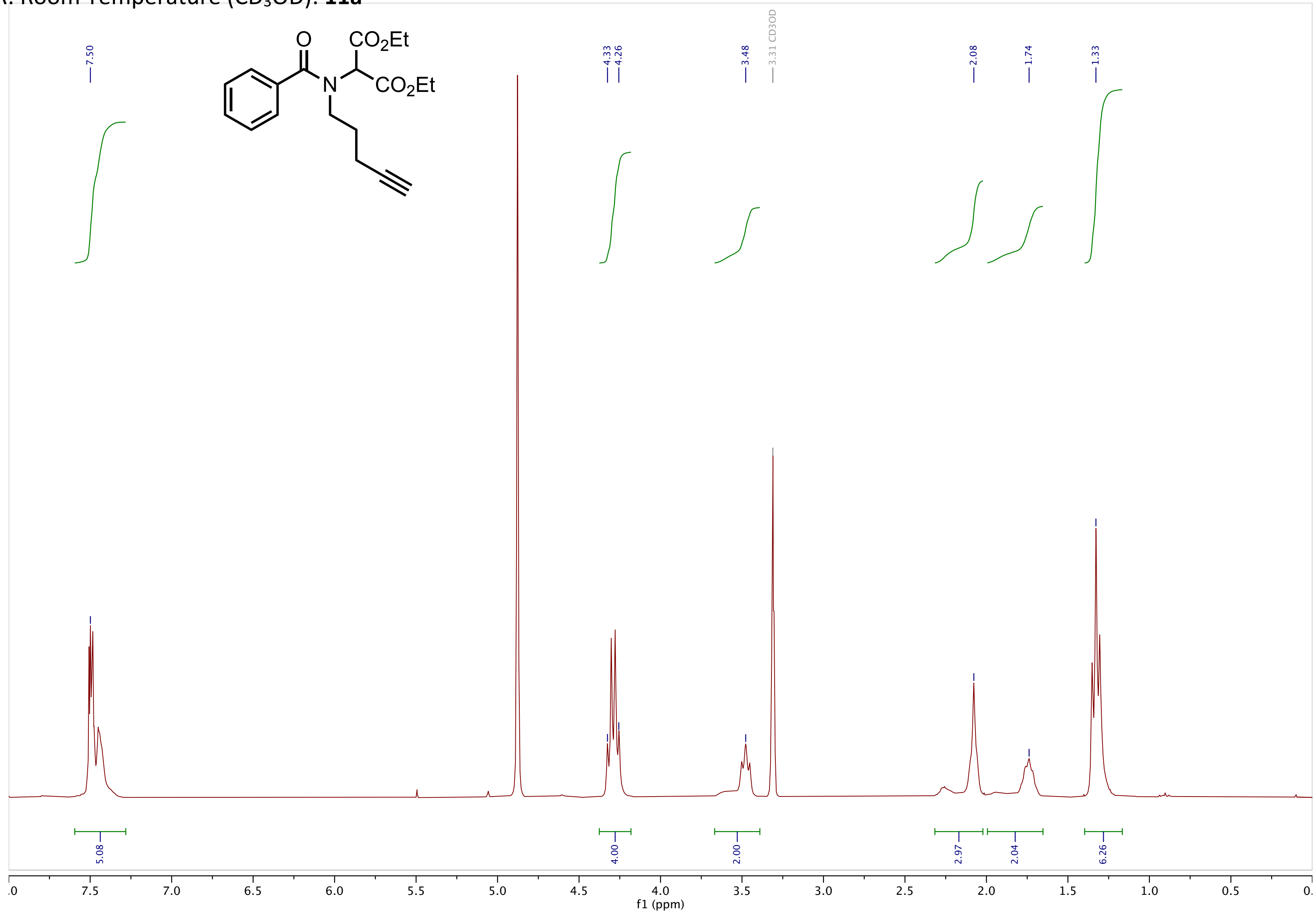

^{1}H-NMR: -60 °C (CD_3OD): **11a**

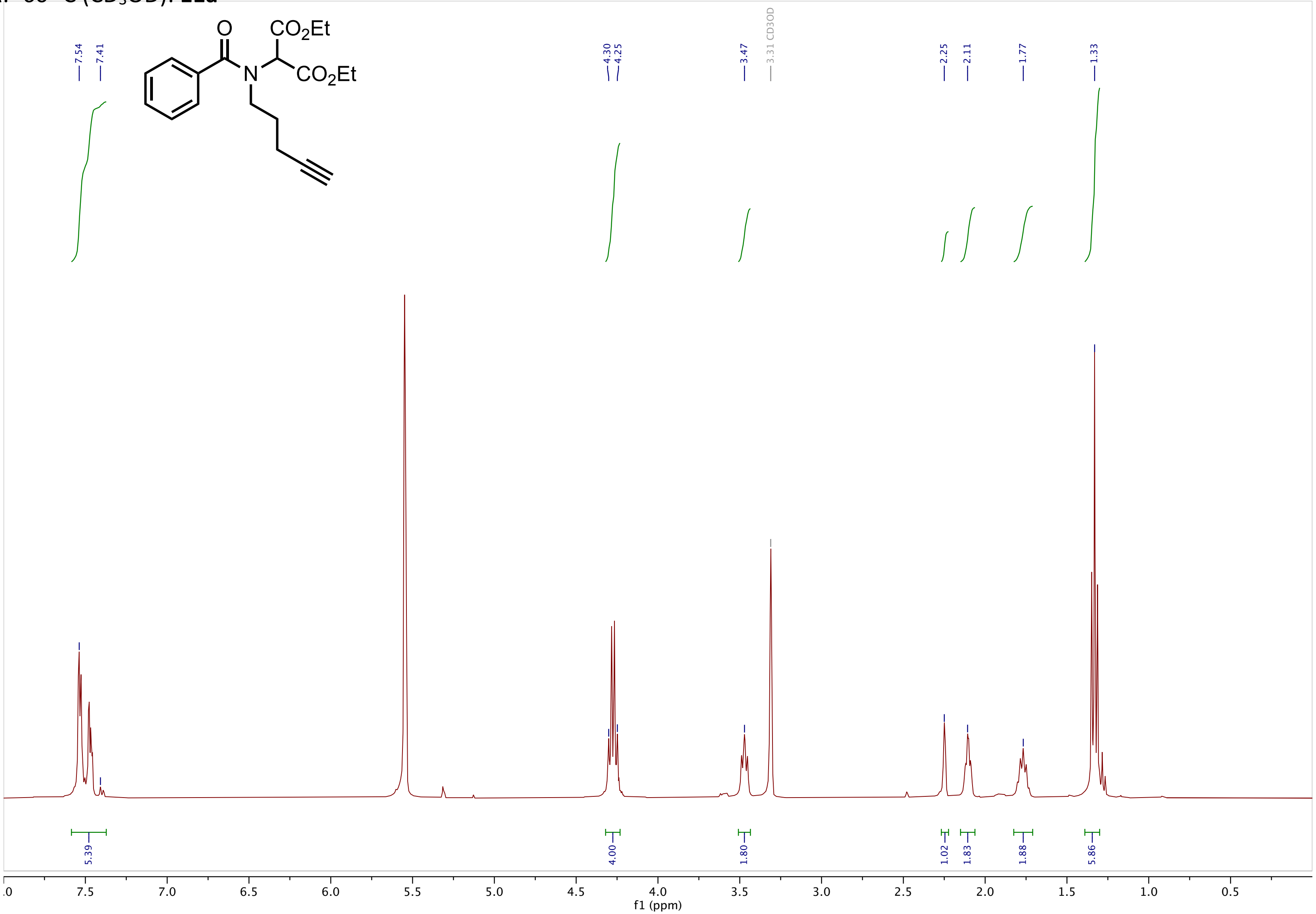

^{13}C-zgpg ($CDCl_3$): **11a**

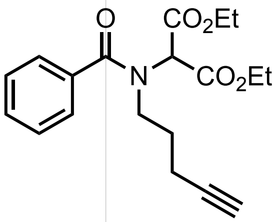

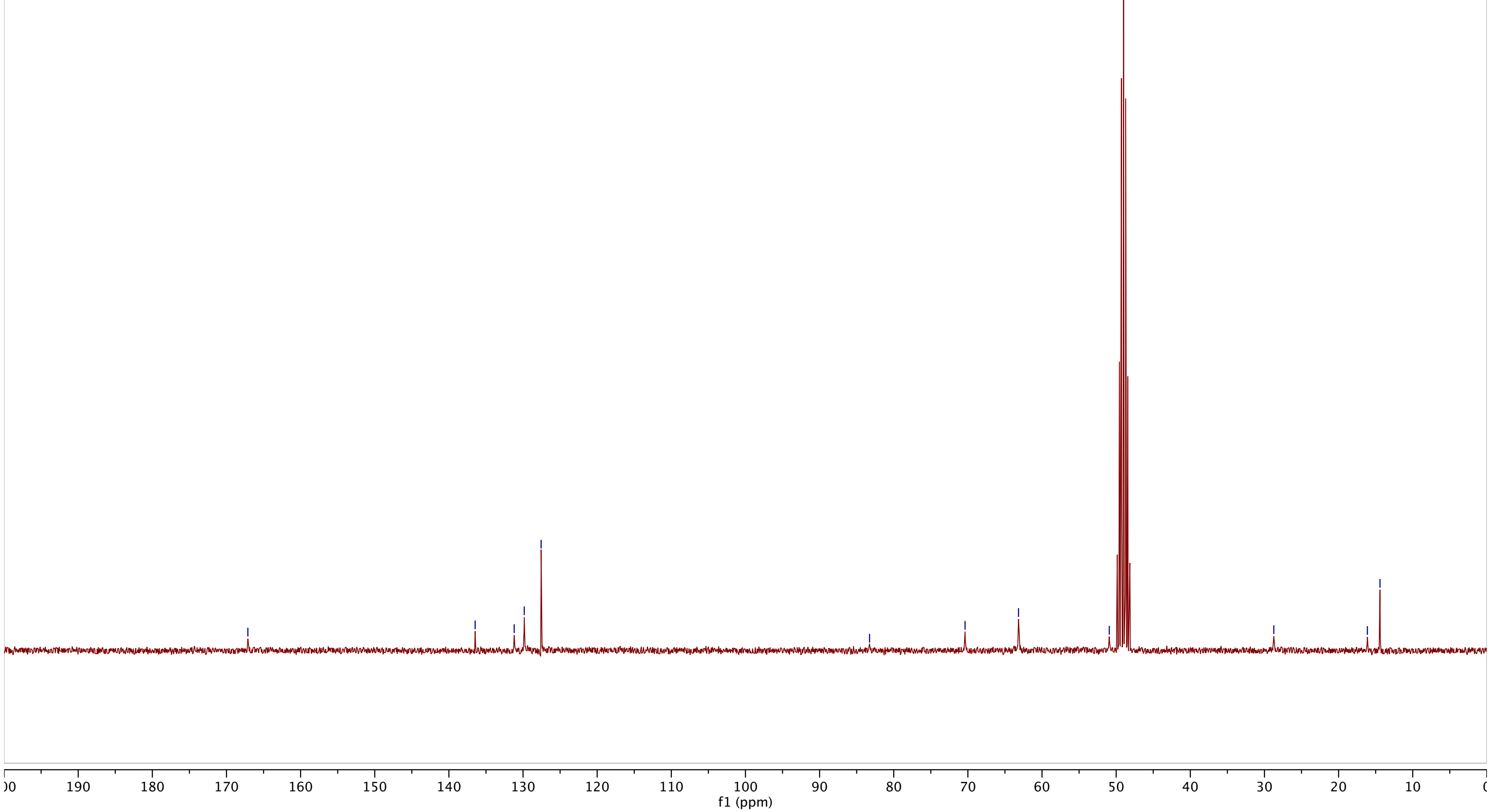

^{1}H-NMR ($CDCl_3$): **12a**

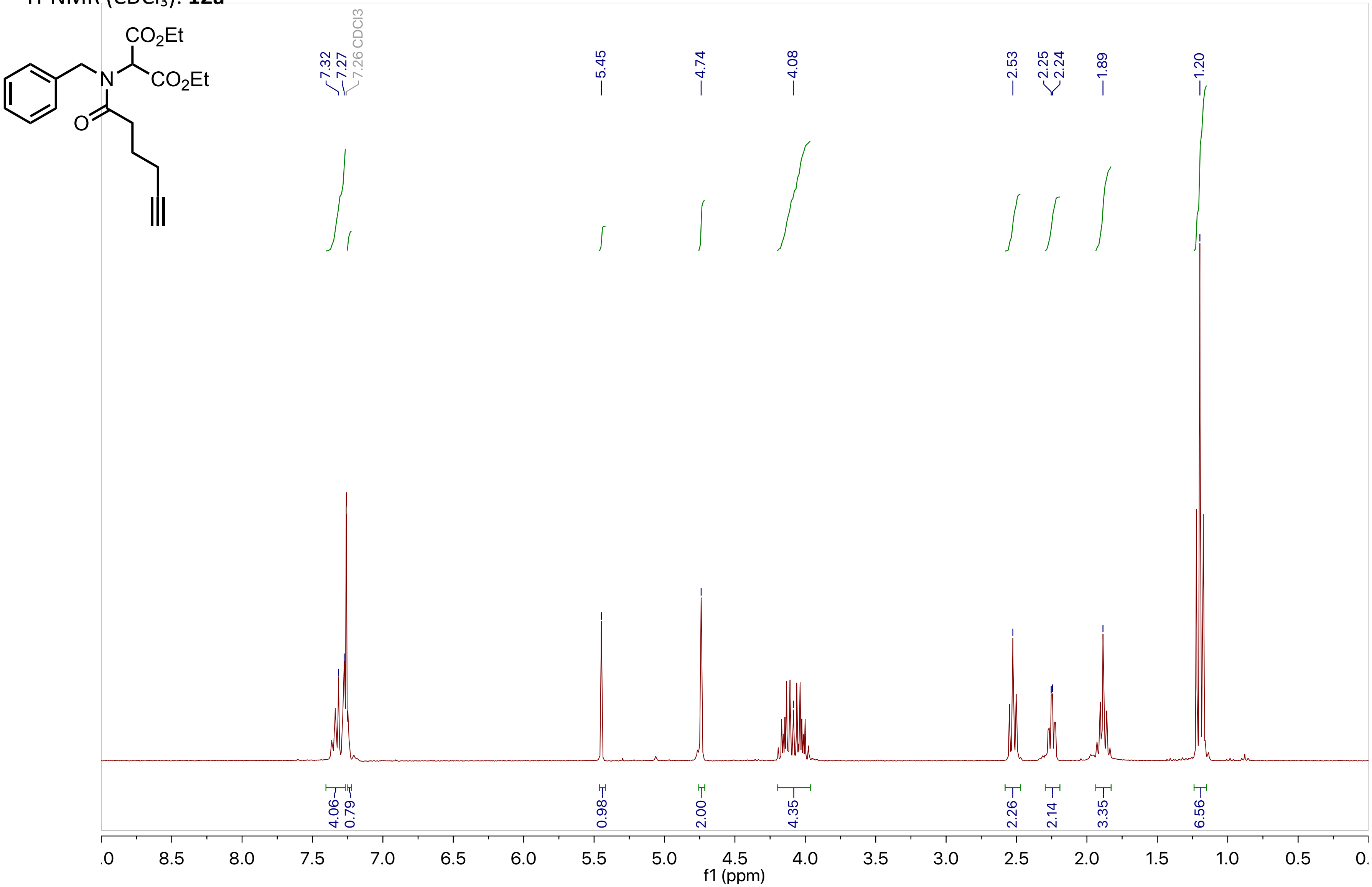

[13]C-zgpg ($CDCl_3$): **12a**

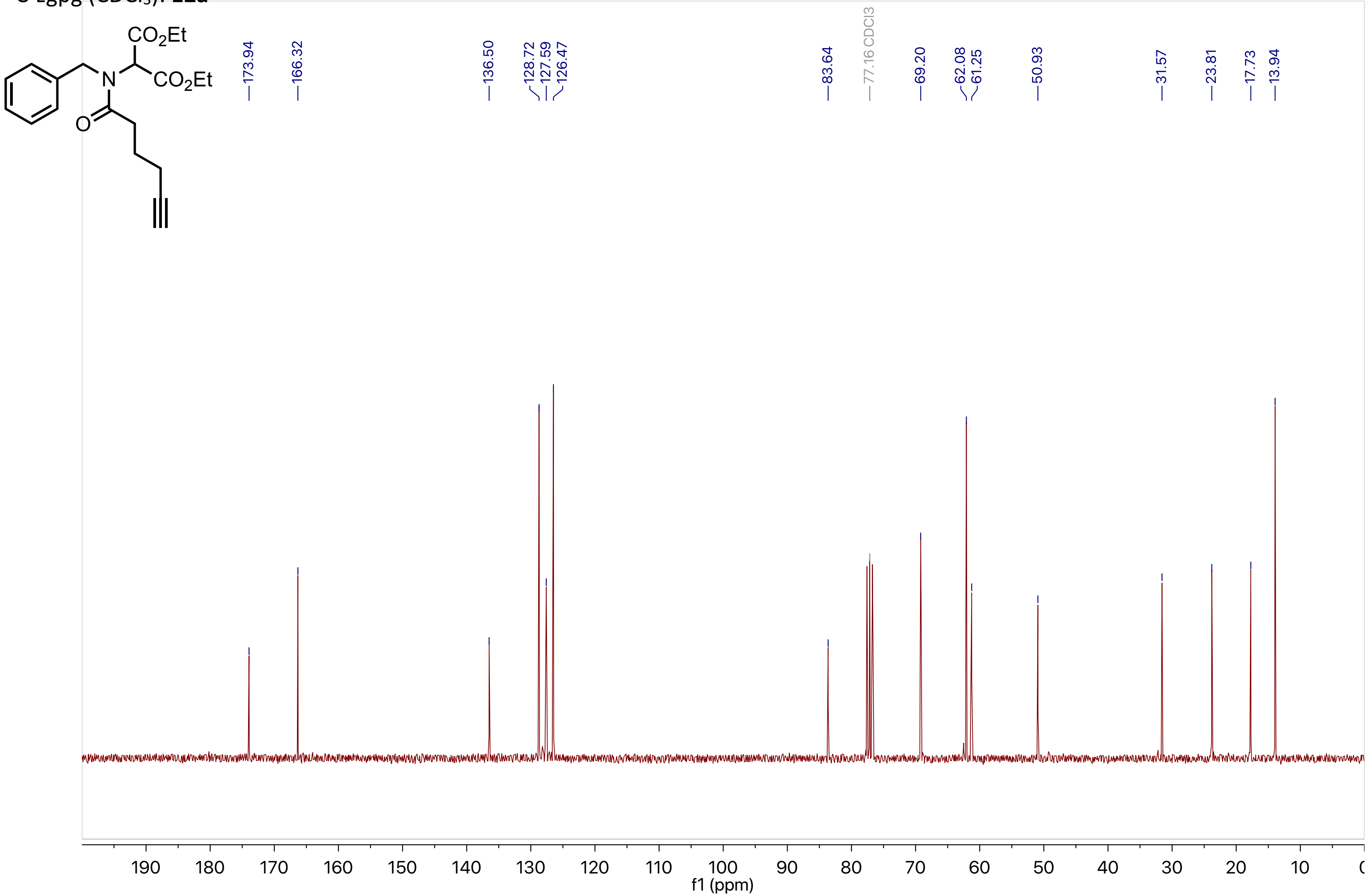

^{1}H-NMR ($CDCl_3$): **13a**

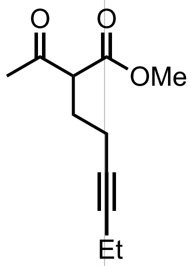

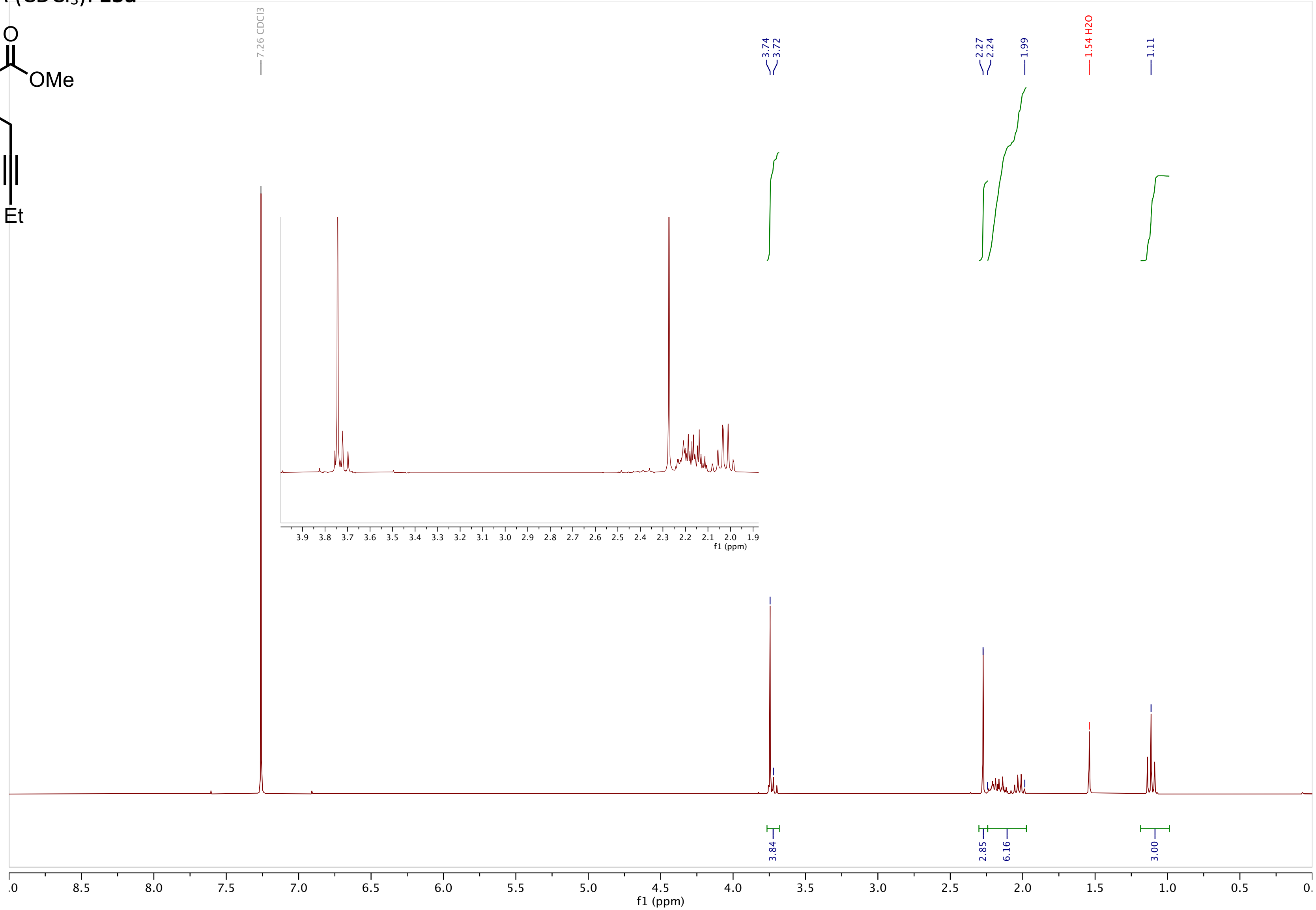

^{1}H-NMR ($CDCl_3$): **14a**

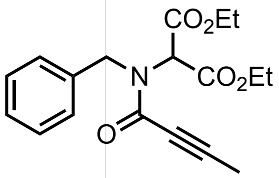

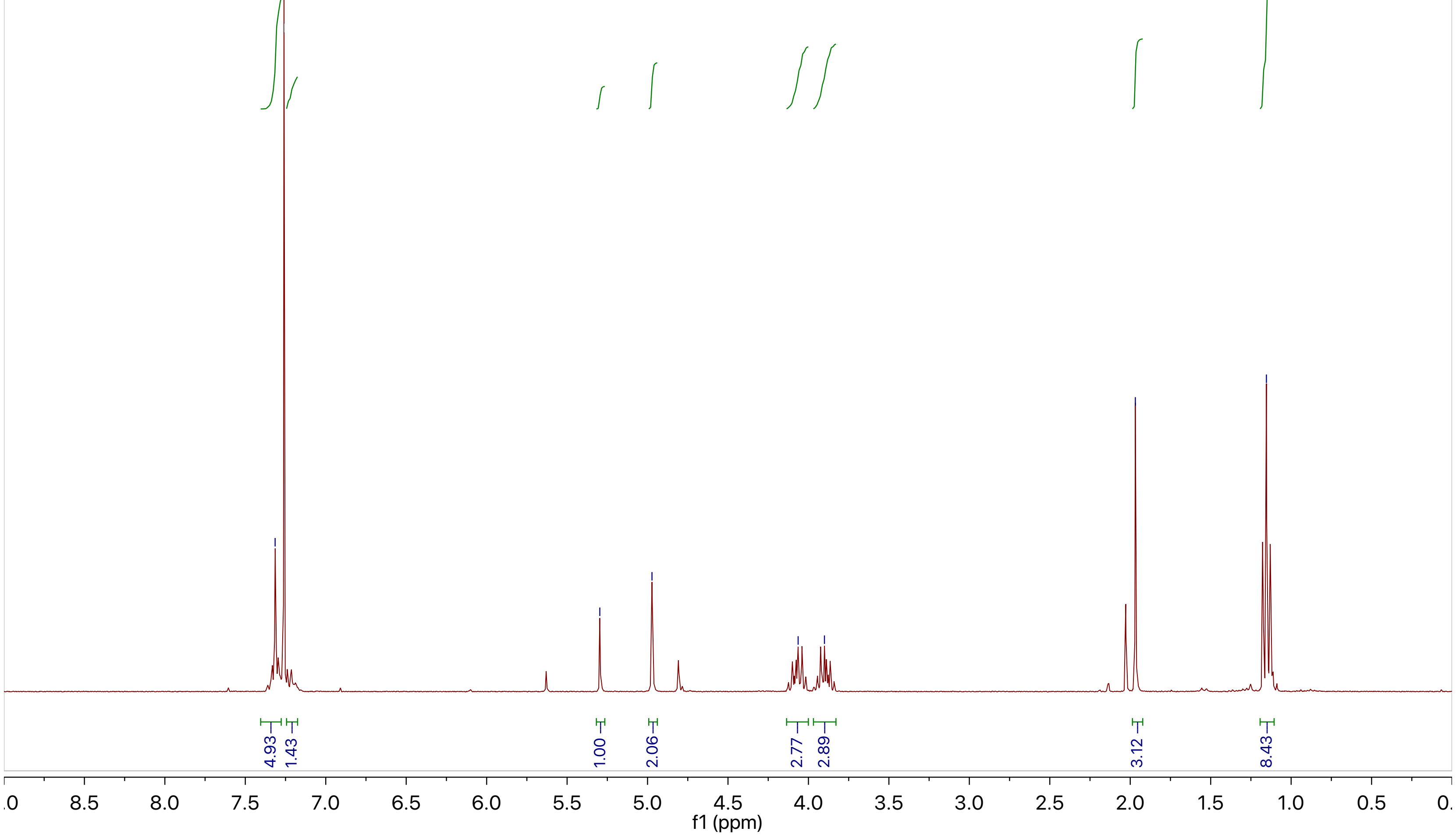

^{13}C-zgpg ($CDCl_3$): **14a**

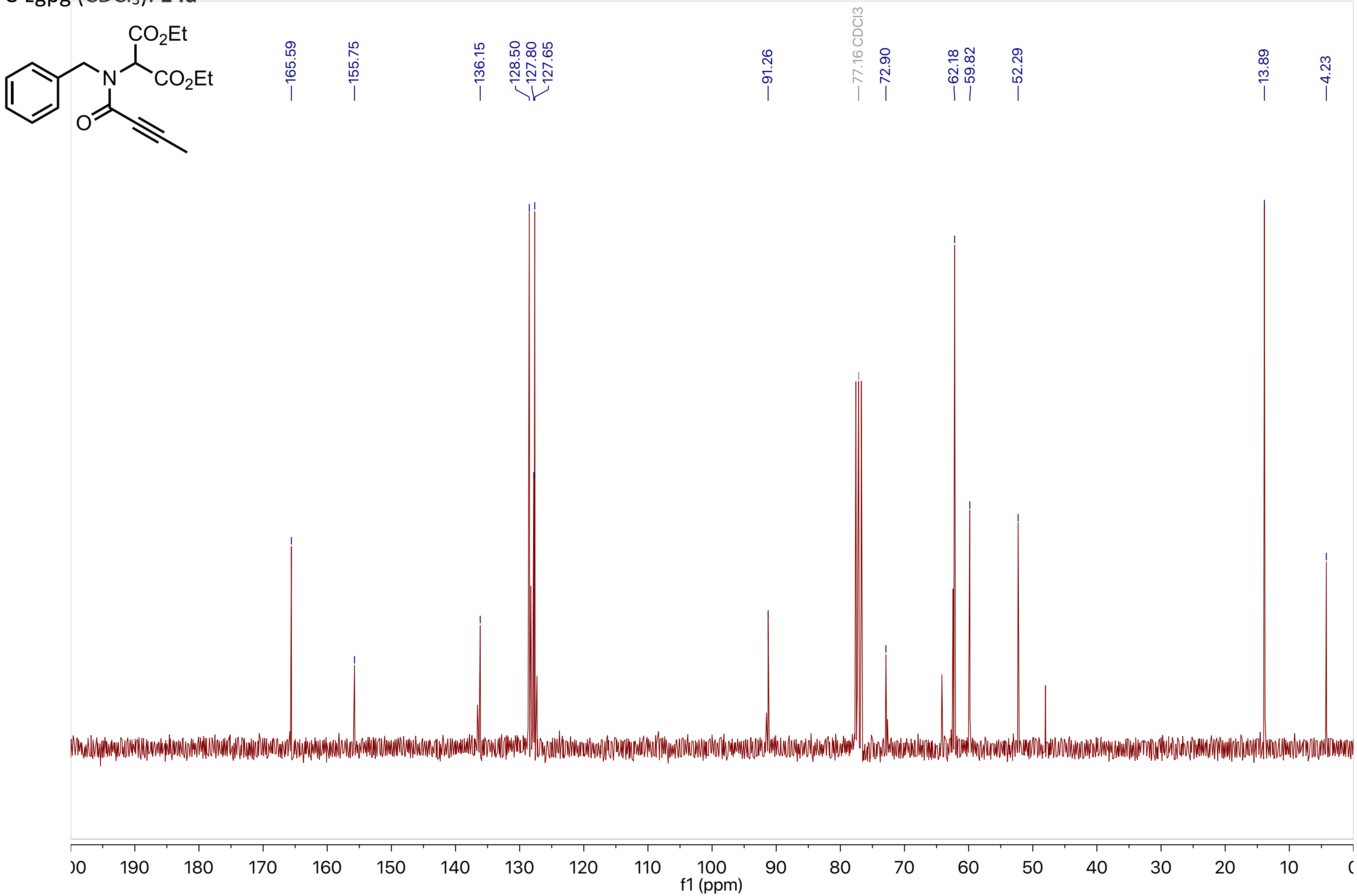

^{1}H-NOESY ($CDCl_3$): **14a**

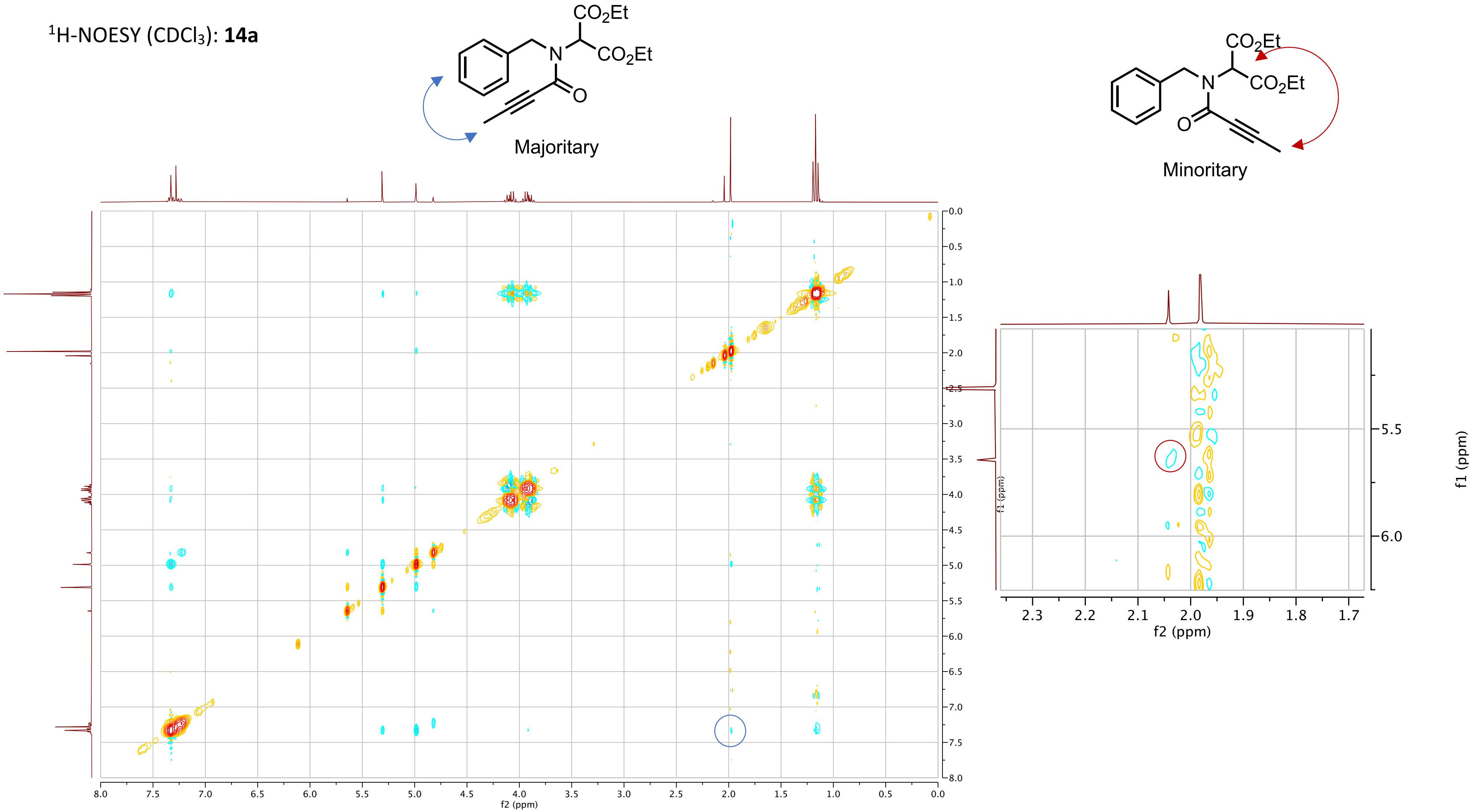

^1H-NMR ($CDCl_3$): **15a**

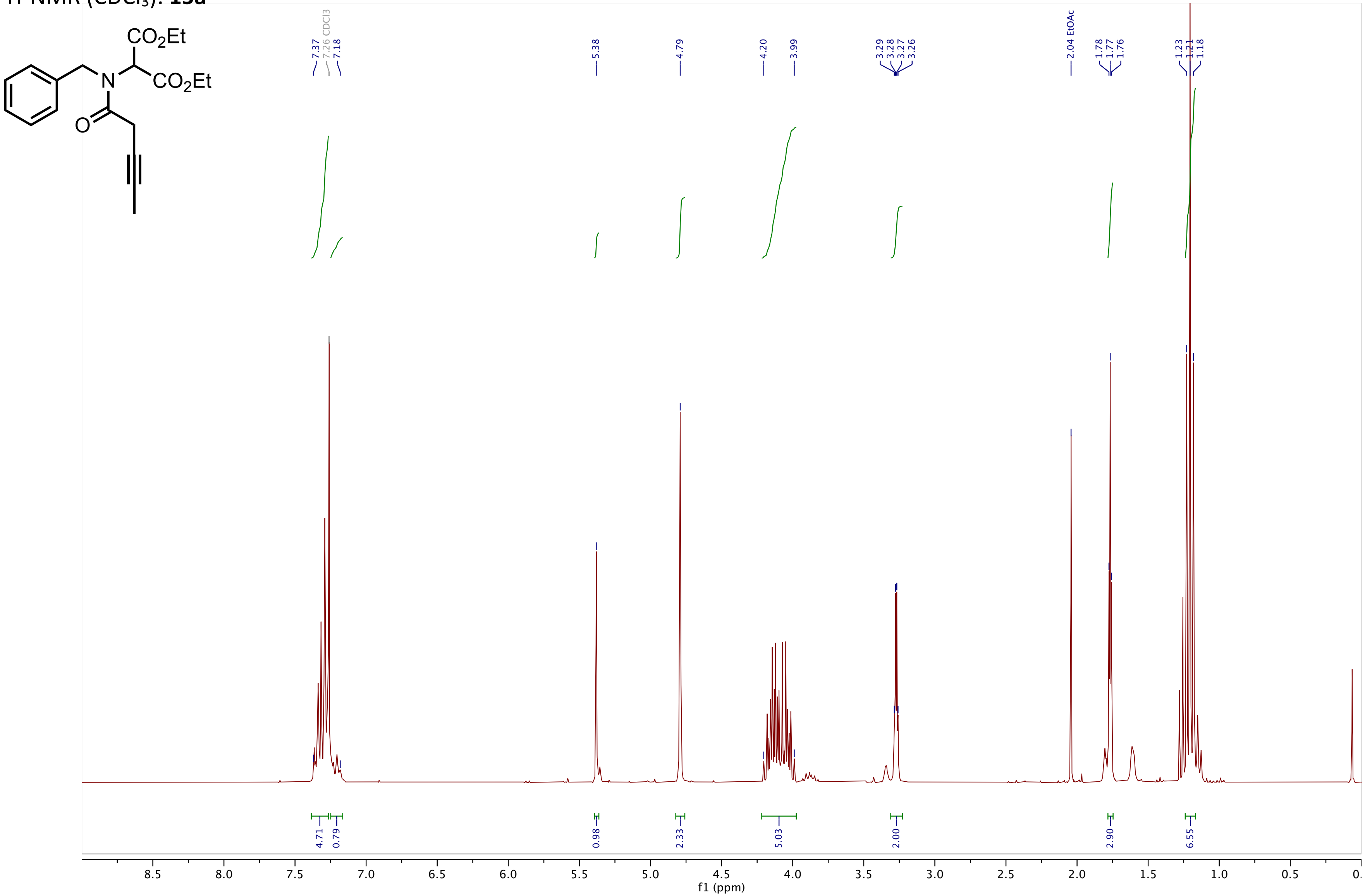

^{1}H-NMR ($CDCl_3$): **16a**

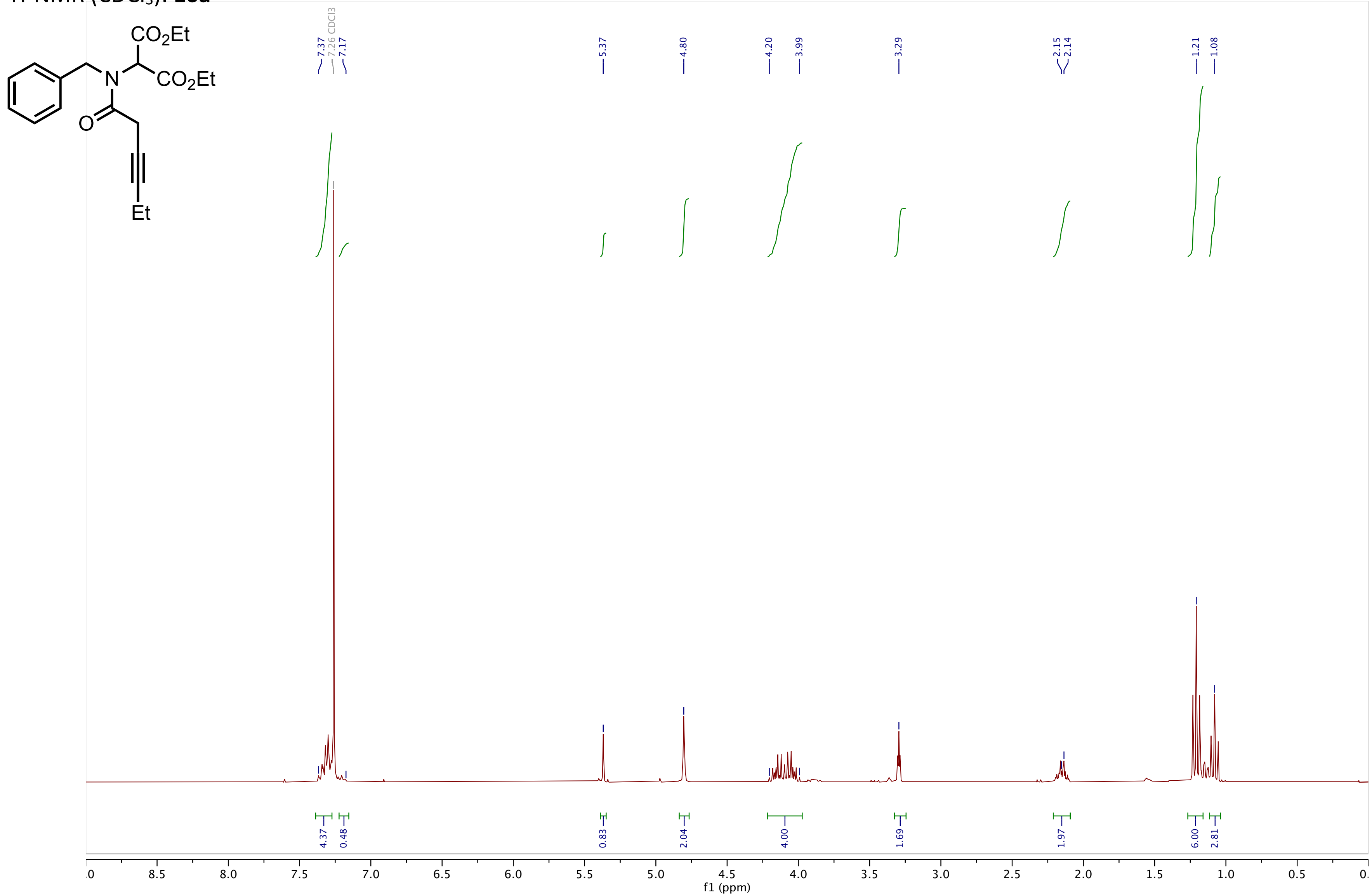

^{1}H-NMR ($CDCl_3$)

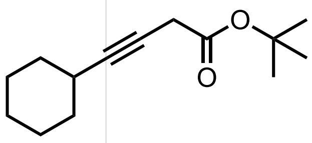

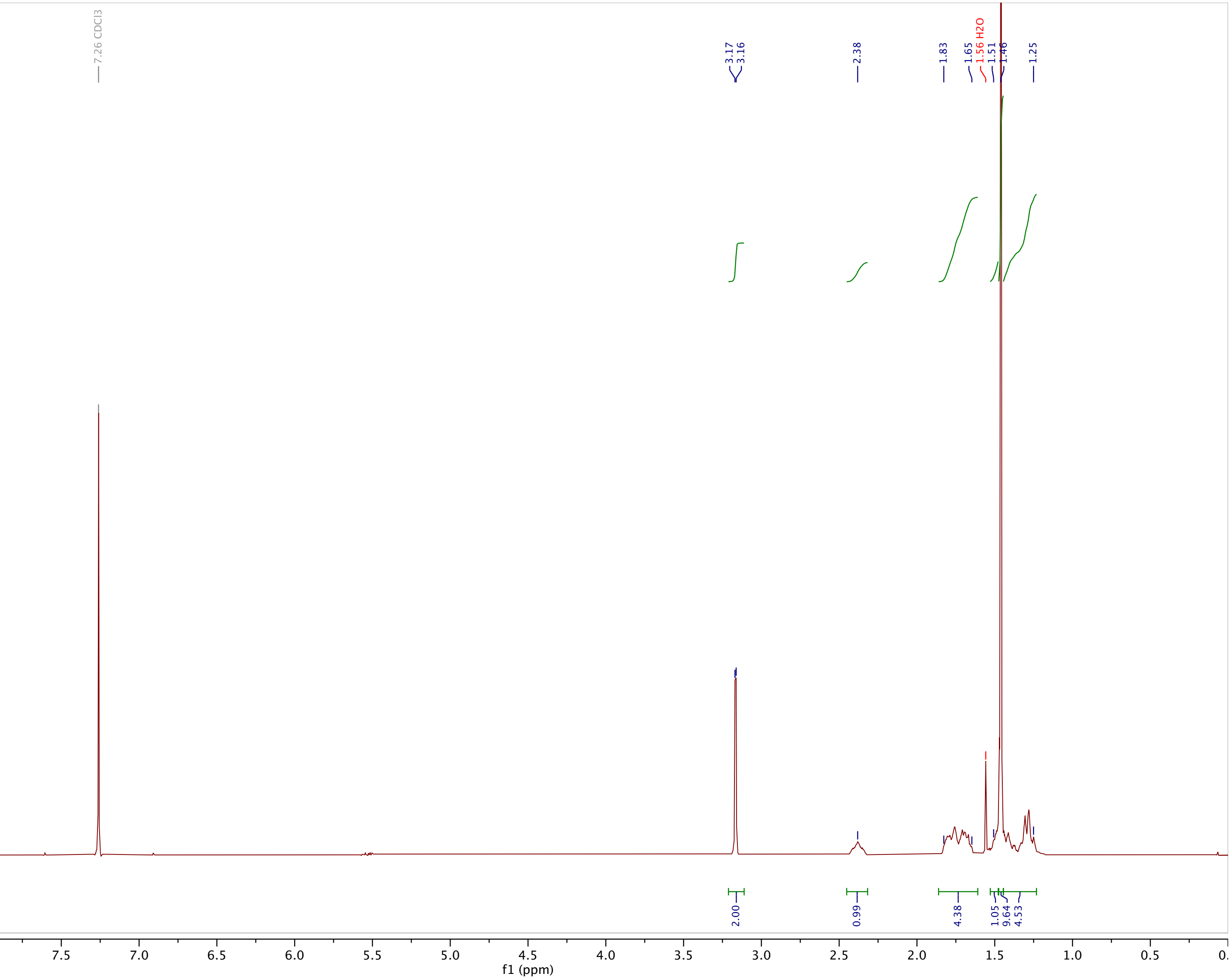
7.26 CDCl3
3.17
3.16
2.38
1.83
1.65
1.56 H2O
1.51
1.46
1.25
2.00
0.99
4.38
1.05
9.64
4.53
f1 (ppm)

^{13}C-zgpg ($CDCl_3$)

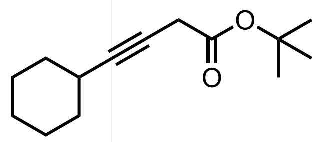

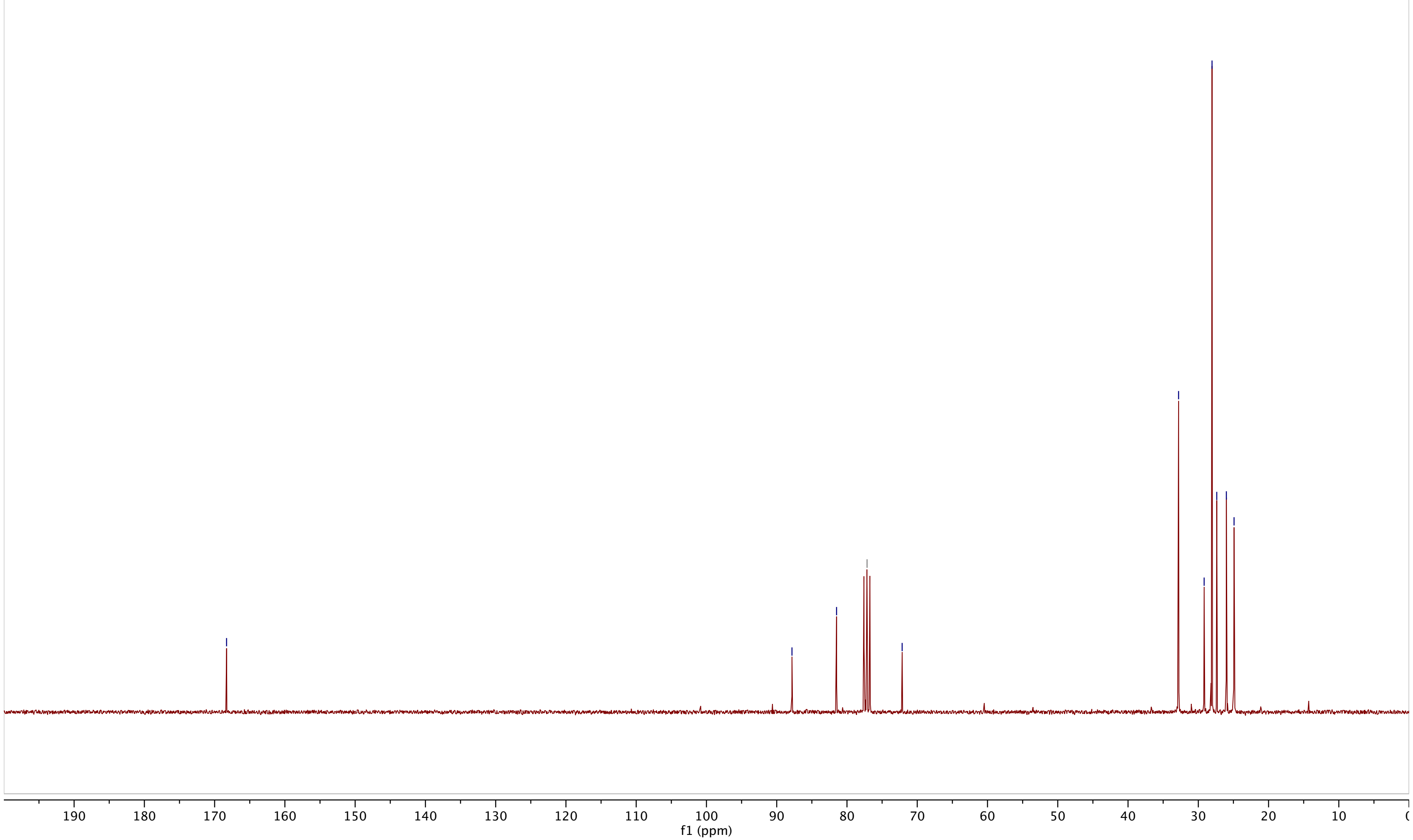

^{1}H-NMR ($CDCl_3$): **17a**

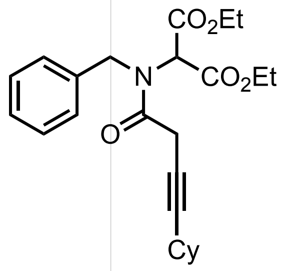

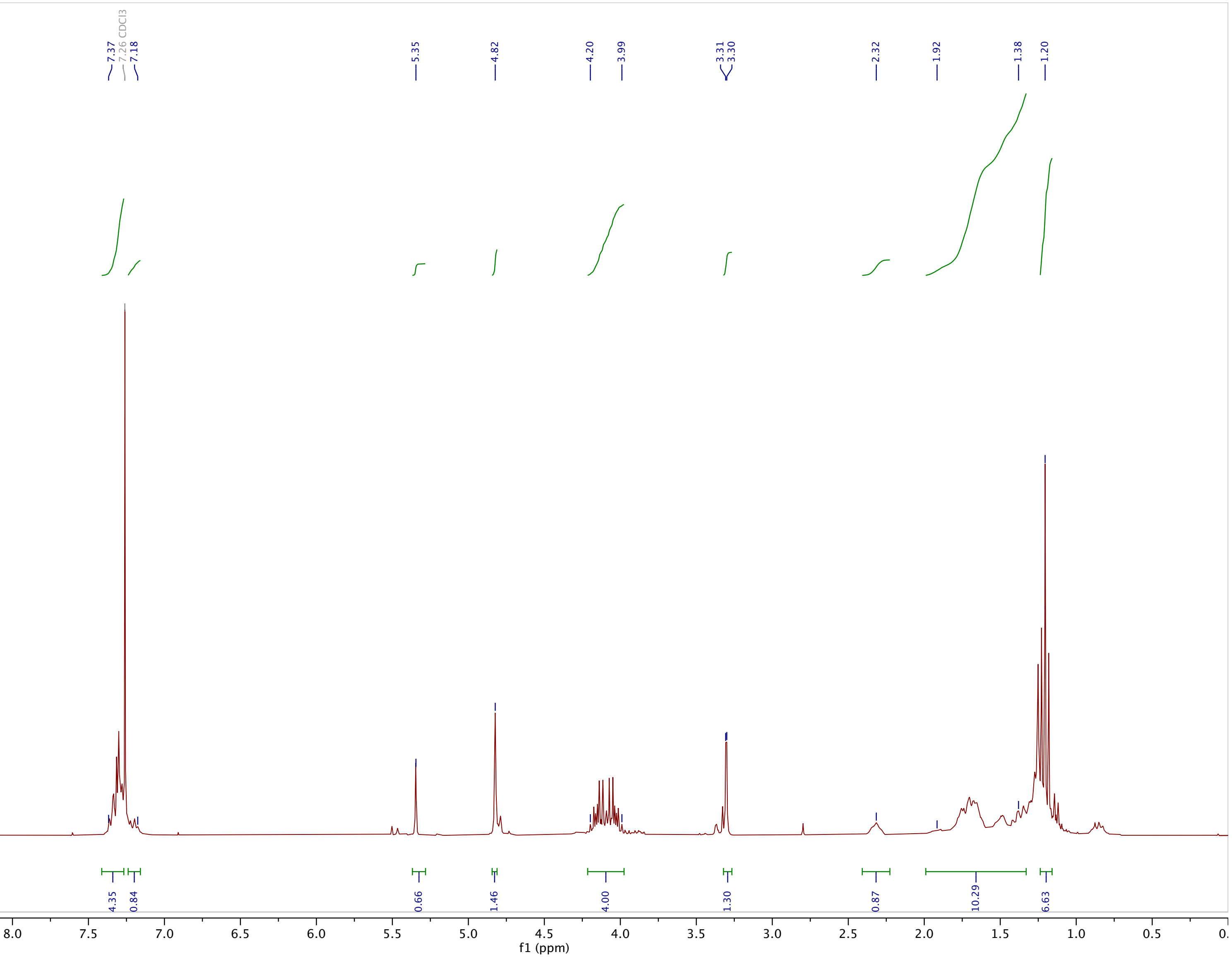

^{13}C-zgpg ($CDCl_3$): **17a**

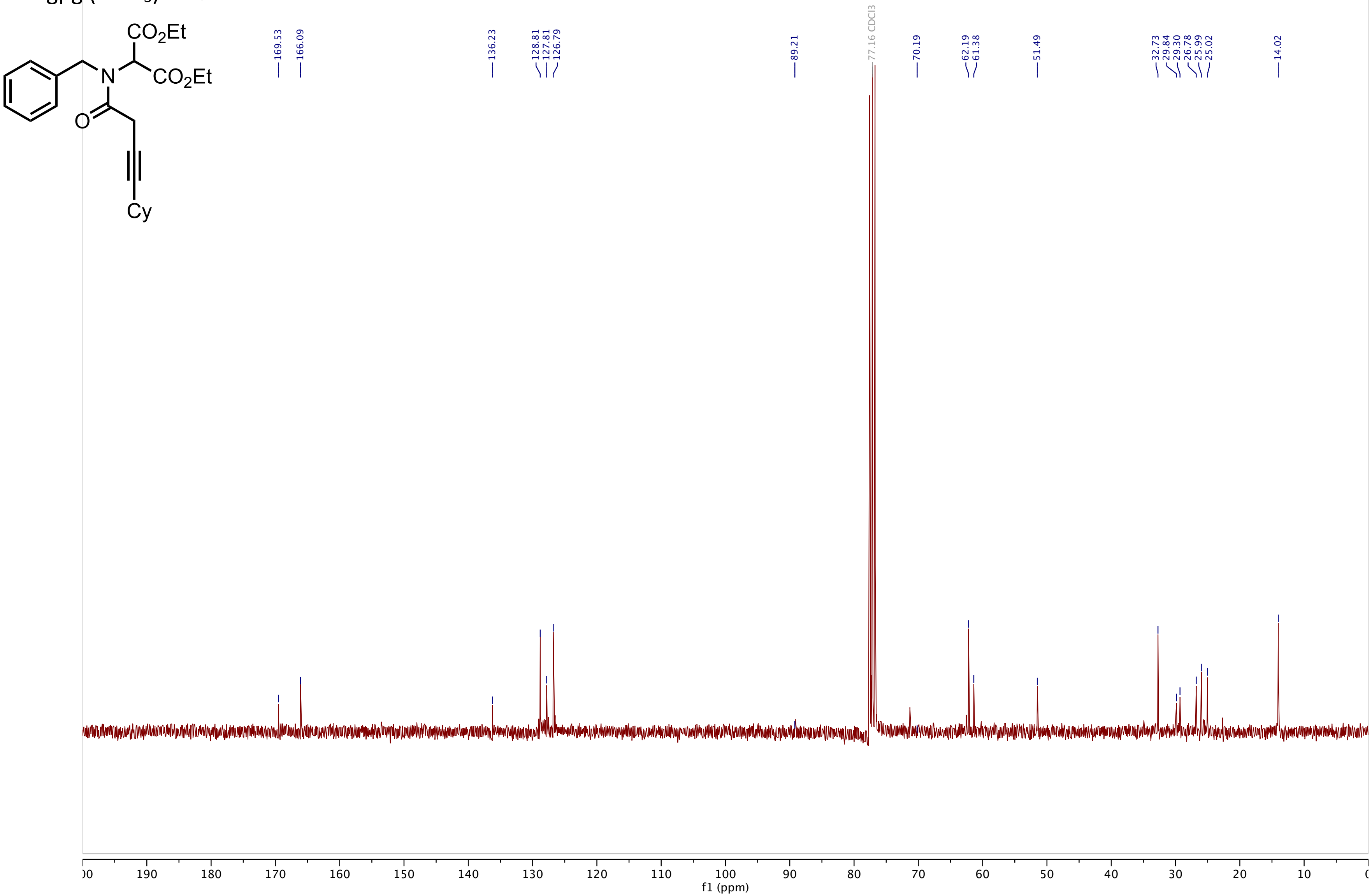

^{13}C-HSQC ($CDCl_3$): **17a**

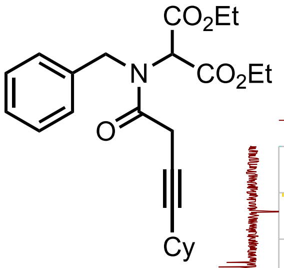

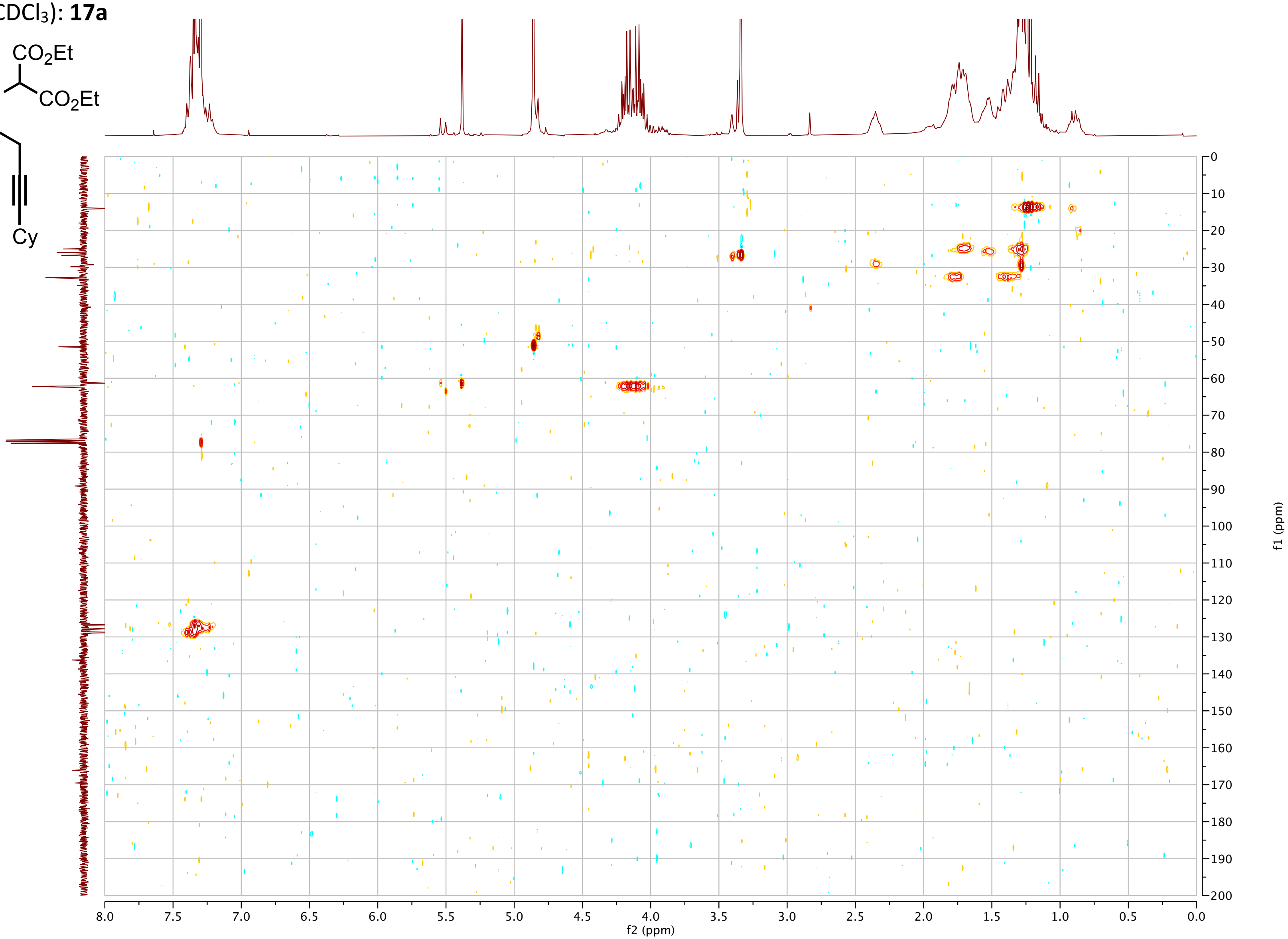

^{13}C-HMBC ($CDCl_3$): **17a**

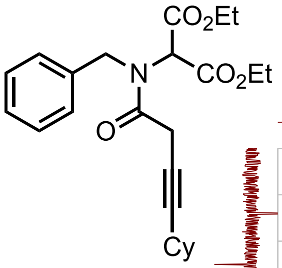

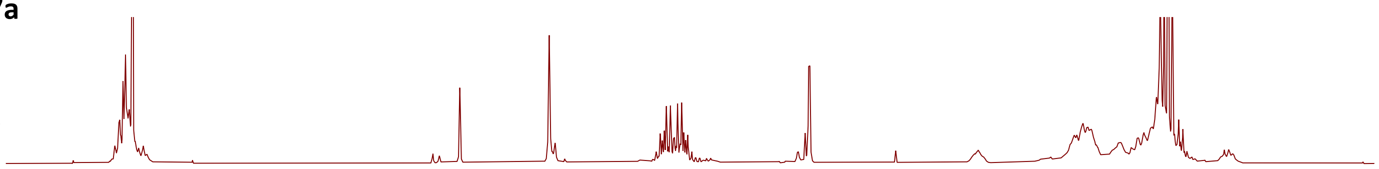

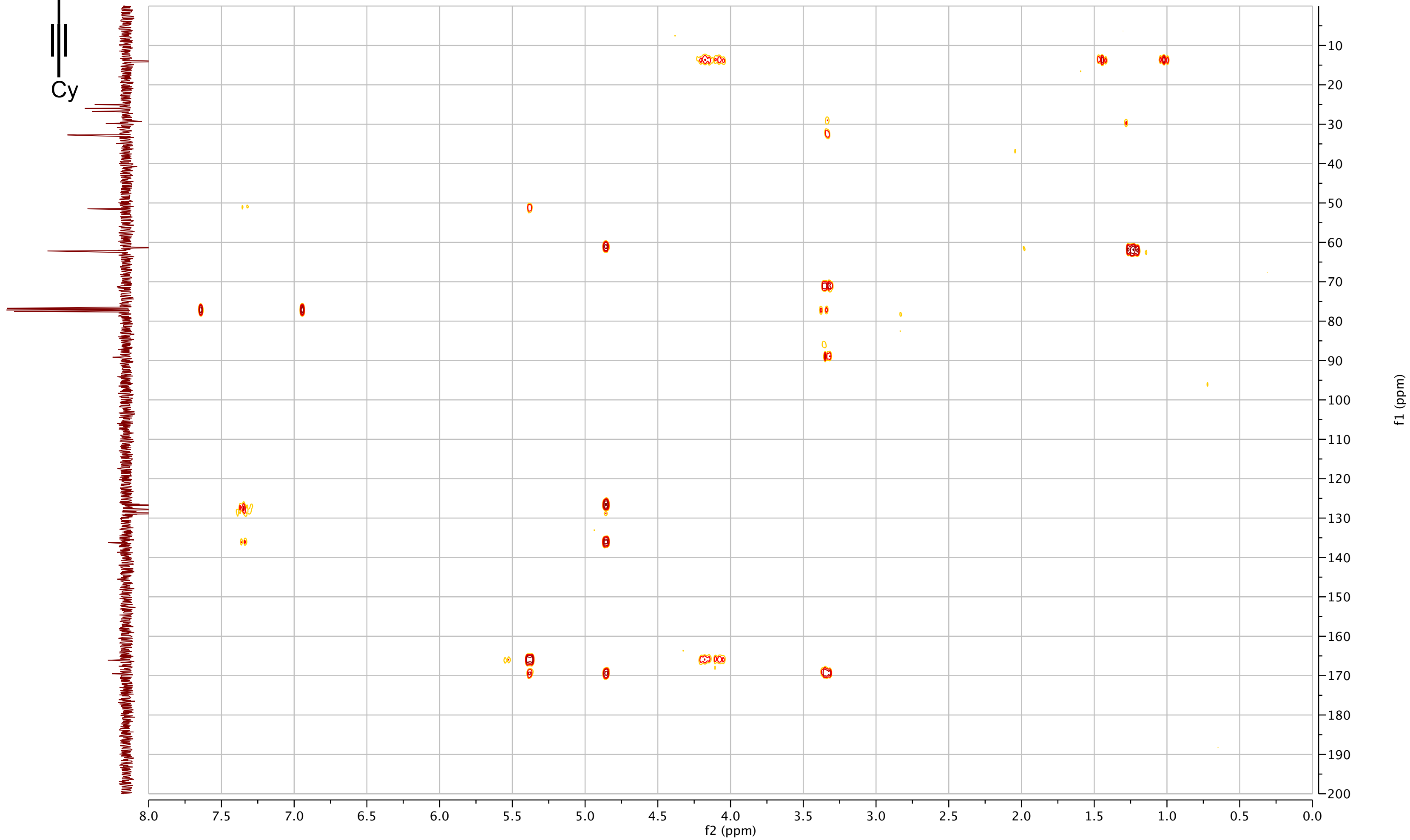

^{1}H-NMR ($CDCl_3$)

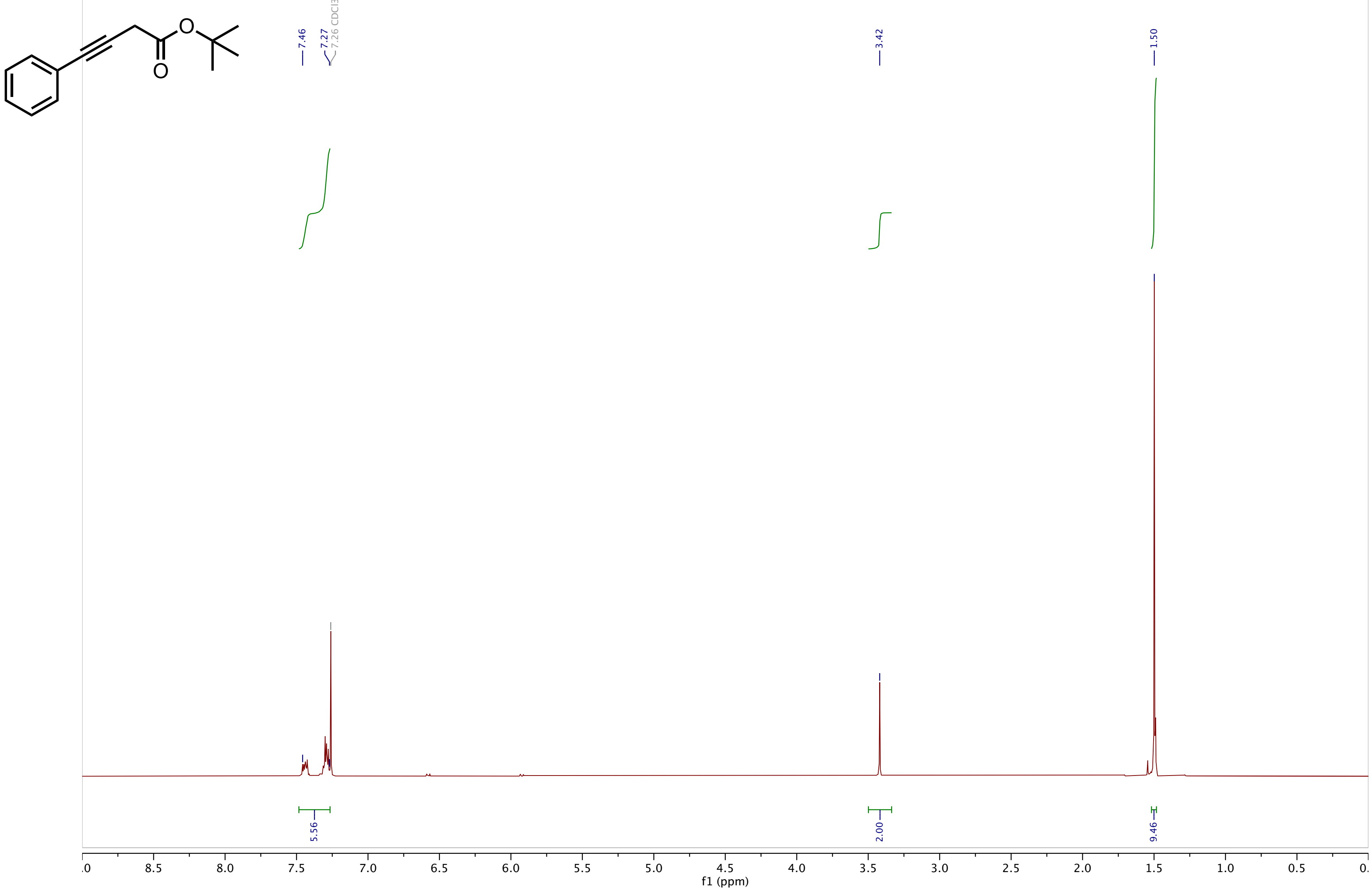

7.46
7.27
7.26 CDCl3
3.42
1.50
5.56
2.00
9.46
.0
8.5
8.0
7.5
7.0
6.5
6.0
5.5
5.0
4.5
4.0
3.5
3.0
2.5
2.0
1.5
1.0
0.5
0.
f1 (ppm)
O
O

^{1}H-NMR ($CDCl_3$): **18a**

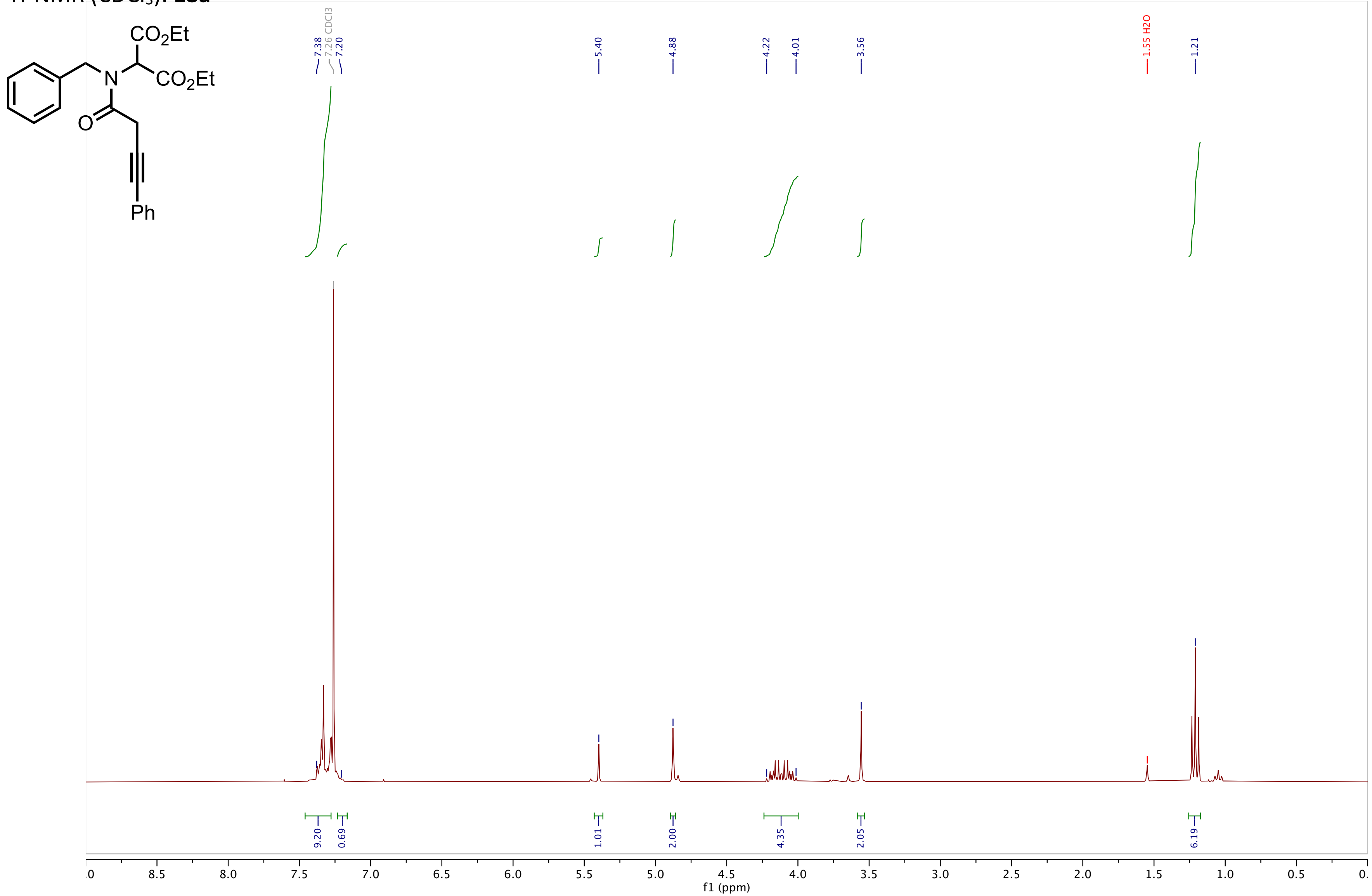

^{1}H-NMR ($CDCl_3$)

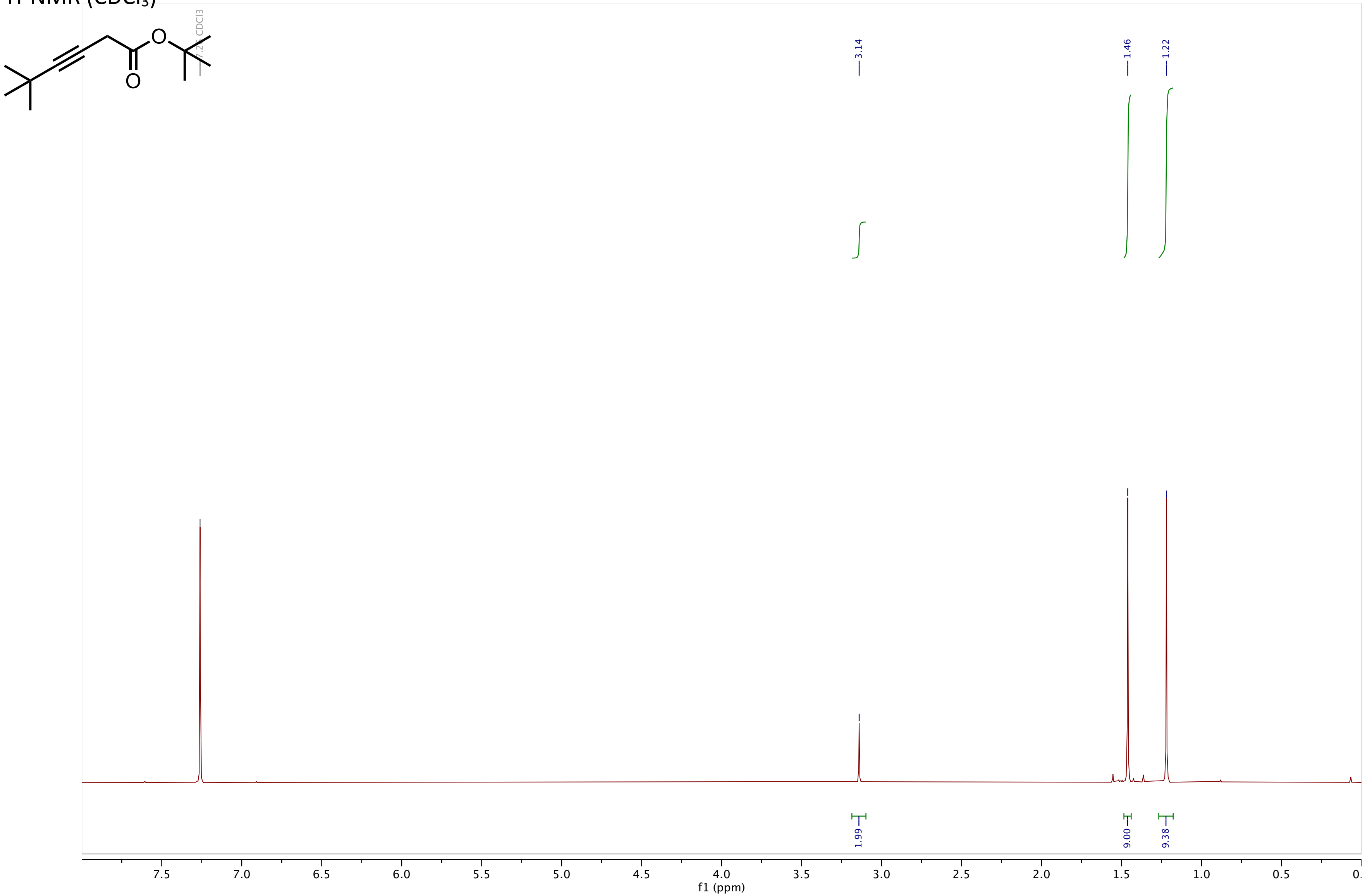
7.26 CDCl3
3.14
1.46
1.22
1.99
9.00
9.38
7.5
7.0
6.5
6.0
5.5
5.0
4.5
4.0
3.5
3.0
2.5
2.0
1.5
1.0
0.5
f1 (ppm)

^{13}C-NMR ($CDCl_3$)

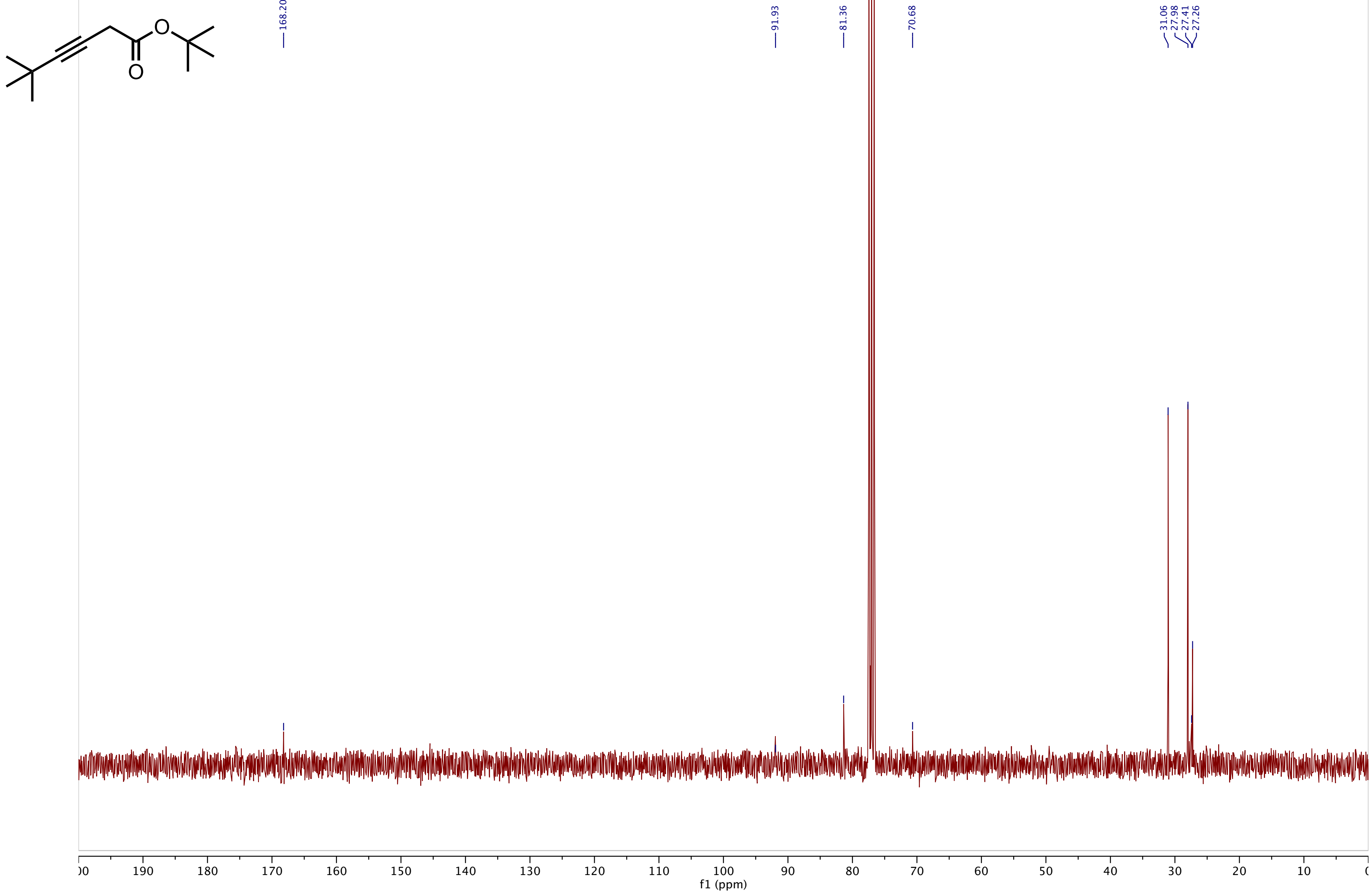

^{1}H-NMR ($CDCl_3$): **19a**

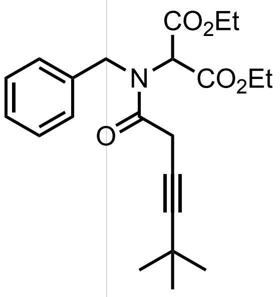

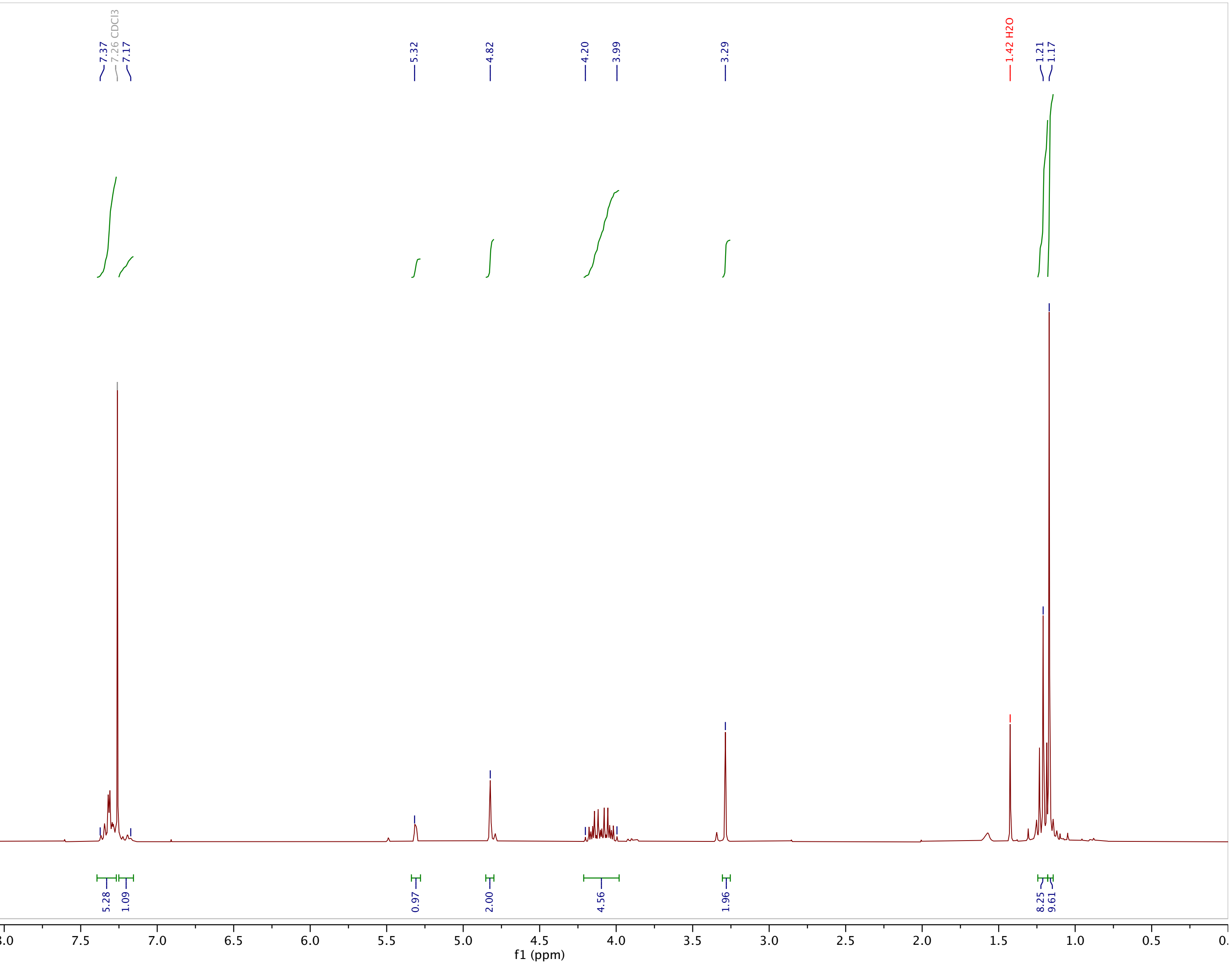

^{13}C-zgpg ($CDCl_3$): **19a**

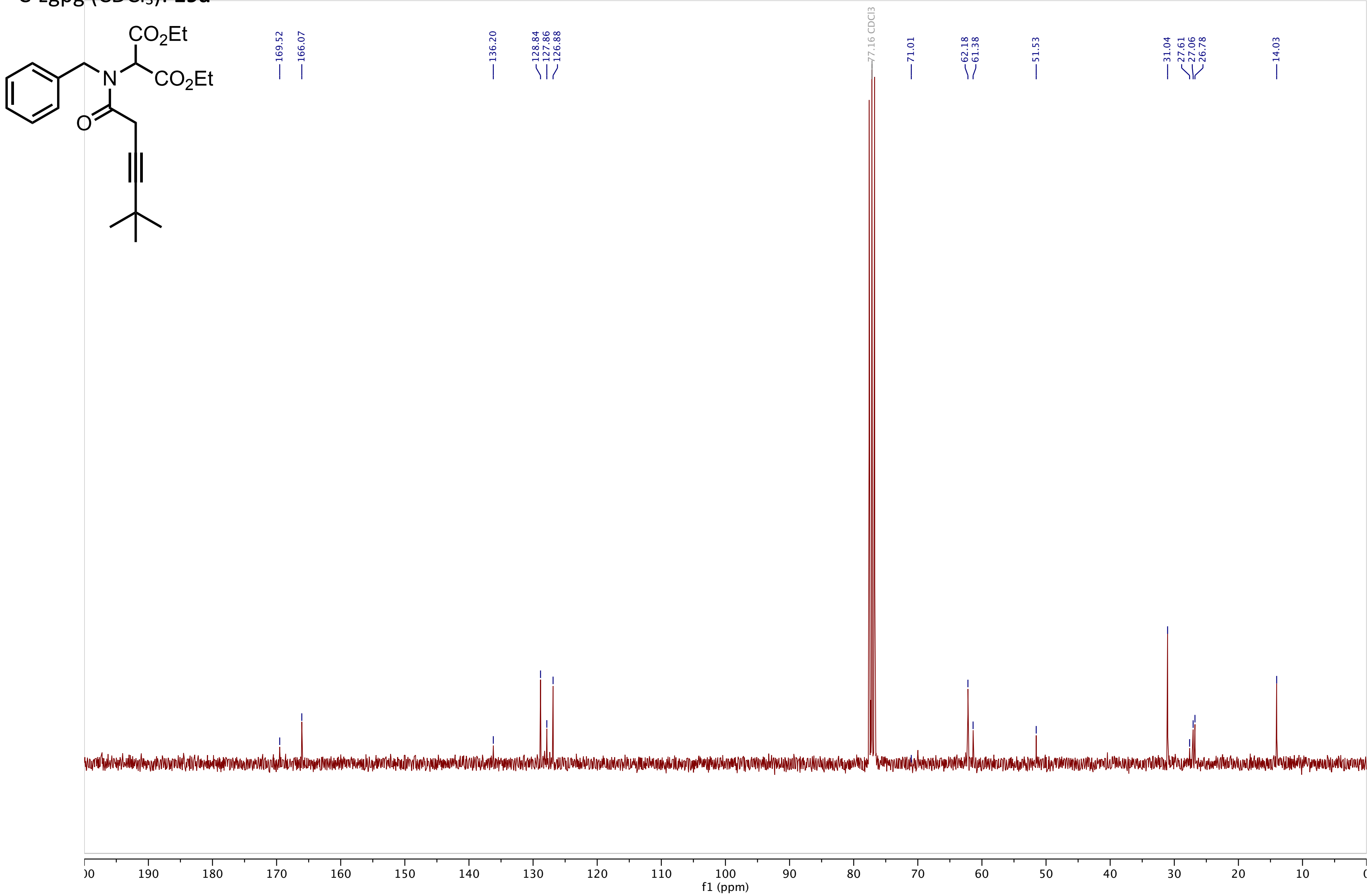

^{13}C-HSQC ($CDCl_3$): **19a**

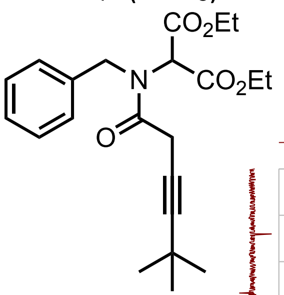

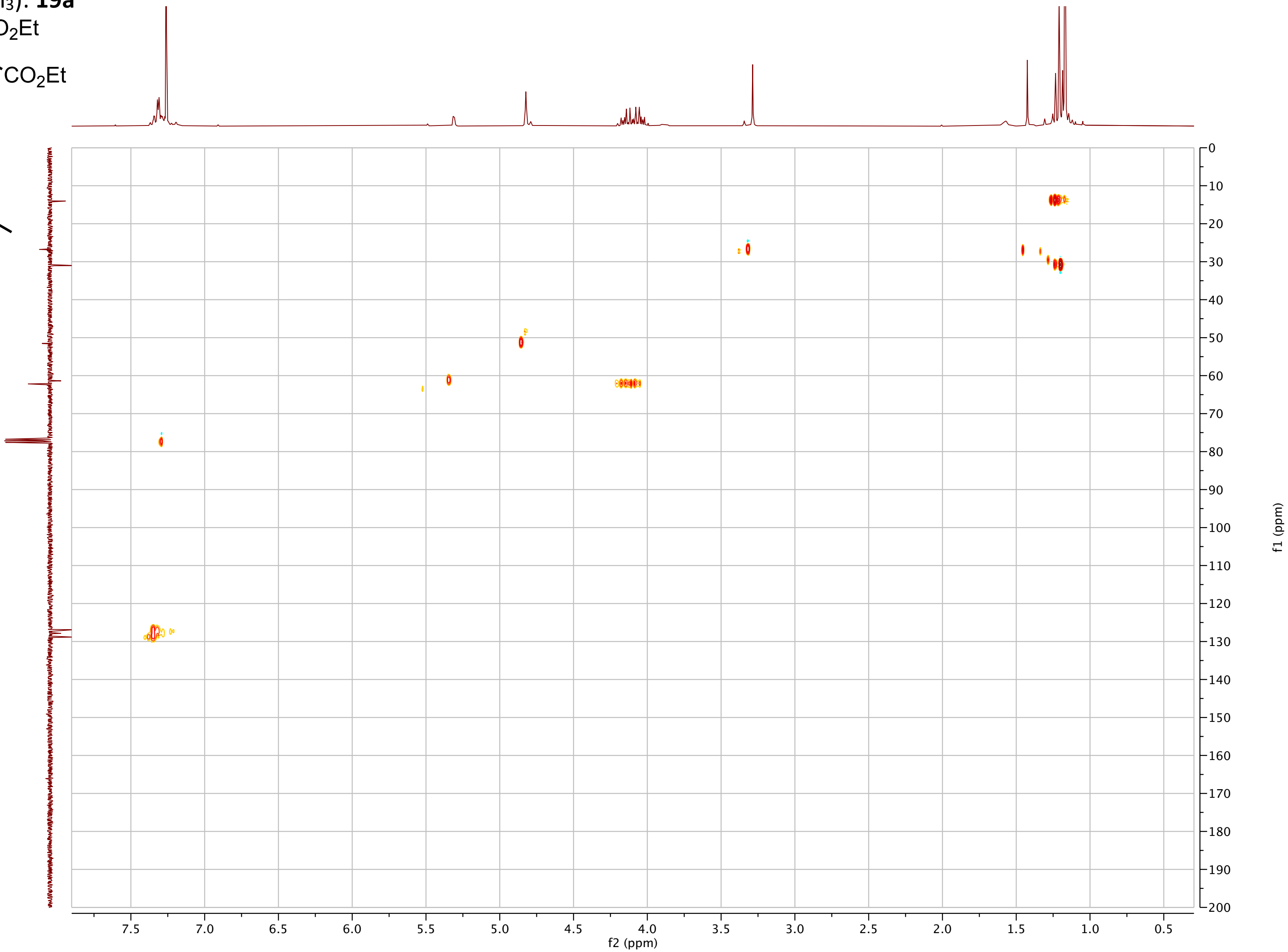

^{13}C-HMBC ($CDCl_3$): **19a**

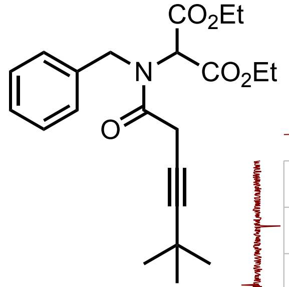

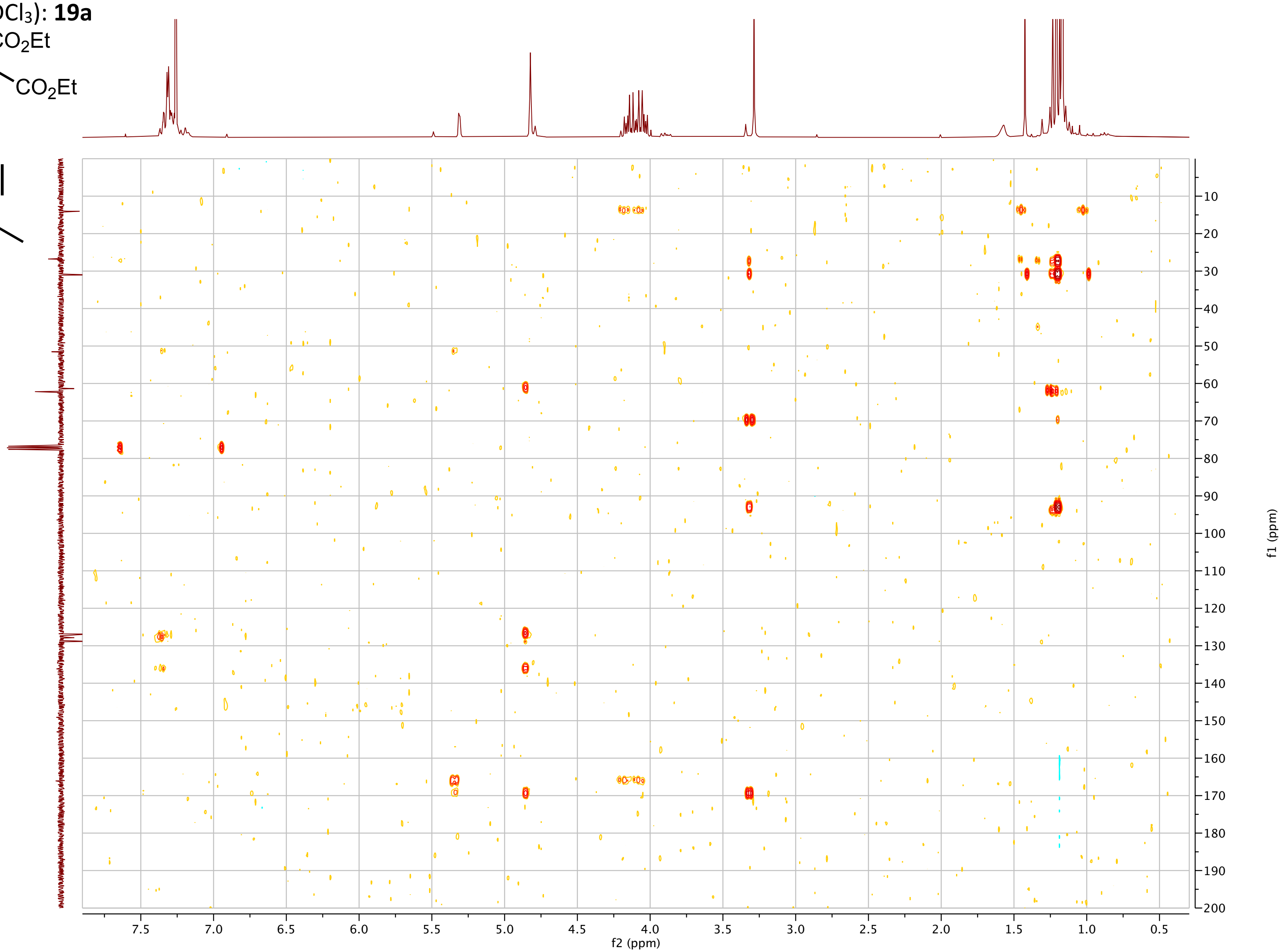

^{1}H-NMR ($CDCl_3$): **1b**

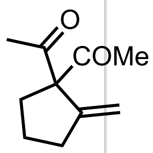

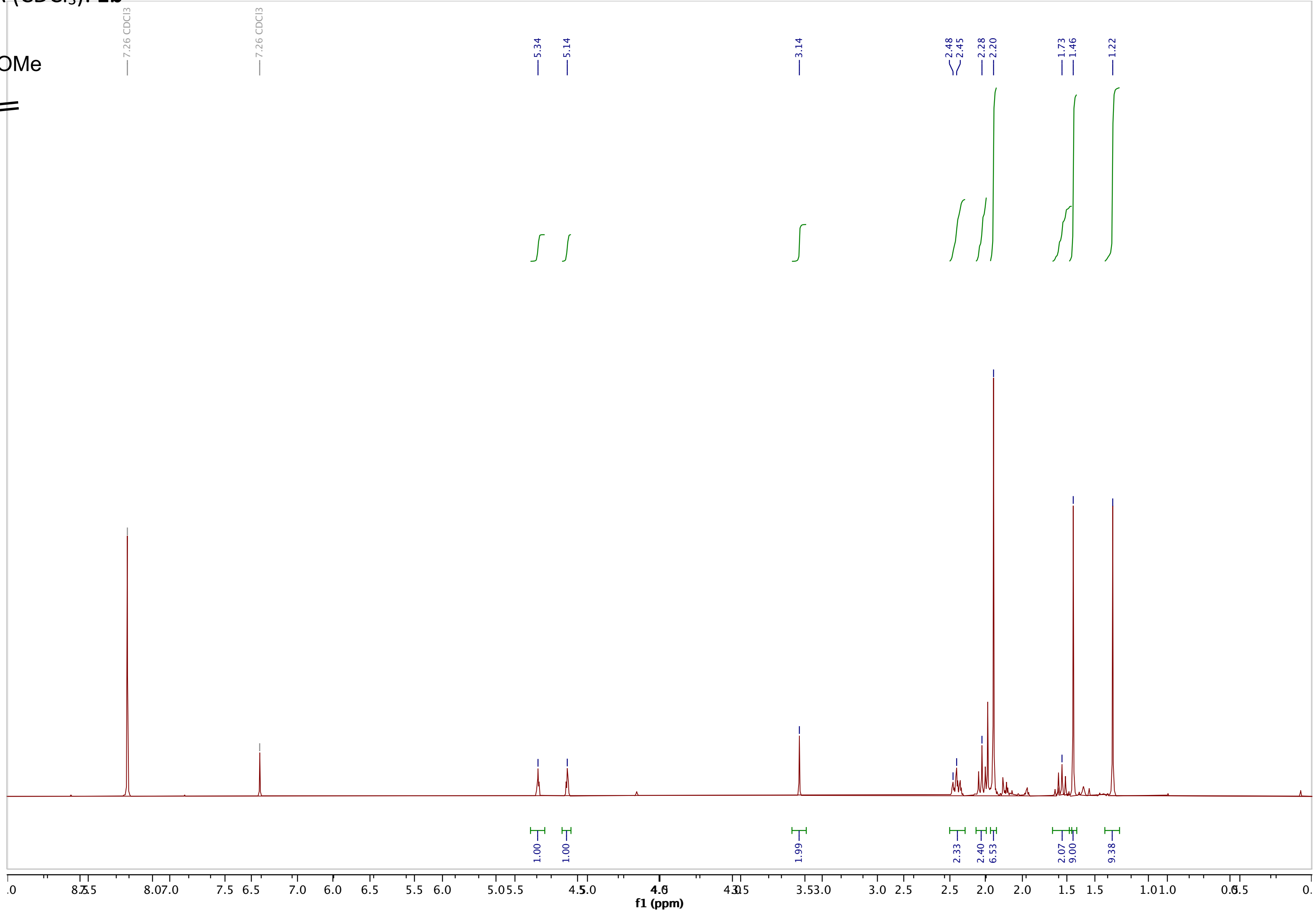

^{1}H-NMR ($CDCl_3$): **2b**

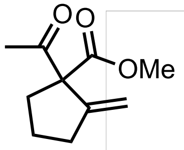

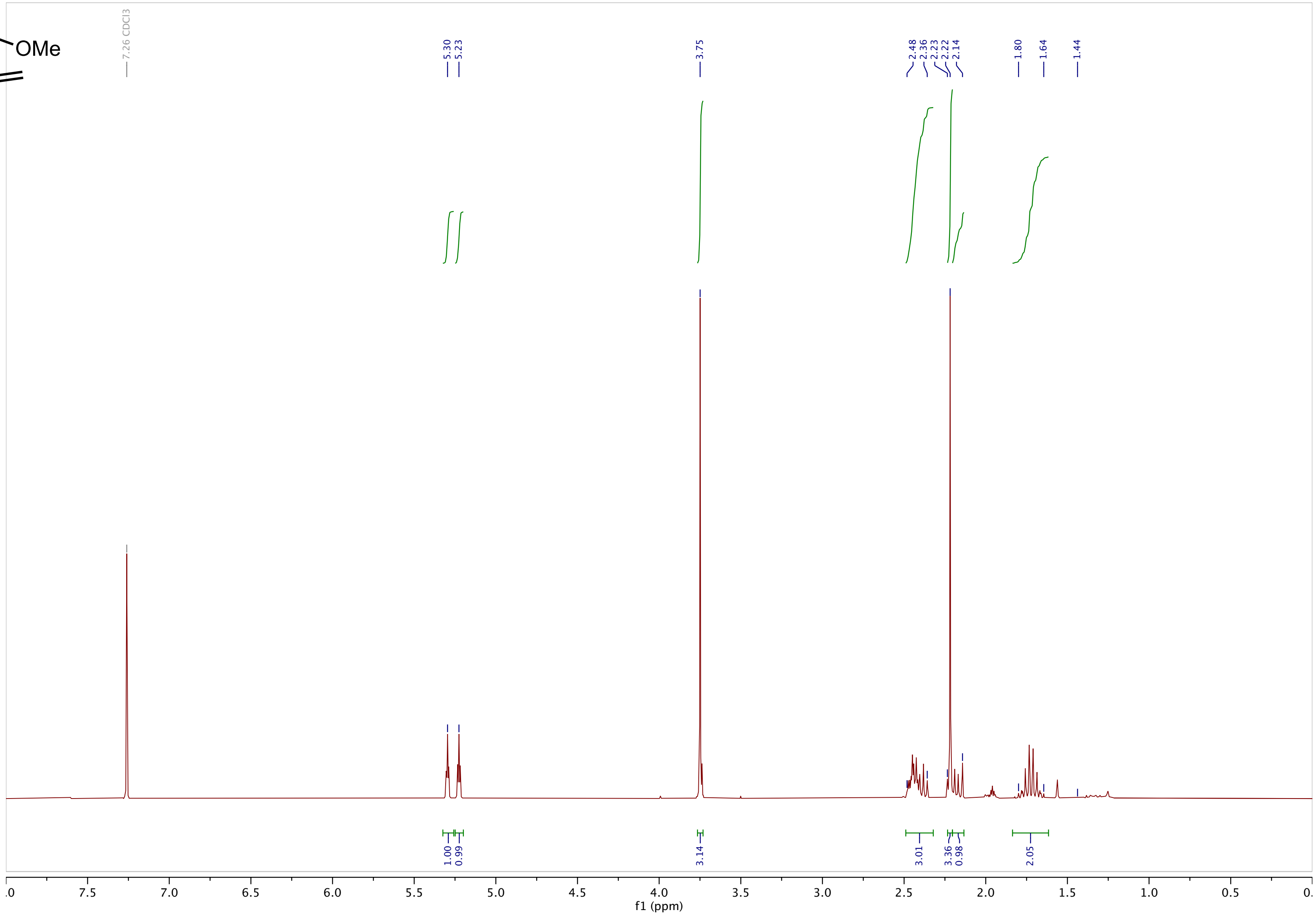

^{1}H-NMR ($CDCl_3$): **3b**

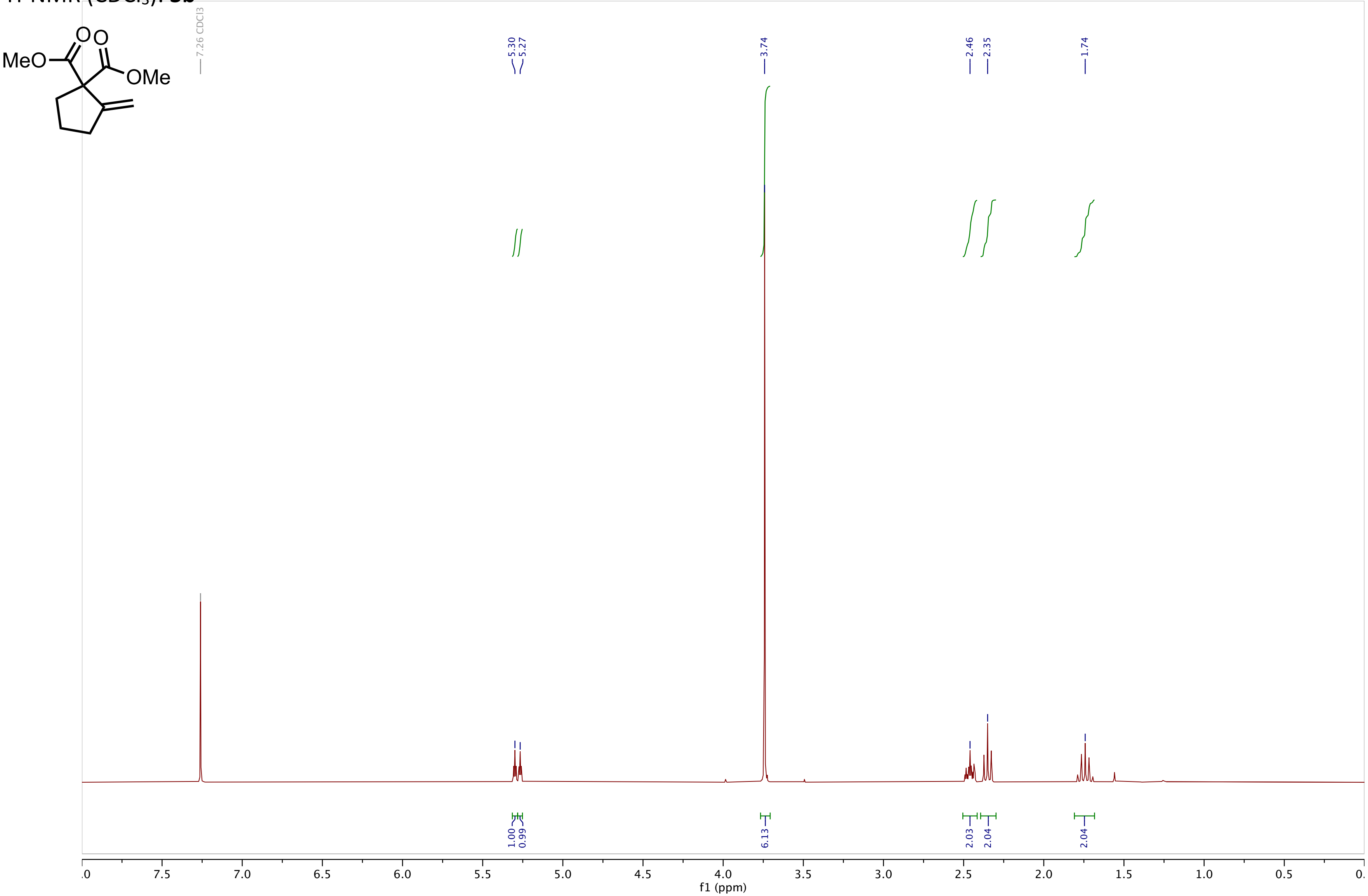

^{1}H-NMR ($CDCl_3$): **4b**

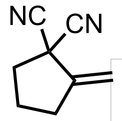

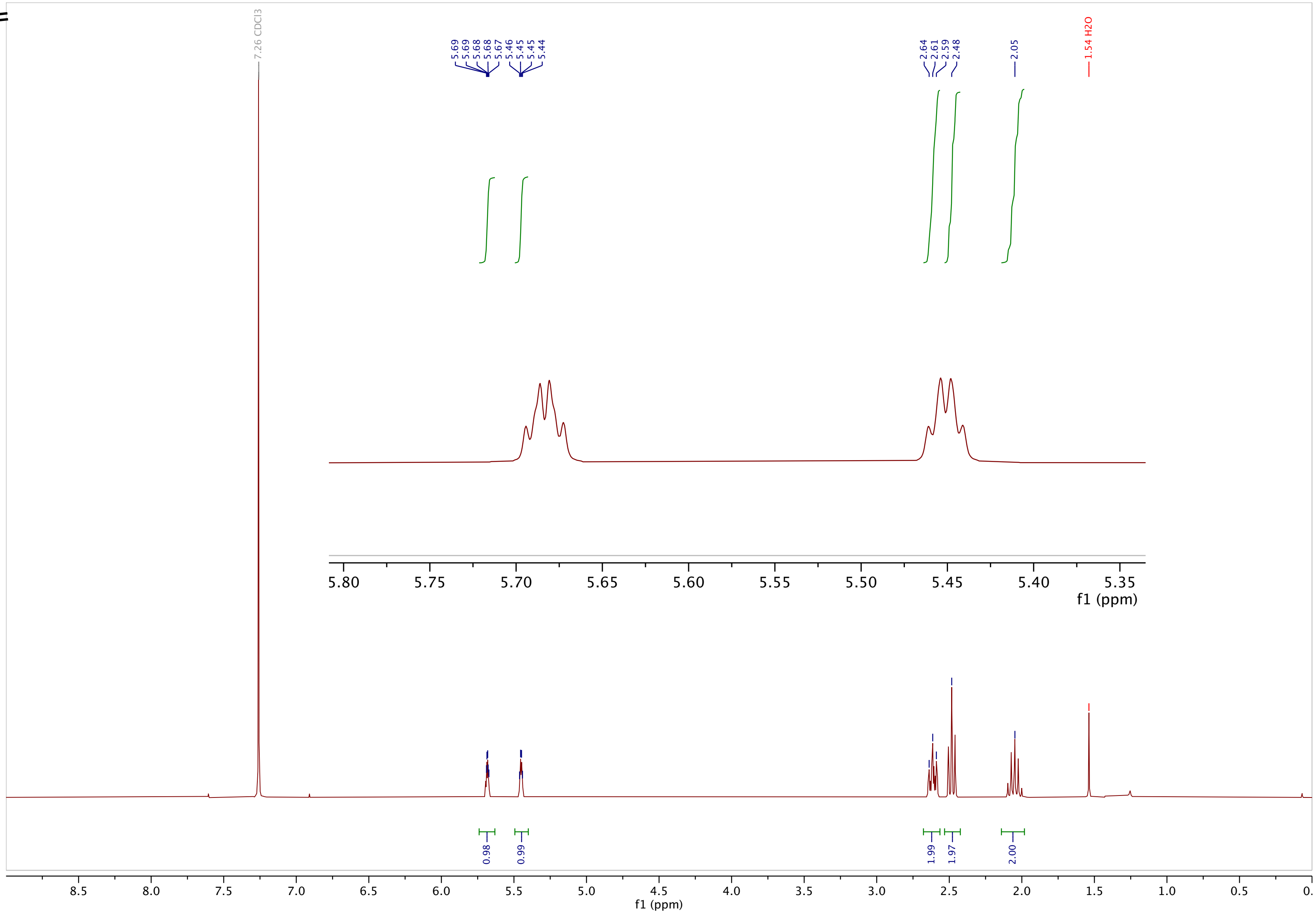

^{1}H-NMR ($CDCl_3$): **5b**

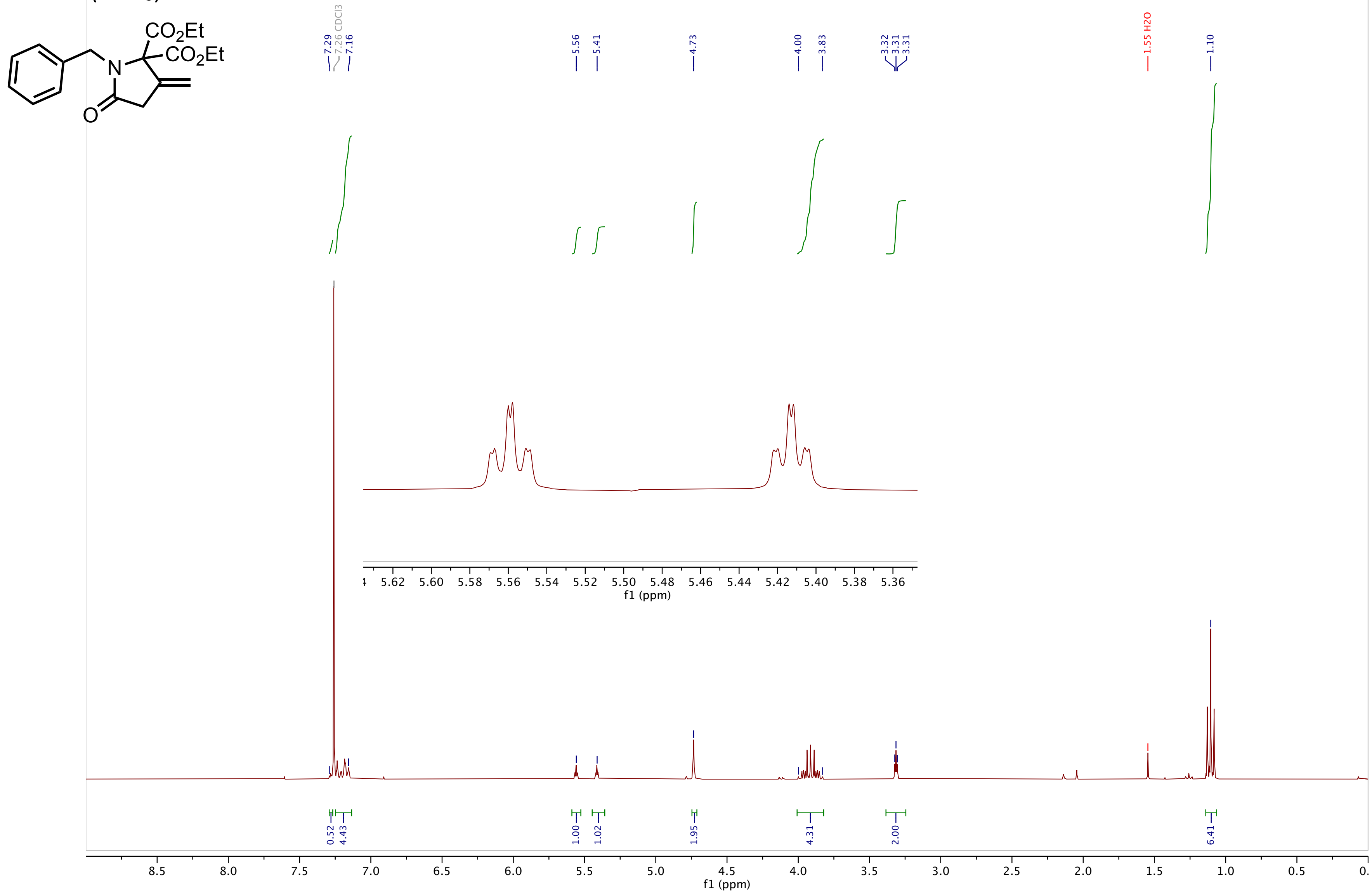

^{1}H-NMR ($CDCl_3$): **6b**

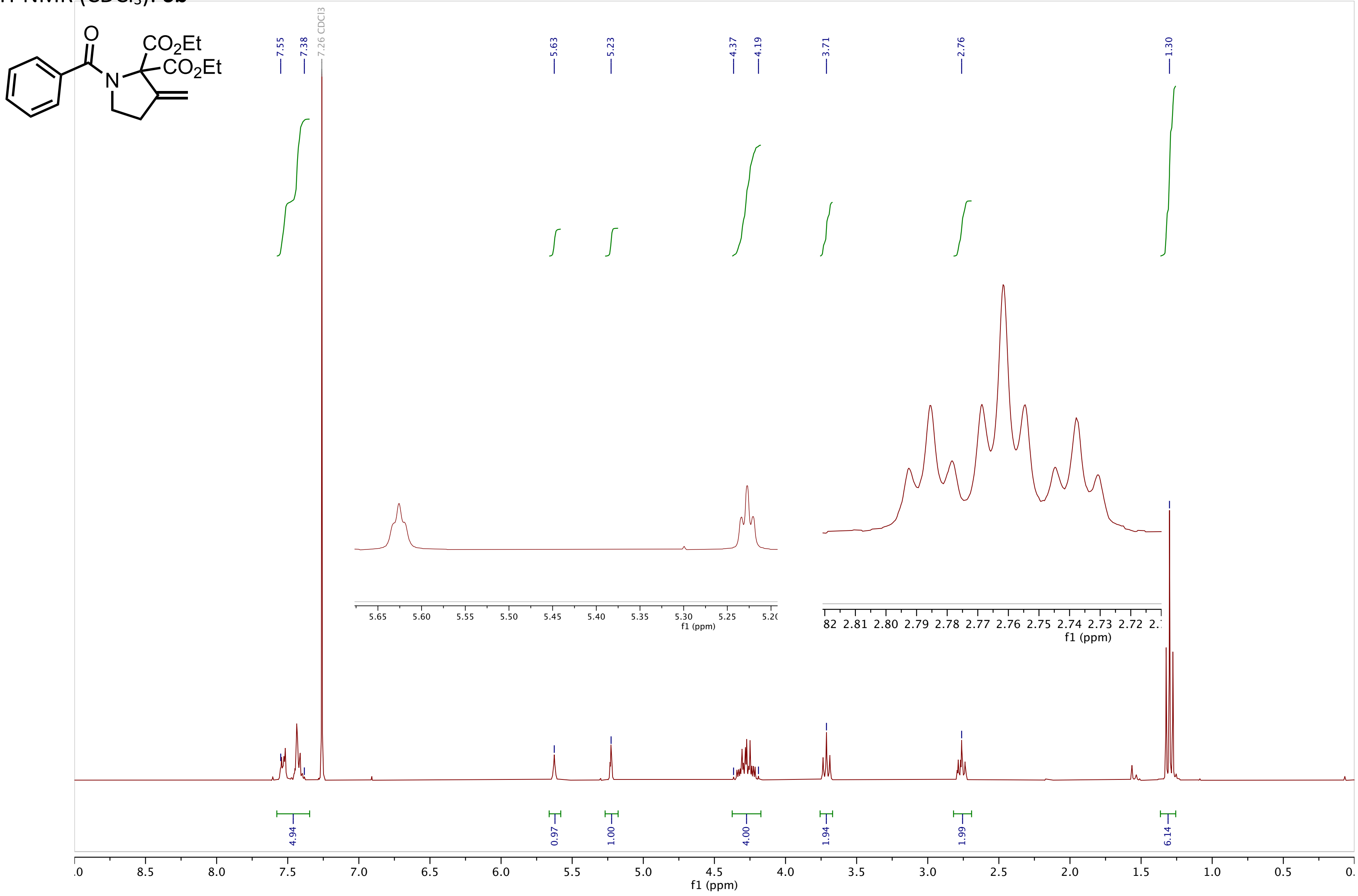

^{13}C-zgpg ($CDCl_3$): **6b**

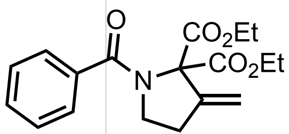

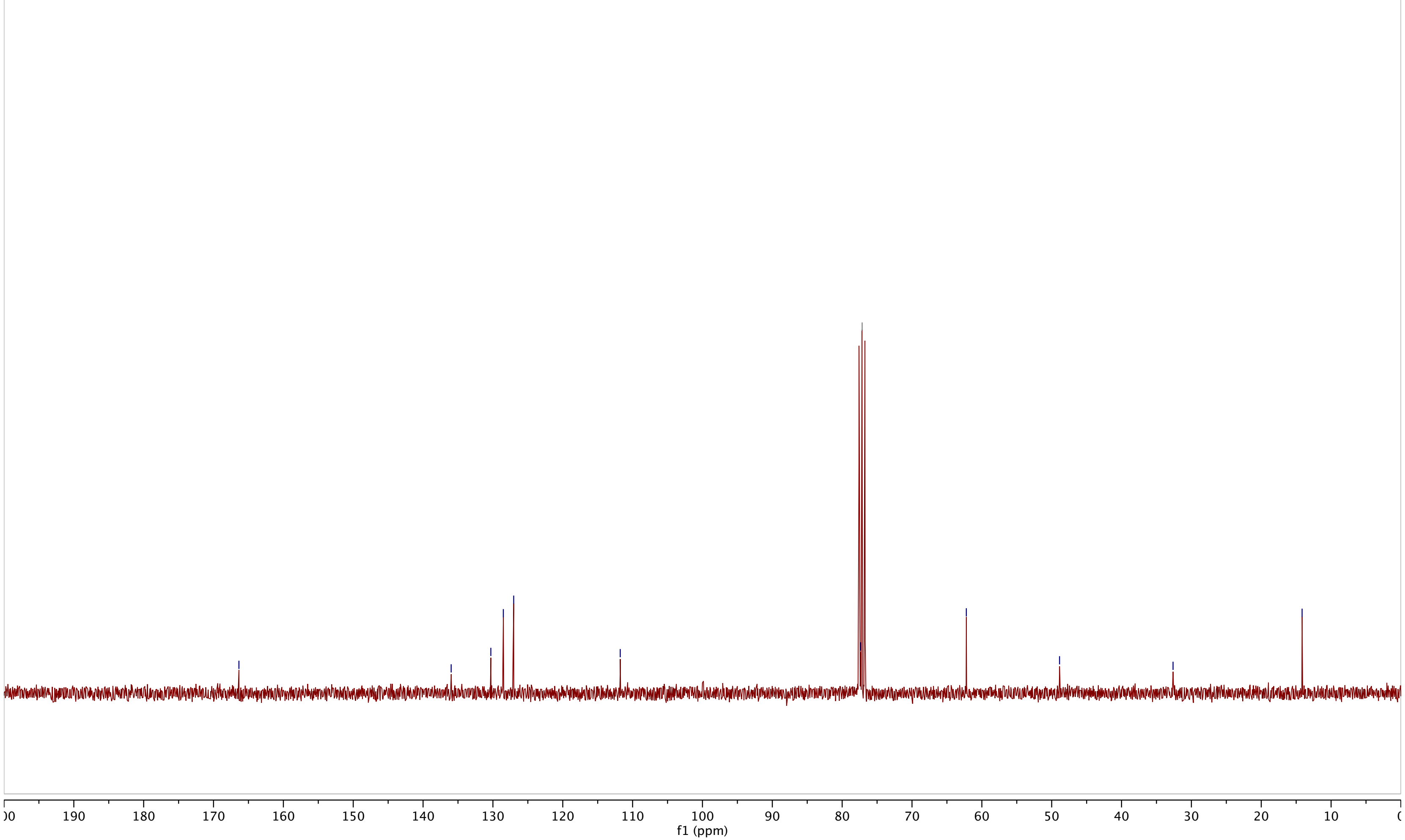

^{1}H-NMR ($CDCl_3$): **7b**

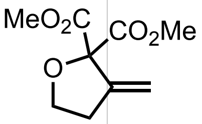

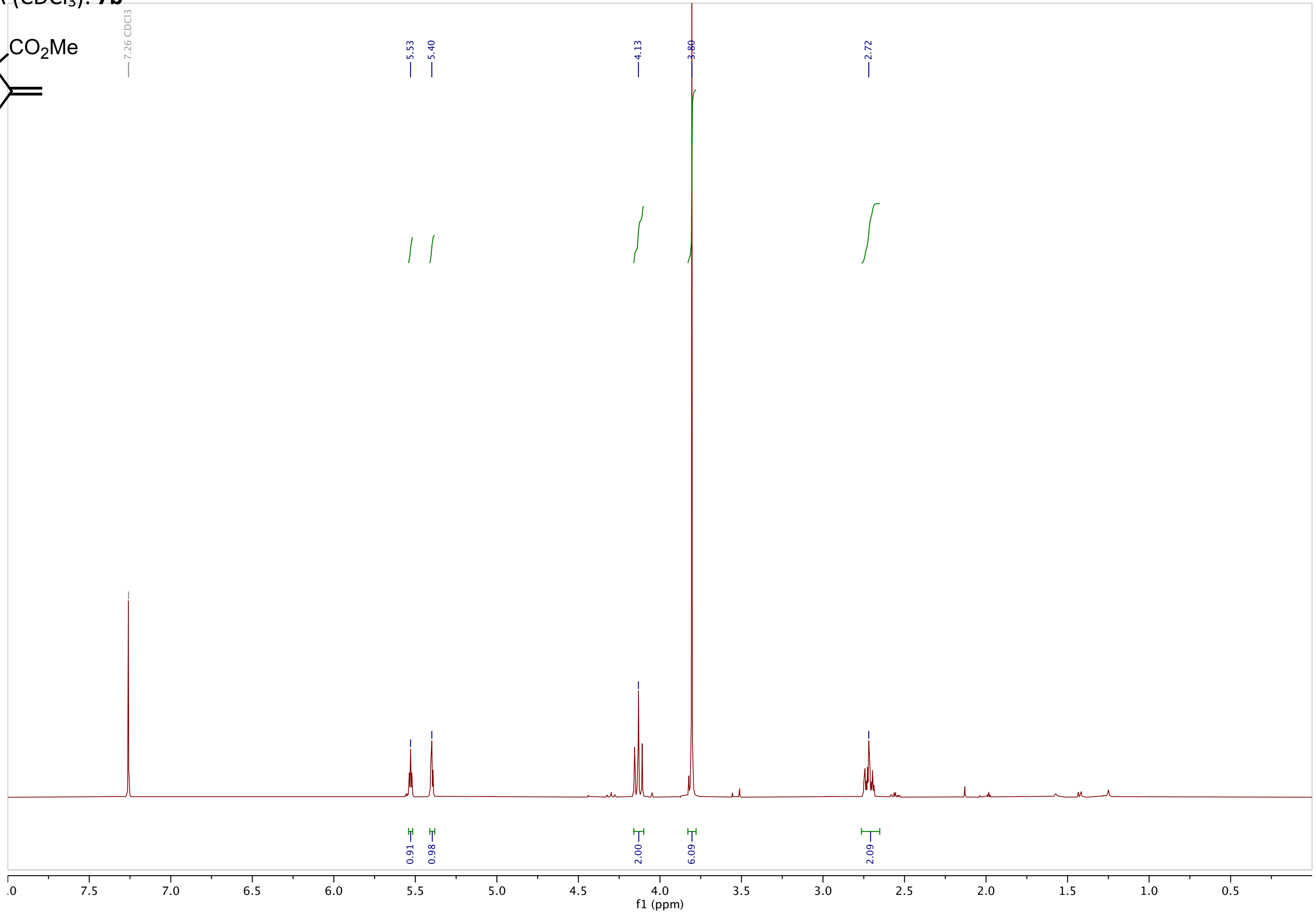

^{1}H-NMR ($CDCl_3$): **8b**

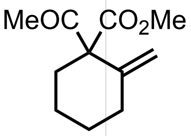

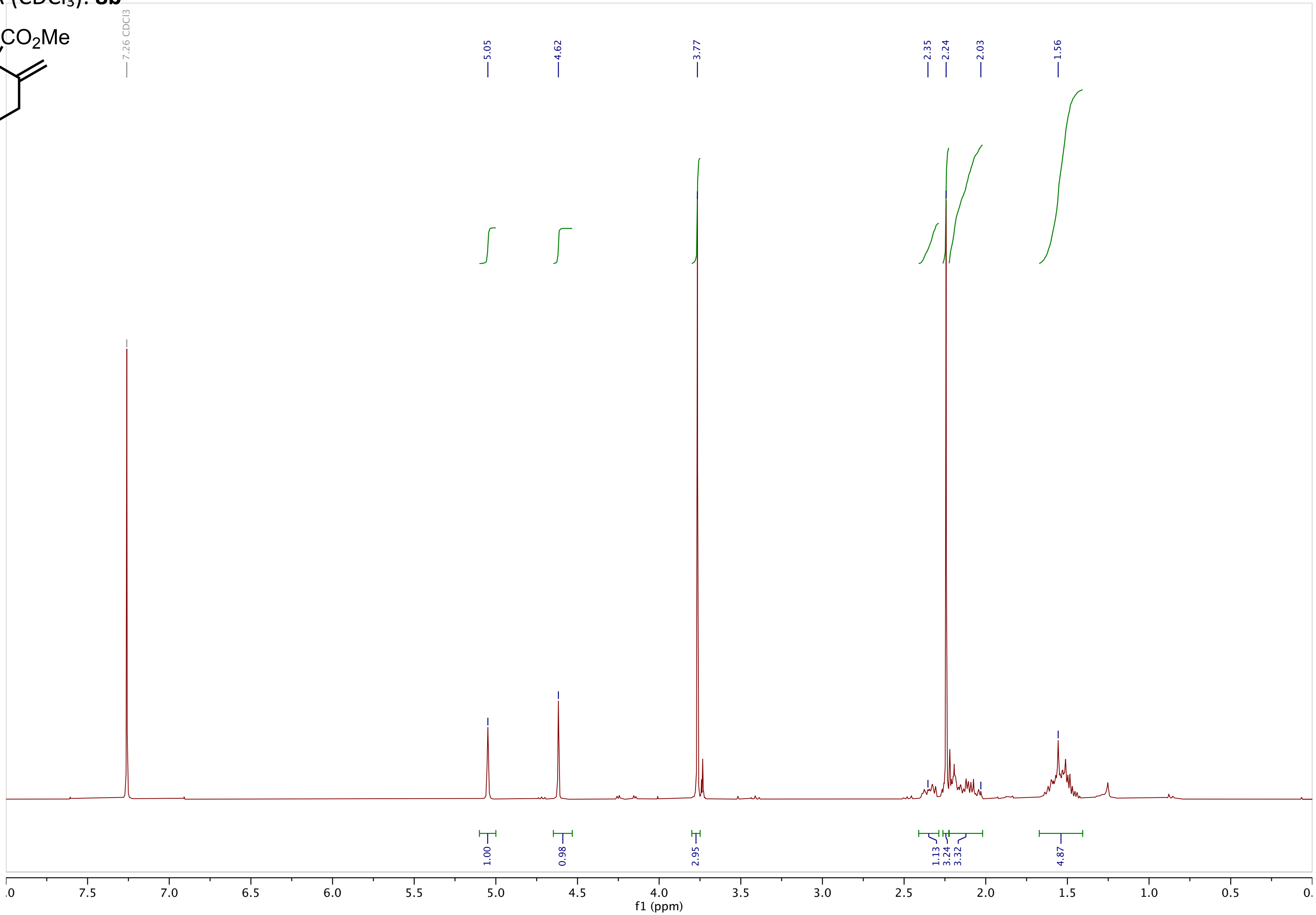

^{1}H-NMR ($CDCl_3$): **9b**

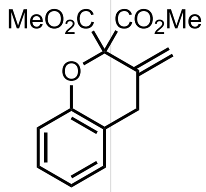

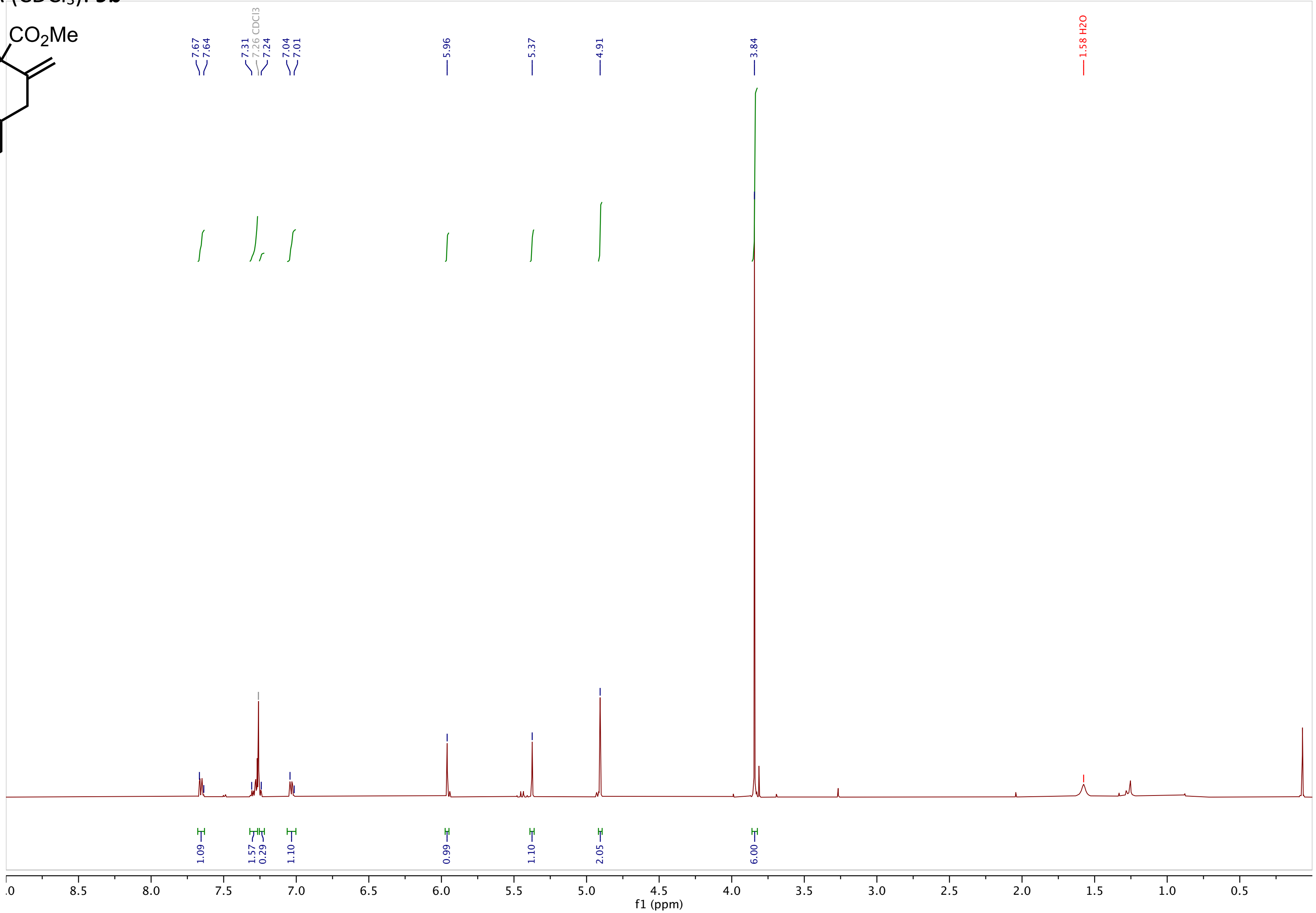

^{13}C-NMR ($CDCl_3$): **9b**

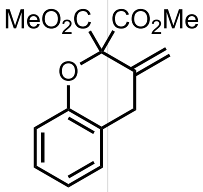

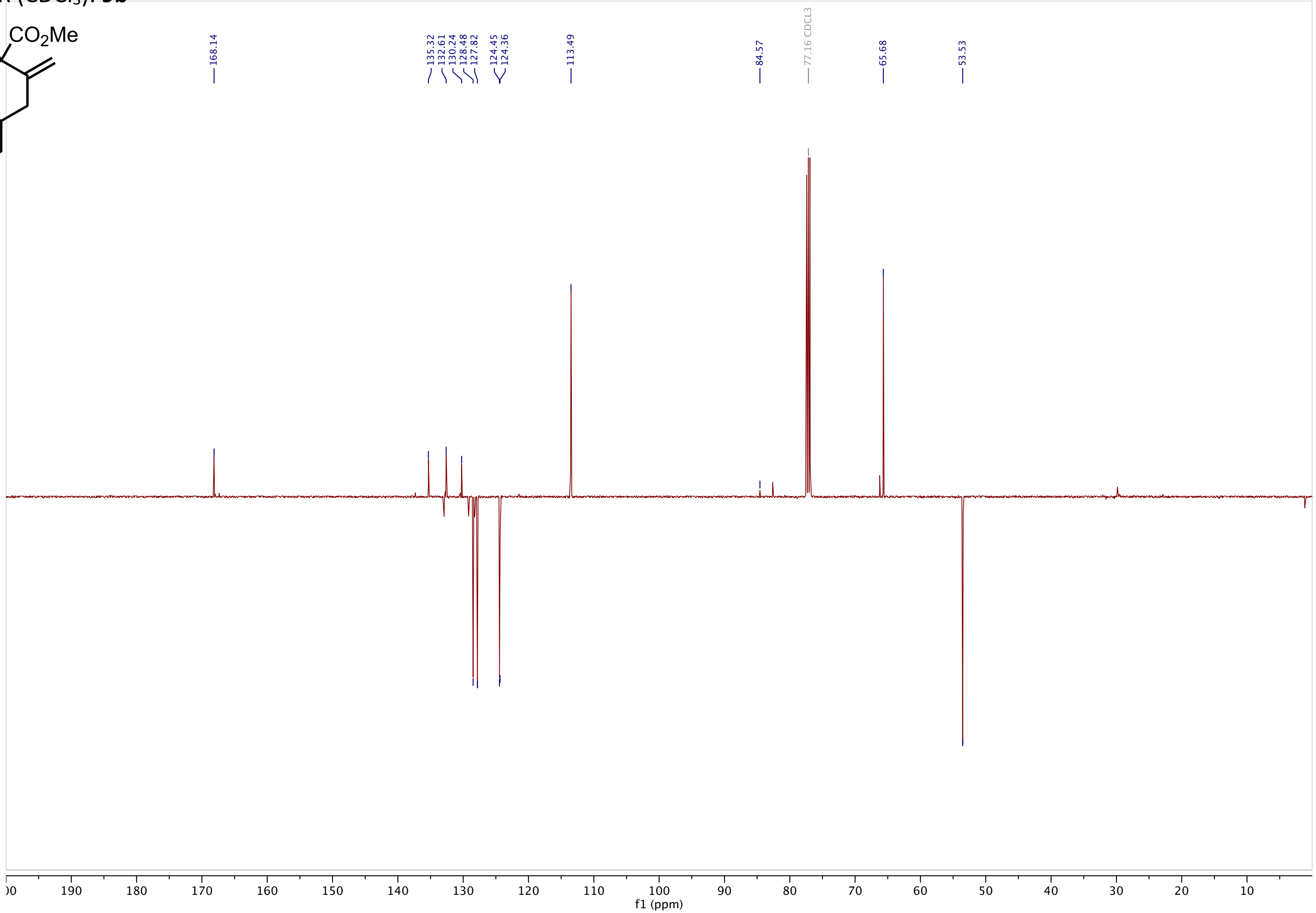

^{1}H-NMR ($CDCl_3$): **10b**

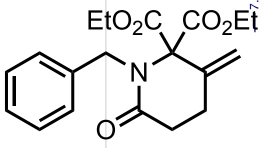

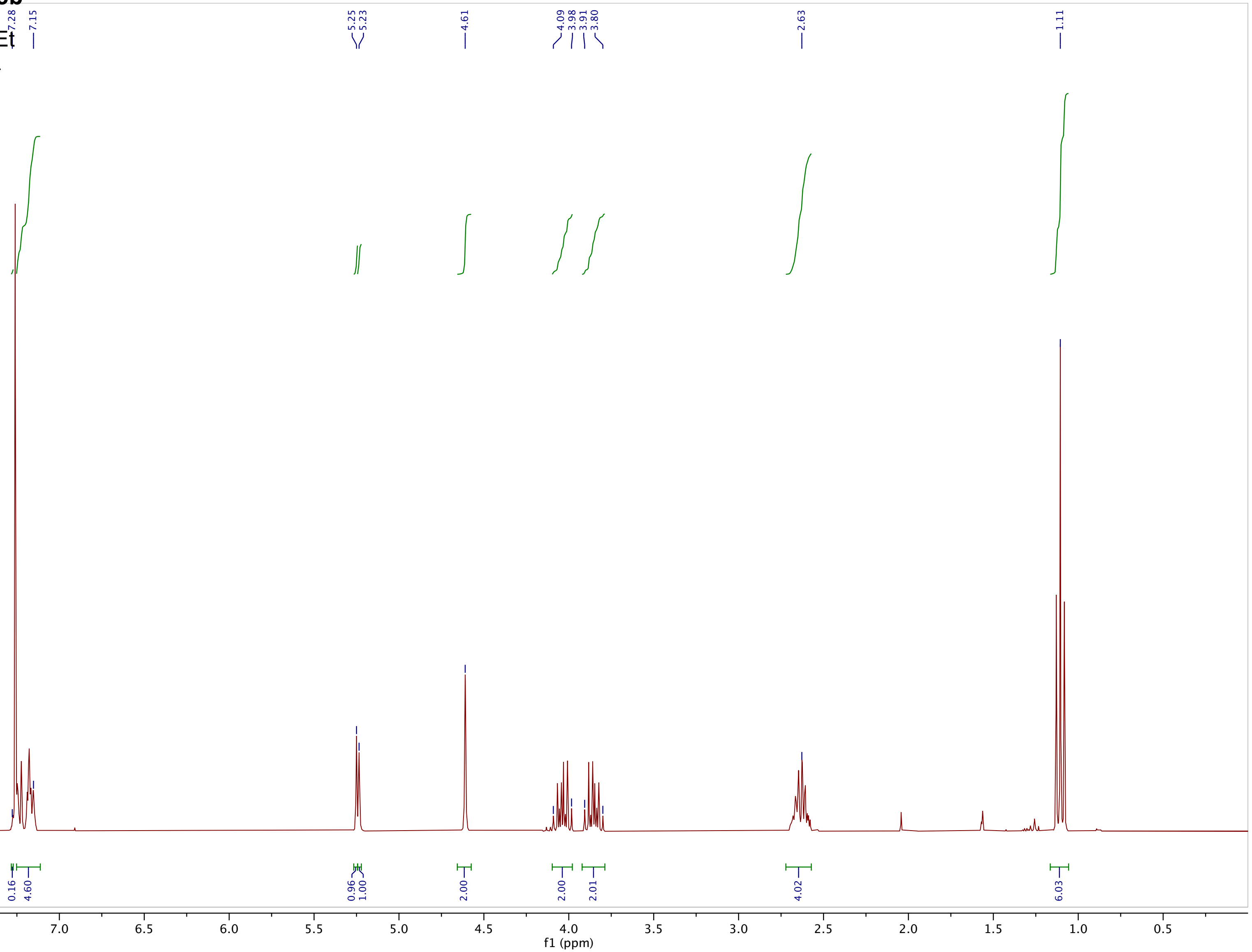

^{1}H-NMR ($CDCl_3$): **11b**

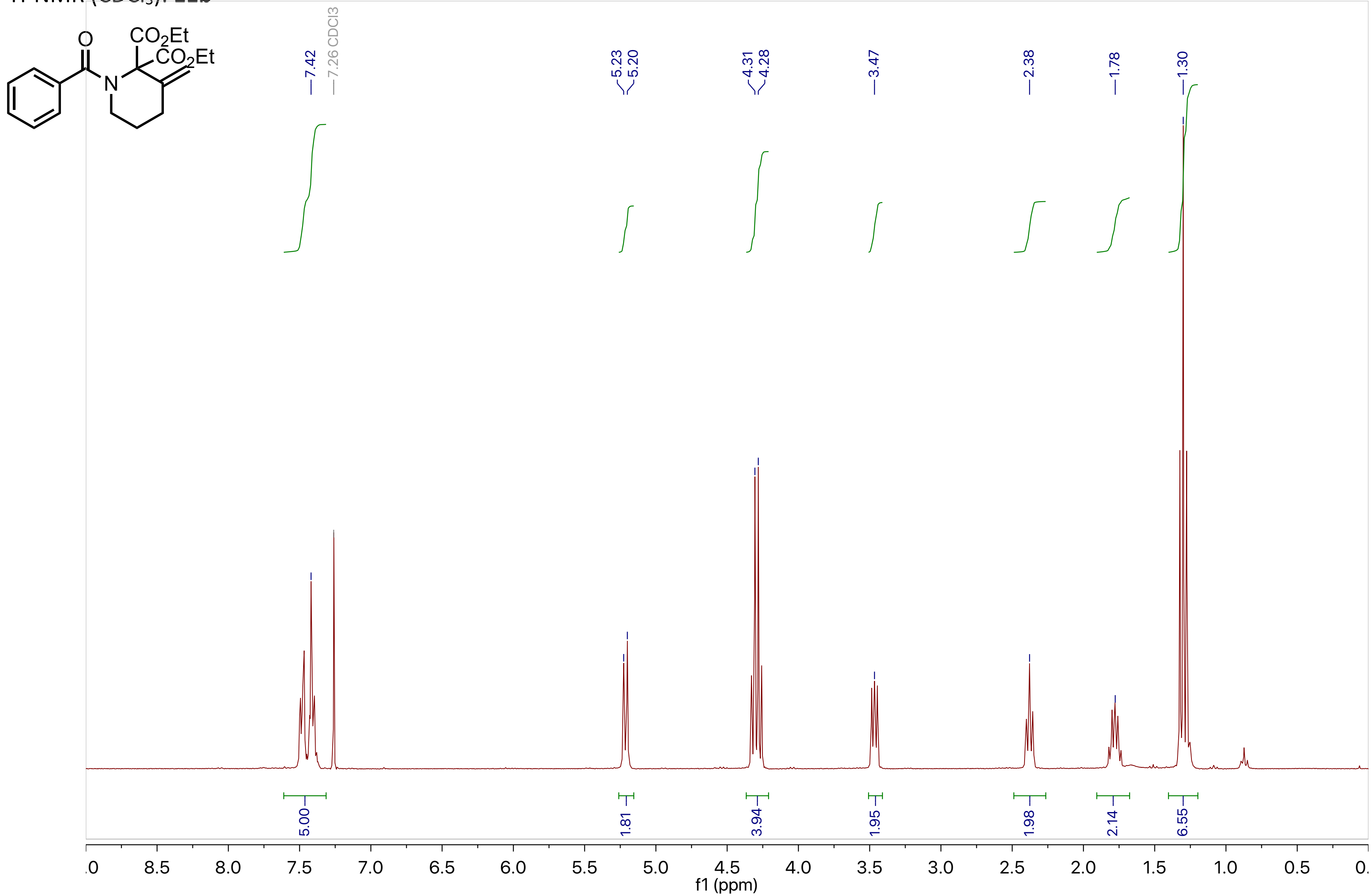

^{13}C-zgpg ($CDCl_3$): **11b**

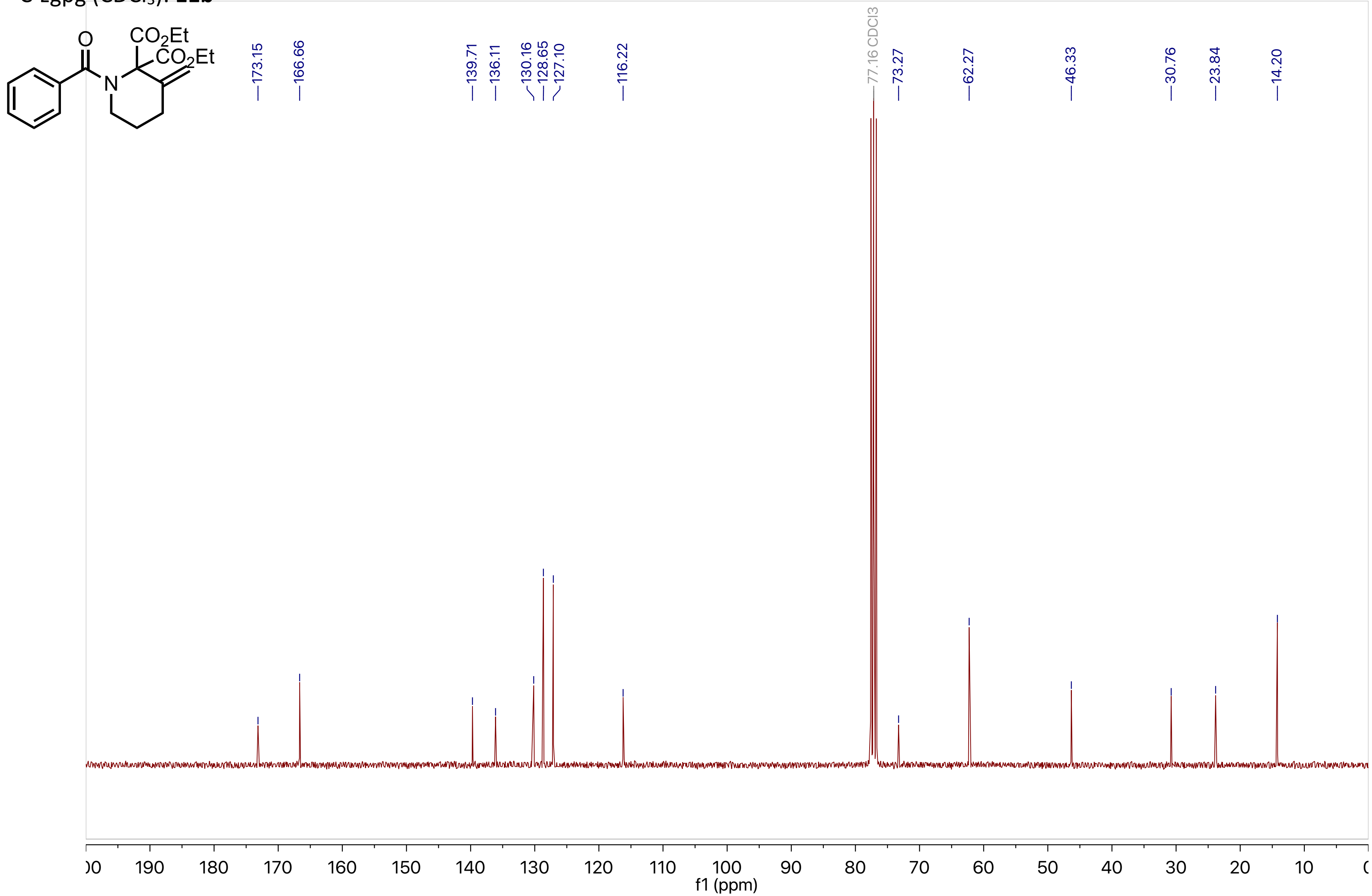

^{1}H-NMR ($CDCl_3$): **12b**

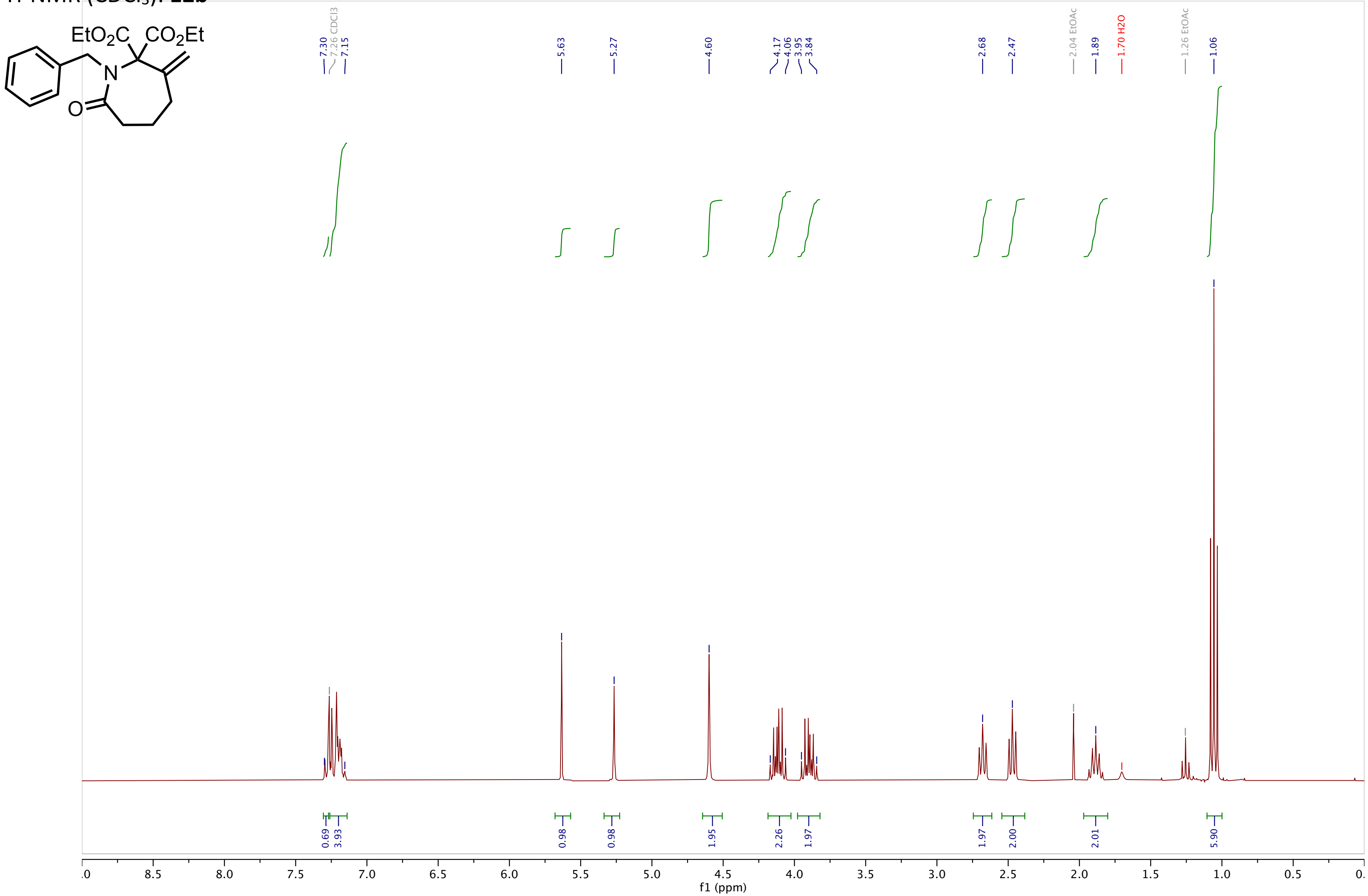

^{13}C-zgpg ($CDCl_3$): **12b**

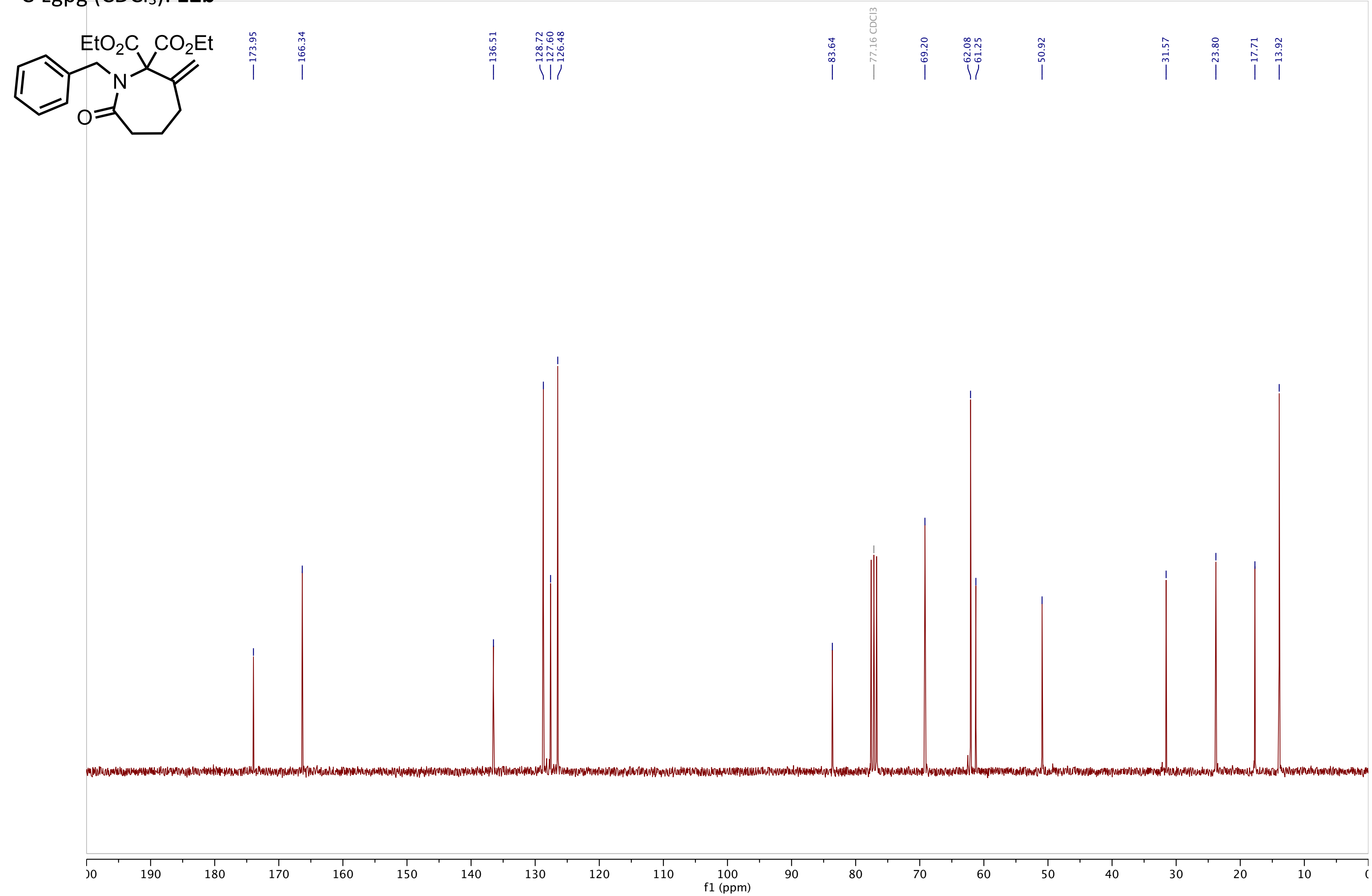

^{1}H-NMR ($CDCl_3$): **13b**

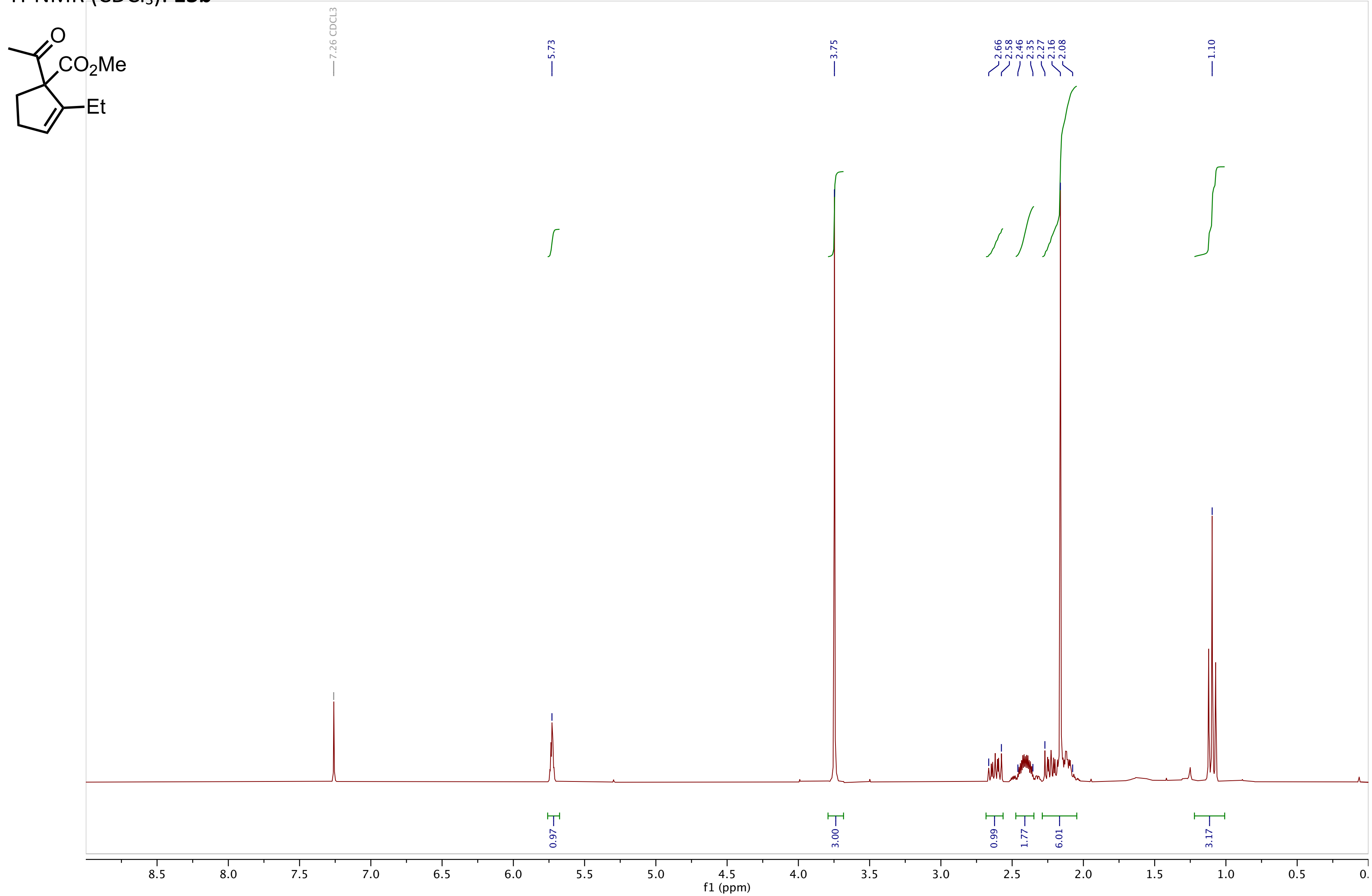

^{1}H-NMR ($CDCl_3$): **14b**

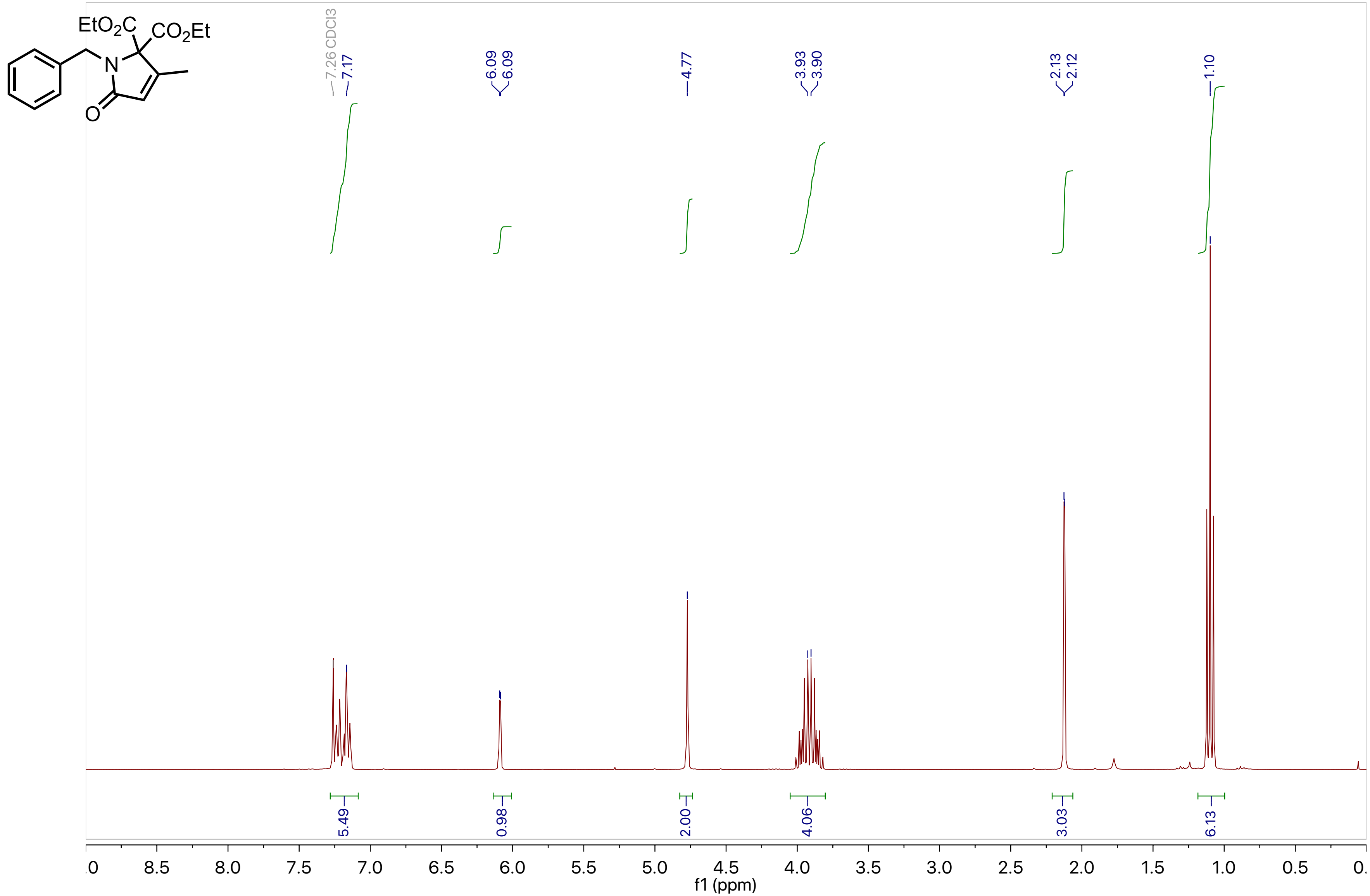

[13]C-zgpg ($CDCl_3$): **14b**

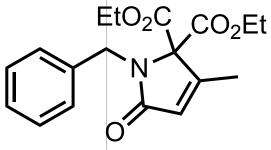

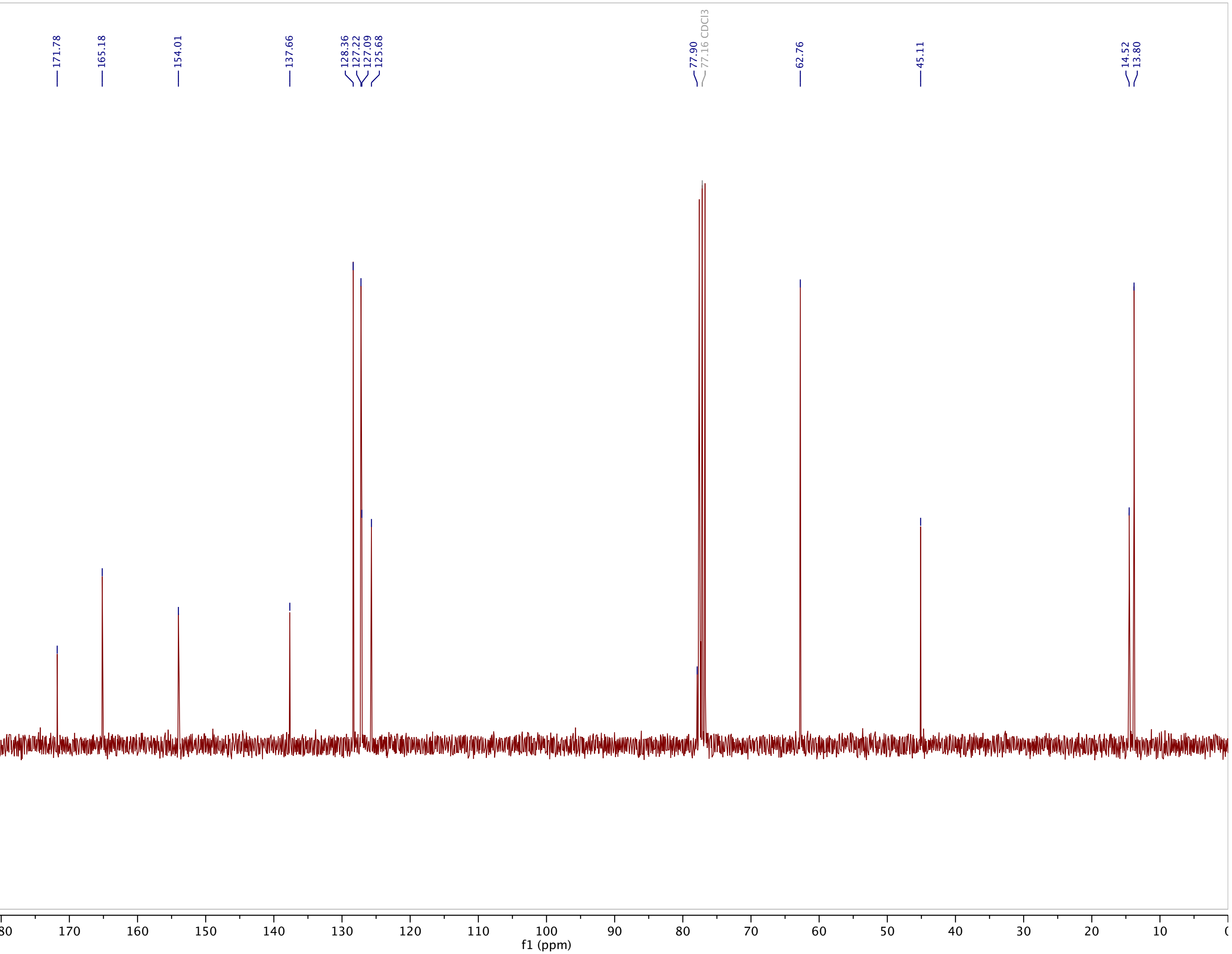

^{1}H-NMR ($CDCl_3$): **15b**

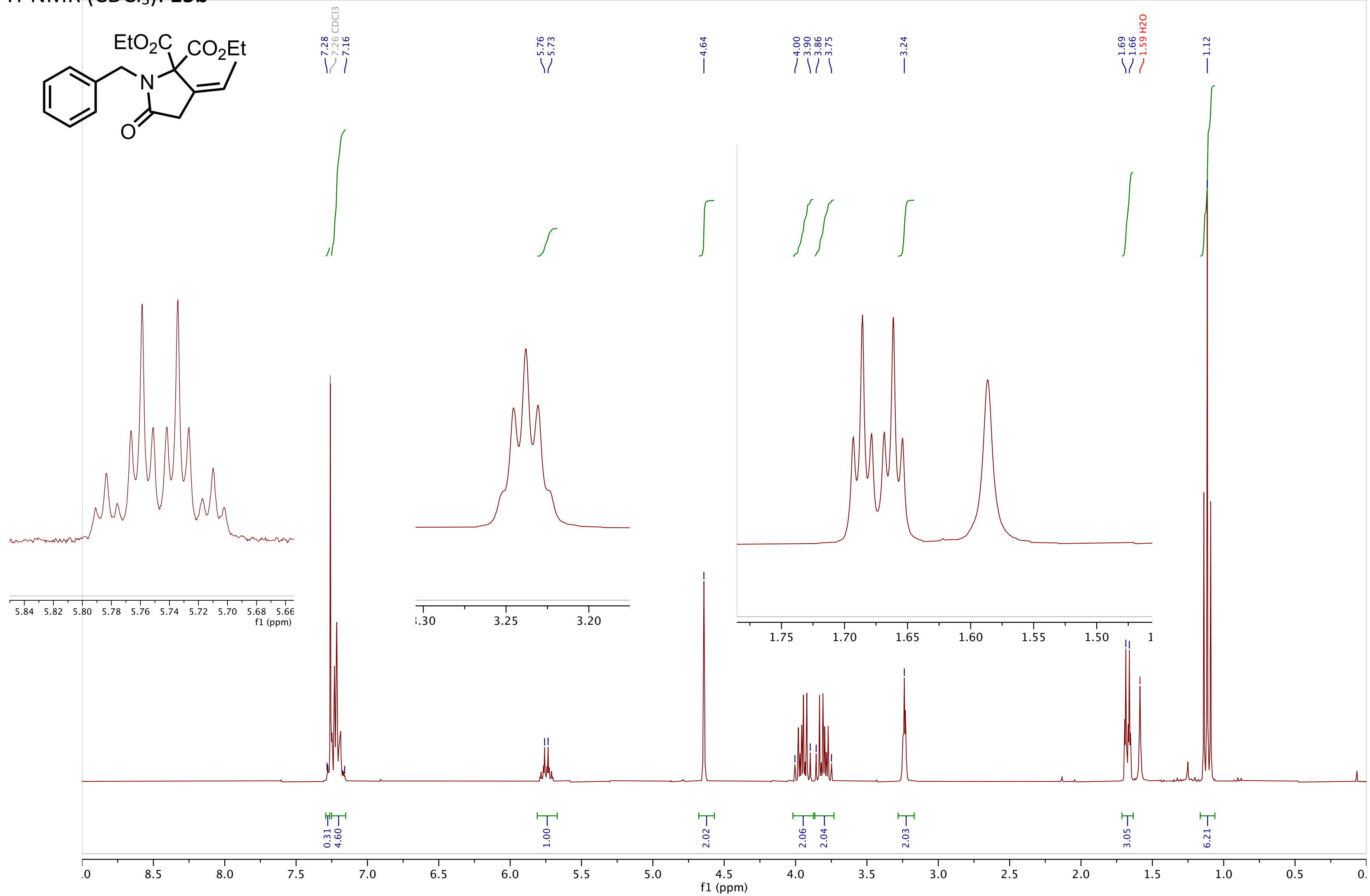

^{1}H-NMR ($CDCl_3$): **16b**

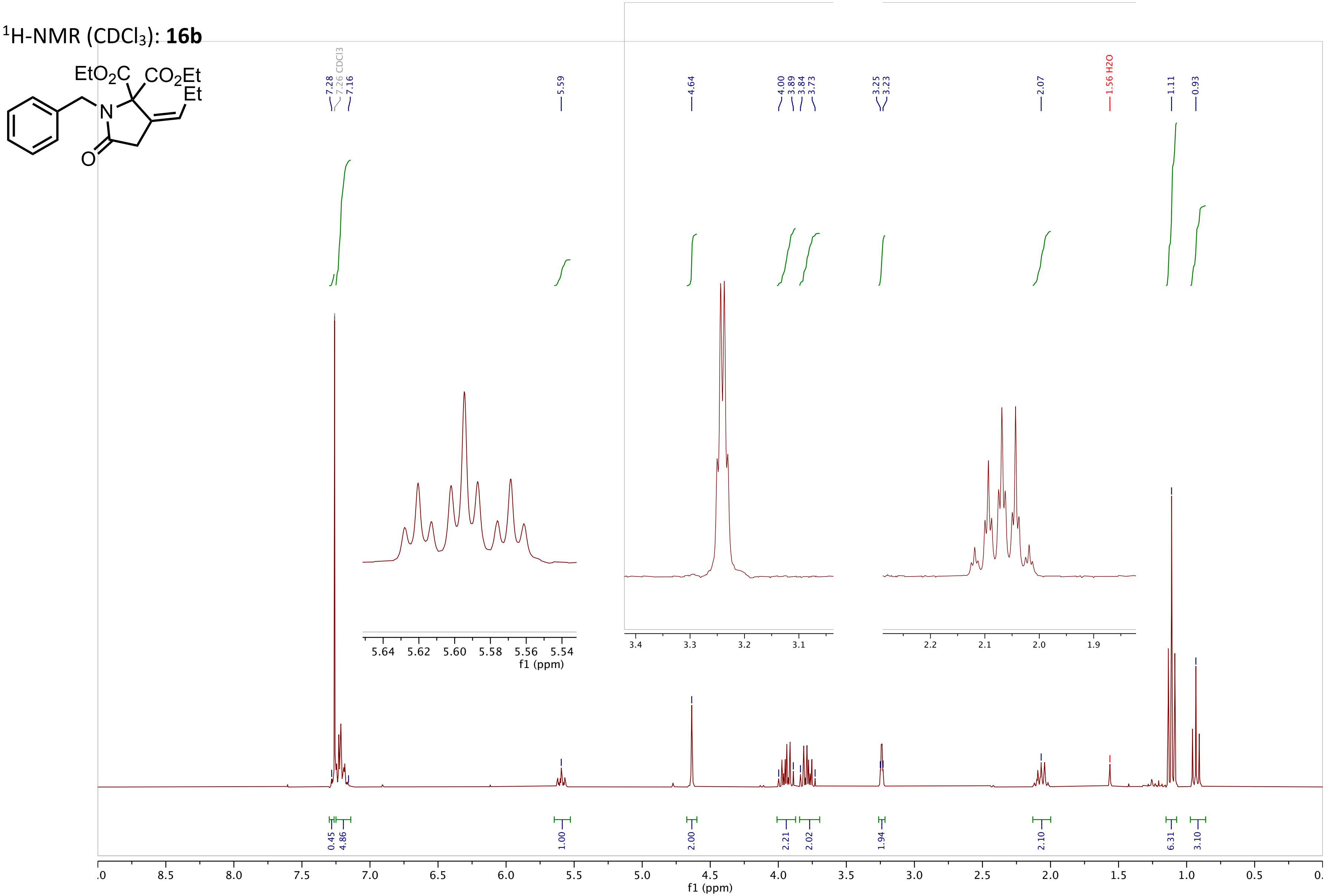

^{1}H-NMR ($CDCl_3$): **17b - *Z***

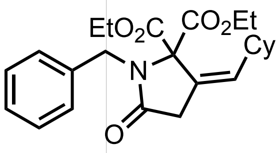

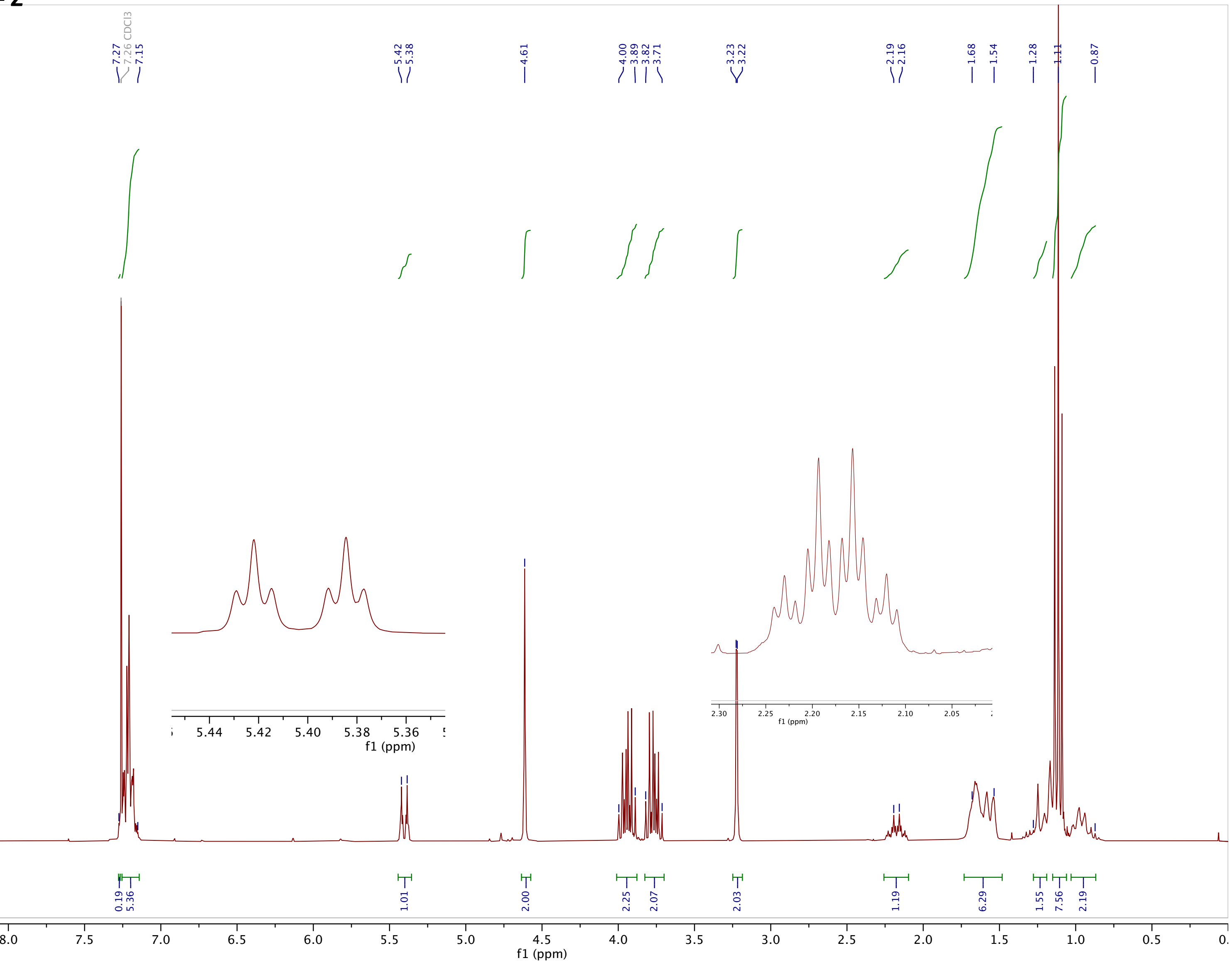

^{13}C-jmod ($CDCl_3$): **17b - *Z***

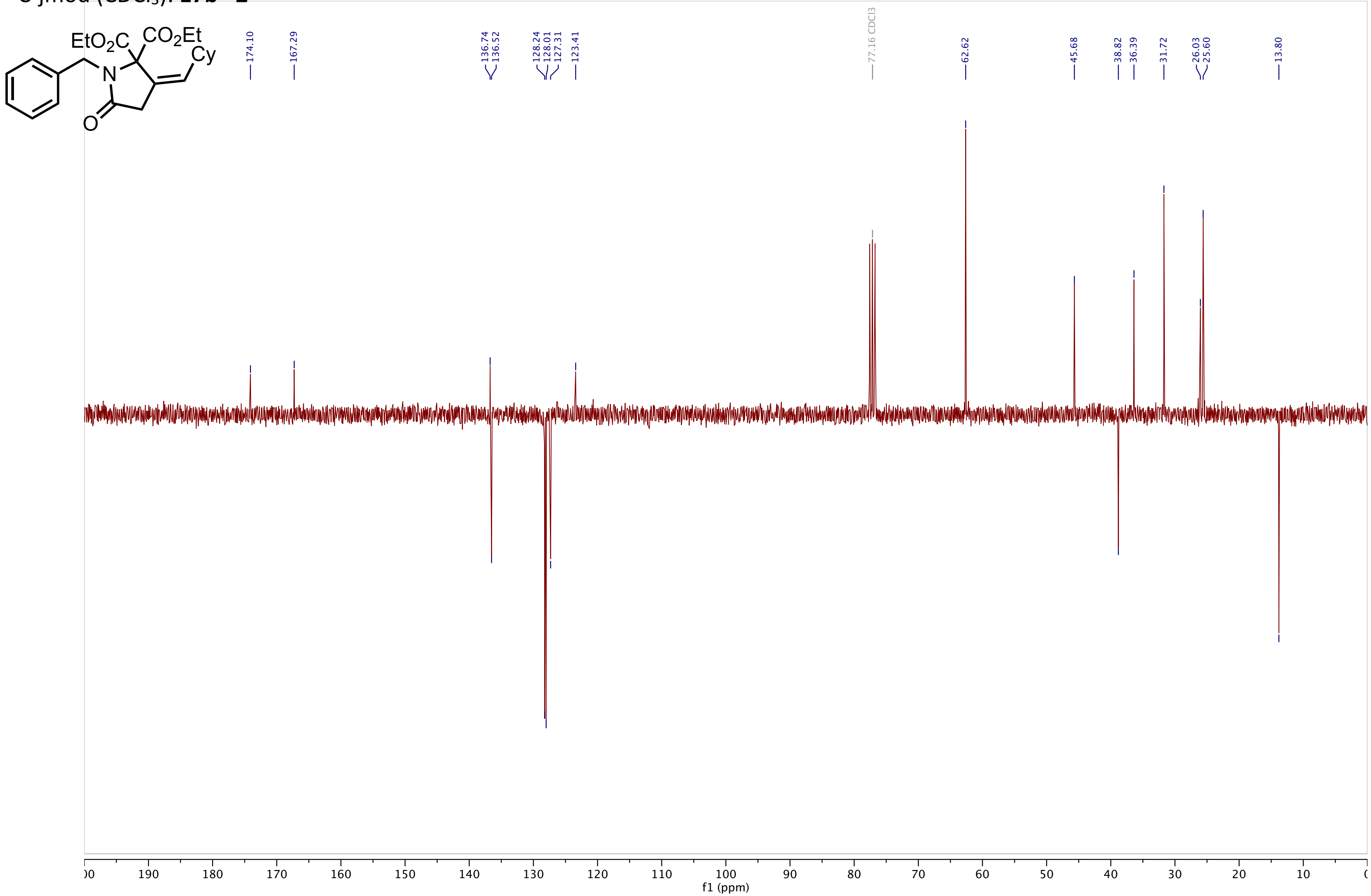

^{13}C-HSQC ($CDCl_3$): **17b - *Z***

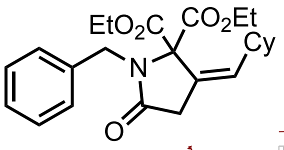

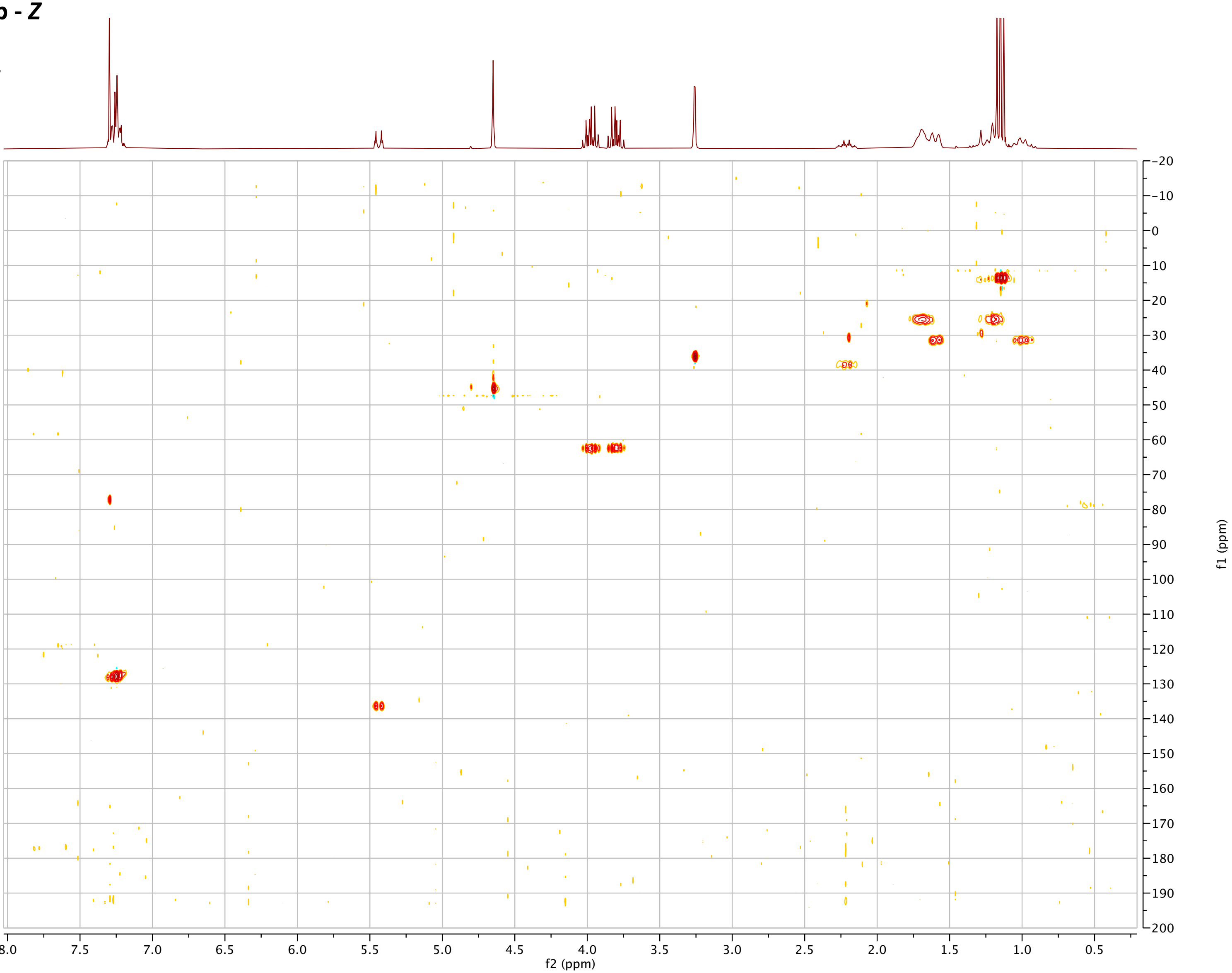

^{13}C-HMBC ($CDCl_3$): **17b - *Z***

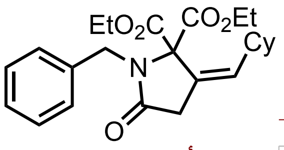

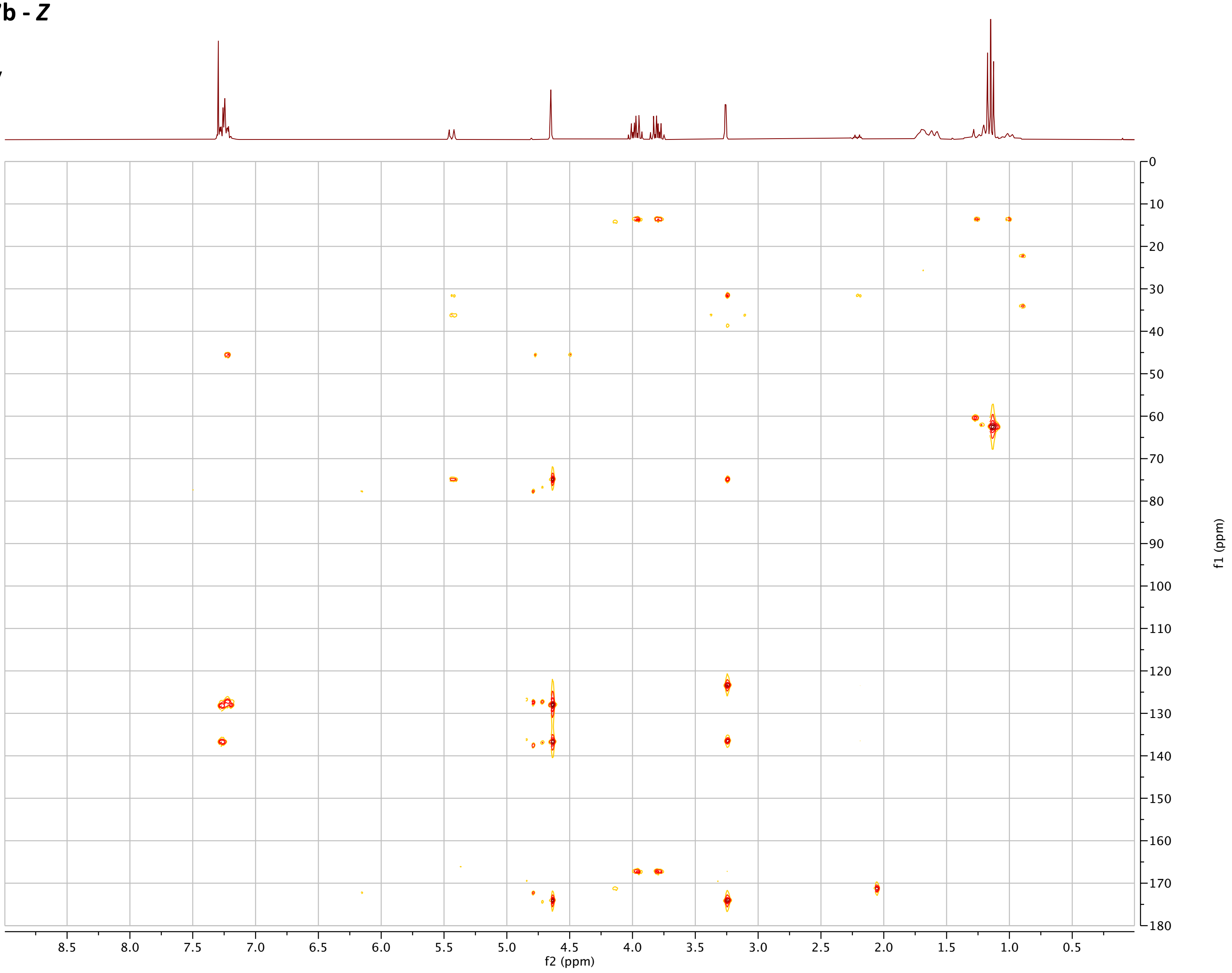

^{13}C-^{13}C ADEQUATE ($CDCl_3$): **17b - *Z***

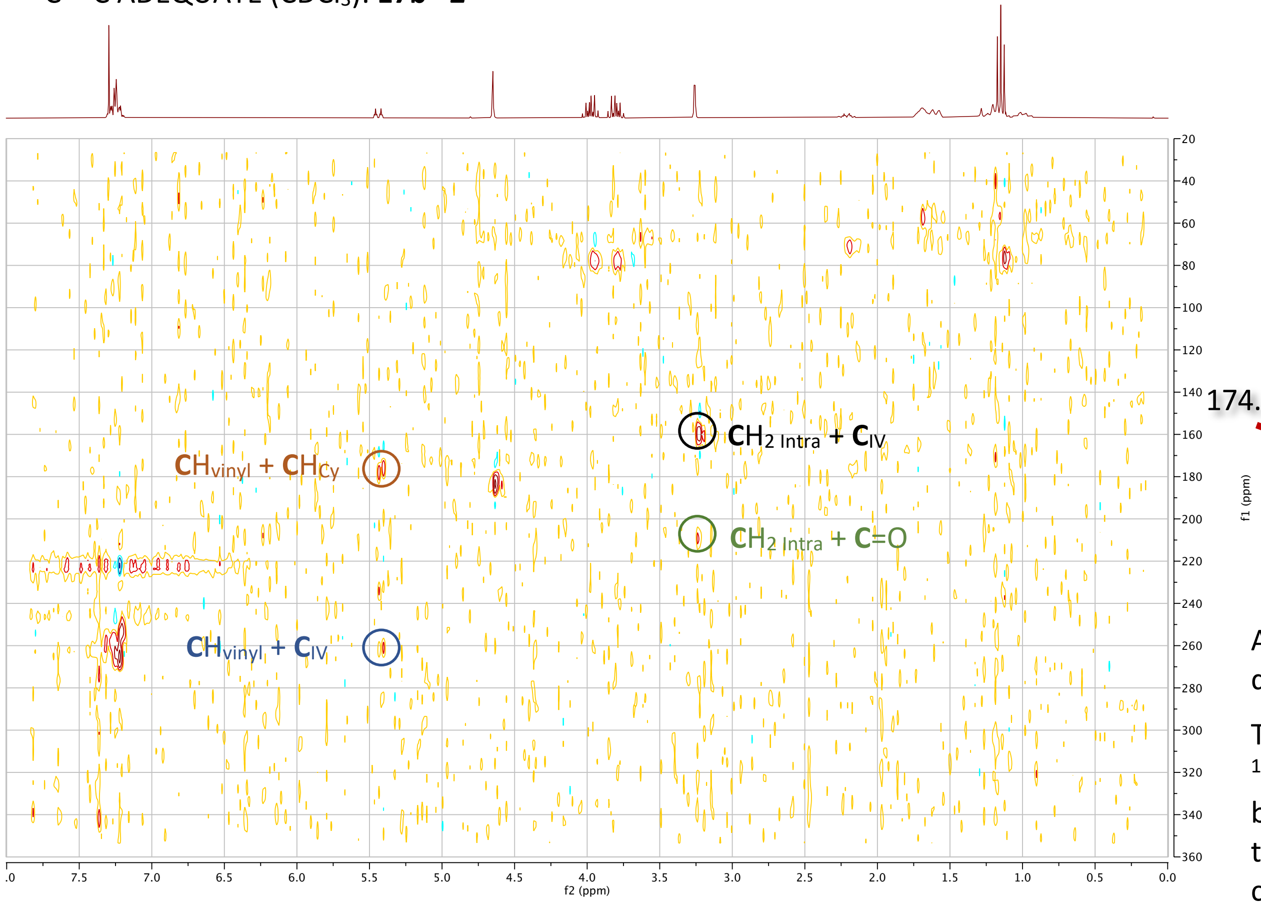

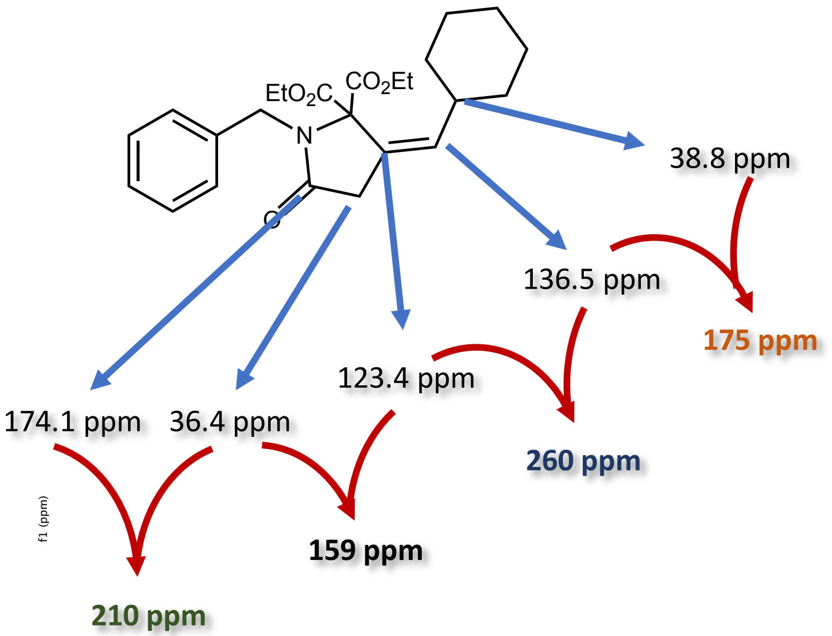

As representative example for the determination of the connectivity:

The CH_2 signal (at 3.22 ppm in ^{1}H; 36.4 ppm in ^{13}C) shows two correlations spots (green and black at 210 ppm and 159 ppm), arising from the two carbons it is connected to. The chemical shift of the spot is determined by the sum of the chemical shift of the CH2 carbon (36.4 ppm) plus the chemical shift of the carbon it is bonded to. This means that the spot at 210 ppm is indicative of a connection with a carbon with a chemical shift of 174 ppm (here the carbonyl) as 174+36=210. The same principle applies for all spots shown here, allowing the unambiguous determination of the structure.

^{1}H-NOESY ($CDCl_3$): **17b - *Z***

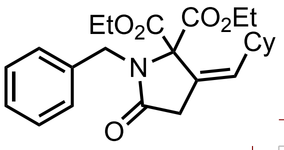

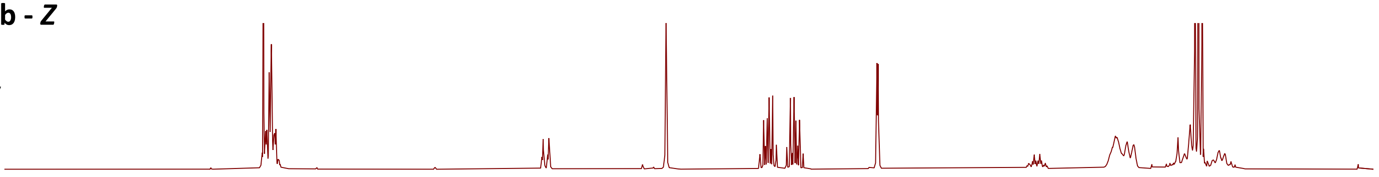

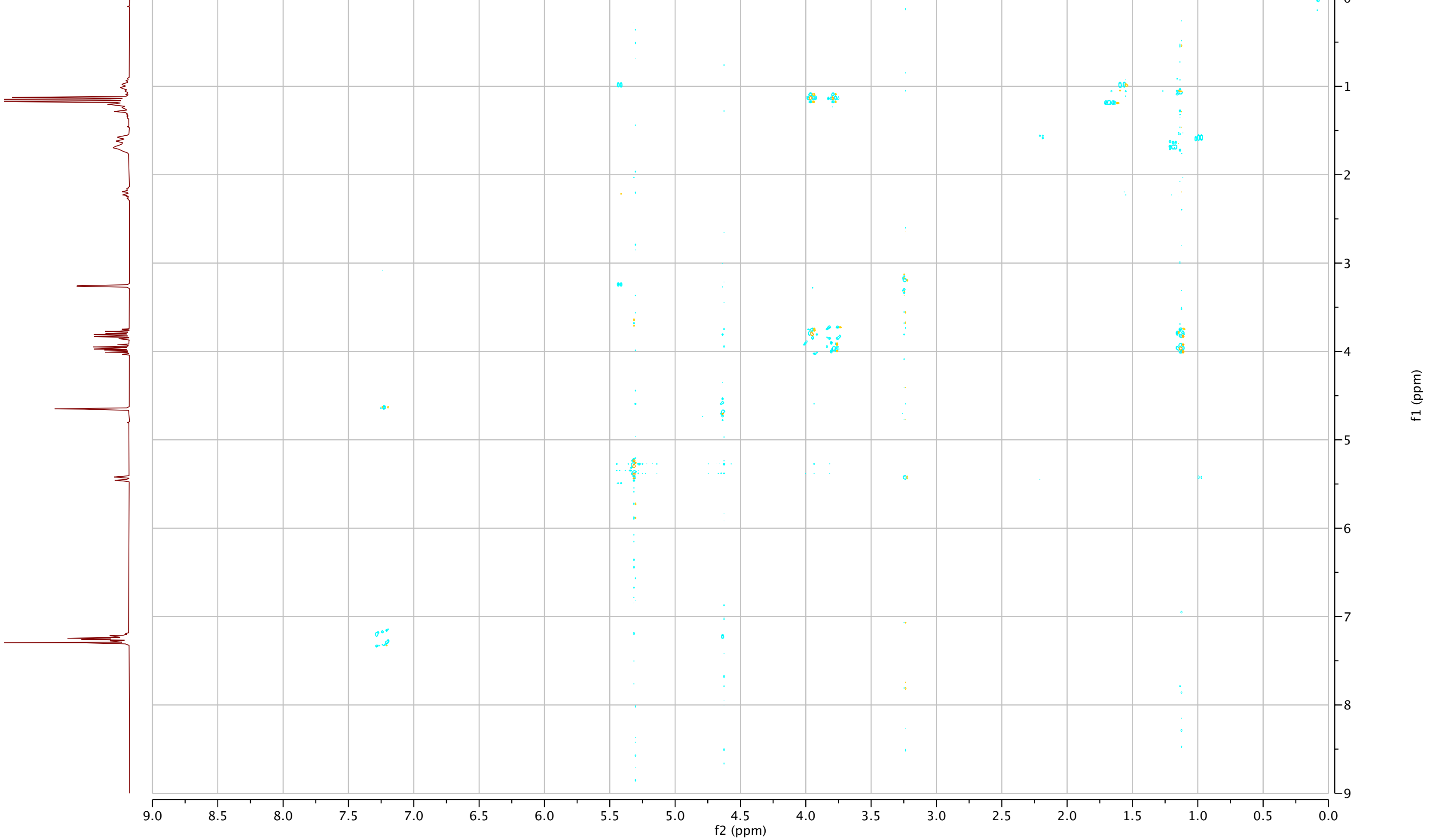

^{1}H-NMR ($CDCl_3$): **17b - *E***

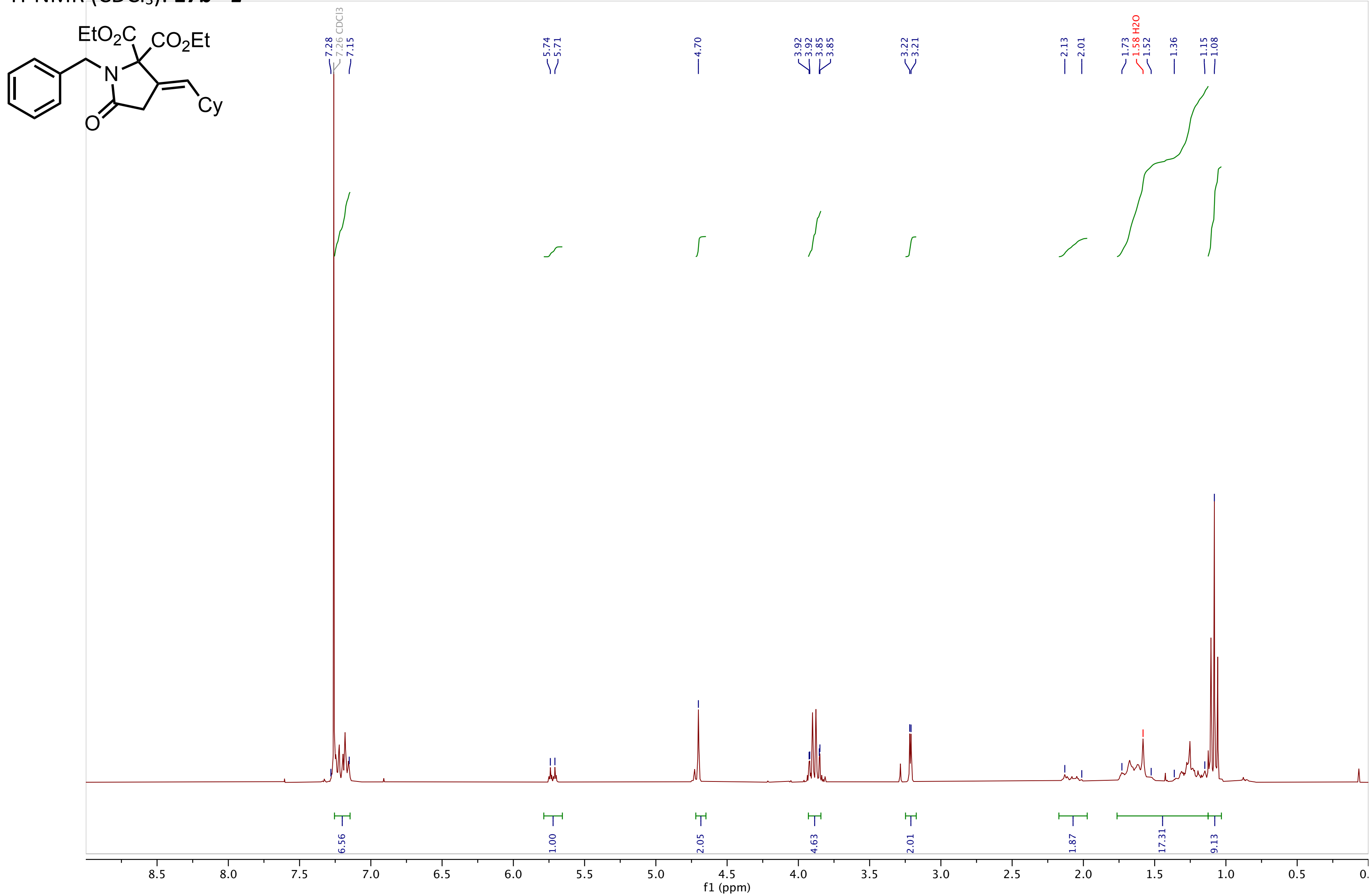

^{13}C-jmod ($CDCl_3$): **17b - *E***

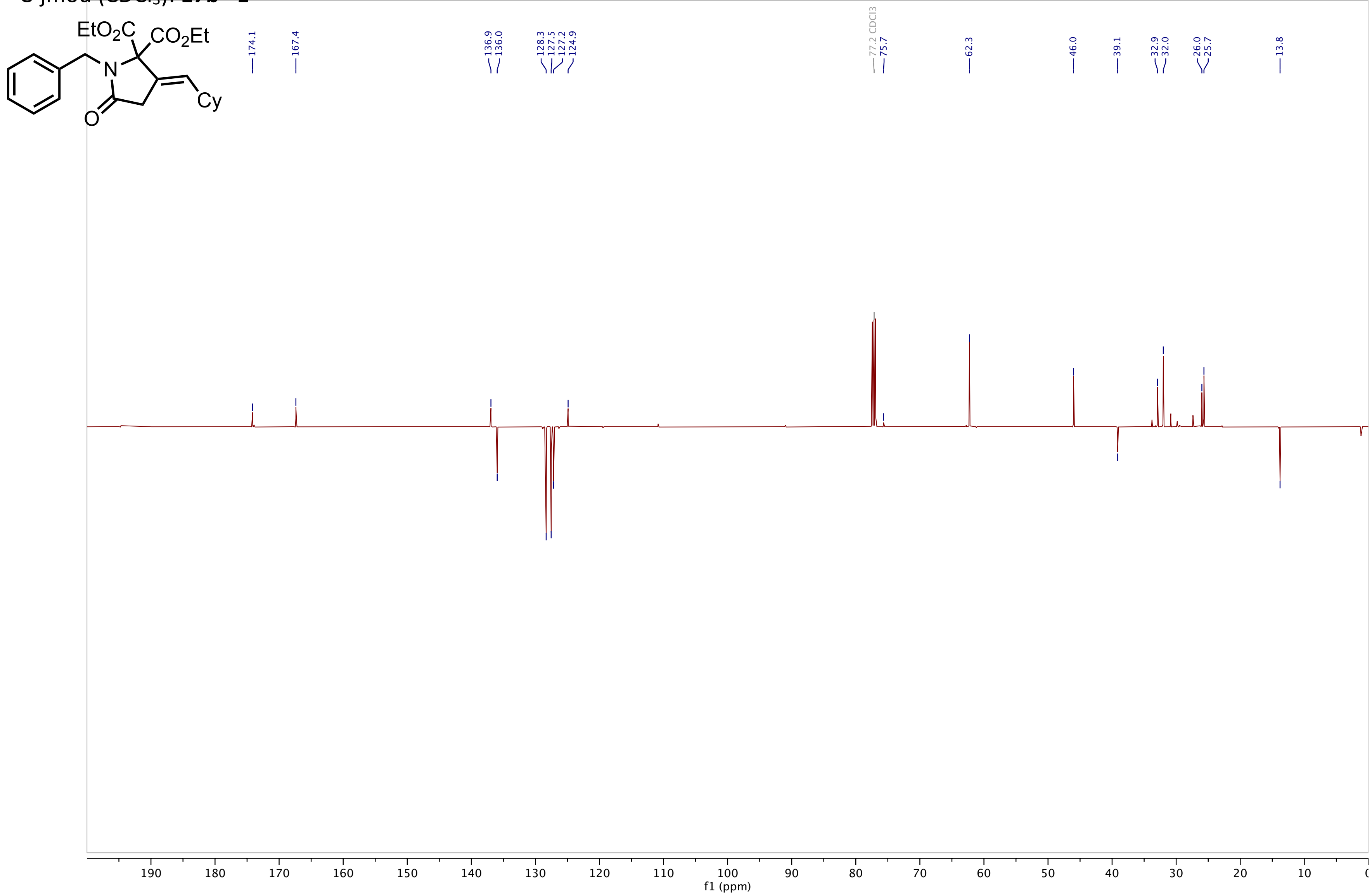

^{13}C-HSQC ($CDCl_3$): **17b - *E***

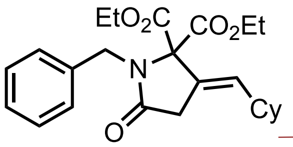

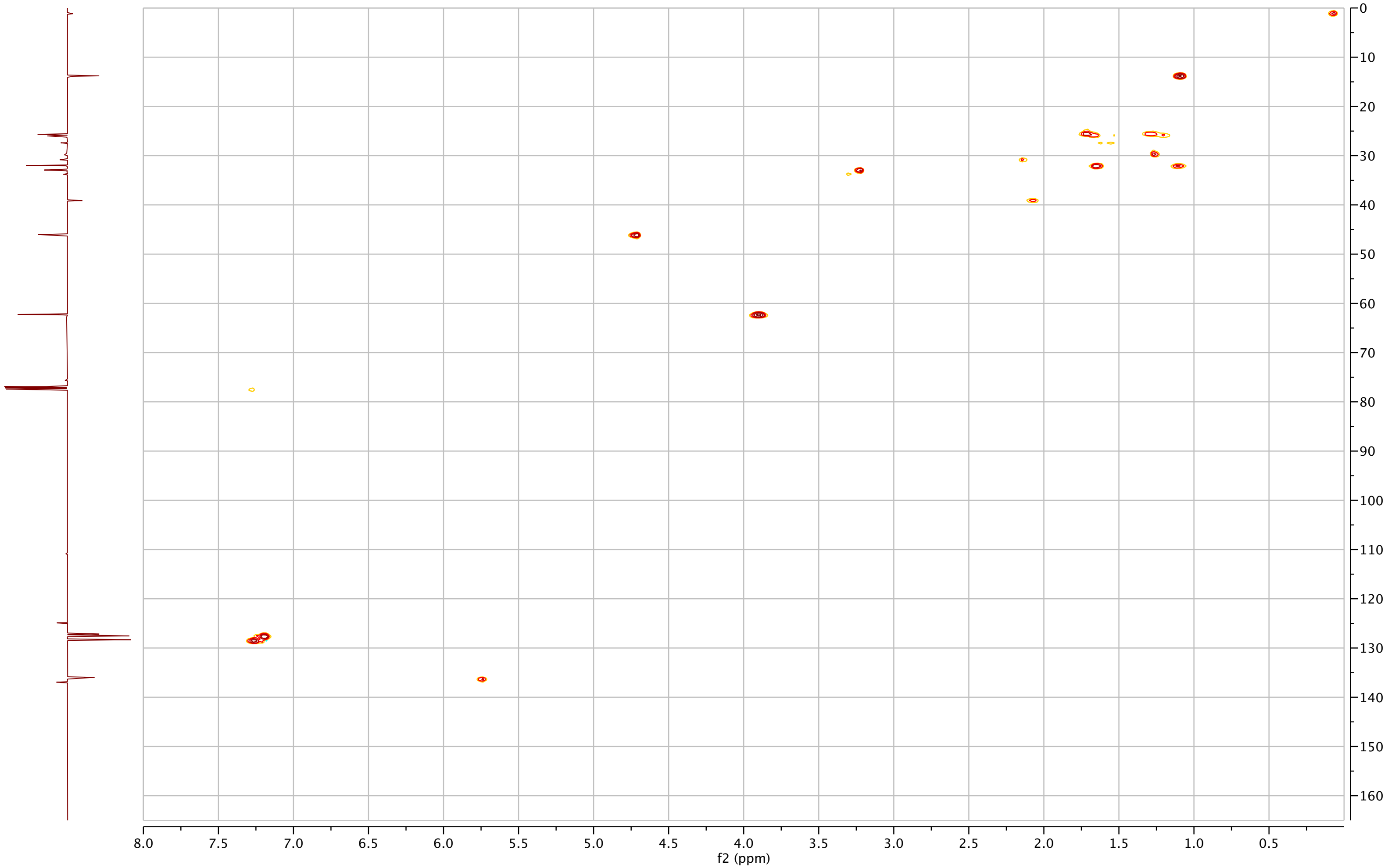

^{13}C-HMBC ($CDCl_3$): **17b - *E***

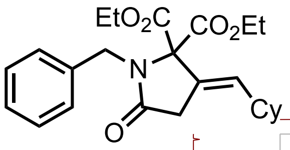

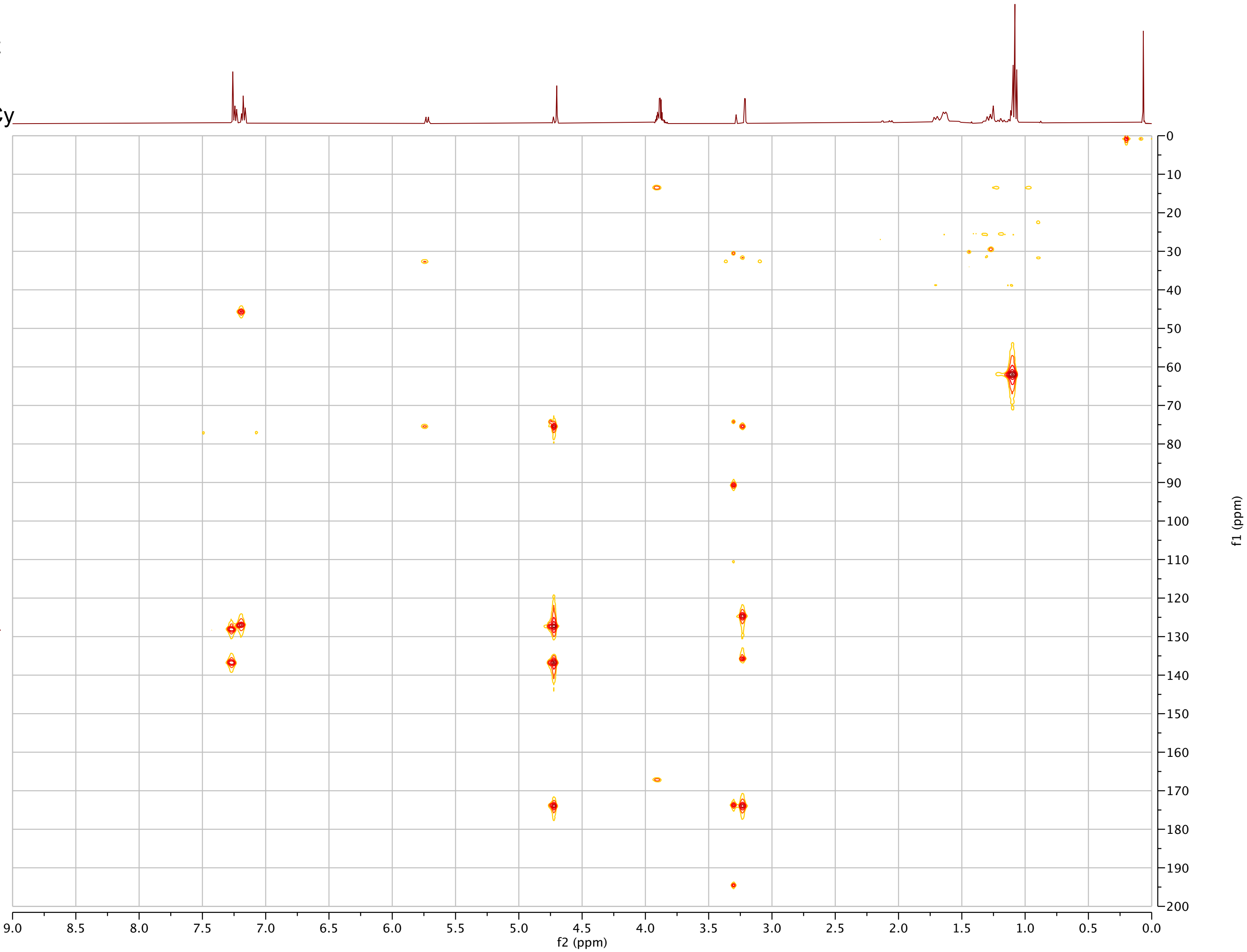

^{1}H-NOESY ($CDCl_3$): **17b - *E***

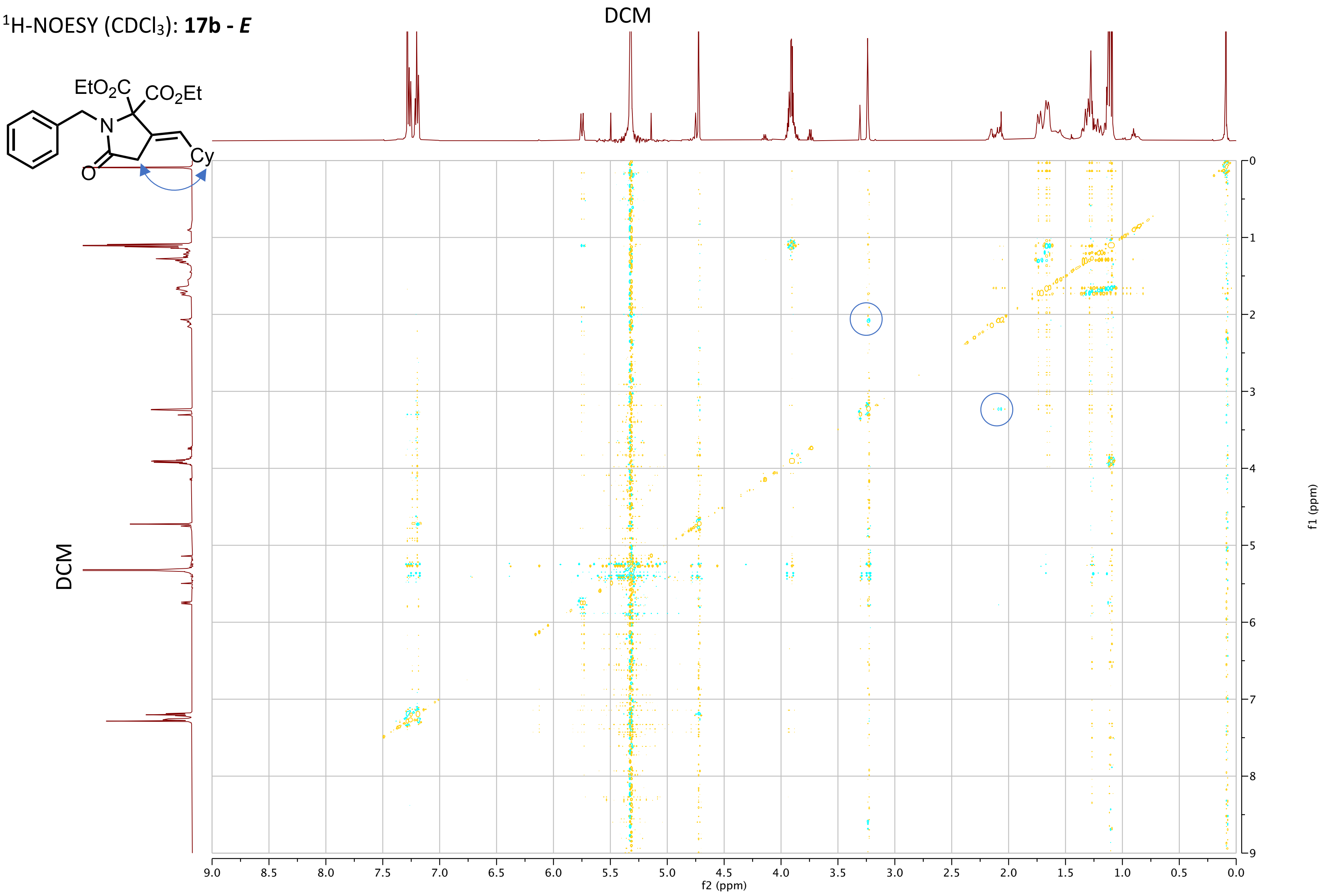

^{1}H-NMR ($CDCl_3$): **18b - *Z***

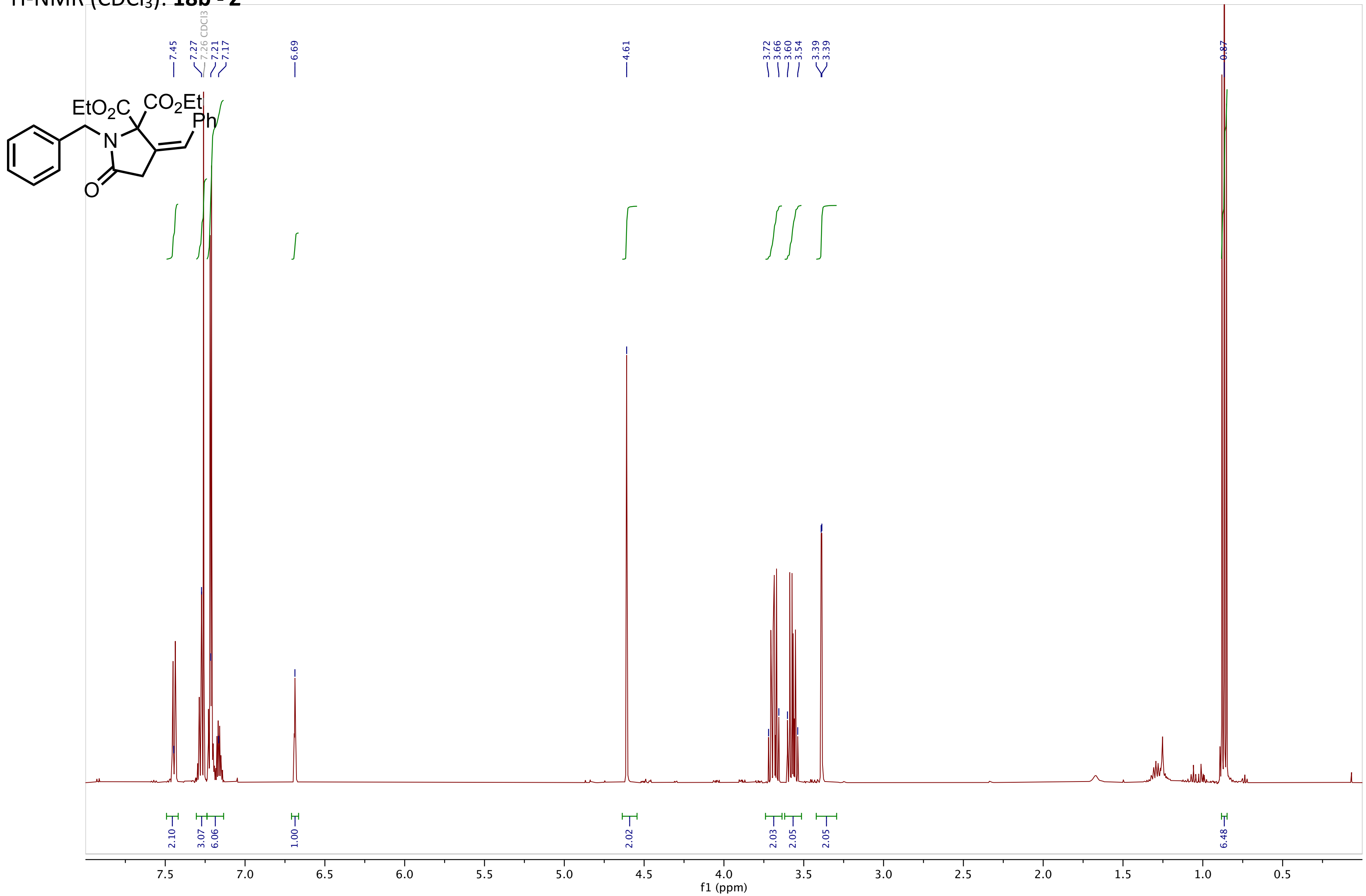

^{13}C-jmod ($CDCl_3$): **18b - *Z***

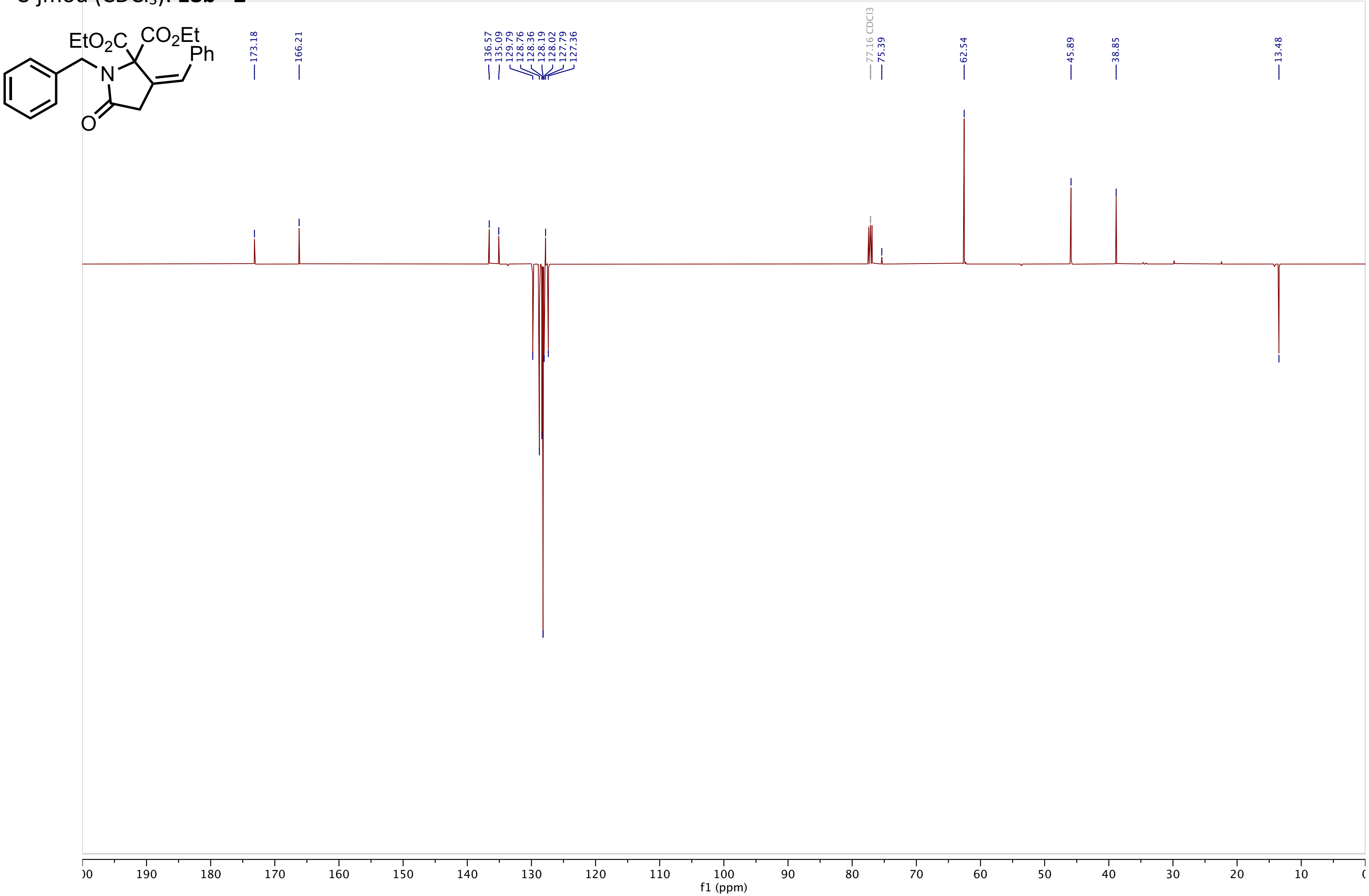

^{13}C-HSQC ($CDCl_3$): **18b - *Z***

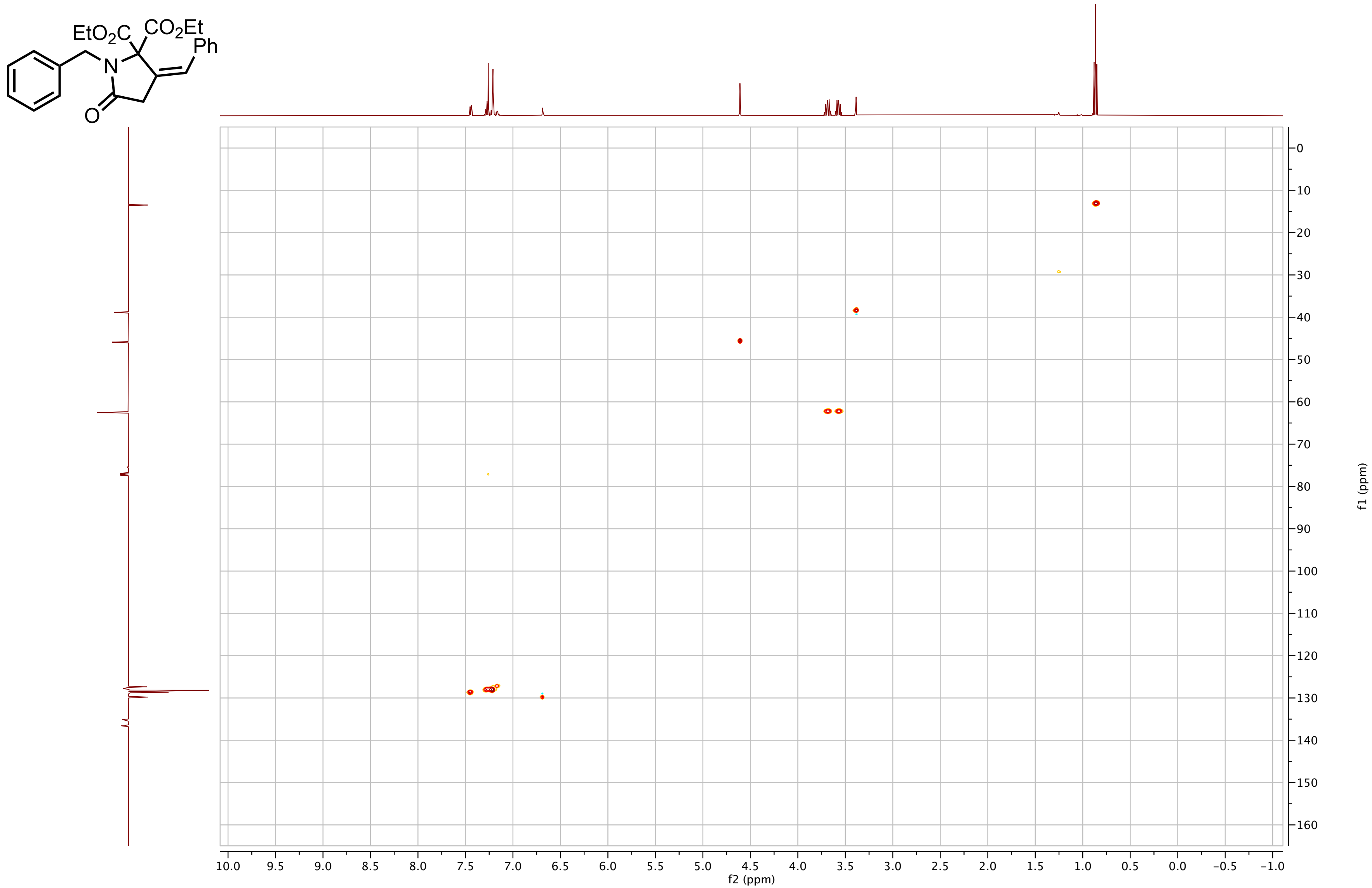

^{13}C-HMBC ($CDCl_3$): **18b - *Z***

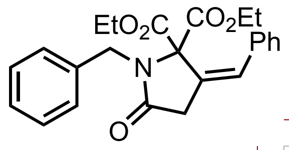

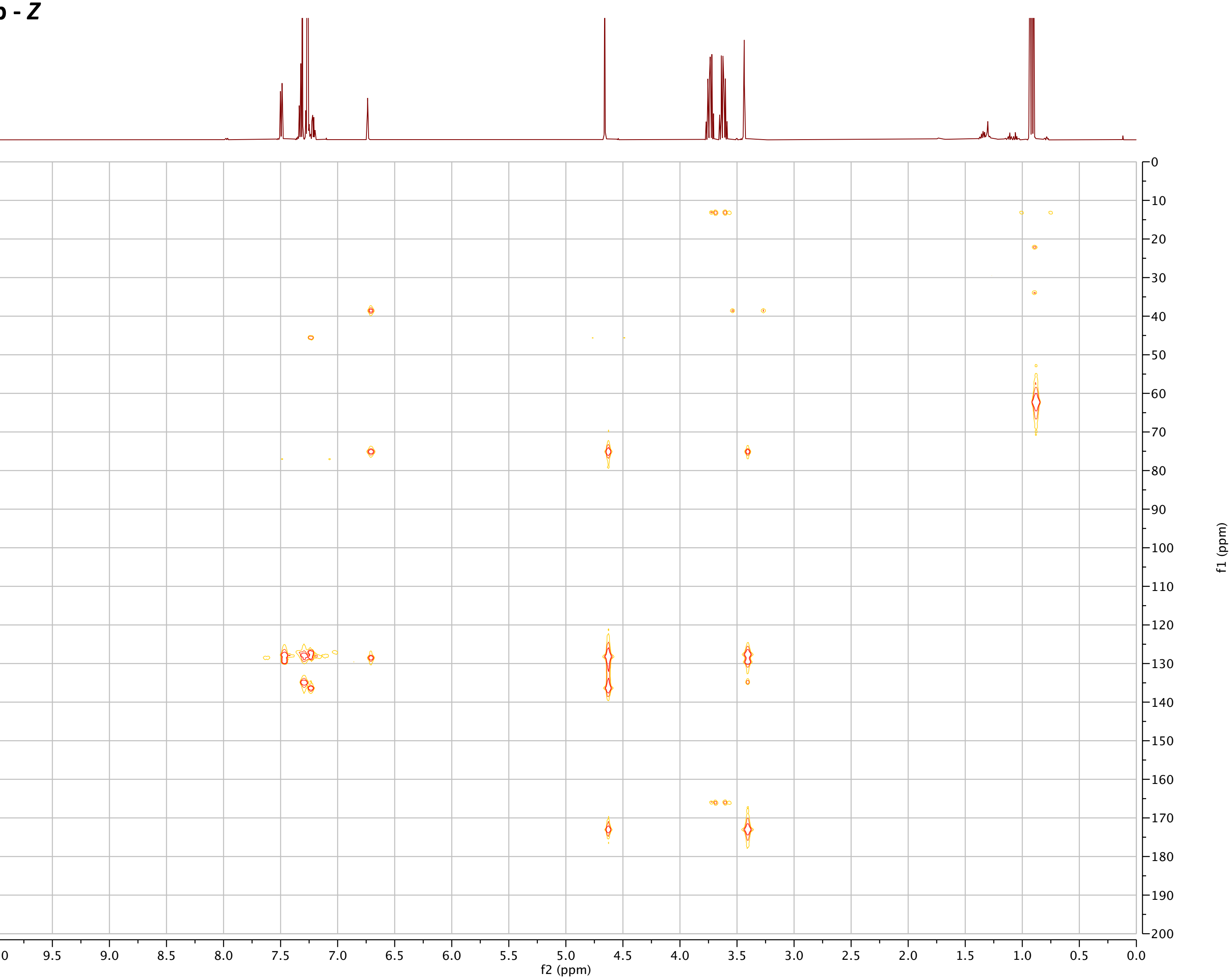

^{1}H-NOESY ($CDCl_3$): **18b - *Z***

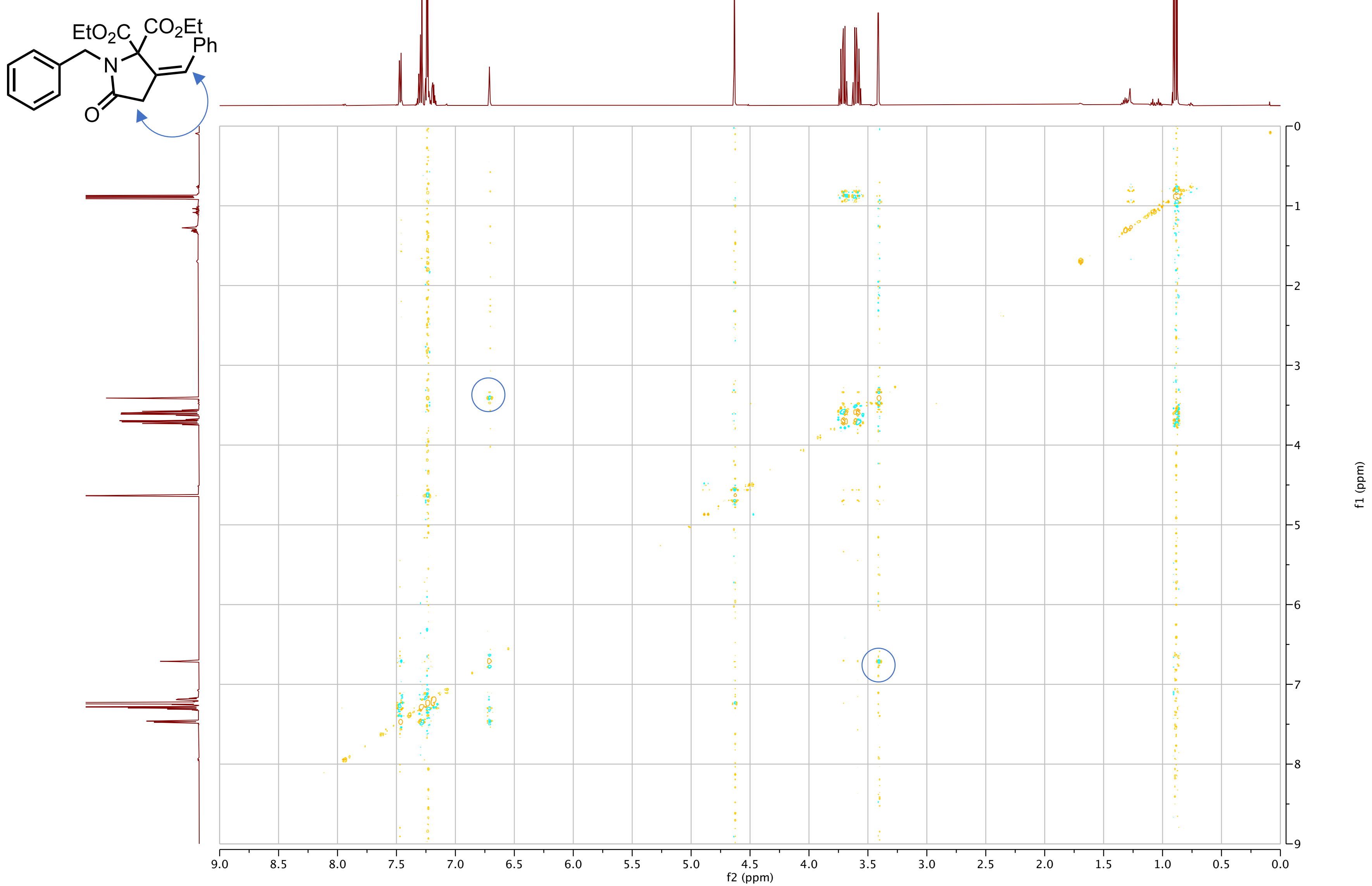

^{1}H-NMR ($CDCl_3$): **18b - *E***

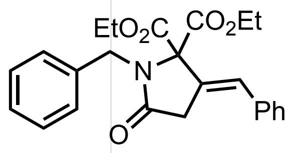

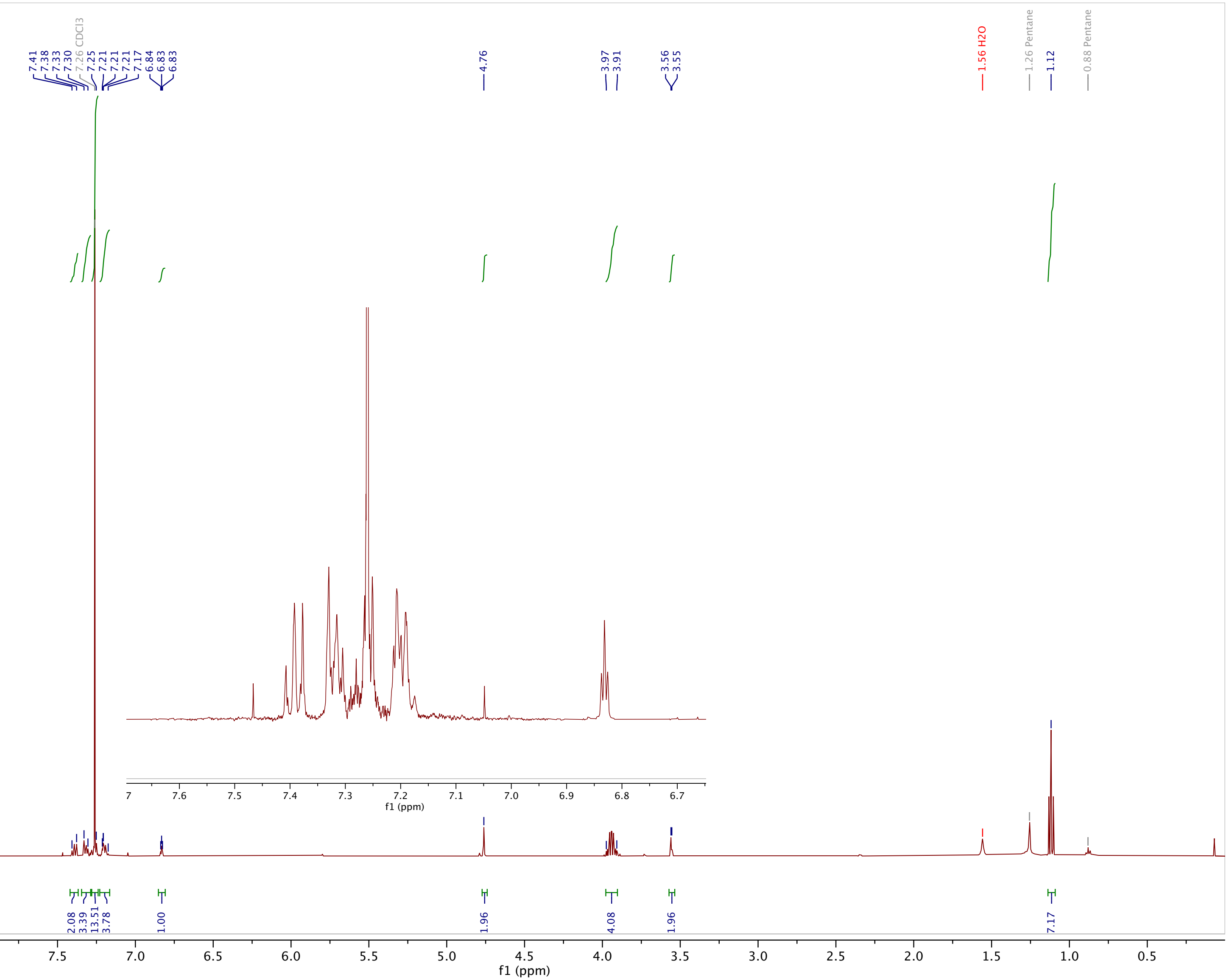

^{13}C-jmod ($CDCl_3$): **18b - *E***

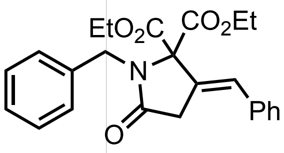

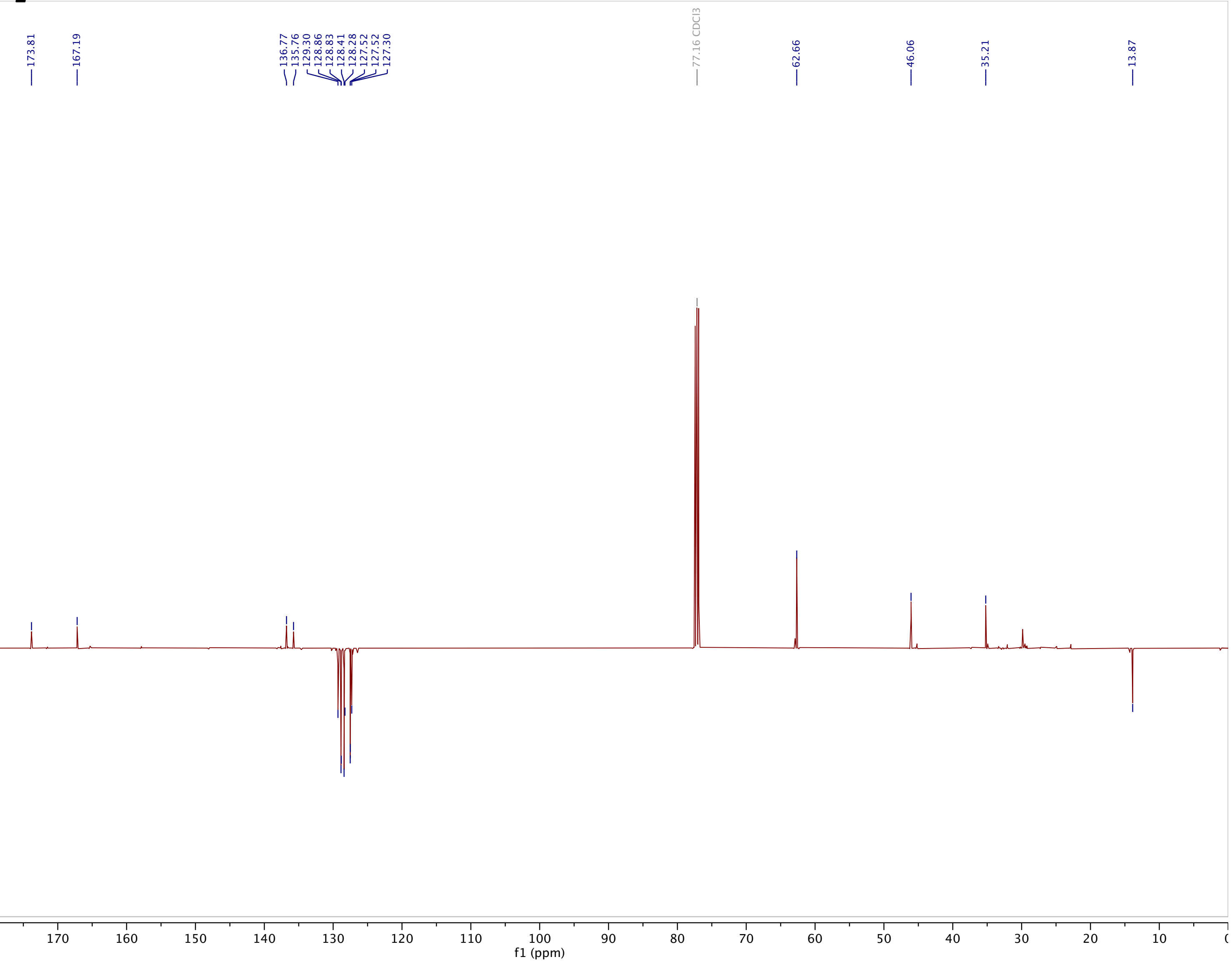

^{13}C-HSQC ($CDCl_3$): **18b - *E***

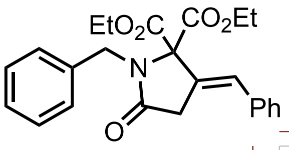

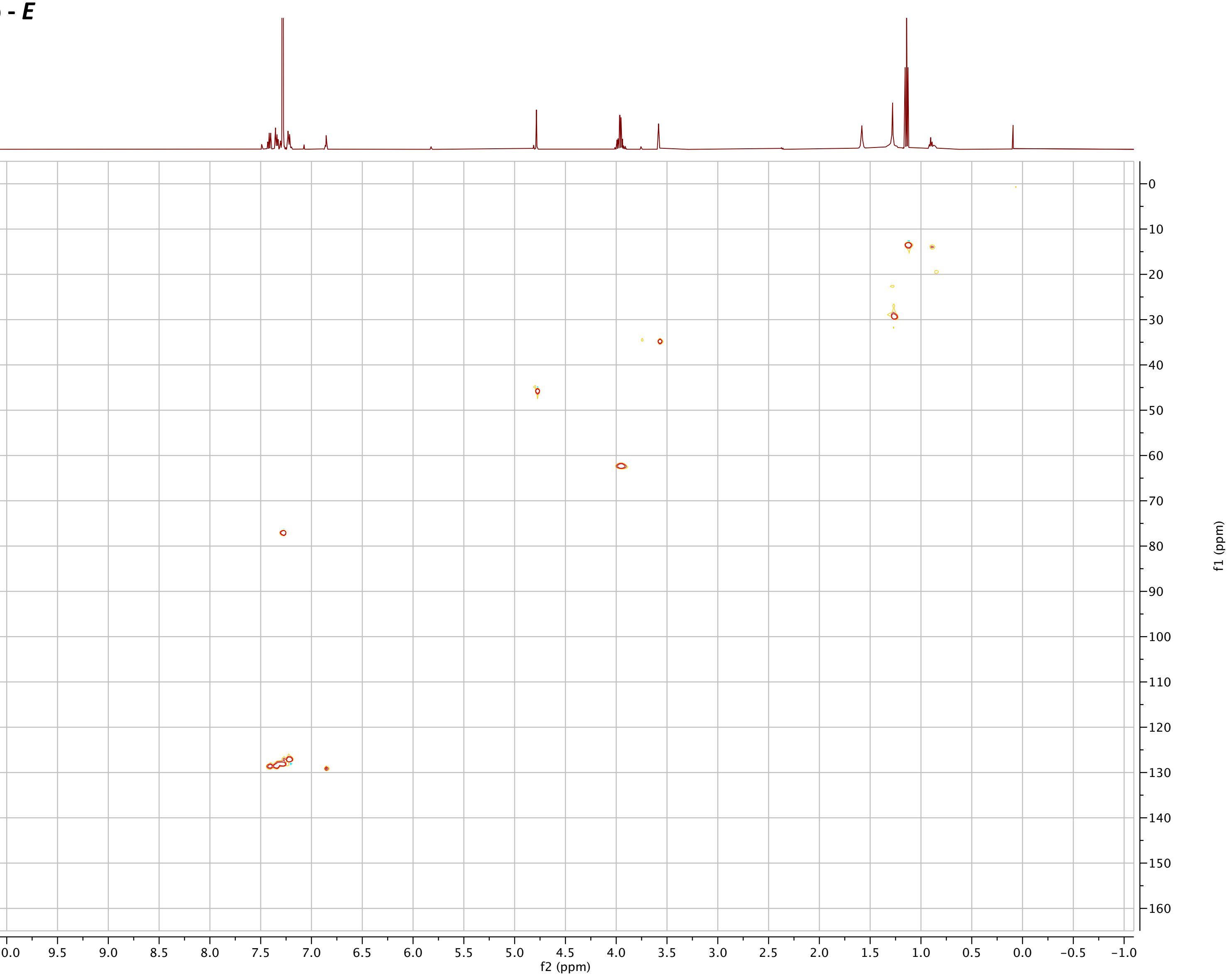

^{13}C-HMBC ($CDCl_3$): **18b - *E***

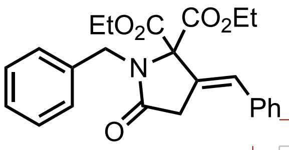

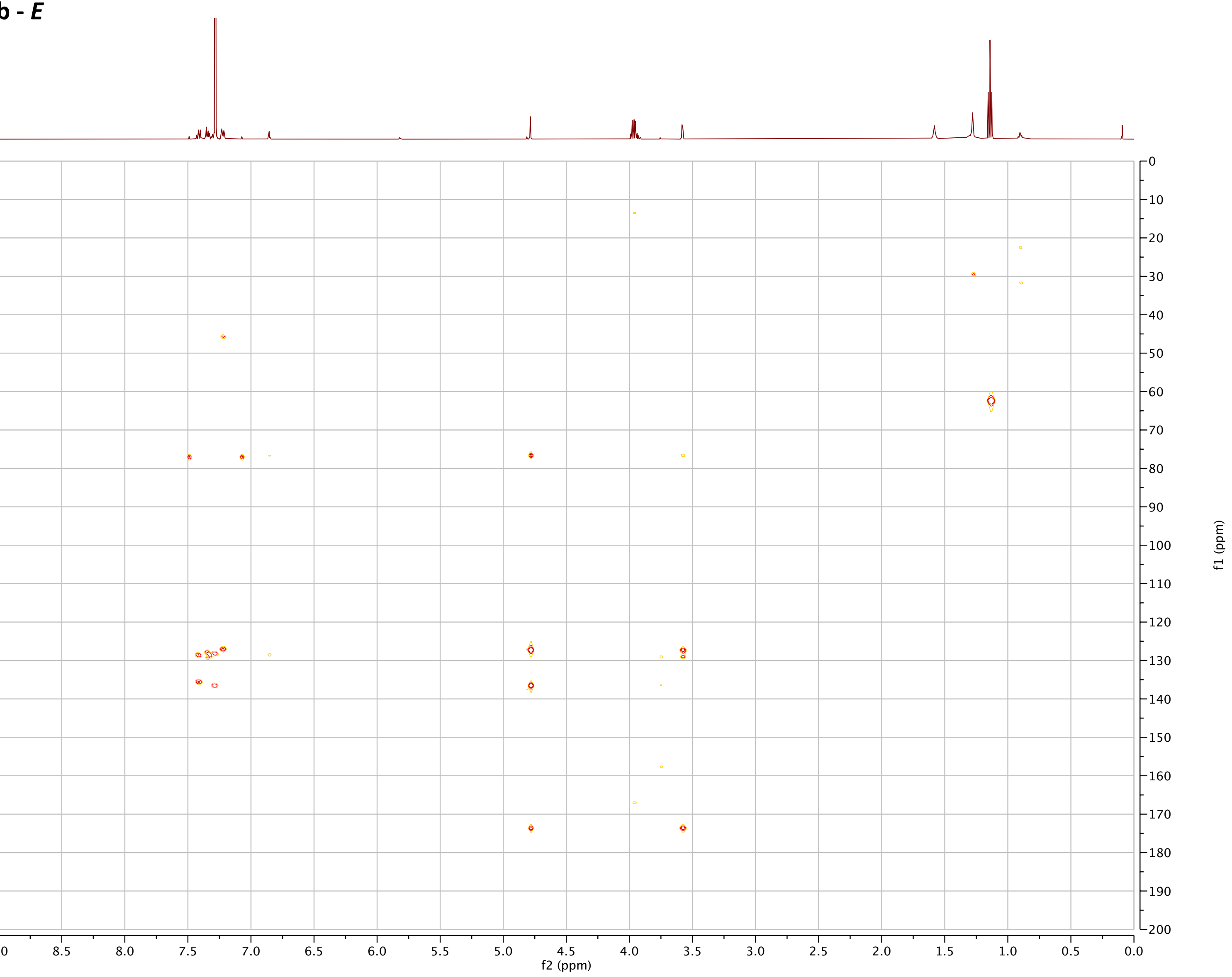

^{1}H-NOESY ($CDCl_3$): **18b - *E***

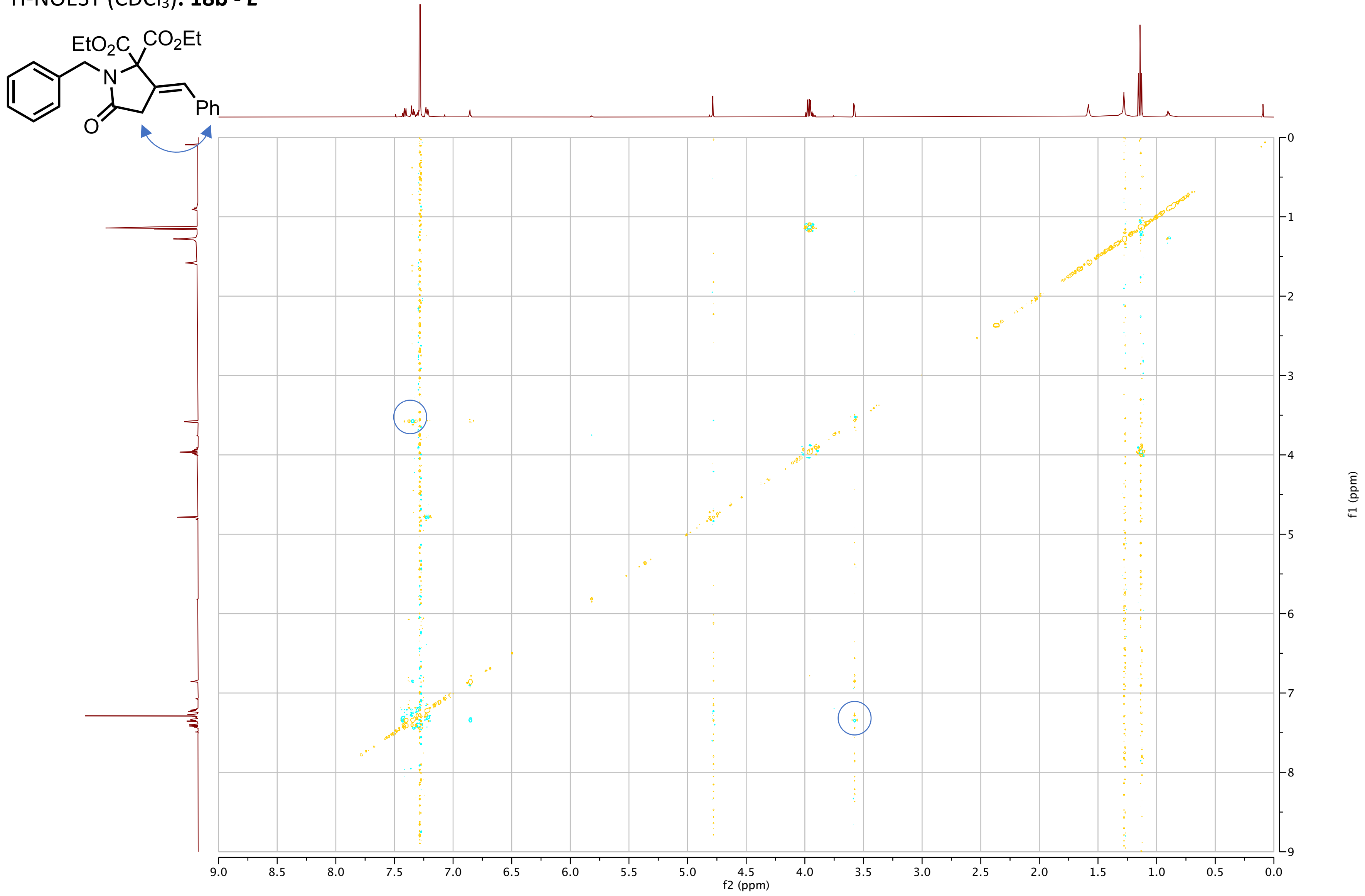

^{1}H-NMR ($CDCl_3$): **19b - *E***

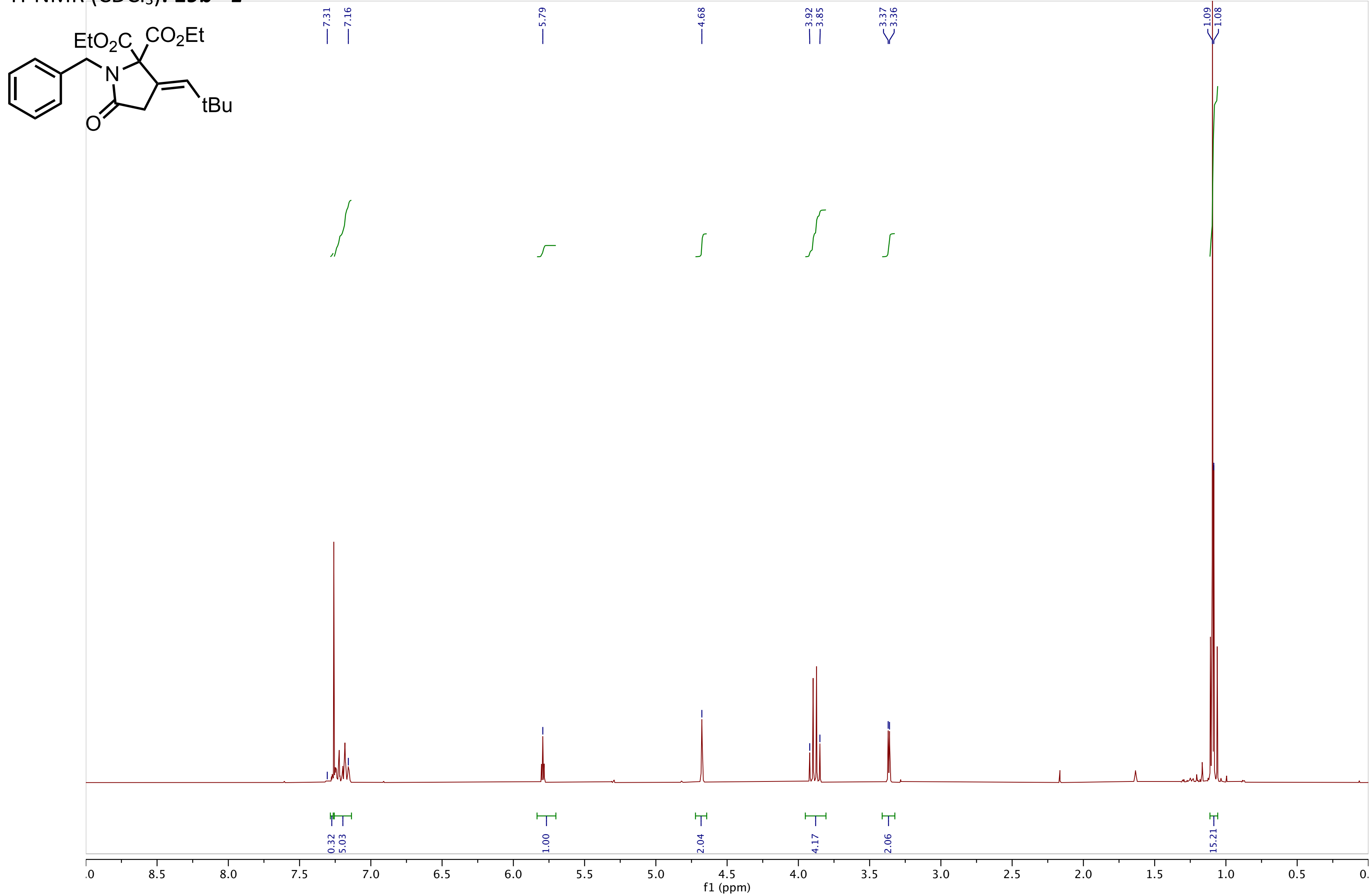

^{13}C-zgpg ($CDCl_3$): **19b - *E***

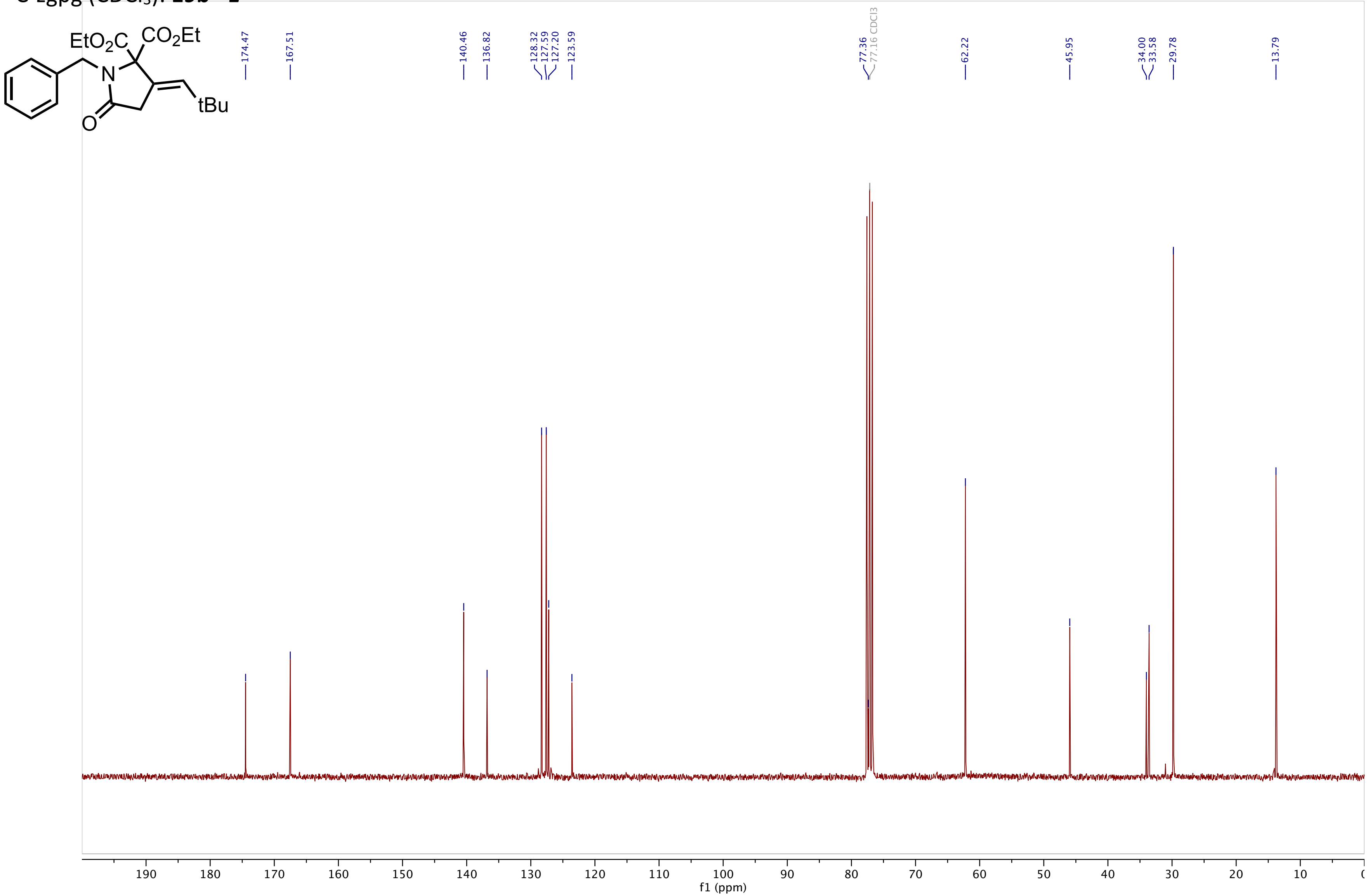

^{13}C-HSQC ($CDCl_3$): **19b - *E***

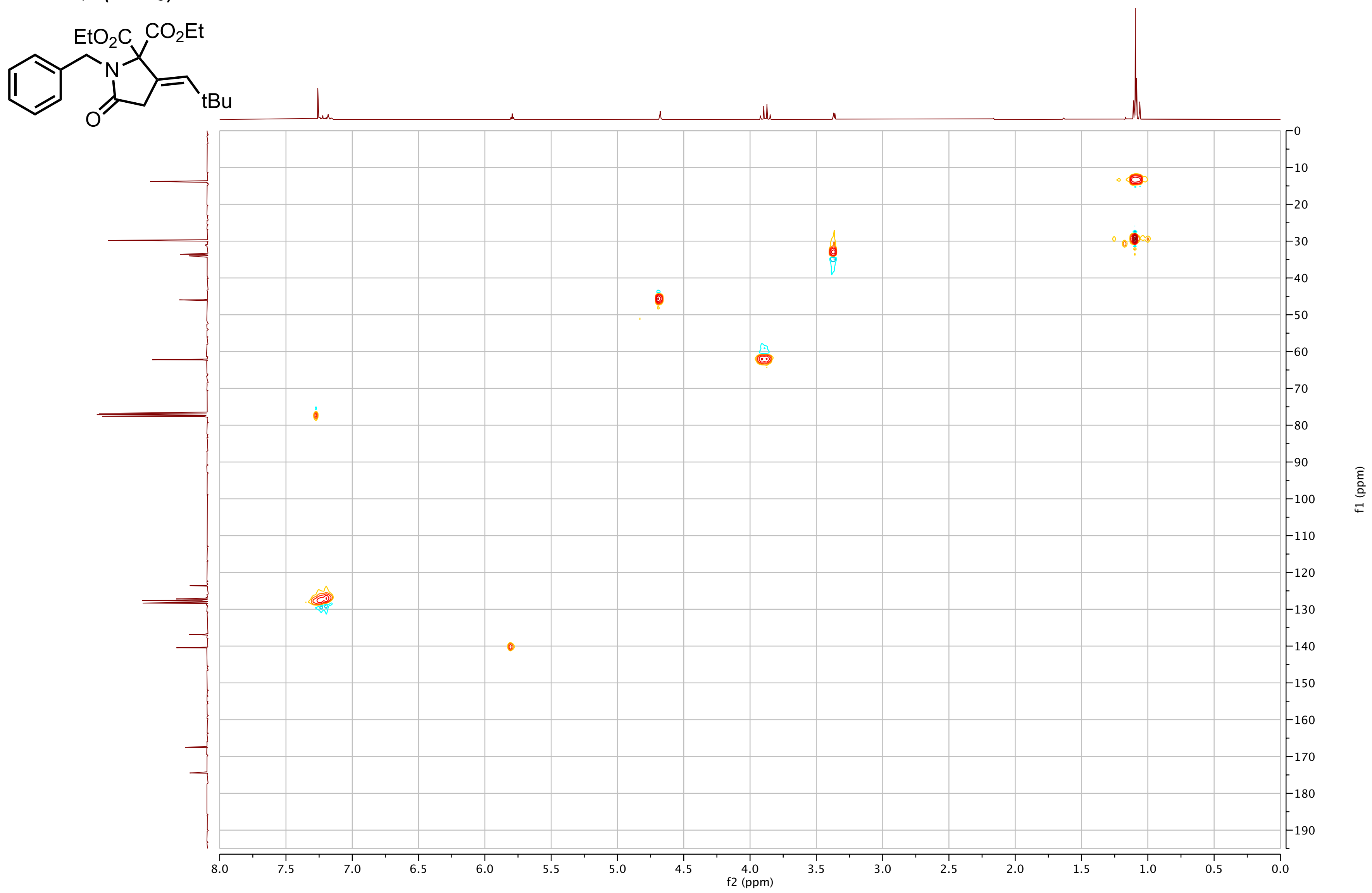

^{13}C-HMBC ($CDCl_3$): **19b - *E***

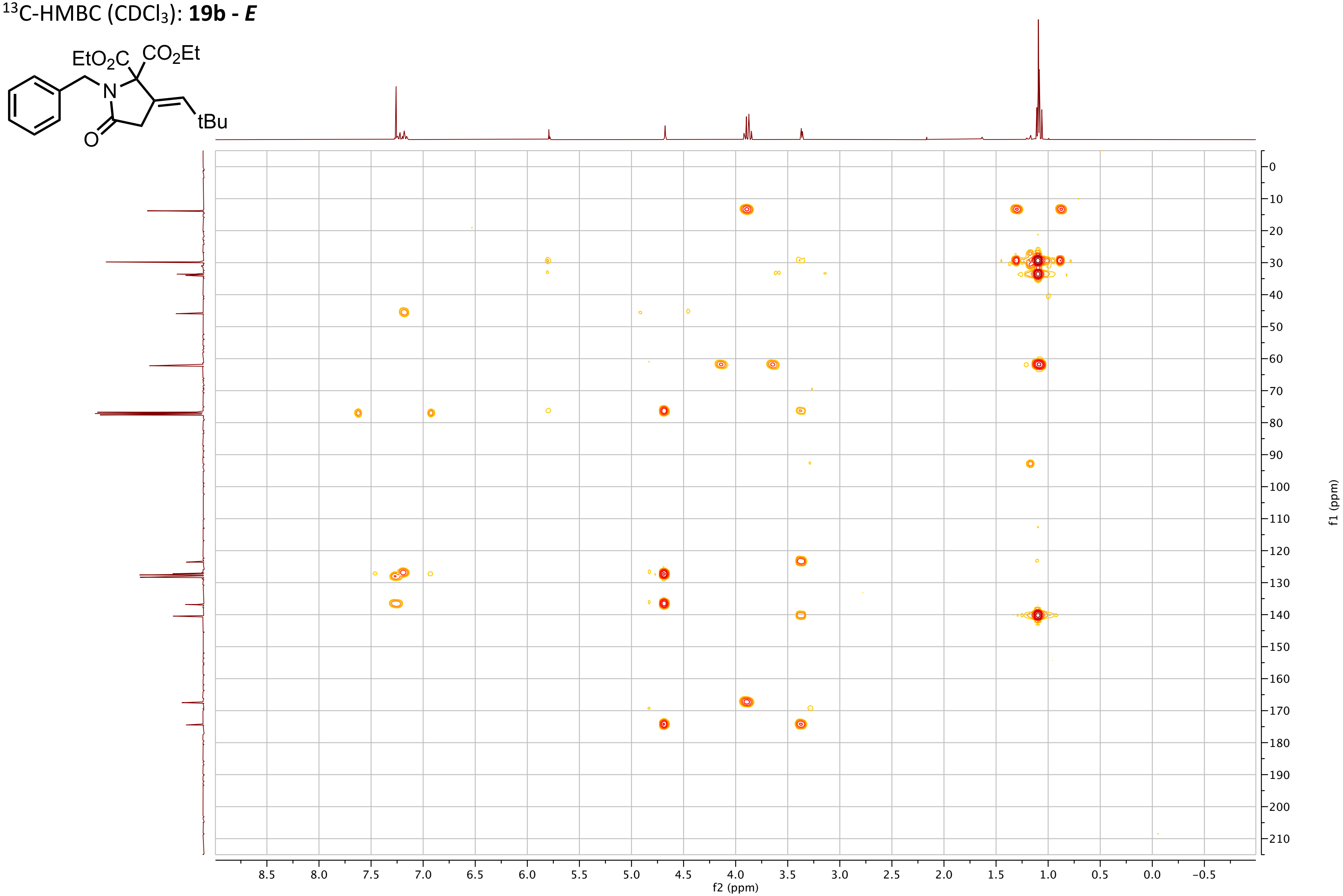

^{1}H-NOESY ($CDCl_3$): **19b - *E***

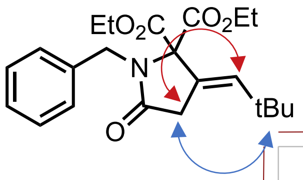

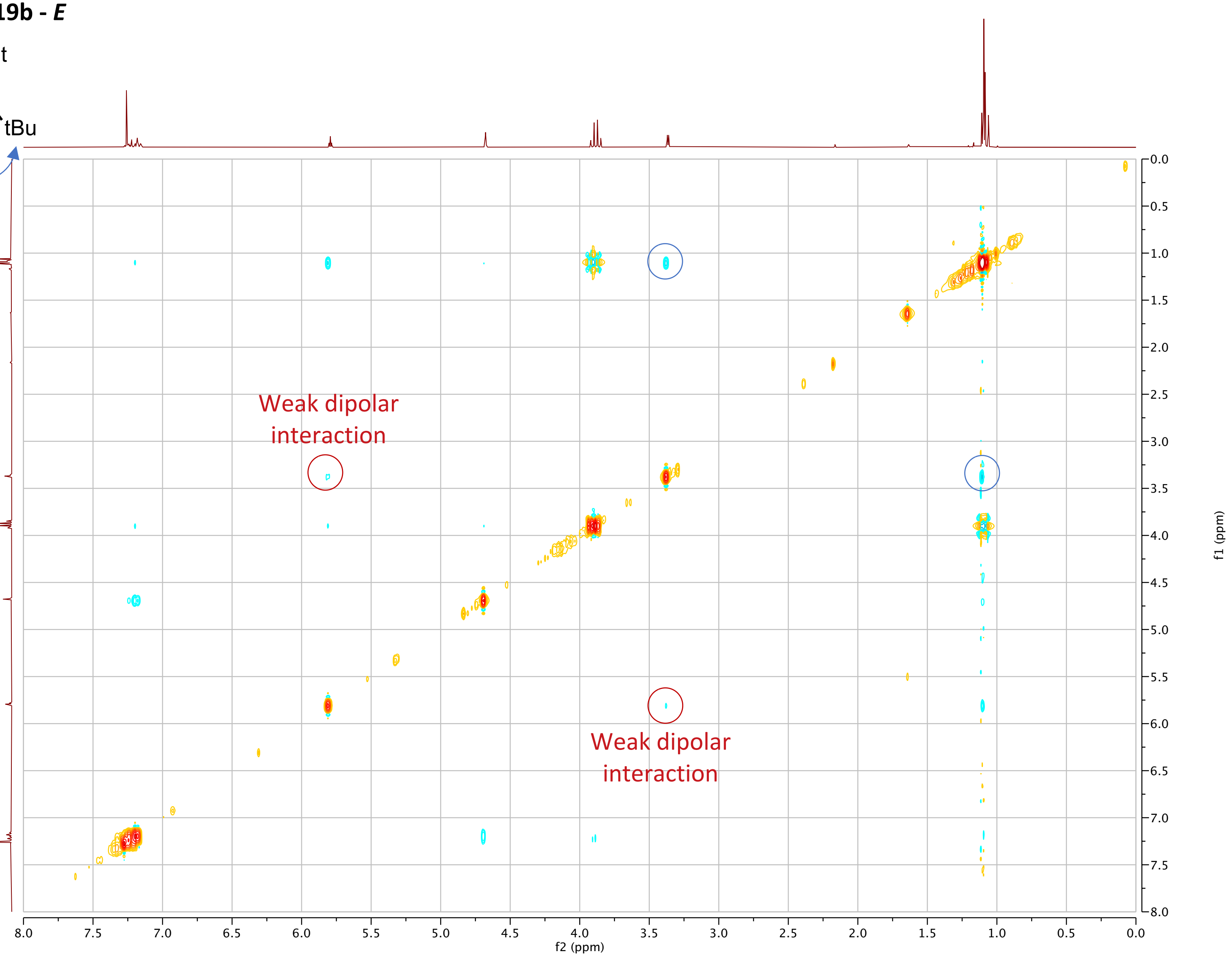

1D [1]H Selective ZQF Noesy ($CDCl_3$): **19b – *E***. Ethylenic proton was selectively excited and the mixing time was 1 s.

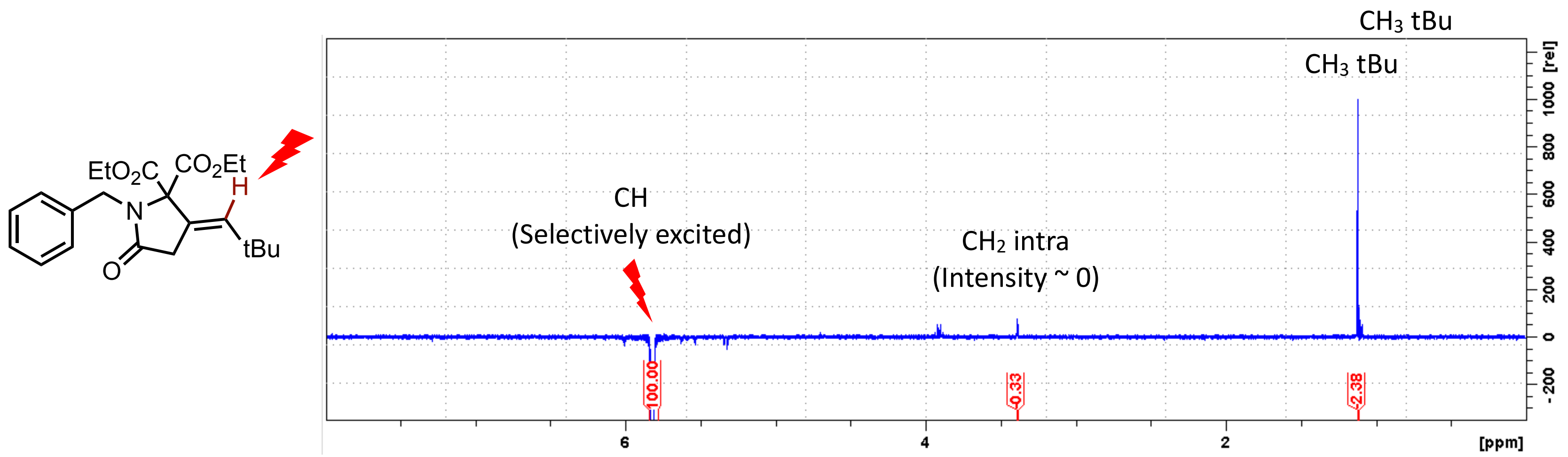

1D [1]H Selective ZQF Noesy ($CDCl_3$): **19b – *E***. CH_2 intra protons were selectively excited and the mixing time was 1 s.

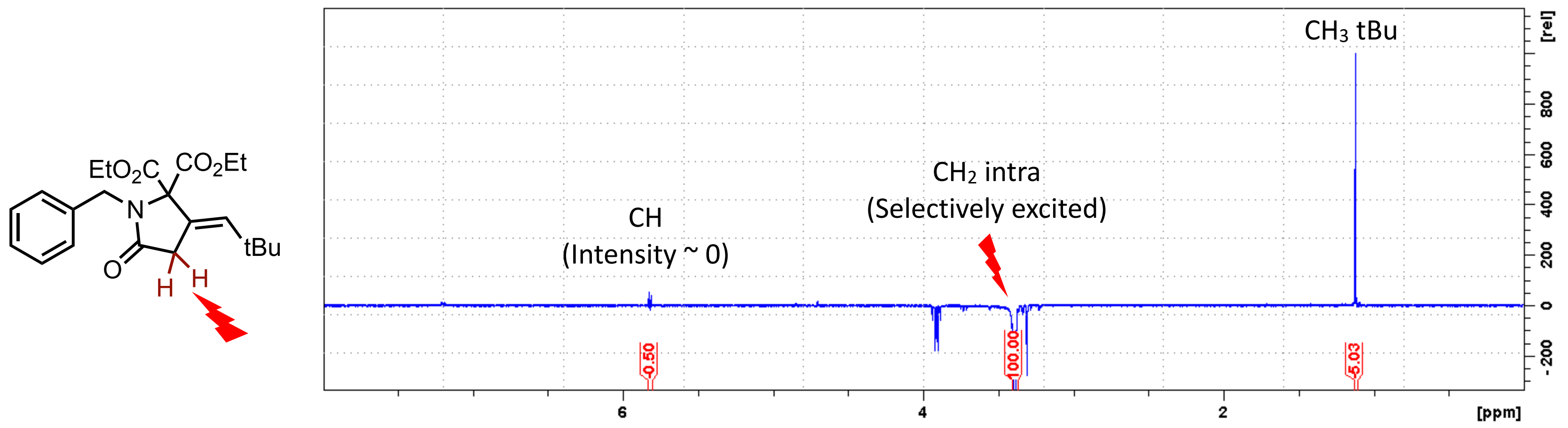

^{13}C-^{13}C ADEQUATE ($CDCl_3$): **19b - *E***

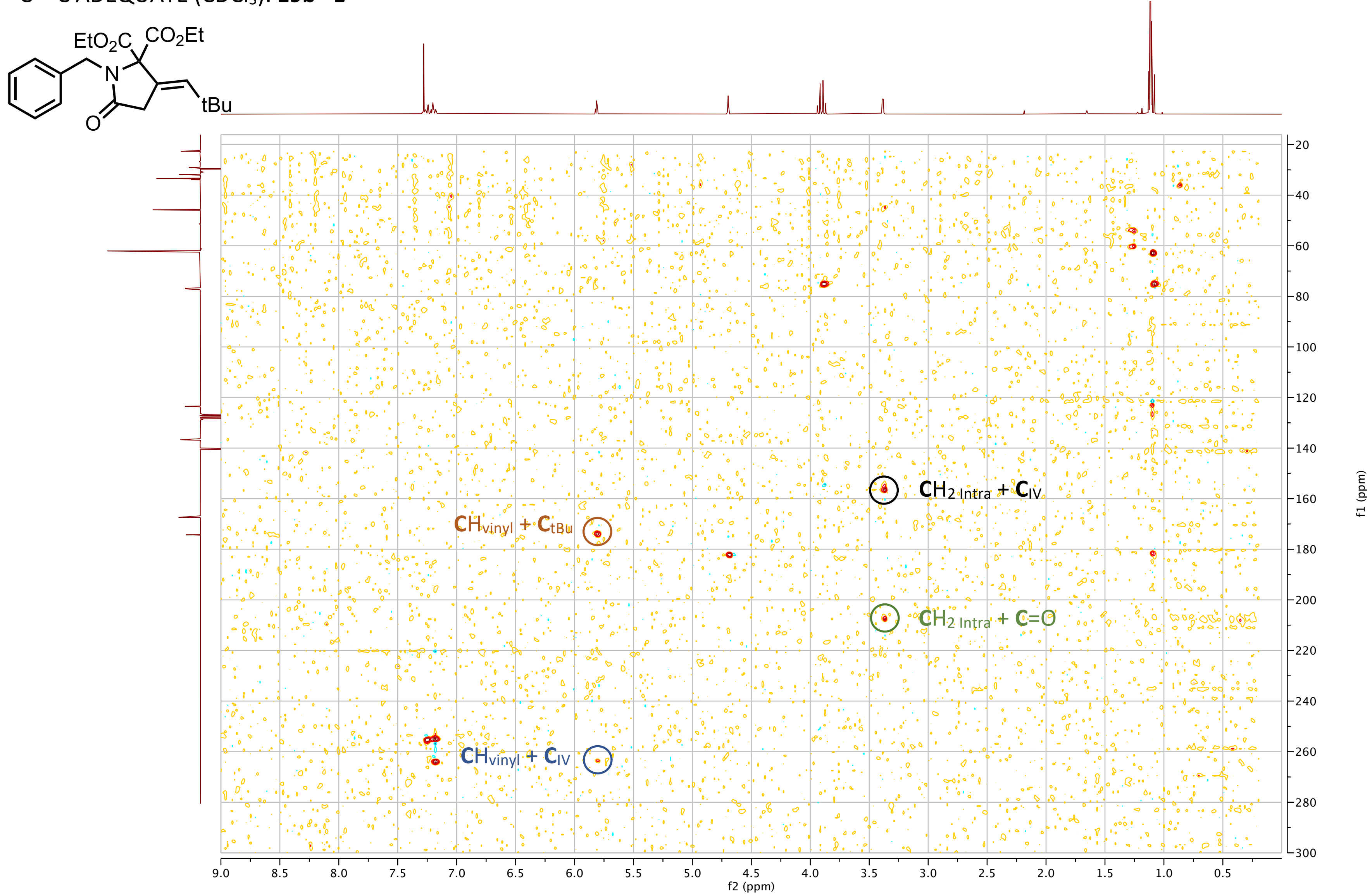

^{1}H-NMR ($CDCl_3$): **19b - *Z***

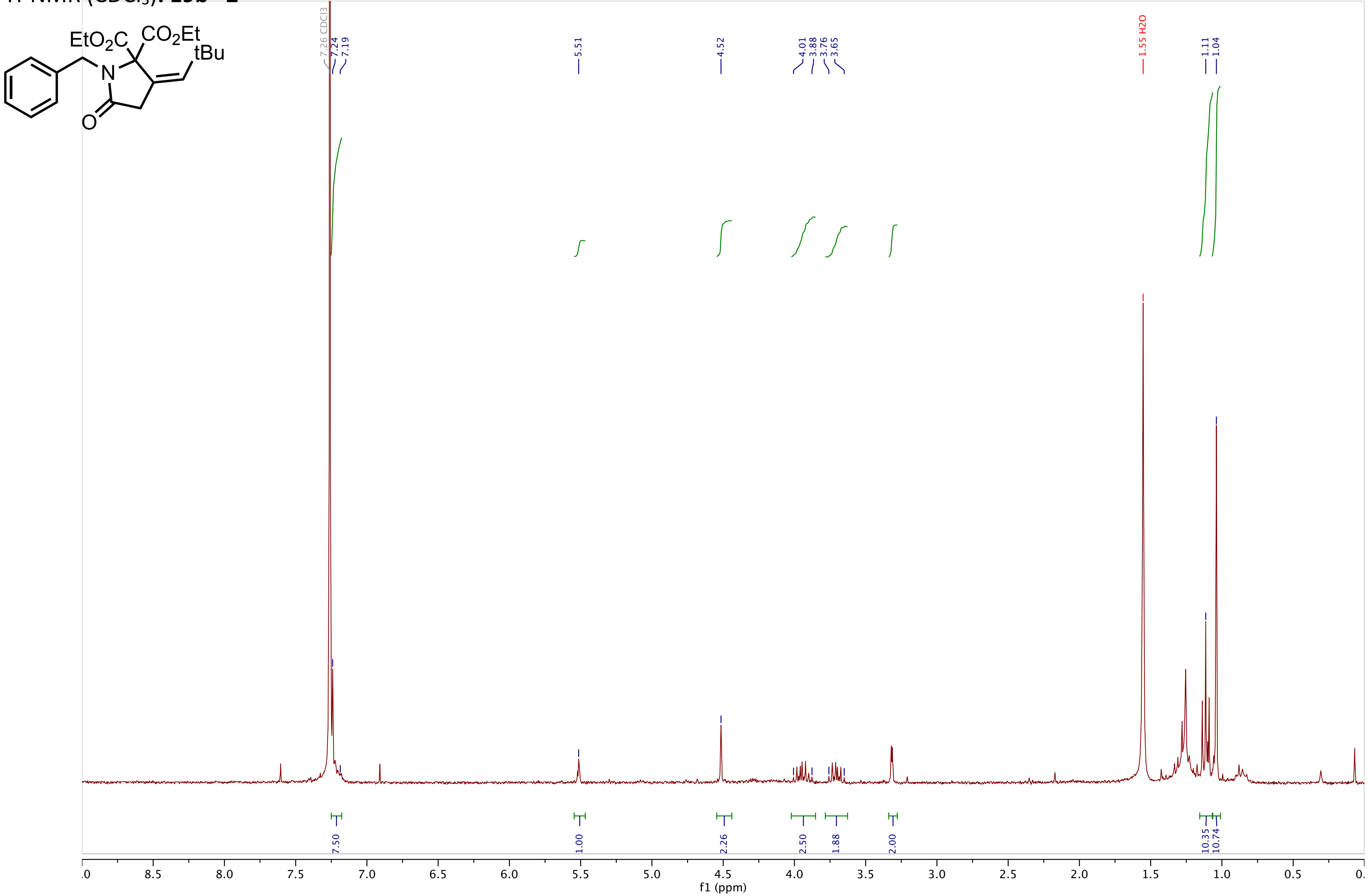

^{13}C-zgpg ($CDCl_3$): **19b - *Z***

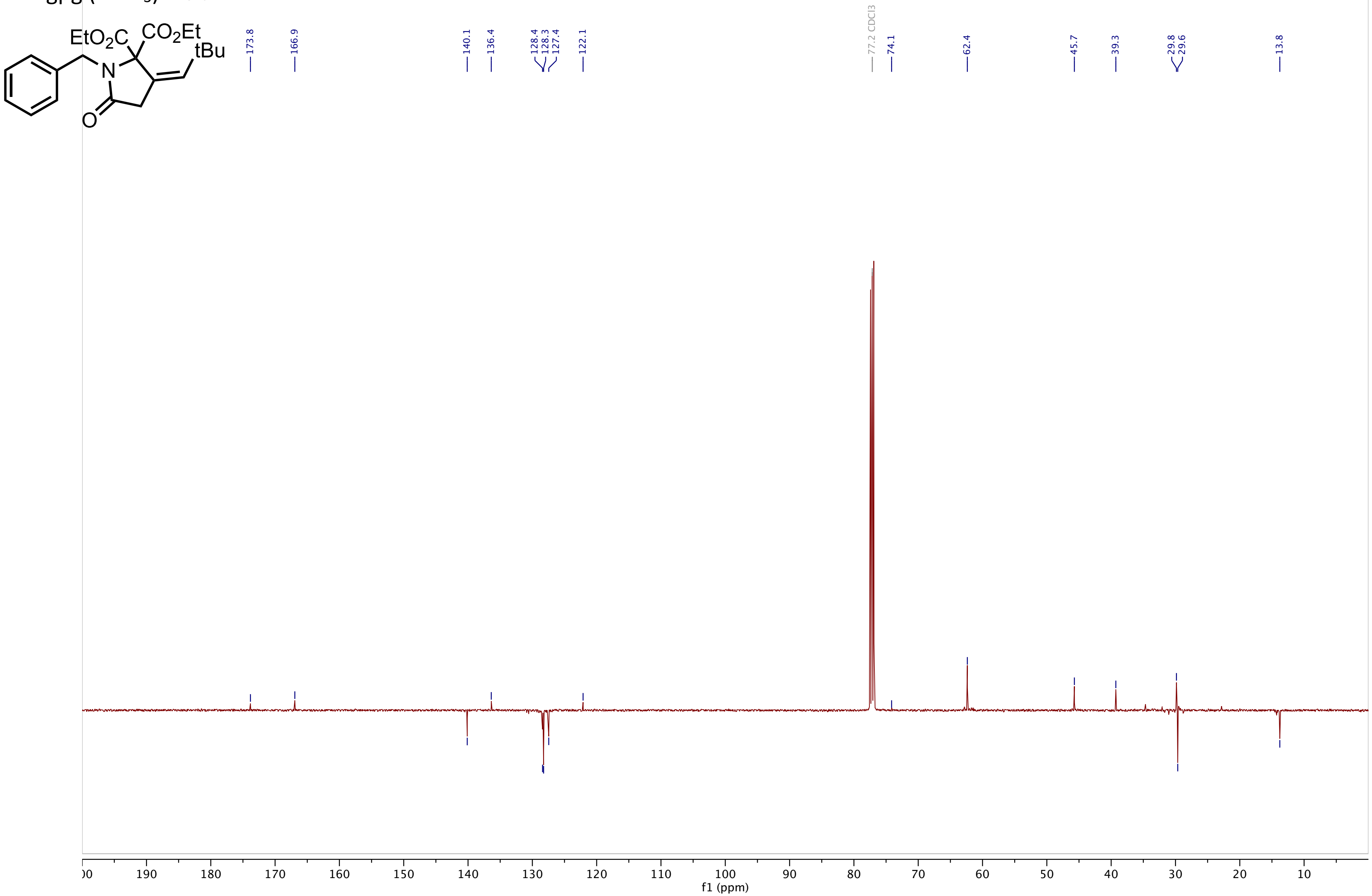

^{13}C-HSQC ($CDCl_3$): **19b - *Z***

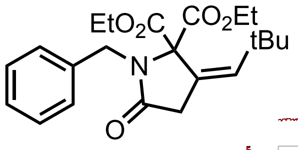

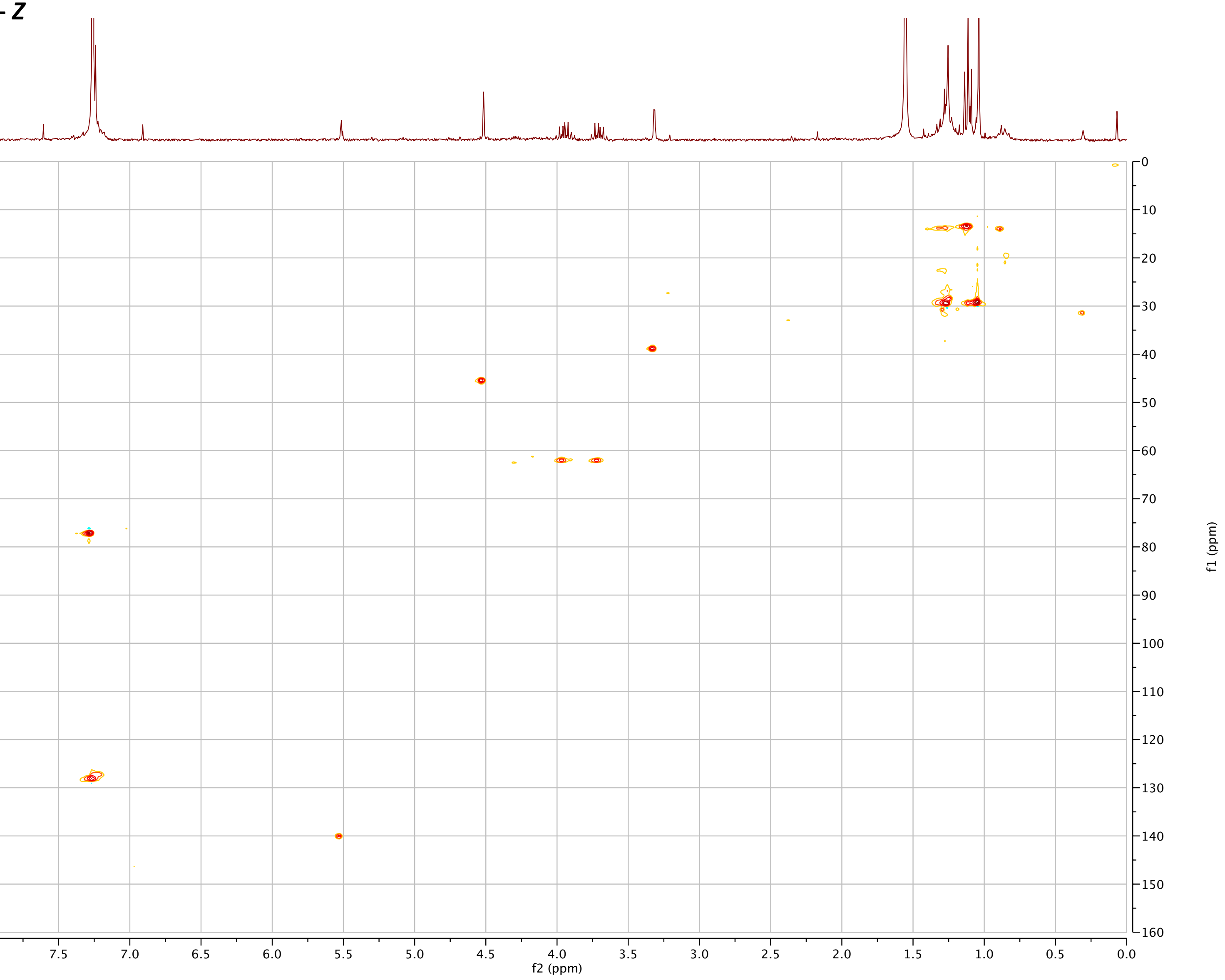

^{13}C-HMBC ($CDCl_3$): **19b - *Z***

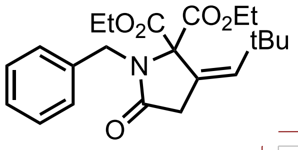

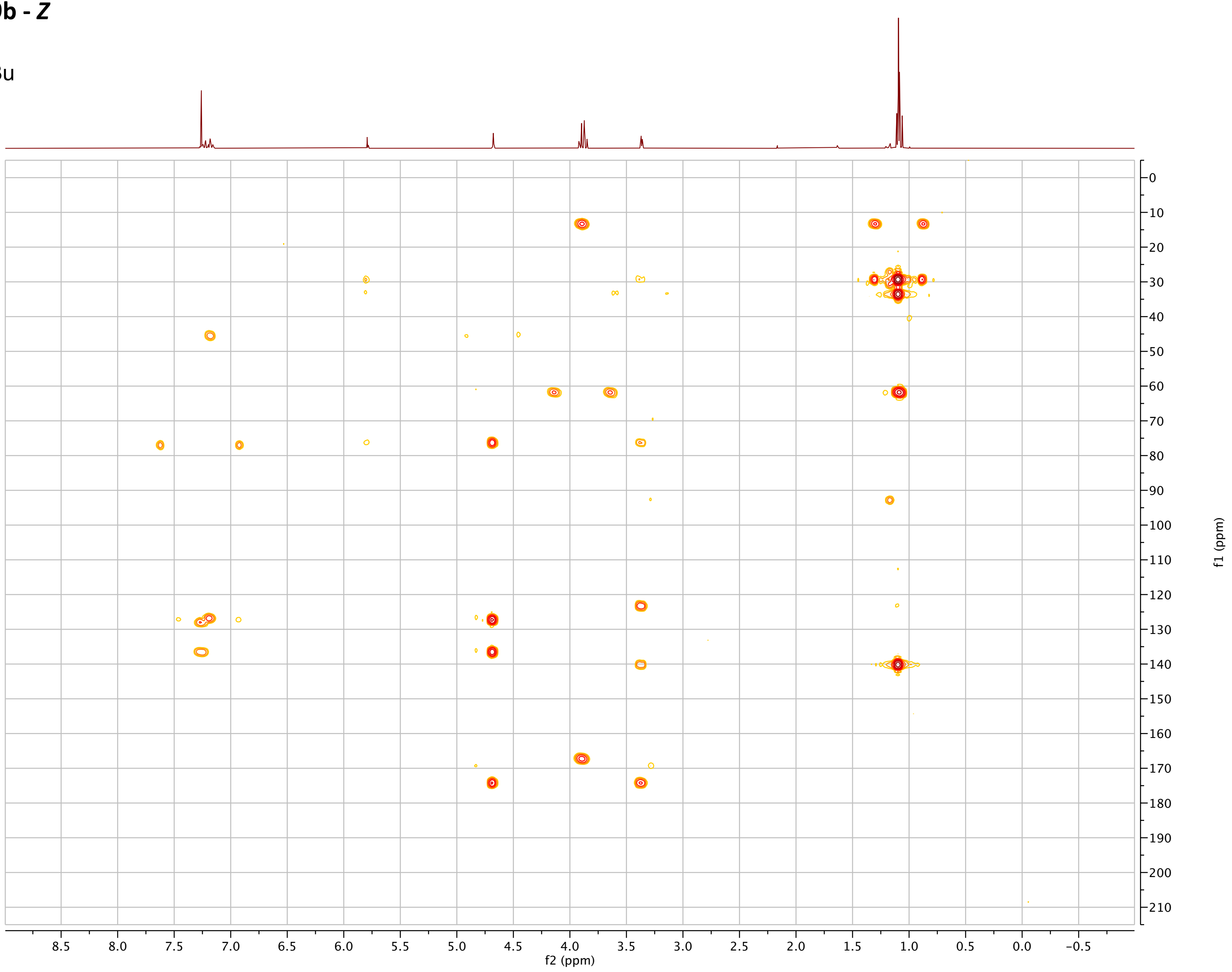

^{1}H-NOESY ($CDCl_3$): **19b - *Z***

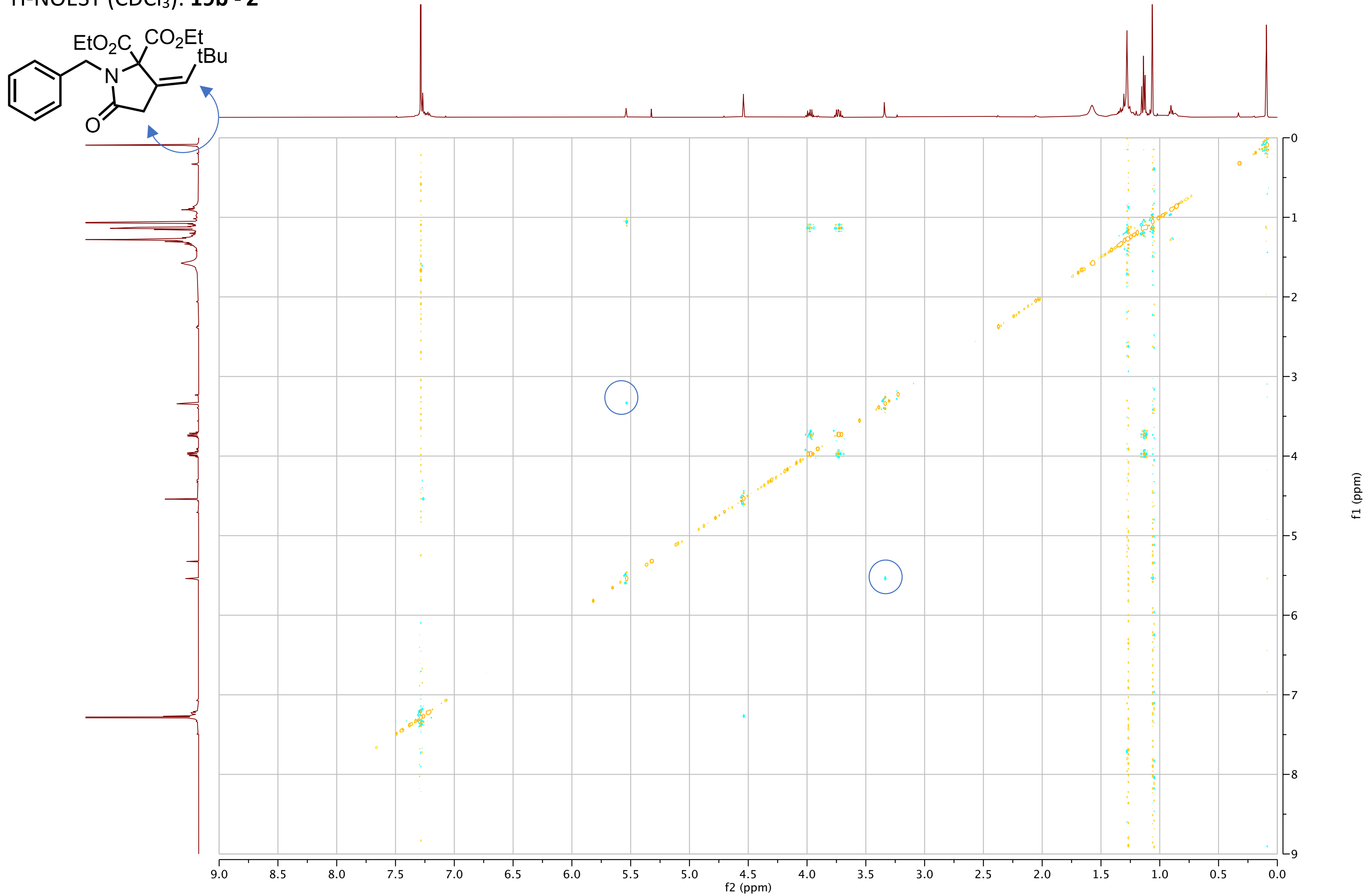

1D ^{1}H Selective ZQF Noesy ($CDCl_3$): **19b - *Z***. Ethylenic proton was selectively excited and the mixing time was 1 s.

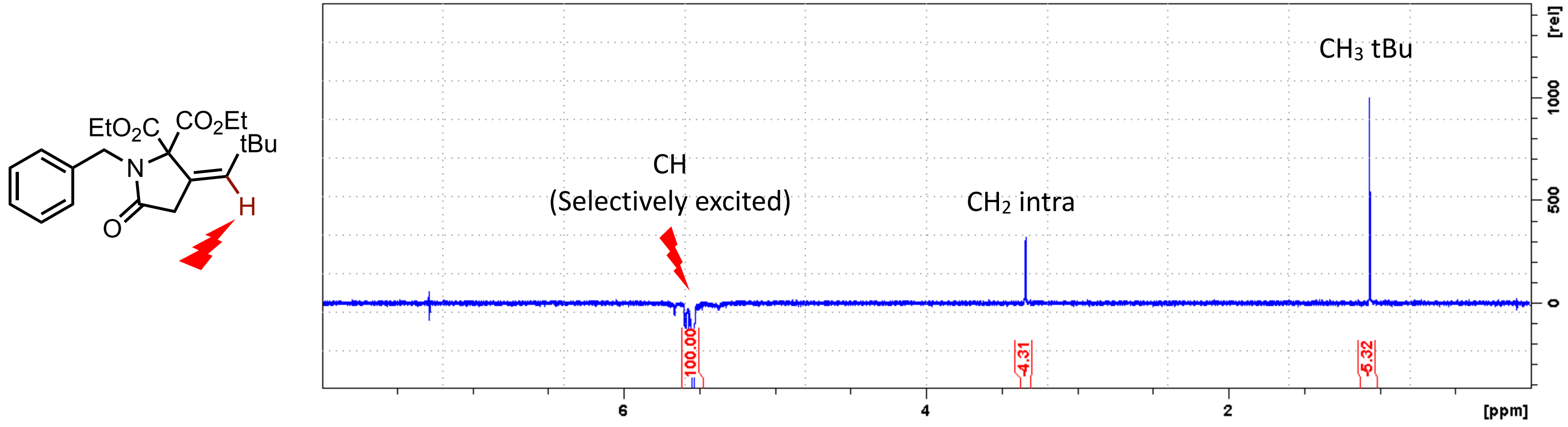

1D ^{1}H Selective ZQF Noesy ($CDCl_3$): **19b - *Z***. CH_2 intra protons were selectively excited and the mixing time was 1 s.

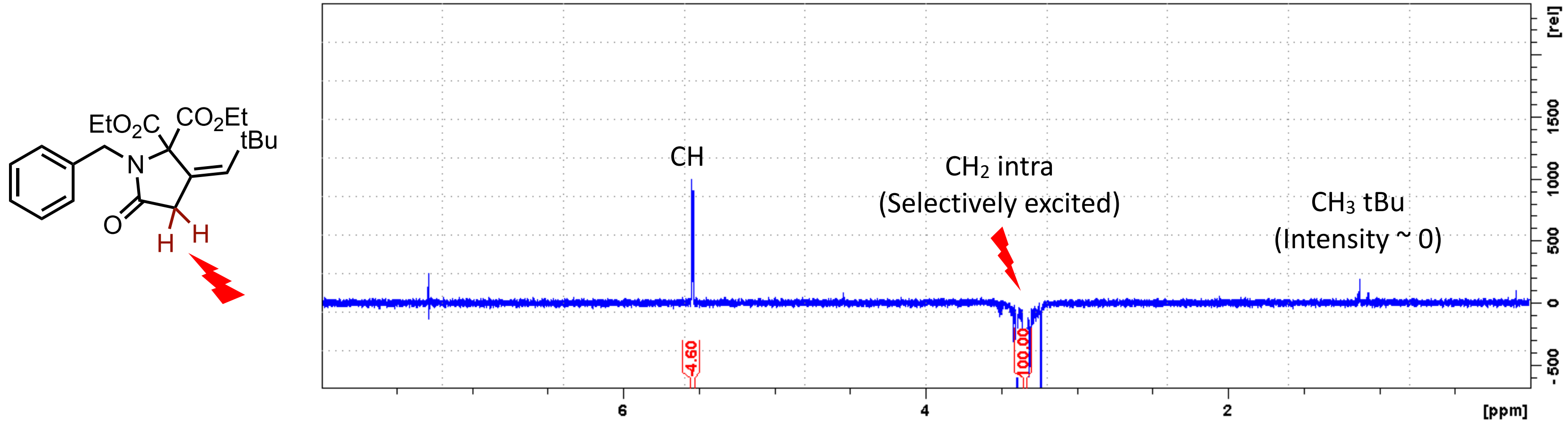

Double selective-HSQMBC-IPAP has been used to measured $^{3}J_{CH}$ heteronuclear coupling constant of the ethylenic bound.[20] From left to right, (A) In Phase (IP) 2D correlation, (B) Anti Phase (AP) 2D correlation, (C) ^{1}H 1D subspectra calculated by summing 2D correlation rows (blue and red curves stand for IP 2D correlation and AP 2D correlation sums of rows, respectively), (D) sum (blue curve) and subtraction (red curve) of IP and AP 1D ^{1}H subspectra.

Double selective-HSQMBC-IPAP correlation between ethylenic proton and carbon of the CH_2 intra for the compound **19b - *E***

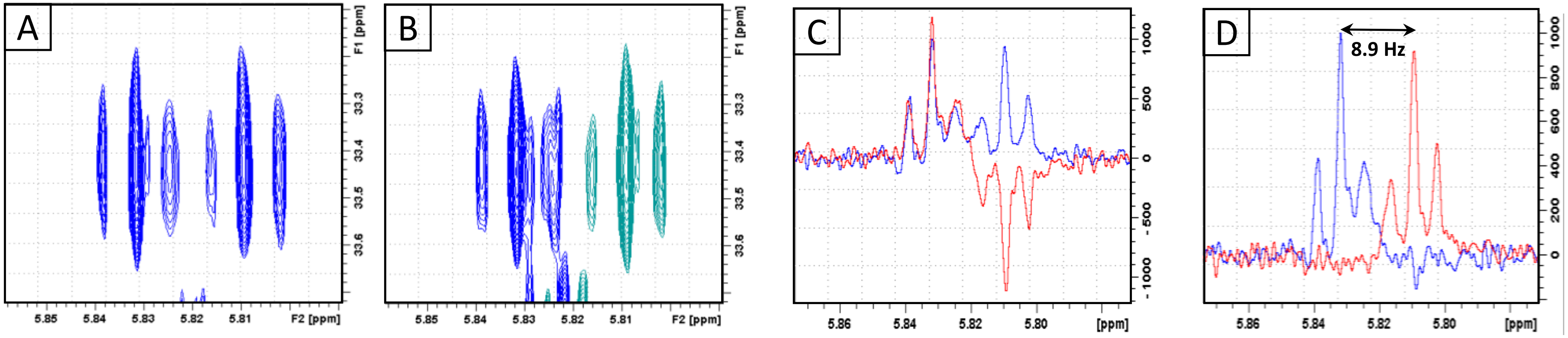

Double selective-HSQMBC-IPAP correlation between ethylenic proton and intra quartenary carbon for the compound **19b - *E***

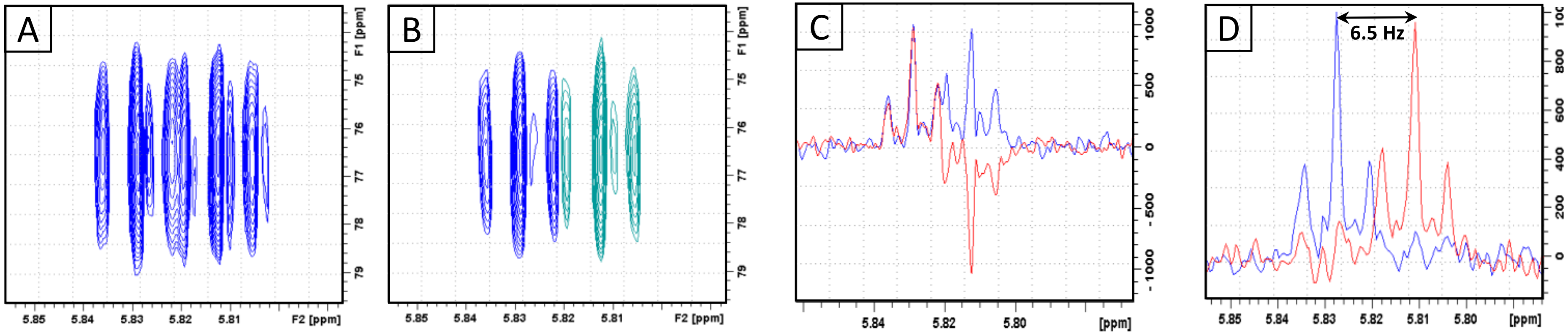

Double selective-HSQMBC-IPAP correlation between ethylenic proton and carbon of the CH_2 intra for the compound **19b - *Z***

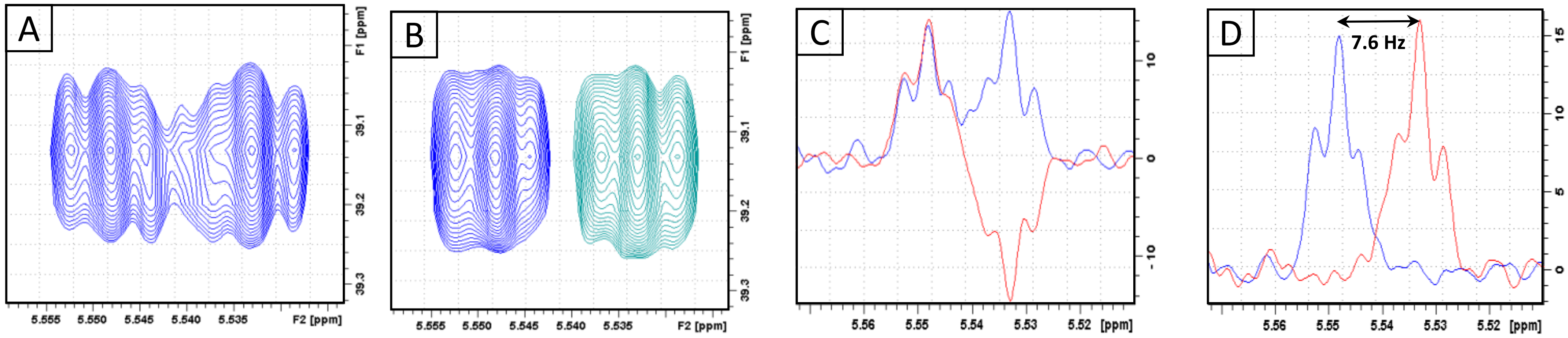

Double selective-HSQMBC-IPAP correlation between ethylenic proton and intra quartenary carbon for the compound **19b - *Z***

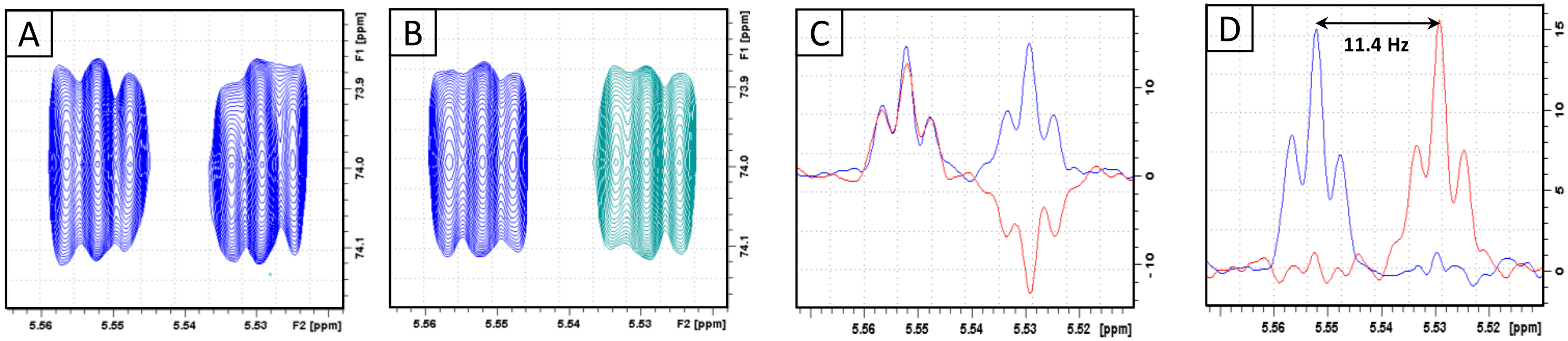

Supplement: SC-012-D0SC05036A-s001 [file SC-012-D0SC05036A-s001.pdf]
